# Incorporating a Polyethyleneglycol Linker to Enhance the Hydrophilicity of Mitochondria‐Targeted Triphenylphosphonium Constructs

**DOI:** 10.1002/cbic.202200774

**Published:** 2023-05-04

**Authors:** Shinpei Uno, Alexander H. Harkiss, Roy Chowdhury, Stuart T. Caldwell, Tracy A. Prime, Andrew M. James, Brendan Gallagher, Julien Prudent, Richard C. Hartley, Michael P. Murphy

**Affiliations:** ^1^ MRC Mitochondrial Biology Unit University of Cambridge Cambridge CB2 0XY UK; ^2^ School of Chemistry University of Glasgow Glasgow G12 8QQ UK; ^3^ Department of Medicine University of Cambridge Cambridge CB2 0QQ UK

**Keywords:** biological membrane, lipophilic cation, mitochondria-targeting, polyethylene glycol

## Abstract

The targeting of bioactive molecules and probes to mitochondria can be achieved by coupling to the lipophilic triphenyl phosphonium (TPP) cation, which accumulates several hundred‐fold within mitochondria in response to the mitochondrial membrane potential (Δψ_m_). Typically, a simple alkane links the TPP to its “cargo”, increasing overall hydrophobicity. As it would be beneficial to enhance the water solubility of mitochondria‐targeted compounds we explored the effects of replacing the alkyl linker with a polyethylene glycol (PEG). We found that the use of PEG led to compounds that were readily taken up by isolated mitochondria and by mitochondria inside cells. Within mitochondria the PEG linker greatly decreased adsorption of the TPP constructs to the matrix‐facing face of the mitochondrial inner membrane. These findings will allow the distribution of mitochondria‐targeted TPP compounds within mitochondria to be fine‐tuned.

## Graphical abstract

Triphenylphosphonium (TPP) cation is widely used for selective delivery of compounds into mitochondria. This study demonstrated that the replacement of an alkyl chain with polyethylene glycol (PEG) chain in TPP‐conjugated compounds can reduce the membrane adsorption of the compounds, leading to fine‐tuning of mitochondria targeting strategies.

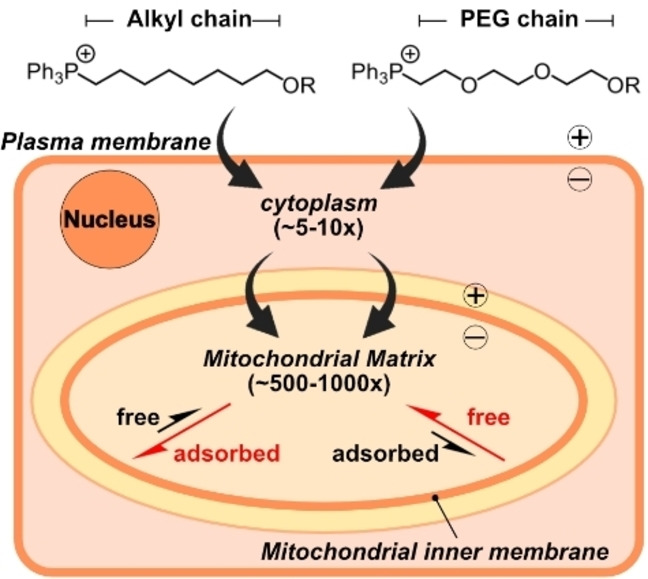

## Introduction

**Figure 1:**
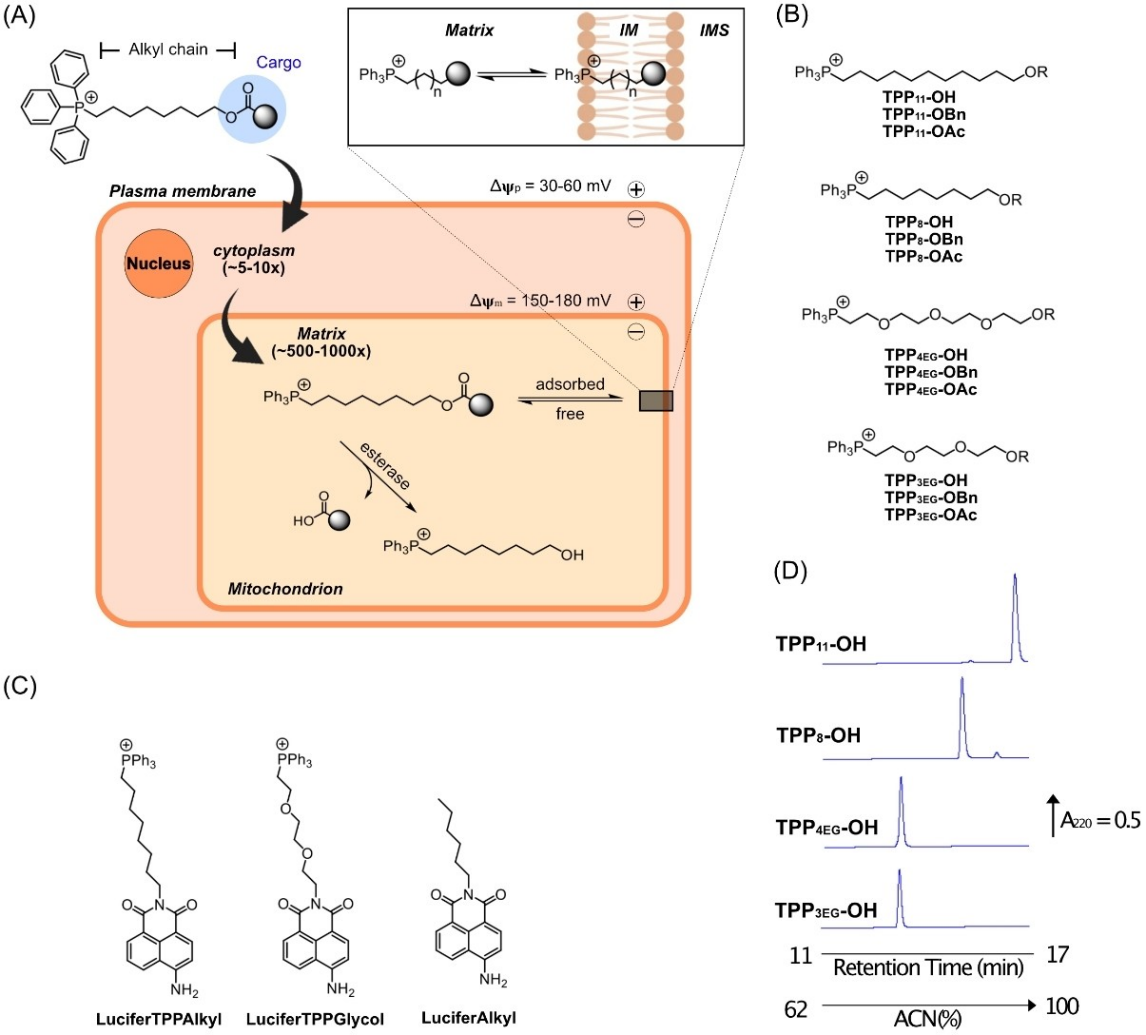
Mitochondria‐targeted delivery of cargoes conjugated to TPP compounds. (A) Uptake of a typical mitochondria‐targeted TPP compound. After accumulation into the cytoplasm driven by the plasma membrane potential (Δψ_p_) the compound is further taken up into the mitochondrial matrix in response to the mitochondrial membrane potential (Δψ_m_). Within the mitochondria matrix these compounds are largely adsorbed on to the matrix‐facing surface of mitochondrial inner membrane (Inset). Compounds can be designed to release bioactive cargo inside the mitochondria, for example by linkage via a cleavable ester bond. (B, C) The structures of the TPP compounds are shown. (D) Relative hydrophobicity of TPP compounds conjugated to a hydroxyl. Each compound (10 nmol) was analyzed by RP‐HPLC.

Mitochondrial function is central to many facets of cellular life, thus mitochondrial dysfunction is associated with a wide range of pathologies.^[1]^ Consequently, there is demand to develop mitochondria‐targeted molecules that can act as probes of mitochondrial activity or as a means of selectively delivering therapeutics to the organelle.[^1^, ^2^] Delivery vectors include peptides, metal complexes, nanocarriers and the attachment of small molecule ′cargos′ to lipophilic cations.^[3]^ The mechanism of uptake and the exact site of delivery may differ depending on the vector used. The mitochondrion is a complex organelle and localisation can occur by association to the outer or inner membranes, delivery to the intermembrane space between them, or targeting to the mitochondrial matrix. One approach to the delivery of active molecular moieties to the matrix of mitochondria within cells is by conjugation to the lipophilic triphenylphosphonium cation (TPP). The charge of these lipophilic cations is shielded by a large and hydrophobic surface area,[^4^, ^5^, ^6^] which lowers the activation energy for movement across phospholipid bilayers enabling their unmediated transport across biological membranes (Figure 1A), provided the cargo has molecular properties conducive to membrane permeability such as small size.^[7]^ The positive charge also causes uptake across biological membranes driven by the membrane potential (Δψ) in accordance with the Nernst equation, consequently lipophilic monocations accumulate ∼10‐fold for every ∼60 mV of Δψ .^[4]^ Therefore, TPP‐conjugates accumulate ∼3–10‐fold within cells driven by the plasma membrane potential (Δψ_p_; typically 30–60 mV, negative inside) and are then taken up within mitochondria a further 300–1000‐fold driven by the mitochondrial membrane potential (Δψ_m_; typically 150–180 mV, negative inside) (Figure 1A). These properties have enabled the development of a wide range of mitochondria‐targeted TPP small molecules that are rapidly and selectively accumulated within mitochondria inside cells both in culture and in vivo. For example, such compounds are used as probes to assess hydrogen peroxide, superoxide, Δψ_m_ and H_2_S, as means of selectively generating superoxide within mitochondria, to modulate mitochondrial thiol homeostasis, or as targeted antioxidants.[^6^, ^8^, ^9^, ^10^, ^11^, ^12^, ^13^, ^14^, ^15^] Of note, the mitochondria‐targeted antioxidant MitoQ has been developed as a potential therapeutic in humans, with several clinical trials reported and more ongoing.[^16^, ^17^, ^18^]

Typically, mitochondria‐targeted TPP compounds comprise a TPP cation linked by an alkyl chain to a bioactive “cargo” moiety (Figure 1A). The overall hydrophobicity of the construct is critical to enable rapid membrane permeation. For hydrophobic cargoes this is not problematic, for example with MitoE_2_ a minimal 2‐carbon link enables the mitochondrial uptake of the α‐tocopherol moiety.^[19]^ In contrast, for the delivery of polar cargoes, such as ascorbate or Paraquat, there was no mitochondrial uptake when a short alkyl chain was used, and a long, hydrophobic alkyl chain was required to balance cargo polarity.[^6^, ^15^] Furthermore, a spacer may be required to allow access to an active site: for MitoQ a 10‐carbon alkyl chain was required to enable the ubiquinone moiety to access the active site of succinate dehydrogenase within mitochondria for reduction to the active antioxidant;^[20]^ similarly, a 3‐carbon alkyl chain was insufficient to allow esterase‐catalyzed cleavage of TPP‐malonate ester conjugates, presumably due to steric interactions between the enzyme and the large TPP group, while an 11‐carobon chain allowed rapid cleavage.^[21]^ However, long, hydrophobic alkyl chains have drawbacks as they decrease water solubility and greatly enhance the binding of TPP compounds to albumin in the plasma and adsorption to the surface of phospholipid bilayers. For example, following uptake into the mitochondrial matrix long‐chain alkyl TPP molecules are largely adsorbed onto the matrix facing surface of the mitochondrial inner membrane (Figure 1A inset).^[22]^ This may be advantageous when a target protein is membrane‐bound, but could be counter productive if the target is sited in the aqueous phase of the matrix.

To expand the options available when designing mitochondria‐targeted TPP compounds it would be useful to tune the overall hydrophobicity of the construct in order to optimize water solubility, binding/membrane adsorption and length of the linker without compromising mitochondria‐targeting. One appealing possibility is to replace the hydrophobic alkyl chain with a more hydrophilic polyethylene glycol (PEG) chain, to enable tuning of the chain length and water solubility while retaining membrane permeation. PEG is hydrophilic and very water‐soluble^[23]^ Even so, low molecular weight PEG 400 comprised of **8**–**9** ethylene glycol units is able to permeate the intestinal gut.[^24^, ^25^] It has the advantages of being non‐toxic and metabolically stable and is widely used by the pharmaceutical industry^[24]^ As a result, short PEG linkers of **3**–**5** ethylene glycol units are widely used in molecular probes to provide a spacer and increase water‐solubility while allowing cell‐permeability. This has been particularly useful in proteolysis targeting chimaeras (PROTACs),^[26]^ which are being studied intensively because they allow the targeted degradation of proteins. Recent examples of PROTACs containing these short PEG linkers include those for degradation of proteins involved in cancer.[^27^, ^28^, ^29^] A TPP‐PEG linker has been incorporated into an atovaquone derivative,^[30]^ but how incorporation of a PEG chain affects the mitochondria‐targeting of TPP compounds is not yet known.

Here we have made a series of TPP‐conjugated compounds with a range of either alkyl or PEG linkers and assessed their uptake into isolated mitochondria and into mitochondria within cells. From this we conclude that TPP constructs which use a PEG linker to connect to the cargo are taken up by mitochondria within cells. Their rate of uptake is less than for alkyl constructs due to slower permeation across the plasma membrane. However, once inside mitochondria incorporation of a PEG linker greatly decreases adsorption of TPP constructs to the matrix‐facing face of the mitochondrial inner membrane providing greater exposure of the cargo to the aqueous phase.

## Results and Discussion

### Design and synthesis of TPP‐conjugated compounds

To explore the effect of replacing alkyl chains with PEG we prepared TPP compounds conjugated to various moieties by either 8 or 11 carbon alkyl chains, or the equivalent length PEG chains comprised of 3 or 4 ethylene glycol moieties (Figure 1B). The moieties conjugated to these constructs comprised a hydroxyl as a hydrophilic example, a benzyl as a hydrophobic version, and an acetyl ester to explore the effect of PEG incorporation on hydrolysis by esterases. In addition, we constructed an 8‐carbon alkyl link, or the equivalent PEG chain, between the fluorophore Lucifer yellow and TPP so as to visualize their uptake into living cells by fluorescence microscopy (Figure 1C).

To compare the effect of the PEG vs alkyl chain on relative hydrophobicity, we subjected the hydroxyl derivatives to RP‐HPLC and compared their retention times (Figure 1D). As expected, the alkyl conjugates had longer retention times than the PEG constructs, and TPP_11_‐OH eluted more slowly than TPP_8_‐OH. The retention time of TPP_4EG_‐OH was similar to that of TPP_3EG_‐OH (Figure 1D), but analysis of a mixture showed that TPP_4EG_‐OH had a slightly longer retention time than TPP_3EG_‐OH (Figure S1). To further assess their relative hydrophobicity, we calculated the logP of the linkers and cargoes of these compounds using a consensus model built on Chem‐axon and developed from Klopman et al.^[31]^ using the PHYSPROP database (Table 1). The TPP group can be assumed to have similar physicochemical properties in all conjugates and calculating the linker and cargo without the TPP group simplifies the calculation and avoids complications associated with the modelling of logPs of single ions.[^32^, ^33^] Thus, we have a series of TPP compounds with side chains spanning a range of hydrophobicities in the order: TPP_11_‐OH>TPP_8_‐OH>TPP_4EG_‐OH>TPP_3EG_‐OH.

**Table 1:** Calculation of sidechain logP. Each log P value was obtained using software MarvinSketch. Corresponding name of TPP‐conjugated compounds are written in each column.

Head group	Chemical structure (Compound name)	LogP
Hydroxyl	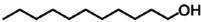	3.42
	TPP_11_‐OH	
	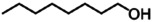	2.23
	TPP_8_‐OH	
	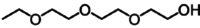	−0.72
	TPP_4EG_‐OH	
	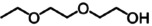	−0.55
	TPP_3EG_‐OH	
Benzyl	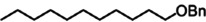	5.77
	TPP_11_‐OBn	
	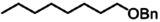	4.58
	TPP_8_‐OBn	
	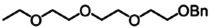	1.64
	TPP_4EG_‐OBn	
	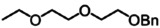	1.80
	TPP_3EG_‐OBn	
Acetyl	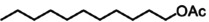	3.85
	TPP_11_‐OAc	
	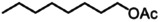	2.66
	TPP_8_‐OAc	
	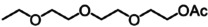	−0.29
	TPP_4EG_‐OAc	
	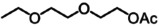	−0.12
	TPP_3EG_‐OAc	

### Δψ_m_‐dependent uptake of compounds into isolated mitochondria

**Figure 2:**
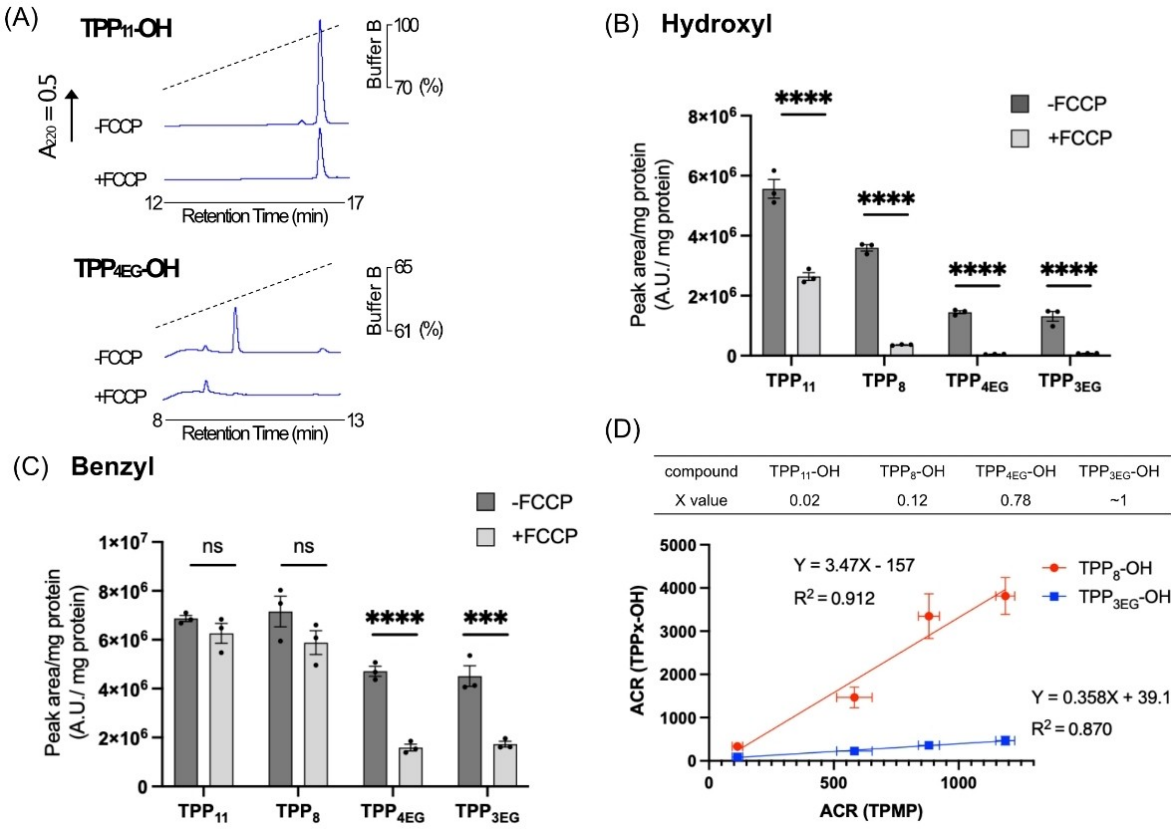
Uptake of TPP‐conjugated compounds by energized mitochondria. Compounds (10 μM) were incubated with energized rat liver mitochondria (2 mg protein/mL)±FCCP (0.5 μM) for 5 min before pelleting the mitochondria by centrifugation, extracting the pellet, and quantifying the amount of compound present by RP‐HPLC. (A) Representative RP‐HPLC traces from incubations with TPP_11_‐OH or TPP_4EG_‐OH±FCCP (B, C) Quantification of peak areas of hydroxyl derivatives (B) and benzyl derivatives (C). (D) Accumulation ratios (ACRs) of TPP_8_‐OH and TPP_3EG_‐OH relative to that of TPMP. Mitochondria were incubated with TPP_8_‐OH or TPP_3EG_‐OH (10 μM) along with TPMP (3 μM) and different concentrations of FCCP (0–0.5 μM) to establish a range of Δψ_m_ values. The mitochondria and supernatants were then isolated and analyzed by RP‐HPLC to indicate the relative concentrations of the compounds in the two compartments, and from the ACR values were calculated. Data are means±SEM of three independent experiments. Statistical significance was assessed by two‐way ANOVA with Dunnett's correction for multiple comparisons; ***p<0.01, ****p<0.001.

To see how PEG incorporation affected Δψ_m_‐dependent uptake of these compounds into mitochondria, we incubated them with isolated, energized rat liver mitochondria for 5 min, then pelleted the mitochondria, extracted the accumulated compounds and assessed their uptake into mitochondria by RP‐HPLC. Figure 2A shows the representative trace of TPP_11_‐OH and its PEG equivalent, TPP_4EG_‐OH. It can be seen that both TPP_11_‐OH and TPP_4EG_‐OH were accumulated within mitochondria and that abolition of Δψ_m_ by addition of the mitochondrial uncoupler FCCP decreased their uptake (Figure 2A). The peak areas for the amounts of −OH and the ‐Bn derivatives accumulated within mitochondria±FCCP were then determined (Figure 2B and C). For the −OH derivatives all were taken up into energized mitochondria and uptake was largely or partially prevented by abolishing Δψ_m_ with the uncoupler FCCP. However, even in the presence of FCCP there was still considerable association of TPP_11_‐OH and TPP_8_‐OH with the mitochondria. In contrast, there was no such association of the equivalent PEG analogs TPP_4EG_ and TPP_3EG_ to uncoupled mitochondria. This pattern was more evident for the hydrophobic benzyl derivatives where the association of TPP_11_‐OBn and TPP_8_‐OBn with mitochondria was unchanged by FCCP, while for both PEG Bn derivatives, uptake was decreased, but not abolished, by FCCP (Figure 2C). These findings are consistent with extensive adsorption of alkyl TPP compounds to the surface of phospholipid bilayers,^[4]^ while that of the TPP‐PEG derivatives is significantly decreased.

A large proportion of TPP compounds accumulated within the mitochondrial matrix is adsorbed to the matrix facing surface of the mitochondrial inner membrane.^[34]^ To see if this was affected by the decreased adsorption of the PEG derivatives to phospholipid bilayers, we quantified the relative adsorption of alkyl and PEG derivatives. To do this we measured the accumulation ratios (ACRs) of these compounds over a range of Δψ_m_ values. The ACR is the ratio of the concentration of the compound in the mitochondria to that in the external medium (ACR, [compound]_mitochondria_/[compound]_external medium_). The ACR of mono cations is related to the Δψ_m_ by the Nernst equation [Eq. 1]

(1) $$\Delta_{\psi_{m}}\frac{\mathrm{RT}}{F}\cdot \mathrm{Ln}\left[x\left(\mathrm{ACR}\right)\right]$$

In the absence of adsorption to the matrix‐facing surface of the inner membrane x=1 and as adsorption increases, x decreases. For example, about 60 % of methyltriphenylphosphonium (TPMP) accumulated within mitochondria is adsorbed, hence x=0.4^[35]^ Thus, determining x enables us to infer the relative membrane adsorption of a compound. To do this, we incubated TPP_8_‐OH or TPP_3EG_‐OH, with TPMP over a range of Δψ_m_ values, set by using different concentrations of FCCP. We then quantified the ACRs by measuring their amounts in the mitochondrial pellet and supernatant, normalized to their volumes (0.6 μL/mg protein for the mitochondrial matrix and 2 mL for the supernatant^[36]^). This enabled us to assess the ACR of the compound and that of TPMP simultaneously over a range of Δψ_m_ values (Figure 2D and S2).[^4^, ^34^] These plots gave straight lines, consistent with the distribution of these compounds equilibrating with Δψ_m_ across a range of values. The ACR for TPP_8_‐OH was considerably larger than that for TPP_3EG_‐OH, consistent with its far greater membrane adsorption. We calculated their membrane adsorption from these plots (Table in Figure 2D). The slopes of these plots for TPP_8_‐OH and TPP_3EG_‐OH are ∼3.5 and ∼0.36, respectively. As x=0.4 for TPMP, this implies that for TPP_8_‐OH x=0.12, indicating that 88 % of TPP_8_‐OH within the mitochondrial matrix is adsorbed to the surface of the inner membrane. In contrast, for TPP_3EG_‐OH x∼1, suggesting that the PEG chain prevents the additional membrane adsorption within the mitochondrial matrix caused by an alkyl linker. Thus, a PEG linker greatly decreases the membrane association of TPP compounds compared to an alkyl link, and in particular lowers the proportion of compound within the mitochondrial matrix adsorbed to the inner membrane.

### Real‐time assessment of mitochondrial uptake using a TPP^+^‐selective electrode

**Figure 3:**
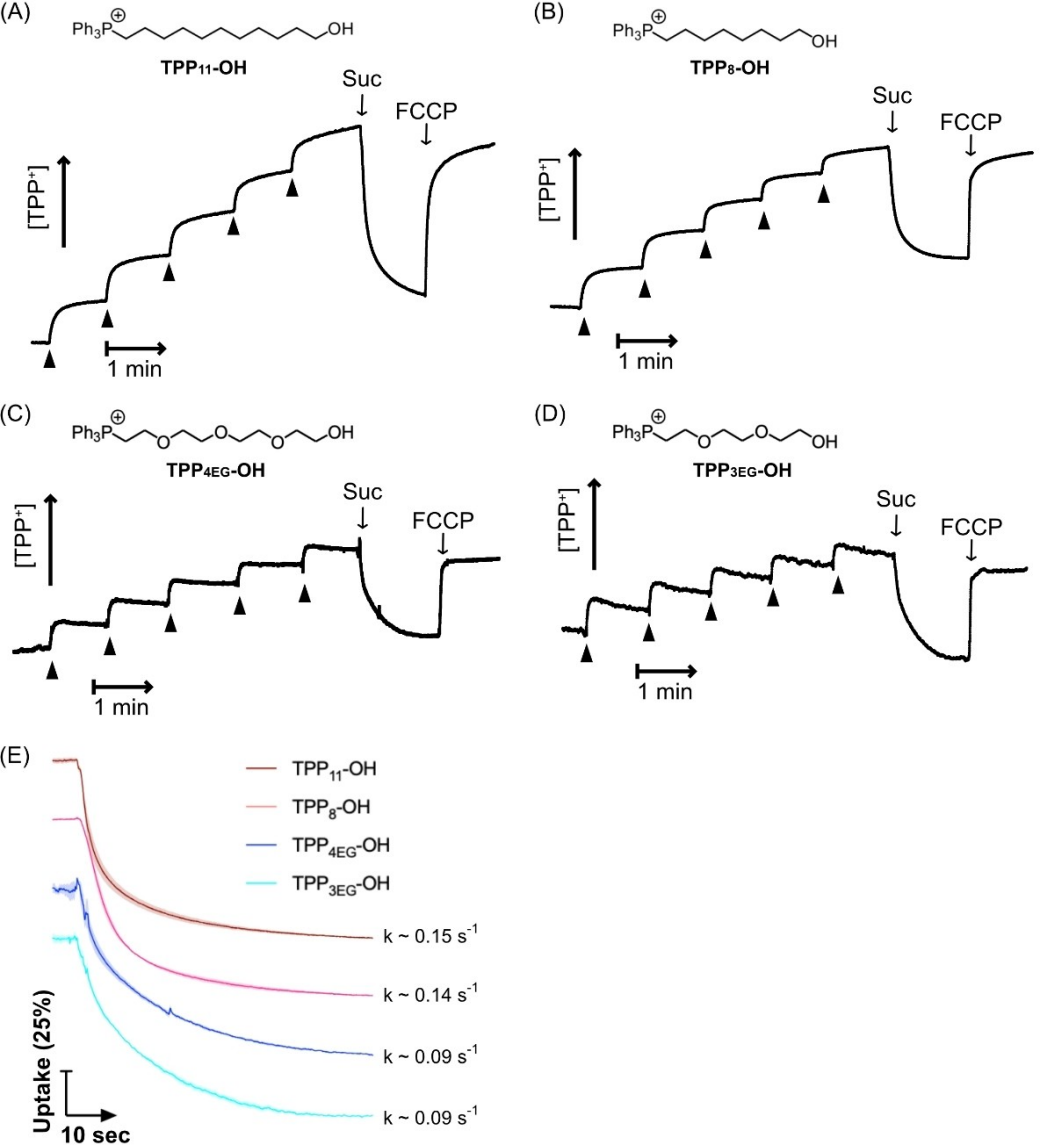
Real‐time measurement of uptake of TPP‐conjugated compounds by energized mitochondria. After addition of rat liver mitochondria (2 mg protein/mL) to the electrode chamber, electrode response was calibrated by 5×1 μM additions of the TPP compound. Mitochondria were then energized by addition of succinate (10 mM) and where indicated FCCP (0.5 μM) was added. (A) TPP_11_‐OH, (B) TPP_8_‐OH, (C) TPP_4EG_‐OH, and (D) TPP_3EG_‐OH. Data are typical traces repeated at least 3 times. (E) Normalized compound uptake. The Ln(electrode response) was divided by the difference between the Ln response at t=0 (0 % uptake) and that at t=60 (100 % uptake) to generate plots of uptake as % of maximum over time. Data are shown for 60 seconds after the addition of succinate (t=0). Data are means±SEM (shading) of three independent experiments and traces are offset for clarity. Pseudo first order rate constants (k) for compound uptake were estimated from the slopes of plots of the Ln values of the normalized uptake data against time over the first 10s of uptake.

The rate of transit of TPP compounds across the hydrophobic core of the mitochondrial inner membrane is decreased by hydrophilicity.^[34]^ The analysis in Figure 2 showed the Δψ_m_‐dependent uptake of both alkyl and PEG‐linked TPP molecules into isolated mitochondria. However, uptake was measured after the molecules had equilibrated and thus their rate of uptake may be different. To see if incorporation of the PEG chain affected the rate of uptake into mitochondria we measured uptake in real time using an ion‐selective electrode that was sensitive to the TPP moiety (Figures 3A–D).^[22]^ We first added mitochondria to the electrode chamber in the presence of rotenone to prevent generation of Δψ_m_, and calibrated the electrode response by titrating the TPP‐conjugated compounds. Succinate was then added to generate Δψ_m_, leading to rapid compound uptake, which was reversed by addition of the uncoupler FCCP. These experiments confirmed the Δψ_m_‐dependent uptake by mitochondria of all these compounds (Figure 3A–D). The electrode response is dependent on compound hydrophobicity,^[22]^ and it is not suitable to compare their uptake simply by comparison of electrode response. However, in each case the electrode response is proportional to Ln[compound],^[37]^ enabling us to express compound uptake as a % of total uptake at equilibrium and thereby compare their rates of uptake (Figure 3E). This showed semi‐qualitatively that the uptake of the PEG compounds was slightly slower than that of the alkyl‐linked compounds. The times required to reach 75 % of maximum uptake are: ∼10s for TPP_11_‐OH and TPP_8_‐OH; ∼18s for TPP_4EG_‐OH and TPP_3EG_‐OH. To extend this analysis we focused on the initial uptake of the compounds into mitochondria following energization, and by assuming that initial uptake was pseudo first order in [compound], estimated a first order rate constant for uptake (Figure 3E). This showed that the pseudo first order rate constant for the PEG analogs was slower by ∼60 % than that of the alkyl‐linked compounds. We conclude that the replacement of an alkyl chain with a PEG chain slows, but does not prevent, the passage of a TPP conjugate through the mitochondrial inner membrane.

### The effect of linker composition on hydrolysis by esterases

**Figure 4:**
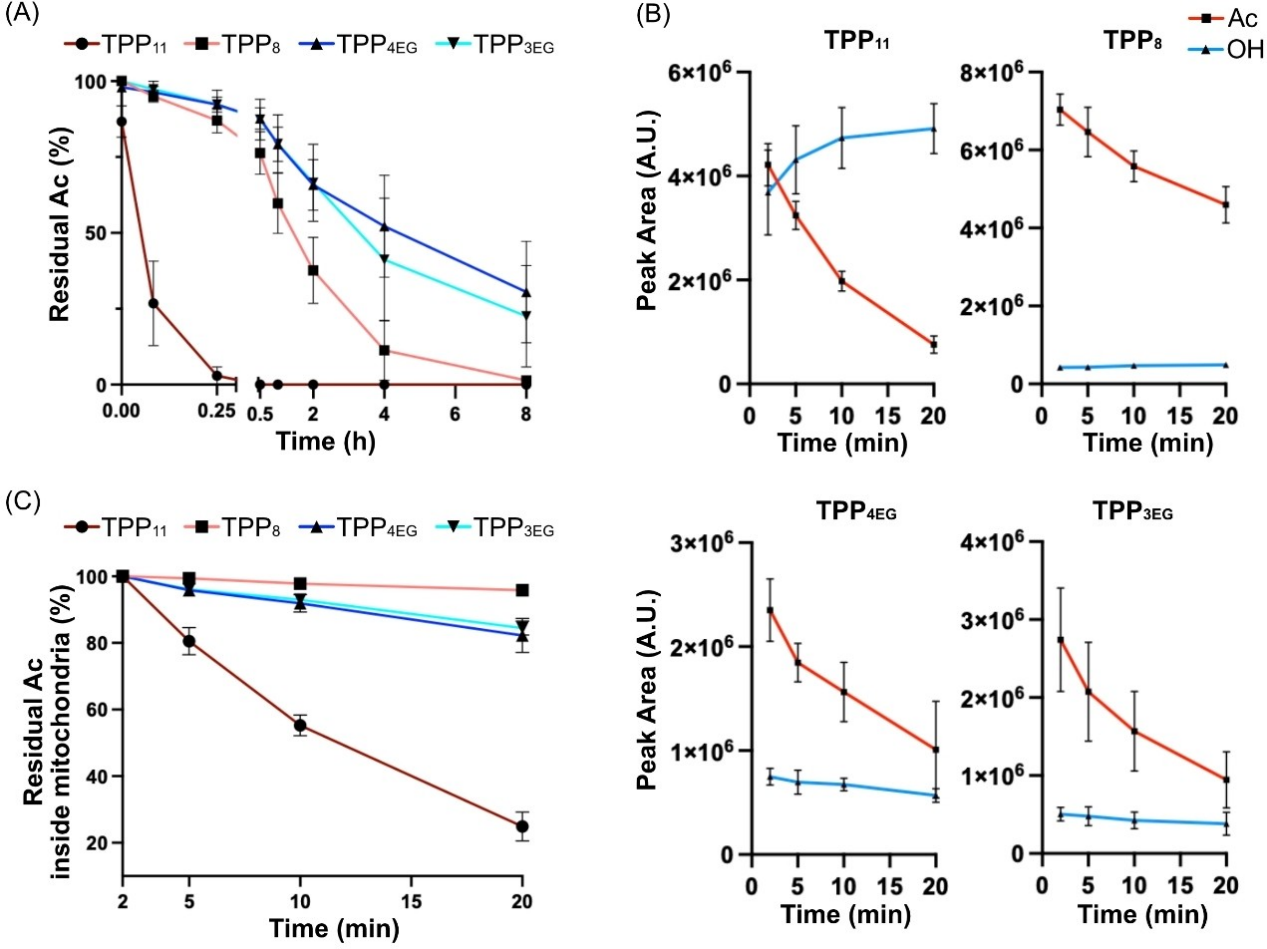
Enzymatic hydrolysis of TPP‐conjugated acetyl esters. (A) TPP acetyl esters (200 μM) were incubated with rat liver cytosol (1 mg protein/mL) for various times before extraction and analysis by RP‐HPLC. (B) TPP acetyl esters (10 μM) and internal standard TPMP (3 μM) were incubated with rat heart mitochondria (0.5 mg protein/mL) before pelleting mitochondria, extracting the pellets and analyzing by RP‐HPLC. The data show the peak areas of the ester and hydroxyl compounds within mitochondria. (C) Hydrolysis rates of the TPP acetyl esters within mitochondria. Data from the experiments described in Panel B were analyzed at each time point by dividing the peak areas of residual acetyl derivatives of the acetyl esters by that of the sum of the peak areas of the acetyl esters and its hydroxyl derivatives. These values are expressed as % of amount present at 2 min. All data are means±SEM of three independent experiments.

TPP compounds can be conjugated to a cargo via a cleavable linker designed to release the targeted compound within mitochondria (Figure 1A).[^21^, ^38^] This is often achieved with an ester linkage that is cleaved by intramitochondrial esterases, therefore we next assessed if a PEG linker could disrupt this process. To do this, the acetyl esters were incubated with a rat liver cytosol fraction and the rate of enzymatic ester hydrolysis was assessed by RP‐HPLC (Figure 4A). TPP_11_‐OAc was very rapidly hydrolyzed, and faster than TPP_8_‐OAc. Hydrolysis of TPP_4EG_‐OAc and TPP_3EG_‐OAc was slower than either of the alkyl‐TPPs. Thus, relative susceptibility to enzymatic esterase cleavage is: TPP_11_≫TPP_8_>TPP_3EG_>TPP_4EG_.

To assess enzymatic cleavage of these esters within mitochondria, we incubated the compounds with rat heart mitochondria (contaminating extra‐mitochondrial esterases makes this impossible with liver mitochondria^[21]^). We incubated the compounds with mitochondria and measured the mitochondrial content of the esters and of their hydrolysis products over time (Figure 4B). In parallel, we quantified the amounts of these compounds in the supernatant after pelleting the mitochondria over time (Figure S3). Within mitochondria the content of the acetyl derivatives decreased over time, while in the supernatant their levels remained relatively stable. Within mitochondria the hydrolysis product (−OH) increased or stayed stable, while increasing in the supernatant. We also calculated the decrease in the mitochondrial content of the acetyl derivatives over time, where the amount of ester present at 2 min was taken as 100 % and that at indicated time point was expressed as a % of this value (Figure 4C). The order of ester cleavage rate within mitochondria, TPP_11_≫TPP_4EG_>TPP_3EG_>TPP_8_, is different from that of cytosolic esterases shown in Figure 4A. The reason for this difference is beyond the scope of this work.

### Uptake of TPP‐conjugated fluorophores by mitochondria within cells

We next assessed the effect of replacing an alkyl chain with PEG on the uptake of TPP conjugates into mitochondria within living cells. To enable visualization of cell uptake, we synthesized two compounds, LuciferTPPAlkyl and LuciferTPPGlycol, both of which contain the fluorescent Lucifer Yellow moiety (Figure 1C). These compounds were accumulated readily by isolated energized mitochondria, but the greater hydrophobicity of the LuciferTPPAlkyl compound meant its association with mitochondria was unaffected by uncoupling with FCCP, while uptake of LuciferTPPGlycol was partially decreased by dissipating the membrane potential (Figure S4A).

**Figure 5:**
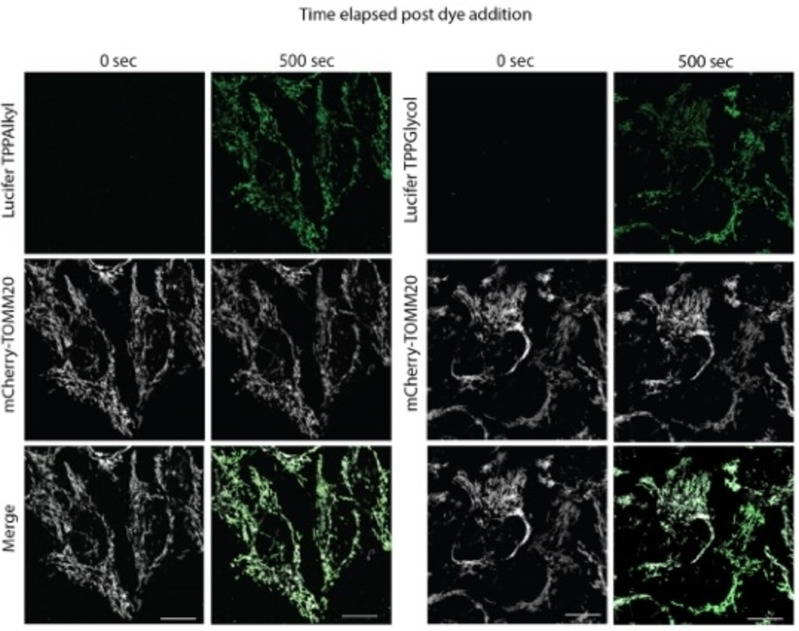
The uptake of LuciferTPPAlkyl and LuciferTPPGlycol by mitochondria within cells. HeLa cells stably expressing the mitochondrial outer membrane protein TOMM20 tagged with the fluorescent protein mCherry (HeLa‐TOMM20‐mCh) (white) were used to visualize mitochondria. The fluorescence of Lucifer Yellow (green) was observed here 500s after the addition of 250 nM of either compound. Scale bars=15 μm.

**Figure 6:**
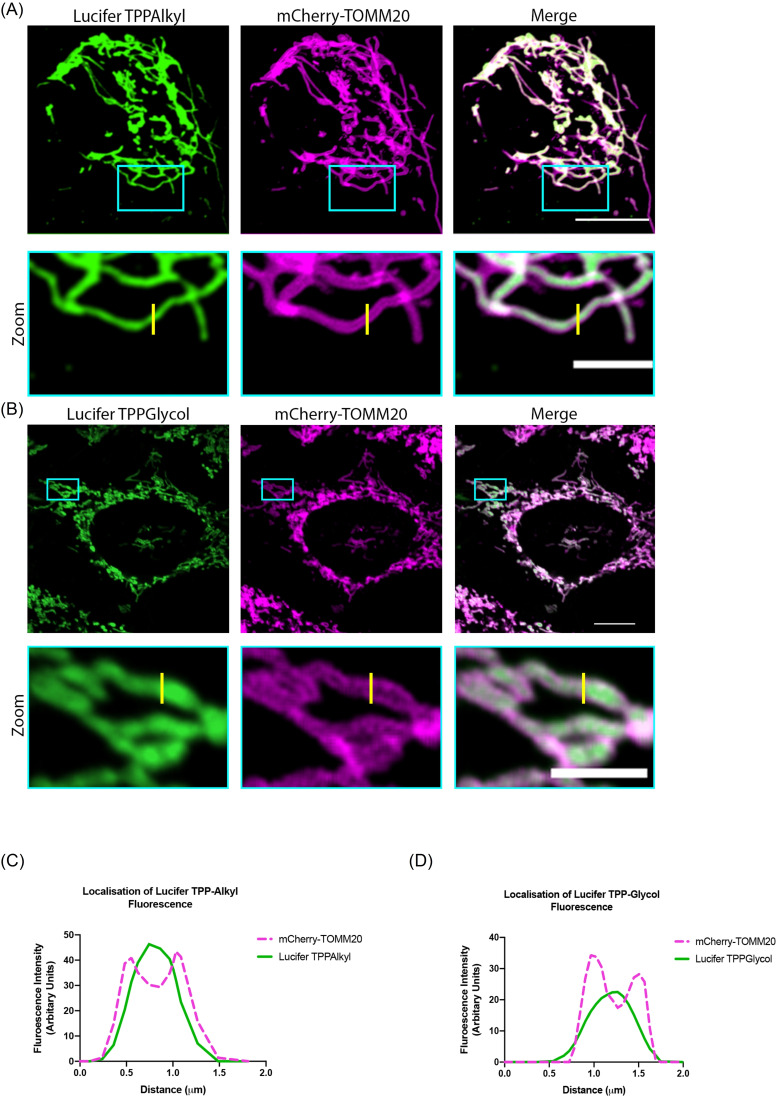
Distribution of LuciferTPPAlkyl and LuciferTPPGlycol. HeLa cells stably expressing the mitochondrial outer membrane protein TOMM20 tagged with the fluorescent protein mCherry (HeLa‐mCherry‐TOMM20) were used to visualize mitochondria (magenta). Fluorescence (green) from LuciferTPPAlkyl (A) and LuciferTPPGlycol (B) was observed within mitochondrial matrix after incubating the cells for 500s with either compound (250 nM). Fluorescence intensity values (arbitrary unit) for mCherry‐TOMM20 and the respective dyes were measured along the bisecting line (2 μm; yellow) and plotted against distance (μm) in panels (C) and (D) for LuciferTPPAlkyl and LuciferTPPGlycol, respectively. Scale bar=15 μm; Scale bar for zoom=5 μm.

We next assessed the uptake of these compounds into mitochondria within cells by live cell confocal microscopy (Figure 5, Movies 1–4). To investigate if LuciferTPPAlkyl and LuciferTPPGlycol entered mitochondria within cells, we used HeLa cells stably expressing the mitochondrial outer membrane protein Tomm20 tagged with the fluorescent protein mCherry (HeLa‐TOMM20‐mCh, white) to visualize mitochondria (Figure 5). The fluorescence of Lucifer Yellow (green) was observed after the addition of compound and colocalized with the fluorescence of TOMM20‐mCh, indicating that both LuciferTPP compounds were targeted to mitochondria (Figure 5), while the untargeted LuciferAlkyl (Figure 1C) distributed throughout the whole cell (Figure S4B). In depth live cell microscopy and fluorescence line‐scan analyses revealed that LuciferTPPAlkyl (Figure 6A, C) and LuciferTPPGlycol (Figure 6B, D) selectively accumulated within mitochondria from living cells.

**Figure 7:**
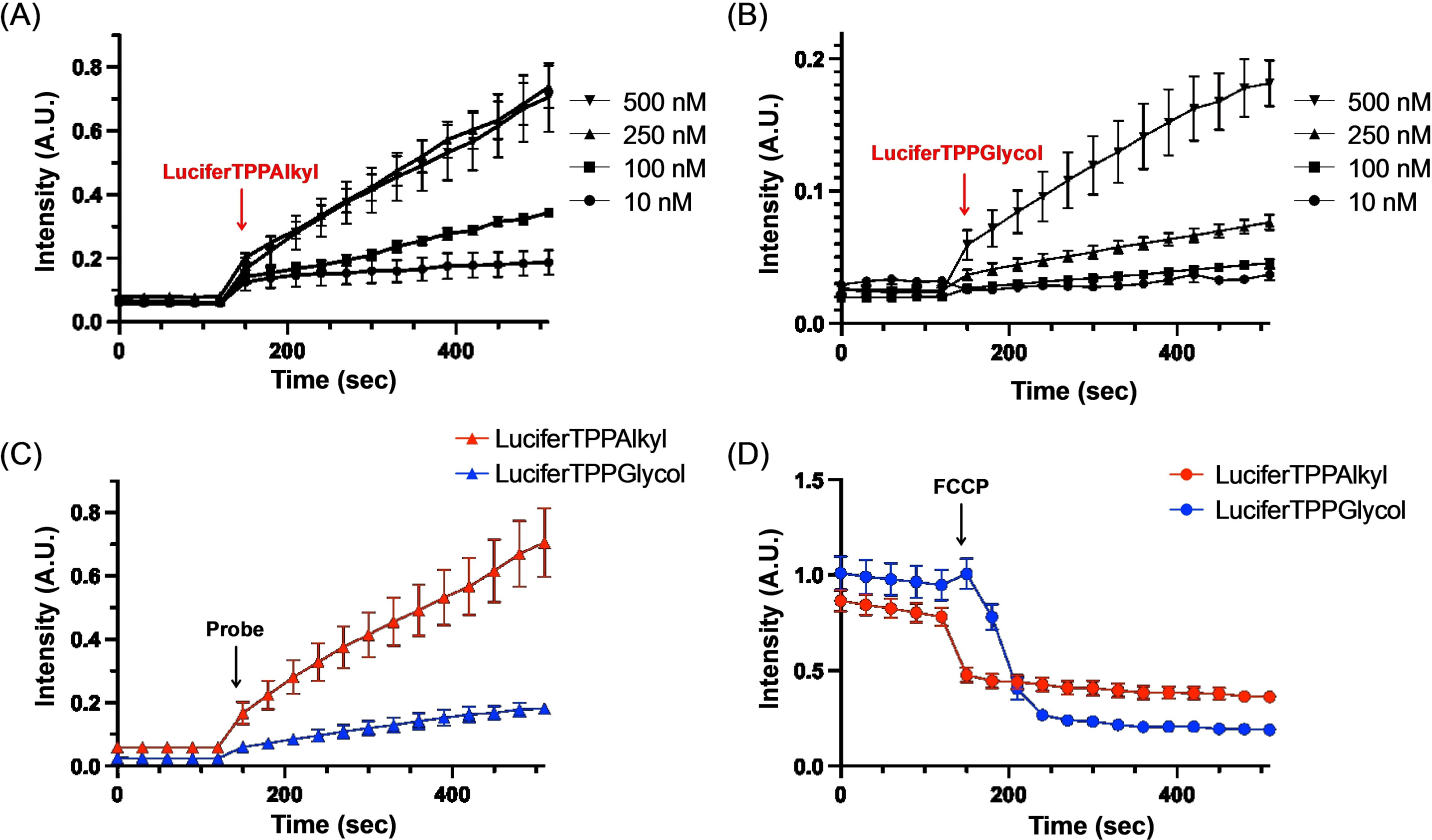
(A, B) The mitochondrial fluorescent intensity of Lucifer Yellow, normalized to that of HeLa‐TOMM20‐mCh, over time at various concentrations for LuciferTPPAlkyl (A) and for LuciferTPPGlycol (B). LuciferTPP was added at t=150. (C) Data for the mitochondrial accumulation of 500 nM of LuciferTPPAlkyl and LuciferTPPGlycol from Panels (A) & (B) are replotted on the same scale. (D) Loss of LuciferTPPAlkyl and LuciferTPPGlycol from mitochondria within cells by the addition of uncoupler FCCP (500 nM). Cells were incubated with the compounds at 10 nM of LuciferTPPAlkyl or 250 nM of LuciferTPPGlycol for 500 s, media was then removed, cells washed and reincubated with new medium before addition of FCCP (500 nM). Data in A–D are means±SEM of three independent experiments.

The real‐time uptake of both compounds evaluated by confocal live cell imaging over concentrations from 10–500 nM are shown in Movies 1 & 2. LuciferTPPAlkyl was readily taken up into mitochondria at all concentrations assessed (Movie 1). LuciferTPPGlycol was also readily taken up into mitochondria at concentrations of 100 nM and above (Movie 2). However, at 10 nM LuciferTPPGlycol did not localize to mitochondria, instead it accumulated in punctate structures, suggesting potential uptake by an endosomal pathway at low concentrations. LuciferTPPAlkyl showed higher intensity than LuciferTPPGlycol at the same concentration at all time points (Movies 1 & 2). To quantify this, we plotted the mitochondrial fluorescent intensity of Lucifer Yellow, normalized to that of HeLa‐TOMM20‐mCh, over time at various concentrations for LuciferTPPAlkyl (Figure 7A) and for LuciferTPPGlycol (Figure 7B). This analysis revealed that the rate of uptake of LuciferTPPGlycol was far slower than for LuciferTPPAlkyl, and this was clearer when the data were plotted on the same scale (Figure 7C, at 500 nM). To see if the compounds were retained within mitochondria in cells by the mitochondrial membrane potential, the cells were incubated with the compounds and then the incubation medium was replaced with medium without compounds and uncoupler FCCP was added and the mitochondrial content of the compounds assessed over time for LuciferTPPAlkyl (Movie 3) and for LuciferTPPGlycol (Movie 4). This analysis showed that there was rapid loss of both compounds from mitochondria upon uncoupling, however, at higher concentrations LuciferTPPAlkyl was partially retained within mitochondria, possibly due to its greater adsorption to membranes (Movie 3). Comparison of the relative uptake of the compounds over 500s indicated that the uptake of 10 nM LuciferTPPAlkyl was similar to that of 250 nM LuciferTPPGlycol, respectively (Figure S5). Therefore we compared the release of compounds upon addition of FCCP after incubation with 10 nM LuciferTPPAlkyl or 250 nM LuciferTPPGlycol for 500s (Figure 7D). This showed release of compounds from mitochondria upon uncoupling. Together these data indicate that both the LuciferTPPAlkyl and LuciferTPPGlycol are taken up into mitochondria within cells in response to the mitochondrial membrane potential.

## Conclusion

We have synthesized a range of TPP‐conjugated compounds, using either an alkyl or PEG linker and compared their distribution and reactivity in isolated mitochondria and in cells. We showed that replacement of alkyl with a PEG linker reduces hydrophobicity, but still enables accumulation of the conjugates within energized mitochondria. However, the rate of accumulation of the PEG conjugates by mitochondria within cells is slower than that of the corresponding alkyl conjugates, due to slower membrane permeation. Within mitochondria, incorporation of a PEG linker greatly decreases adsorption of conjugates to the matrix facing surface of the mitochondrial inner membrane. These findings should allow the selective delivery of bioactive molecules to matrix and membrane locations within mitochondria in vivo.

## Experimental Section

**Synthesis of TPP‐conjugated compounds**: The syntheses of TPP‐conjugated compounds and full experimental procedures provided in Supporting Information 1.

**Isolation of mitochondria and cytosolic fraction**: Rat liver and heart mitochondria were prepared by homogenization of tissues obtained from 10 to 12 weeks old female Wistar rats (Charles River, UK) that were killed by stunning and cervical dislocation. The liver was homogenized in STE buffer (250 mM sucrose, 5 mM Tris‐HCl and 1 mM EGTA, pH 7.4) and the heart in STEB buffer (STE buffer+0.1 % (w/v) bovine serum albumin (BSA)). Homogenate was centrifuged at 1000×g for 3 min (for livers) or 700×g for 5 min (for hearts). The resulting supernatant (for hearts, the supernatant was filtered through pre‐wetted muslin) was centrifuged to pellet mitochondria (10000×g for 10 min at 4 °C). The supernatant from liver was collected, centrifuged (17000×g for 10 min at 4 °C), and the supernatant was collected as a cytosolic fraction after removing fluffy stuff. The mitochondrial pellet was resuspended in buffer and recentrifuged under the same conditions to pellet mitochondria. Protein concentration was determined by the bicinchoninic acid (BCA) assay using BSA as a standard.

**RP‐HPLC analysis of mitochondrial uptake**: Liver mitochondria (2 mg protein/mL) were incubated in 1 mL KCl buffer (120 mM KCl, 10 mM HEPES, 1 mM EGTA, pH 7.2) at 37 °C for 5 min supplemented with rotenone (4 mg/mL), succinate (10 mM), nigericin (0.1 μM), and appropriate TPP^+^‐conjugated compounds (10 μM). When acetyl derivatives were used, heart mitochondria (0.5 mg protein/mL) were incubated with TPP^+^‐conjugated compounds (10 μM) and TPMP (3 μM) for the indicated times to follow the reaction. Where indicated FCCP (0.01–0.5 μM) was added. After the incubation, samples were cooled on ice before pelleting mitochondria by centrifugation (17000 g, 5 min, 4 °C). Then supernatant was removed, the mitochondrial pellet was dried with tissue paper, and extracted with 250 μL ACN/0.1 %TFA. After centrifugation, the supernatant was diluted with 750 μL H_2_O/0.1 %TFA and filtered through a 0.20 μm 96‐well polyethylene filter plate (ThermoFisher Scientific) before analyzing by RP‐HPLC using a C18 column (Jupiter 300 A, Phenomenex) attached to a Widepore C18 guard column (Phenomenex), all driven by a Gilson 321 pump. A flow rate of 1 mL/min was used with a gradient of 0.1 % (v/v) TFA in water (Buffer A) and 0.1 % (v/v) TFA in ACN (Buffer B). UV absorbance and fluorescence detection were measured by a UV‐Visible detector (Gilson 151) at 220 nm and fluorescence detector (Shimadzu Rf‐10 A), respectively. A gradient (%B) of buffer A and B was: 0–2 min, 5 %; 2–17 min, 5–100 %; 17–20 min, 100 %; 20–22 min, 100–5 % for hydroxyl derivatives: 0–2 min, 5 %; 2–4 min, 5–55 %; 4–17 min, 55–70 %; 17–18 min, 70–100 %; 18–21 min, 100 %; 21–23 min, 100–5 % for benzyl derivatives: 0–2 min, 5 %; 2–4 min, 5–50 %; 4–21 min, 50–100 %; 21–24 min, 100 %; 24–26 min, 100–5 % for acetyl derivatives.

**Ion‐selective electrode measurements**: Ion‐selective electrodes sensitive to the TPP cation moiety were constructed as previously described.[^22^, ^39^] The electrode and a Ag/AgCl reference electrode were put in a stirred incubation chamber containing 1 mL of KCl buffer and rotenone (4 μg/mL) at 37 °C. When the rates of the uptake were compared, the time point where succinate was added was set as t=0. The percentage were calculated by setting t=0, 60 as 0, 100 % of uptake, respectively.

**Hydrolysis of acetyl derivatives in cytosolic fraction**: Acetyl derivatives (200 μM) and the internal standard (TPMP, 200 μM) were incubated with cytosolic fraction (1 mg protein/mL) in 0.5 mL KCl buffer at 37 °C. Then, 20 μL samples were taken at the indicated time points and extracted in 250 μL of HPLC buffer B. The extraction was diluted with 750 μL of HPLC buffer A and filtered through a 0.20 μm 96‐well polyethylene filter plate (ThermoFisher Scientific) before analyzing RP‐HPLC by the same condition as mitochondrial uptake.

**Plasmids and constructs**: mCherry‐TOMM20‐N‐10 was a gift from Michael Davidson (Addgene plasmid # 55146). pCDH‐EF1‐copGFP‐T2A‐Puro was a gift from Kazuhiro Oka (Addgene plasmid # 72263). mCherry‐TOMM20‐N‐10 was amplified by mCherry‐TOMM20‐N‐10 by PCR using the forward and reverse primers mChTOMM20F and mChTOMM20R. pCDH‐EF1‐copGFP‐T2A‐Puro was linearized by PCR using the forward and reverse primers LinF and LinR thus removing the copGFP fragment. The pCDH‐mCherry‐TOMM20‐T2 A‐Puro lentiviral vector was created by inserting the mCherry‐TOMM20‐N‐10 fragment into the linearized pCDH‐EF1‐T2A‐Puro plasmid with an HD Infusion Cloning kit.

mChTOM20F: GCAATTGGATCCACCGCCACCATGGTGGGT

mChTOM20R: CTGAGCGCGGGATCTCTTGTACAGCTCGTCCATGCCG

LinF: AGATCCCGCGCTCAGT

LinR: GGTGGATCCAATTGCTCACG

**Lentiviral production and transduction**: Lentiviral particles for pCDH‐mCherry‐TOMM20‐T2A‐Puro were generated in HEK‐293T cells via co‐transfection of the target vector together with packaging psPAX2 (Addgene, #12260) and envelope pMD2.G (Addgene, #12259) vectors. The virus‐containing supernatants were cleared by filtration through a 0.45 μm PES filter. Target cells (WT HeLa) were transduced by addition of viral supernatant and 10 μg/ml polybrene. Twenty‐four hours after transduction, cells were selected for puromycin resistance.

**Cell culture**: HeLa cells stably expressing the mitochondrial outer membrane protein TOM20 tagged with the fluorescent protein mCherry (HeLa‐mCherry‐TOMM20) were cultured in Dulbecco's Modified Eagles Medium (DMEM) supplemented with 10 % fetal bovine serum (FBS), 2 mM L‐glutamine, non‐essential amino acids and 100 units/mL penicillin/streptomycin (all from GIBCO) at 37 °C with 5 % CO_2_. Cells were seeded at a confluency of 15,000 cells in 8‐well Ibidi imaging chambers (Ibidi), grown overnight and processed for imaging.

**Cell imaging**: Cells seeded in 8‐well Ibidi imaging chambers were imaged using a 100X objective lenses (NA1.4) on a Nikon Eclipse TiE inverted microscope with appropriate lasers using an Andor Dragonfly 500 confocal spinning disk system, equipped with an iXon Ultra 888 EMCCD camera (for live cell) (Andor), coupled with Fusion software (Andor). Live cell imaging was performed at 37 °C with 5 % CO_2_ using an Okolab stage top incubator system. Images were acquired every 30 seconds with the 488 nm and 561 nm laser lines of the Andor Dragonfly 500 confocal spinning disk system. The intensity of both the dye (488 nm) and TOMM20 tagged with the fluorescent protein mCherry (561 nm) were measured using the plot profile function of FIJI. The intensity of the dyes was normalized to the intensity of TOMM20 tagged with the fluorescent protein mCherry and plotted as arbitrary units against time. To measure dye uptake, dyes were added at t=150s. To measure dye efflux, cells were loaded with the indicated concentrations of the dyes for 10 min. Following 3× washes with warm media, 500 nM FCCP was added at t=150s and the cells were then imaged every 30 seconds for 500 seconds or till the dye was no longer found within mitochondria. All experiments were performed in triplicate with 6 regions of interests quantified from each experiment.

## Conflict of interest

The authors declare no conflict of interest.

1

## Supporting information

As a service to our authors and readers, this journal provides supporting information supplied by the authors. Such materials are peer reviewed and may be re‐organized for online delivery, but are not copy‐edited or typeset. Technical support issues arising from supporting information (other than missing files) should be addressed to the authors.

Supporting Information

Supporting Information

Supporting Information

Supporting Information

Supporting Information

## Data Availability

The processed NMR spectra for synthesised compounds appear in Supporting Information 2. The raw NMR data files can be found at https://doi.org/10.5525/gla.researchdata.1385.
