# Supplementary material for: Incorporating a Polyethyleneglycol Linker to Enhance the Hydrophilicity of Mitochondria‐Targeted Triphenylphosphonium Constructs
Source: Chembiochem. 2023 May 4;24(11):e202200774. doi: 10.1002/cbic.202200774 (PMC10946768; doi:10.1002/cbic.202200774)
Supplement: Supplementary file 1 — Supporting Information [file CBIC-24-0-s002.pdf]

ChemBioChem

Supporting Information

Incorporating a Polyethyleneglycol Linker to Enhance the Hydrophilicity of Mitochondria-Targeted Triphenylphosphonium Constructs

Shinpei Uno, Alexander H. Harkiss, Roy Chowdhury, Stuart T. Caldwell, Tracy A. Prime, Andrew M. James, Brendan Gallagher, Julien Prudent, Richard C. Hartley,* and Michael P. Murphy*

Contents

1	Supplementary Figures	Pages S2–S6
2	Outline of Syntheses	Pages S7–S8
3	Experimental Procedures for Synthesis	
	3.1 Tested Compounds	Pages S9–S14
	3.2 Synthetic Intermediates (Ordered by Compound Number)	Pages S14-S18
4	References	Page S19
5	NMR spectra of synthesised compounds	69 pages after S19
	(ordered in the same way as the compounds in the experimental)	

1. Supplementary Figures

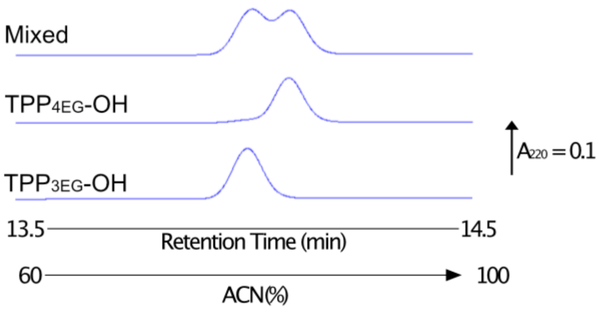

Figure S1. Comparison of the hydrophobicity of TPP_{4EG}-OH and TPP_{3EG}-OH. A mixture of TPP_{4EG}-OH and TPP_{3EG}-OH (1 nmol of each) was analyzed by RP-HPLC (upper trace) and compared with samples (1 nmol) of TPP_{4EG}-OH (middle trace) and TPP_{3EG}-OH (lower trace).

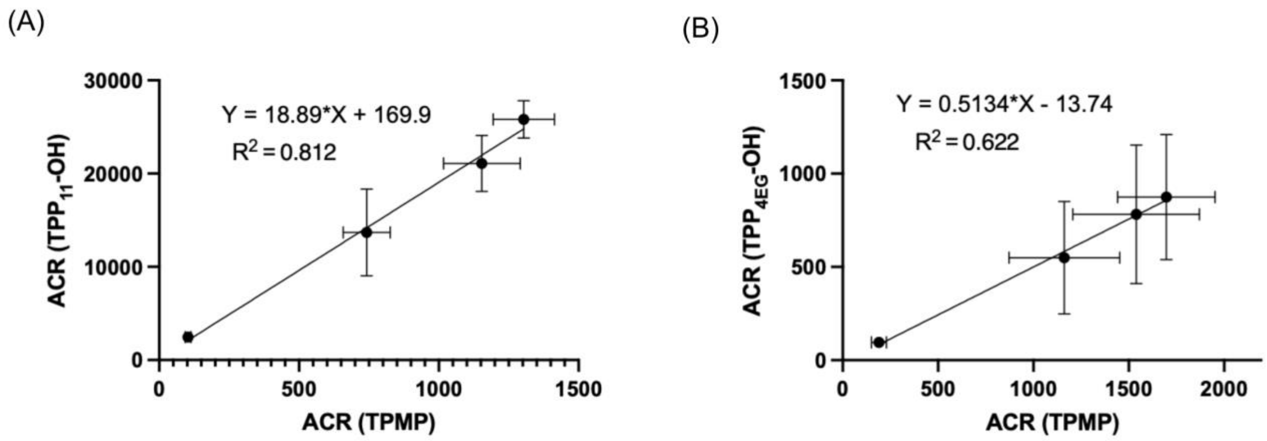

Figure S2. Accumulation ratios (ACRs) of TPP_{11}-OH and TPP_{4EG}-OH relative to that if TPMP. Mitochondria were incubated with TPP_{11}-OH or TPP_{4EG}-OH (10 µM) along with TPMP (3 µM) and different concentrations of FCCP (0 – 0.5 µM). The mitochondria and supernatants were then extracted and analyzed by RP-HPLC to indicate the relative concentrations of the compounds in the two compartments, and from the ACR values were calculated.

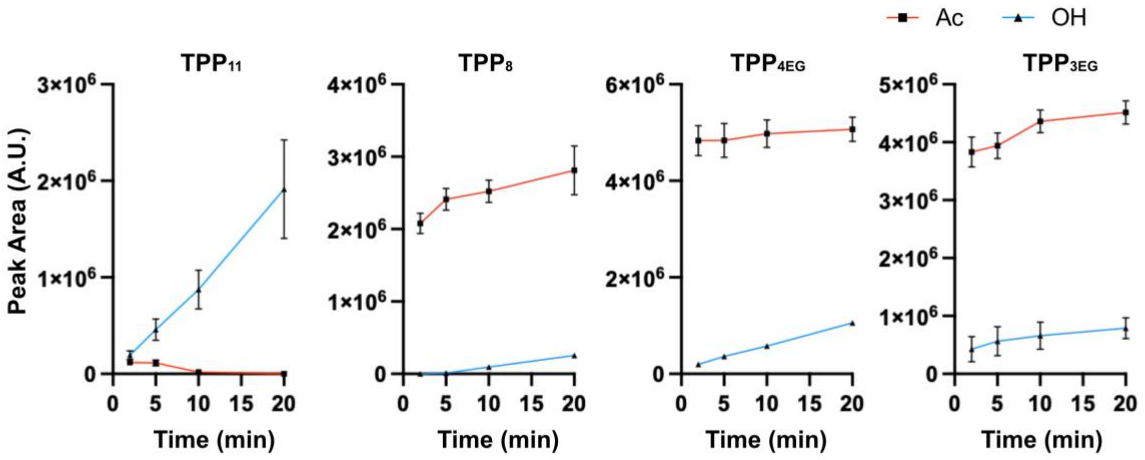

Figure S3. Enzymatic hydrolysis of TPP-conjugated acetyl esters. TPP acetyl esters (10 µM) and internal standard TPMP (3 µM) were incubated with rat heart mitochondria (0.5 mg protein/mL) before pelleting mitochondria, extracting the supernatants, and analyzing by RP-HPLC. The data show the peak areas of the ester and hydroxyl compounds in the supernatants. All data are means ± SEM of three independent experiments.

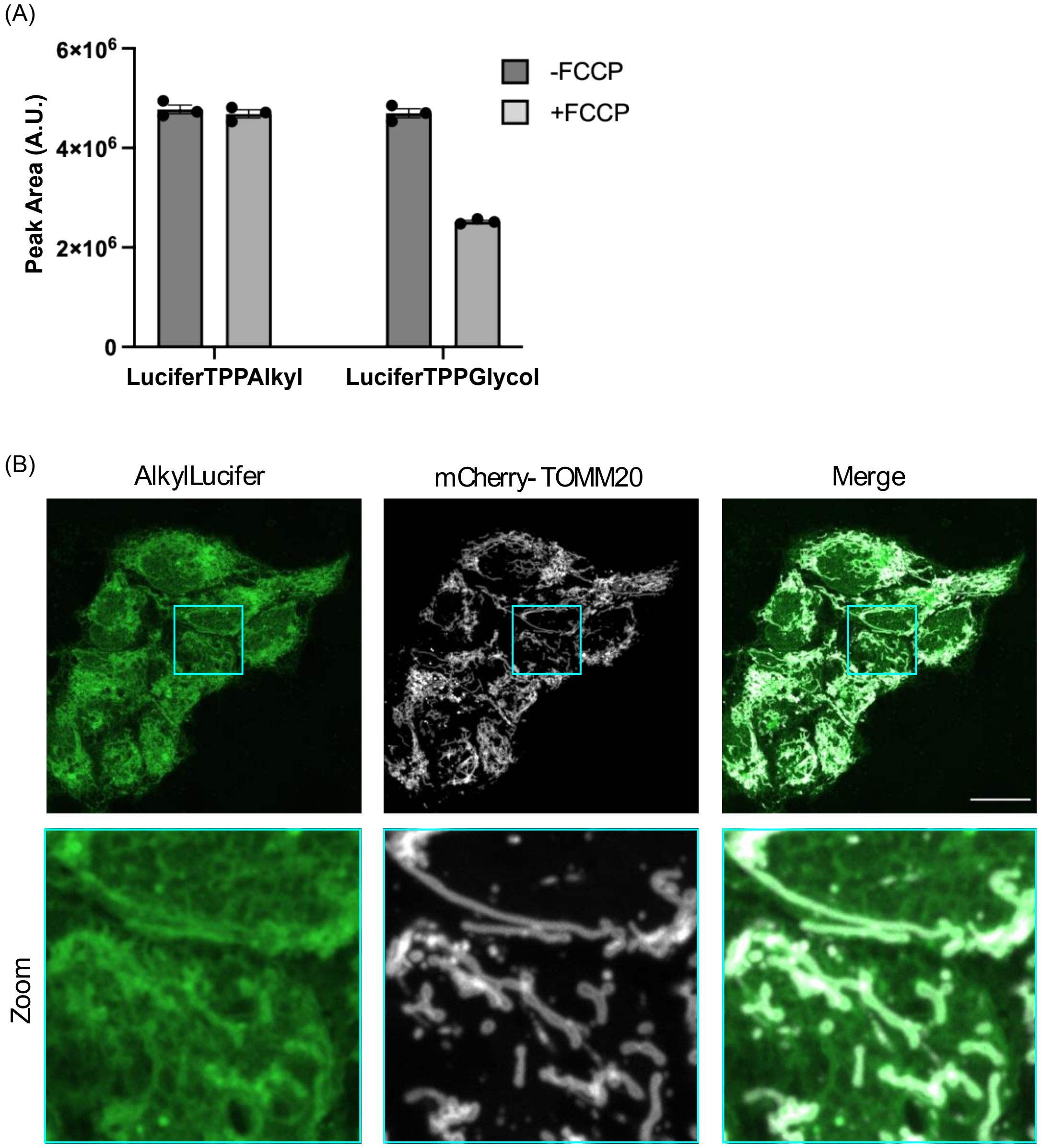

Figure S4. (A) Uptake of LuciferTPPAlkyl and LuciferTPPGlycol by isolated energized mitochondria. Compounds (10 μM) were incubated with energized rat liver mitochondria (2 mg protein/mL) ± FCCP (0.5 μM) for 5 min before pelleting the mitochondria by centrifugation, extracting the pellet, and quantifying the amount of compound present by RP-HPLC. All data are means ± SEM of three independent experiments. (B) Distribution of the control dye, LuciferAlkyl, in HeLa cells stably expressing the mitochondrial outer membrane protein TOMM20 tagged with the fluorescent protein mCherry (HeLa-mCherry-TOMM20) at 500 s after addition of 250 nM of compound. Scale bar =15 μm. Scale bar for zoomed insets = 5 μm.

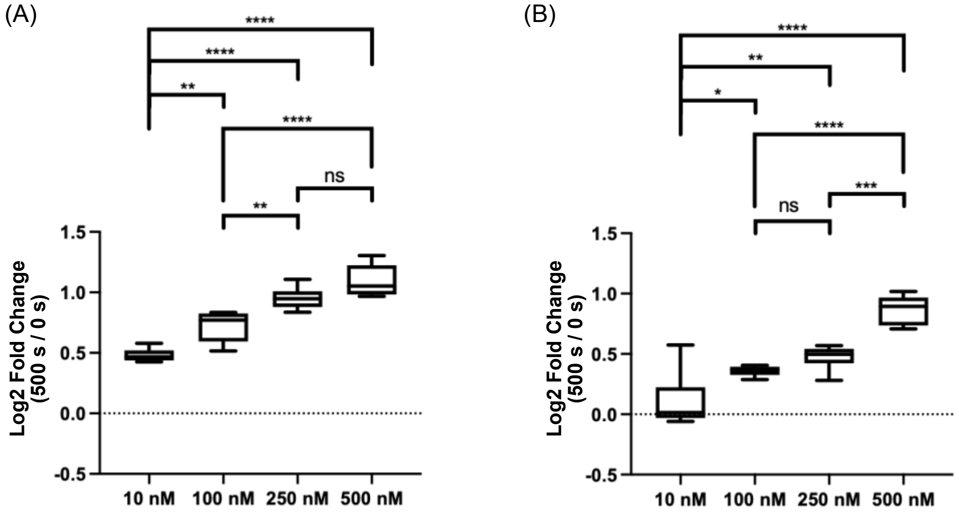

Figure S5. Comparison of the fluorescence intensity of LuciferTPPAlkyl (A) and LuciferTPPGlycol (B). The fluorescence of Lucifer Yellow was observed here 500 s after the addition of either compound at various concentration. Their intensity was expressed with Log2 Fold Change by comparing arbitrary fluorescence intensity values of the dyes at 0s versus intensity at 500s. Statistical significance was assessed by one-way ANOVA with Tukey's test for multiple comparisons; *p < 0.05, **p < 0.01, ***p < 0.001, ****p < 0.0001.

2. Outline of Syntheses

The TPP-alkyl series of alcohols **TPP$_8$-OH** and **TPP$_{11}$-OH**, acetate esters **TPP$_8$-OAc** and **TPP$_{11}$-OAc**, and benzyl ethers **TPP$_8$-OBn** and **TPP$_{11}$-OBn** were prepared from the corresponding bromoalcohols **1** and **2** (Scheme 1). Esterification of alcohols **1** and **2** with acetyl chloride gave acetates **3** and **4** in good yield. Deprotonation of alcohols **1** and **2** with sodium hydride and reaction with benzyl bromide gave a modest yield of the benzyl ethers **5** and **6**. Bromooctyl acetate **5** could not be fully purified and was sufficiently pure for the next reaction. The TPP-alkyl bromides **1**-**6** were then heated with triphenylphosphine in acetonitrile to give the phosphonium salts **TPP$_8$-OH**, **TPP$_{11}$-OH**, **TPP$_8$-OAc**, **TPP$_{11}$-OAc**, **TPP$_8$-OBn** and **TPP$_{11}$-OBn**, generally in good yield.

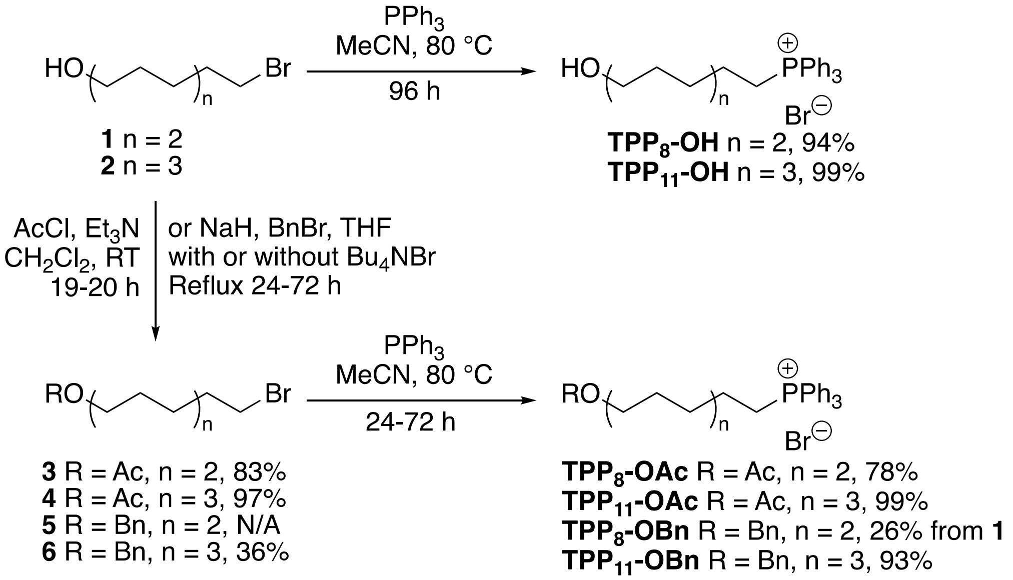

Scheme 1 Synthesis of TPP compounds with alkyl chains

The TPP-ethylene glycol series of alcohols **TPP$_{3EG}$-OH** and **TPP$_{4EG}$-OH**, acetate esters **TPP$_{3EG}$-OAc** and **TPP$_{4EG}$-OAc**, and benzyl ethers **TPP$_{3EG}$-OBn** and **TPP$_{4EG}$-OBn** were prepared similarly (Schemes 2 and 3). Triethylene glycol **7** and tetraethylene glycol **8** were converted to the corresponding monobromides **9** and **10** with concentrated hydrobromic acid in modest yield. Acetylation then gave esters **11** and **12**. The bromides bearing a benzyl ether **13** and **14** were prepared by the methods of David *et al.*[1] and Felber *et al.*[2] on a 4.1 mmol and 2.8 mmol scale respectively (Scheme 3). Finally reacting the bromides **9**-**12** with triphenylphosphine gave the corresponding TPP salts **TPP$_{3EG}$-OH**, **TPP$_{4EG}$-OH**, **TPP$_{3EG}$-OAc**, **TPP$_{4EG}$-OAc**, **TPP$_{3EG}$-OBn** and **TPP$_{4EG}$-OBn** in good to excellent yields.

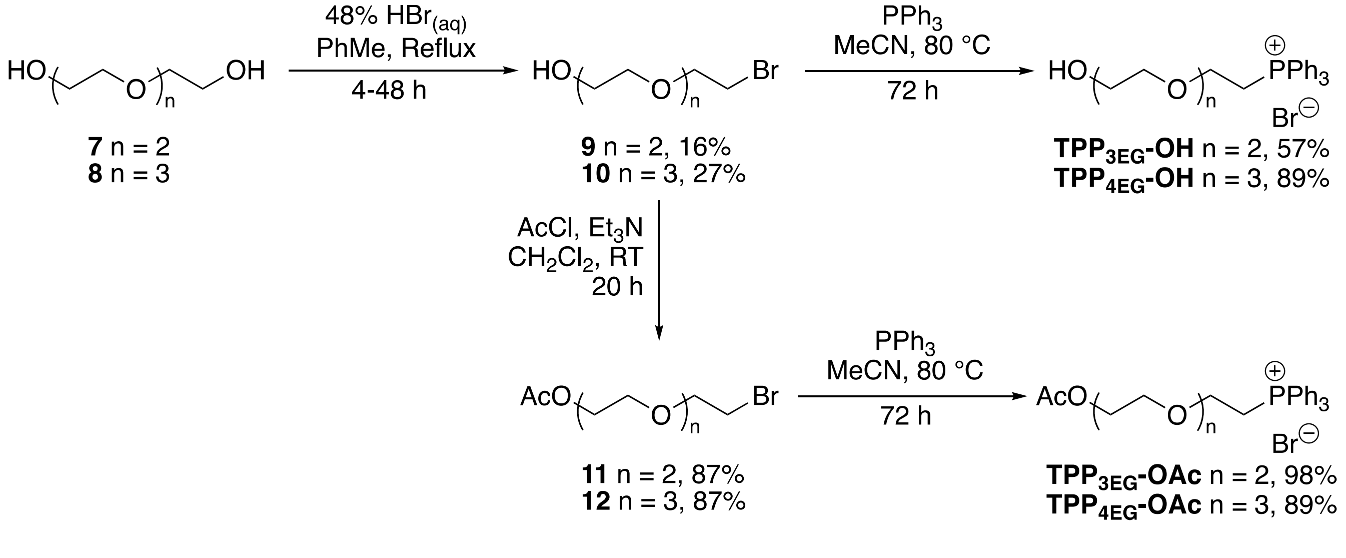

Scheme 2 Synthesis of TPP compounds with chains comprised of three or four ethylene glycol units

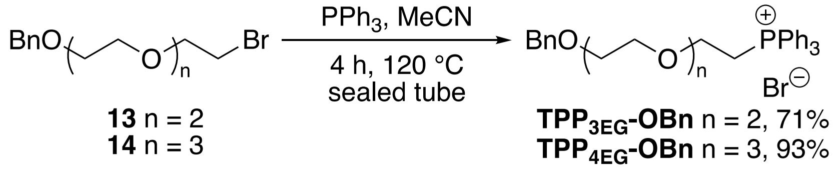

Scheme 3 Synthesis of TPP benzyl compounds with chains comprised of three or four ethylene glycol units

Amino alcohols **15** and **16** were then used in the synthesis of **LuciferTPPAlkyl** and **LuciferTPPGlycol** (Scheme 4). They were themselves prepared on an approximately 30 mmol scale by the methods of Puchkov *et al.*[3] and Salvagini *et al.*[4], respectively, with DMSO used instead of DMF in the reaction to produce 8-azidooctan-1-ol. The amino alcohols **15** and **16** were converted into naphthalimides **17** and **18** by reaction with electron-poor 4-nitro-1,8-naphthalic anhydride. The nitro group was reduced to give amines **19** and **20** in good yield. Appel bromination then produced the alkyl bromides **21** and **22** in modest yield. Finally, the bromine was displaced by triphenylphosphine to give the mitochondria-targeted dyes, **LuciferTPPAlkyl** and **LuciferTPPGlycol**.

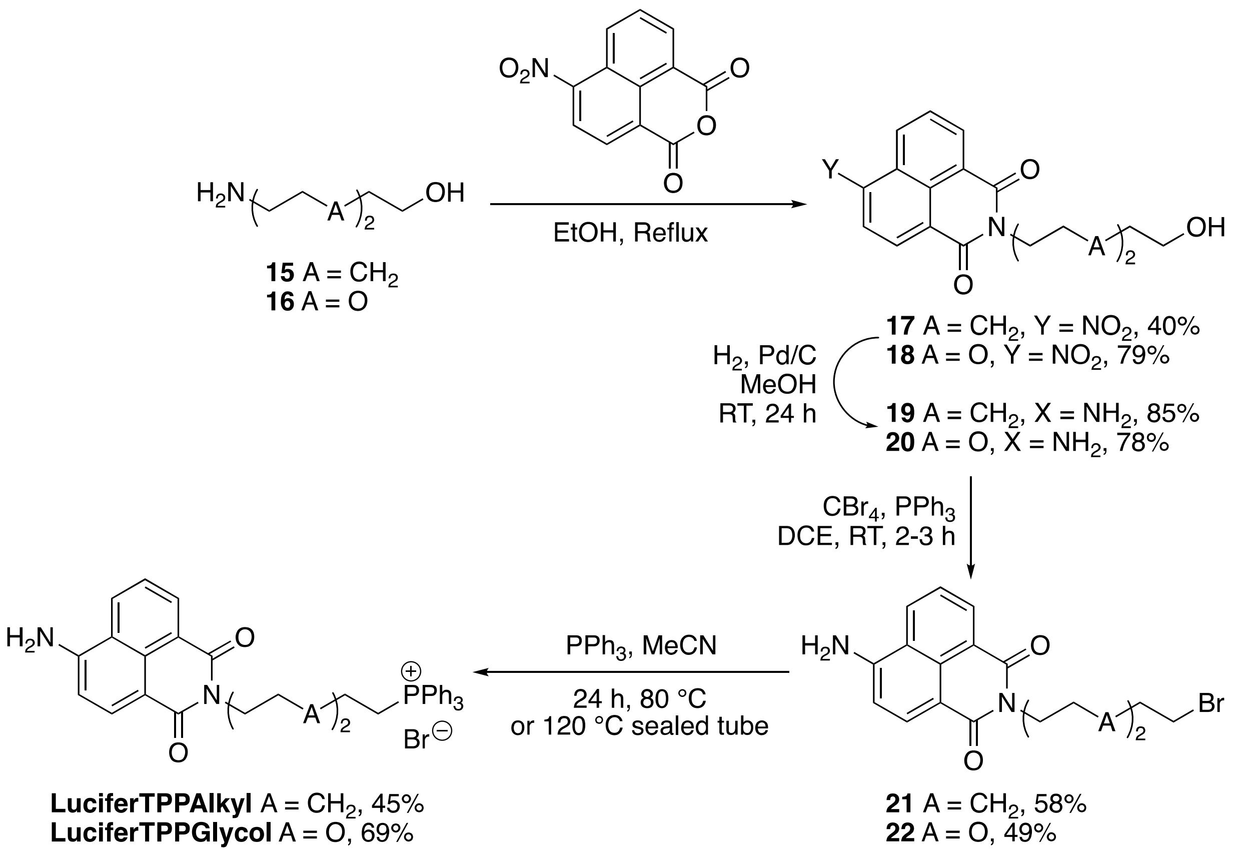

Scheme 4 Synthesis of TPP-Lucifer dye conjugates

The untargeted Lucifer dye was prepared by first converting hexylamine into the electron poor napththalimide **17** in modest yield, and then reducing the nitro group to give the amine, **LuciferAlkyl**, in excellent yield (Scheme 5).

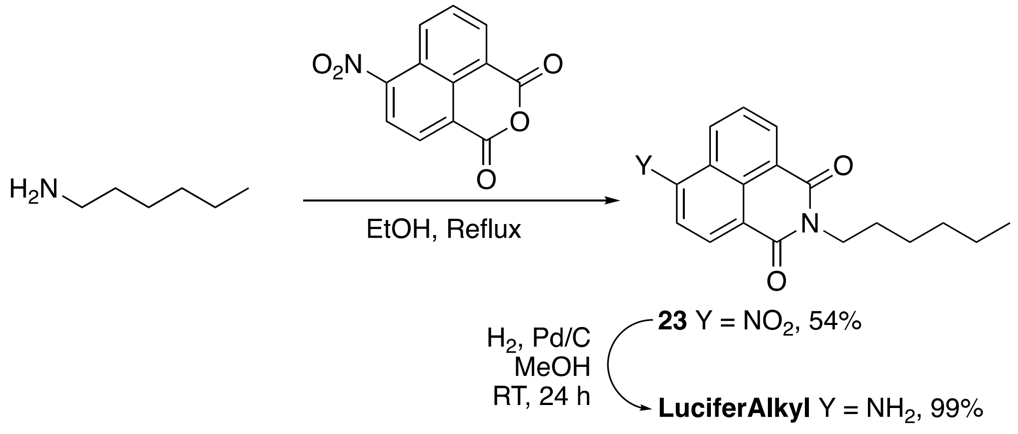

Scheme 5 Synthesis of untargeted Lucifer dye

3. Experimental Procedures for Synthesis

3.1 Tested Compounds

(8-Hydroxyoct-1-yl)triphenylphosphonium bromide TPP$_8$-OH

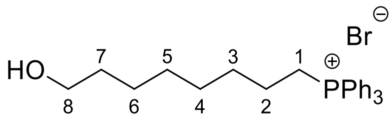

8-Bromo-1-octanol **1** (205 mg, 0.980 mmol) was dissolved in argon degassed anhydrous acetonitrile (10 mL), followed by addition of triphenylphosphine (514 mg, 1.96 mmol). The reaction vessel was then purged with argon and heated under reflux for 72 h. The reaction was then cooled and concentrated *in vacuo*. The crude material was purified by manual flash column chromatography on silica, eluting 5% methanol in dichloromethane to produce **TPP$_8$-OH** (434 mg, 94%) as a waxy colourless oil. δ_H (400 MHz, $CDCl_3$): δ 7.88–7.65 (15H, m, ArH × 15), 3.82–3.71 (2H, m, 1-H_2), 3.58 (2H, t, *J* = 6.5 Hz, 8-H_2), 2.14 (br s, 1H, OH), 1.70–1.44 (6H, m, 2-H_2, 3-H_2 and 7-H_2), 1.37–1.17 (6H, m, 4–6-H_2). δ_C (101 MHz, $CDCl_3$): 135.11 (d, *J* = 3.0 Hz, CH × 3), 133.81 (d, *J* = 10.0 Hz, CH × 6), 130.61 (d, *J* = 12.5 Hz, CH × 6), 118.52 (d, *J* = 85.8 Hz, C × 3), 62.70 (CH_2), 32.56 (CH_2), 30.14 (d, *J* = 15.9 Hz, CH_2), 28.80 (CH_2), 28.73 (CH_2), 25.46 (CH_2), 22.81 (d, *J* = 49.8 Hz, CH_2), 22.62 (d, *J* = 4.5 Hz, CH_2). δ_P (162 MHz, $CDCl_3$): 24.42 (s). *m/z* (ESI) 391.2184 (M^+. $C_{26}H_{32}OP$ requires 391.2185). ^1H and ^{13}C NMR data agree with the literature.[5]

(8-Acetoxyoct-1-yl)triphenylphosphonium bromide TPP$_8$-OAc

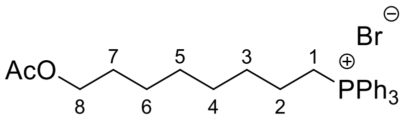

Ester **3** (174 mg, 0.693 mmol) was dissolved in argon degassed anhydrous acetonitrile (10 mL), followed by addition of triphenylphosphine (363 mg, 1.39 mmol). The reaction vessel was then purged with argon and heated under reflux for 72 h. The reaction was then cooled and concentrated *in vacuo*. The crude material was purified by manual flash column chromatography on silica, eluting 5% methanol in dichloromethane to produce **TPP$_8$-OAc** (279 mg, 78%) as a viscous colourless oil. δ_H (400 MHz, $CDCl_3$): 7.92–7.65 (15H, m, ArH × 15), 4.00 (2H, t, *J* = 6.8 Hz, 8-H_2), 3.93–3.83 (2H, m, 1-H_2), 2.02 (3H, s, CH_3), 1.70–1.50 (6H, m, overlapping water peak, 2-H_2, 3-H_2 and 7-H_2), 1.33–1.19 (6H, m, 4–6-H_2). δ_C (101 MHz, $CDCl_3$): 171.40 (C), 135.06 (d, *J* = 3.0 Hz, CH × 3), 133.90 (d, *J* = 9.9 Hz, CH × 6), 130.58 (d, *J* = 12.5 Hz, CH × 6), 118.70 (d, *J* = 85.8 Hz, C × 3), 64.65 (CH_2), 30.42 (d, *J* = 15.6 Hz, CH_2), 29.26 (CH_2), 28.92 (CH_2), 28.63 (CH_2), 25.90 (CH_2), 22.94 (d, *J* = 49.7 Hz, CH_2), 22.83 (d, *J* = 4.6 Hz, CH_2), 21.17 (CH_3). δ_P (162 MHz, $CDCl_3$): 24.64 (s). *m/z* (ESI) 433.2292 (M^+. $C_{28}H_{34}O_2P$ requires 433.2291).

(8-Benzyloxyoct-1-yl)triphenylphosphonium bromide TPP$_8$-OBn

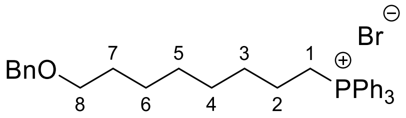

8-Bromo-1-octanol **1** (217 mg, 1.04 mmol) was dissolved in anhydrous tetrahydrofuran (5 mL) and cooled to 0 °C. Sodium hydride (44.0 mg, 1.09 mmol, 60% dispersion in mineral oil) was added and the solution was allowed to stir for 15 minutes. Benzyl bromide (148 µL, 1.25 mmol) and tetrabutylammonium iodide (38.0 mg, 0.1037 mmol) were added and the mixture was heated under reflux for 24 h. The reaction was then cooled to 0 °C and quenched by addition of a saturated aqueous solution of ammonium chloride (5 mL). The solution was diluted with water (10 mL), extracted with ethyl acetate (10 mL × 3), dried over $MgSO_4$, filtered, and concentrated *in vacuo*. The crude material was purified by manual flash column chromatography on silica, eluting 20% dichloromethane in petroleum ether (40–60) to produce benzyl ether **5** (130 mg) as a colourless oil, which was impure and was used without further purification. The benzyl ether **5** (86 mg, 0.29 mmol, impure) was dissolved in argon degassed anhydrous acetonitrile (10 mL), followed by addition of triphenylphosphine (150 mg, 0.575 mmol). The reaction vessel was then purged with argon and heated under reflux for 72 h. The reaction was then cooled and concentrated *in vacuo*. The crude material was

purified by manual flash column chromatography on silica, eluting 5% methanol in dichloromethane to produce **TPP$_8$-OBn** (101 mg, 26% over 2 steps) as a viscous colourless oil. δ_H (400 MHz, $CDCl_3$): 7.82–7.62 (15H, m, ArH of PPh_3), 7.30–7.17 (5H, m, overlapping $CDCl_3$, ArH of Bn), 4.42 (s, 2H, C*H*$_2$Ph), 3.71–3.59 (2H, m, 1-H_2), 3.37 (2H, t, *J* = 6.6 Hz, 8-H_2), 1.64–1.44 (6H, m, 2-H_2, 3-H_2 and 7-H_2), 1.31–1.12 (6H, m, 4–6-H_2). δ_C (101 MHz, $CDCl_3$): 138.67 (C), 135.08 (d, *J* = 3.0 Hz, CH × 3), 133.62 (d, *J* = 10.0 Hz, CH × 6), 130.54 (d, *J* = 12.5 Hz, CH × 6), 128.30 (CH × 2), 127.59 (CH × 2), 127.43 (CH), 118.28 (d, *J* = 85.9 Hz, C × 3), 72.82 (CH_2), 70.41 (CH_2), 30.32 (d, *J* = 15.5 Hz, CH_2), 29.62 (CH_2), 29.06 (CH_2), 28.96 (CH_2), 26.00 (CH_2), 22.84 (d, *J* = 44.6 Hz, CH_2), 22.57 (CH_2). δ_P (162 MHz, $CDCl_3$): 24.19 (s). *m/z* (ESI) 481.2651 (M^+. $C_{33}H_{38}OP$ requires 481.2655).

(11-Hydroxyundec-1-yl)triphenylphosphonium bromide TPP$_{11}$-OH

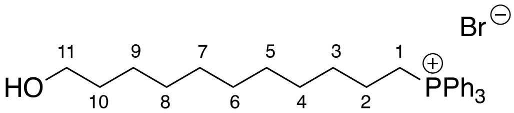

11-Bromo-1-undecanol **2** (1.51 g, 6.00 mmol) was dissolved in argon degassed anhydrous acetonitrile (50 mL), followed by addition of triphenylphosphine (3.15 g, 12.0 mmol). The reaction vessel was then purged with argon and heated under reflux for 96 h. The reaction was then cooled and concentrated *in vacuo*. The crude material was purified via trituration, by dissolving the crude material in a minimal amount of dichloromethane and dropping into excess diethyl ether, followed by filtration to produce **TPP$_{11}$-OH** (3.07 g, 99%) as a waxy white solid. δ_H (400 MHz, $CDCl_3$): 7.83–7.73 (9H, m, ArH × 9), 7.71–7.64 (6H, m, ArH × 6), 3.66 (2H, dt, *J* = 12.3, 7.7 Hz, H_2-1), 3.56 (2H, t, *J* = 6.7 Hz, H_2-11), 2.10 (1H, br s, OH), 1.63–1.43 (6H, m), 1.30–1.10 (12H, m). δ_C (101 MHz, $CDCl_3$): 135.11 (d, *J* = 3.0 Hz, CH × 3), 133.66 (d, *J* = 10.0 Hz, CH × 6), 130.57 (d, *J* = 12.5 Hz, CH × 6), 118.31 (d, *J* = 85.8 Hz, C × 3), 62.70 (CH_2), 32.73 (CH_2), 30.38 (d, *J* = 15.5 Hz, CH_2), 29.35 (CH_2), 29.27 (CH_2), 29.24 (CH_2), 29.05 (CH_2 × 2), 25.70 (CH_2), 22.79 (d, *J* = 50.0 Hz, CH_2), 22.61 (d, *J* = 4.5 Hz, CH_2). δ_P (162 MHz, $CDCl_3$): 24.23. *m/z* (ESI) 433.2656 (M^+. $C_{29}H_{38}OP$ requires 433.2655). ^1H NMR data agree with the literature.[6]

(11-Acetoxyundec-1-yl)triphenylphosphonium bromide TPP$_{11}$-OAc

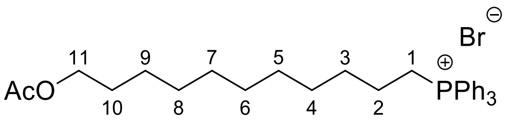

11-Bromoundec-1-yl acetate **4** (589 mg, 2.01 mmol) was dissolved in argon degassed anhydrous acetonitrile (10 mL), followed by addition of triphenylphosphine (1.05 g, 4.02 mmol). The reaction vessel was then purged with argon and heated under reflux for 24 h. The reaction was then cooled and concentrated *in vacuo*. The crude material was purified by manual flash column chromatography on silica, eluting 5% methanol in dichloromethane to produce **TPP$_{11}$-OAc** (1.21 g, 99%) as a viscous colourless oil. δ_H (400 MHz, $CDCl_3$): 7.90–7.65 (15H, m, ArH × 15), 4.02 (2H, t, J = 6.8 Hz, 11-H_2), 3.87–3.76 (2H, m, 1-H_2), 2.03 (3H, s, CH_3), 1.69–1.52 (6H, m, 2-H_2, 3-H_2 and 10-H_2), 1.34–1.13 (12H, m, 4–9-H_2). δ_C (101 MHz, $CDCl_3$): 171.40 (C), 135.07 (d, J = 3.0 Hz, CH × 3), 133.84 (d, J = 10.0 Hz, CH × 6), 130.58 (d, J = 12.5 Hz, CH × 6), 118.61 (d, J = 85.8 Hz, C × 3), 64.75 (CH_2), 30.54 (d, J = 15.5 Hz, CH_2), 29.51 (CH_2 × 2), 29.37 (CH_2), 29.28 (CH_2 × 2), 28.68 (CH_2), 25.97 (CH_2), 22.91 (d, J = 49.1 Hz, CH_2), 22.81 (d, J = 4.6 Hz, CH_2), 21.16 (CH_3). δ_P (162 MHz, $CDCl_3$): 24.48 (s). *m/z* (ESI) 475.2759 (M^+. $C_{31}H_{40}O_2P$ requires 475.2760). ^1H and ^{13}C NMR data agree with the literature.[7]

(11-Benzyloxyundec-1-yl)triphenylphosphonium bromide TPP$_{11}$-OBn

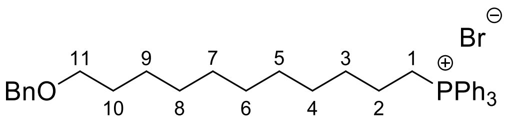

Benzyl ether **6** (204 mg, 0.598 mmol) was dissolved in argon degassed anhydrous acetonitrile (10 mL), followed by addition of triphenylphosphine (313 mg, 1.20 mmol). The reaction vessel was then purged with argon and heated under reflux for 72 h. The reaction was then cooled and concentrated *in vacuo*. The crude material was purified by manual flash column chromatography on silica, eluting 5% methanol in dichloromethane to produce **TPP$_{11}$-OBn** (333 mg, 93%) as a viscous colourless oil. δ_H (400 MHz, $CDCl_3$): 7.90–7.65 (15H, m, ArH of PPh_3), 7.35–7.23 (5H, m, ArH of

Bn), 4.49 (2H, s, CH_2Ph), 3.88–3.76 (2H, m, 1-H_2), 3.44 (2H, t, J = 6.7 Hz, 11-H_2), 1.67–1.53 (6H, m, 2-H_2, 3-H_2 and 10-H_2), 1.36–1.14 (12H, m, 4–9-H_2). δ_C (101 MHz, $CDCl_3$): 138.84 (C), 135.06 (d, *J* = 2.9 Hz, CH × 3), 133.86 (d, *J* = 10.0 Hz, CH × 6), 130.58 (d, *J* = 12.5 Hz, CH × 6), 128.45 (CH × 2), 127.75 (CH × 2), 127.57 (CH), 118.63 (d, *J* = 85.7 Hz, C × 3), 72.97 (CH_2), 70.66 (CH_2), 30.52 (d, *J* = 15.5 Hz, CH_2), 29.87 (CH_2), 29.58 (CH_2), 29.55 (CH_2), 29.53 (CH_2), 29.36 (CH_2), 29.26 (CH_2), 26.27 (CH_2), 22.91 (d, *J* = 49.5 Hz, CH_2), 22.80 (d, *J* = 4.6 Hz, CH_2). δ_P (162 MHz, $CDCl_3$): 24.49 (s). *m/z* (ESI) 523.3122 (M^+. $C_{36}H_{44}OP$ requires 523.3124). ^1H and ^{13}C NMR data agree with the literature.[8]

{2-[2'-(2"-Hydroxyethoxy)ethoxy]ethyl}triphenylphosphonium bromide TPP$_{3EG}$-OH

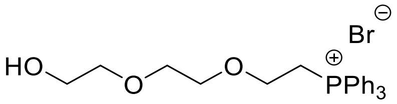

Alkyl bromide **9** (101 mg, 0.474 mmol) was dissolved in argon degassed anhydrous acetonitrile (10 mL), followed by addition of triphenylphosphine (248 mg, 0.948 mmol). The reaction vessel was then purged with argon and heated under reflux for 72 h. The reaction was then cooled and concentrated *in vacuo*. The crude material was purified by manual flash column chromatography on silica, eluting 10% methanol in dichloromethane to produce **TPP$_{3EG}$-OH** (129 mg, 57%) as a white solid. δ_H (400 MHz, $CDCl_3$): 7.72–7.85 (9H, m, ArH), 7.60–7.70 (6H, m, Ar-H), 4.10 (2H, dt, *J* = 11.6 and 5.7 Hz, P*CH₂*), 3.95 (2H, dt, *J* = 21.8 and 5.7 Hz, PCH_2*CH_2*), 3.63 (2H, t, *J* = 4.6 Hz, OCH_2), 3.34–3.40 (4H, m, 2 × OCH_2), 3.28–3.33 (2H, m, OCH_2) 2.88 (1H, br s, OH). δ_C (101 MHz, $CDCl_3$): 134.83 (d, *J* = 3.0 Hz, CH × 3), 134.07 (d, *J* = 10.3 Hz, CH × 6), 130.21 (d, *J* = 12.8 Hz, CH × 6), 118.98 (d, *J* = 86.8 Hz, C × 3), 72.45 (CH_2), 70.51 (CH_2), 70.06 (CH_2), 64.21 (d, *J* = 7.1 Hz, CH_2), 61.49 (CH_2), 25.48 (d, *J* = 52.6 Hz, CH_2). δ_P (162 MHz, $CDCl_3$): δ 25.23 (s). *m/z* (ESI) 395.1773 (M^+. $C_{24}H_{28}O_3P$ requires 395.1771).

{2-[2'-(2"-Acetoxyethoxy)ethoxy]ethyl}triphenylphosphonium bromide TPP$_{3EG}$-OAc

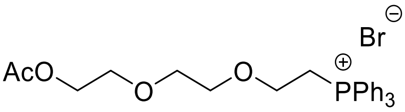

Alkyl bromide **11** (102 mg, 0.400 mmol) was dissolved in argon degassed anhydrous acetonitrile (10 mL), followed by addition of triphenylphosphine (209 mg, 0.800 mmol). The reaction vessel was then purged with argon and heated under reflux for 72 h. The reaction was then cooled and concentrated *in vacuo*. The crude material was purified by manual flash column chromatography on silica, eluting 10% methanol in dichloromethane to produce **TPP$_{3EG}$-OAc** (202 mg, 98%) as a viscous colourless oil. δ_H (400 MHz, $CDCl_3$): 7.81–7.89 (6 H, m, ArH), 7.72–7.78 (3 H, m, ArH,), 7.60–7.68 (6 H, m, ArH), 4.23 (2H, dt, *J* = 11.6 and 5.7 Hz, PCH_2), 4.08–4.04 (2H, m, AcOCH_2), 3.94 (2H, dt, *J* = 22.4, 5.7 Hz, PCH_2*CH_2*), 3.43–3.38 (2H, m, AcOCH_2*CH_2*), 3.32–3.37 (2H, m, OCH_2), 3.27–3.32 (2H, m, OCH_2), 2.03 (s, 3H, CH_3). δ_C (101 MHz, $CDCl_3$): 171.02 (C), 134.73 (d, *J* = 3.0 Hz, CH × 3), 134.22 (d, *J* = 10.3 Hz, CH × 6), 130.12 (d, *J* = 12.8 Hz, CH × 6), 119.15 (d, *J* = 86.9 Hz, C × 3), 70.34 (CH_2), 70.07 (CH_2), 68.95 (CH_2), 64.25 (d, *J* = 7.6 Hz, CH_2), 63.57 (CH_2), 25.52 (d, *J* = 52.6 Hz, CH_2), 21.09 (CH_3). δ_P (162 MHz, $CDCl_3$): 25.64 (s). *m/z* (ESI) 437.1877 (M^+. $C_{26}H_{30}O_4P$ requires 437.1876).

{2-[2'-(2"-Benxyloxyethoxy)ethoxy]ethyl}triphenylphosphonium bromide TPP$_{3EG}$-OBn

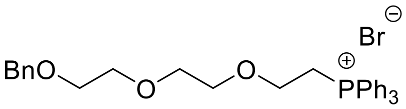

Triphenylphosphine (377 mg, 1.44 mmol, 2.0 eq) was added to solution of alkyl bromide **13** (173 mg, 0.72 mmol, 1.0 eq) in MeCN (2 mL) in a microwave vial. The vial was flushed with argon, sealed, and heated to 120 °C for 4 h (CAUTION Sealed tube). The reaction was cooled, concentrated *in vacuo* and the crude material was purified by column chromatography using a 12 g Agela cartridge elution DCM:MeOH (100:0) increasing to (92:8) over 10 column volumes to give the **TPP$_{3EG}$-OBn** as a colourless viscous oil (290 mg, 71%). δ_H (400 MHz, $CDCl_3$): 7.85–7.75 (6H, m, ArH of PPh_3), 7.74–7.66 (3H, m, ArH of PPh_3), 7.64–7.54 (6H, m, ArH of PPh_3), 7.33–7.19 (5H, m, ArH of Bn), 4.47 (2H, s, PhCH_2), 4.10 (2H, dt, *J* = 11.6 and 5.7 Hz, PCH_2), 3.89 (2H, dt, *J* = 22.4 and 5.7 Hz, PCH_2*CH_2*), 3.51–3.44 (2H, m, OCH_2), 3.41–3.34 (2H, m, OCH_2), 3.33–3.28 (2H, m, OCH_2), 3.28–3.22 (2H, m, OCH_2). δ_C (101 MHz, $CDCl_3$): 138.08 (C), 134.63 (d, *J* = 3.1 Hz, CH), 134.08 (d, *J* = 10.3 Hz, CH), 130.04 (d, *J* = 12.8 Hz, CH), 128.41 (CH), 127.84 (CH), 127.74 (CH), 118.94 (d, *J* = 86.9

Hz, C), 73.31 (CH_2), 70.41 (CH_2), 70.40 (CH_2), 69.99 (CH_2), 69.44 (CH_2), 64.07 (d, *J* = 7.5 Hz, (CH_2), 25.47 (d, *J* = 52.7 Hz, (CH_2). δ_P (162 MHz, $CDCl_3$): 25.47 (s). *m/z* (ESI) 485.2250 (M^+. $C_{31}H_{34}O_3P$ requires 485.2240).

(2-{2'-[2"-(2"'-Hydroxyethoxy)ethoxy]ethoxy}ethyl)triphenylphosphonium bromide TPP_{4EG}-OH

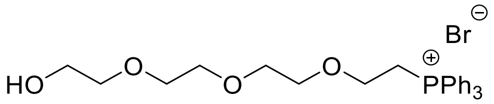

Alkyl bromide **10** (153 mg, 0.595 mmol) was dissolved in argon degassed anhydrous acetonitrile (10 mL), followed by addition of triphenylphosphine (312 mg, 1.19 mmol). The reaction vessel was then purged with argon and heated under reflux for 72 h. The reaction was then cooled and concentrated *in vacuo*. The crude material was purified by manual flash column chromatography on silica, eluting 5% methanol in dichloromethane to produce **TPP_{4EG}-OH** (275 mg, 89%) as a viscous colourless oil. δ_H (400 MHz, $CDCl_3$): δ 7.90–7.61 (15H, m, ArH × 15), 4.20 (2H, dt, *J* = 11.6 and 5.7 Hz, PCH_2), 3.97 (2H, dt, *J* = 22.1 and 5.7 Hz, PCH_2CH_2), 3.71 (2H, m, CH_2OH), 3.61–3.53 (4H, m, 2 × OCH_2), 3.47–3.40 (2 H, m, OCH_2), 3.38–3.31 (8 H, m, 4 × OCH_2), 2.82 (1H, br t, *J* = 5.8 Hz, OH). δ_C (101 MHz, $CDCl_3$): 134.74 (d, *J* = 3.1 Hz, CH × 3), 134.22 (d, *J* = 10.3 Hz, CH × 6), 130.18 (d, *J* = 12.8 Hz, CH × 6), 119.15 (d, *J* = 86.8 Hz, C × 3), 72.61 (CH_2), 70.48 (CH_2), 70.37 (CH_2), 70.27 (CH_2), 70.14 (CH_2), 64.24 (d, *J* = 7.1 Hz, CH_2), 61.64 (CH_2), 25.49 (d, *J* = 52.4 Hz, CH_2). δ_P (162 MHz, $CDCl_3$): 25.44 (s). *m/z* (ESI) 439.2033 (M^+. $C_{26}H_{32}O_4P$ requires 439.2033).

(2-{2'-[2"-(2"'-Acetoxyethoxy)ethoxy]ethoxy}ethyl)triphenylphosphonium bromide TPP_{4EG}-OAc

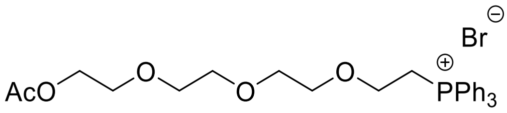

Alkyl bromide **12** (91.0 mg, 0.304 mmol) was dissolved in argon degassed anhydrous acetonitrile (10 mL), followed by addition of triphenylphosphine (159 mg, 0.608 mmol). The reaction vessel was then purged with argon and heated under reflux for 72 h. The reaction was then cooled and concentrated *in vacuo*. The crude material was purified by manual flash column chromatography on silica, eluting 5% methanol in dichloromethane to produce **TPP_{4EG}-OAc** (105 mg, 61%) as a viscous colourless oil. δ_H (400 MHz, $CDCl_3$): 7.69–7.84 (9 H, m, ArH), 7.57–7.66 (6 H, m, ArH), 4.16–4.11 (2H, m, $AcOCH_2$), 4.06 (2H, dt, *J* = 11.6 and 5.7 Hz, PCH_2), 3.88 (2H, dt, *J* = 22.2 and 5.7 Hz, PCH_2CH_2), 3.63–3.58 (2H, m, $AcOCH_2CH_2$), 3.52–3.47 (2H, m, OCH_2), 3.39–3.34 (2H, m, OCH_2), 3.32–3.28 (2H, m, OCH_2), 3.27–3.23 (2H, m, OCH_2), 2.00 (s, 3H, CH_3). δ_C (101 MHz, $CDCl_3$): 170.99 (C), 134.72 (d, *J* = 3.0 Hz, CH × 3), 134.09 (d, *J* = 10.4 Hz, CH × 6), 130.10 (d, *J* = 12.8 Hz, CH × 6), 118.95 (d, *J* = 86.9 Hz, C × 3), 70.55 (CH_2), 70.36 (CH_2), 70.32 (CH_2), 70.01 (CH_2), 69.12 (CH_2), 64.04 (d, *J* = 7.4 Hz, CH_2), 63.50 (CH_2), 25.48 (d, *J* = 52.8 Hz, CH_2), 20.98 (CH_3). δ_P (162 MHz, $CDCl_3$): 25.40 (s). *m/z* (ESI) 481.2137 (M^+. $C_{28}H_{34}O_5P$ requires 481.2138).

(2-{2'-[2"-(2"'-Benzyloxyethoxy)ethoxy]ethoxy}ethyl)triphenylphosphonium bromide TPP_{4EG}-OBn

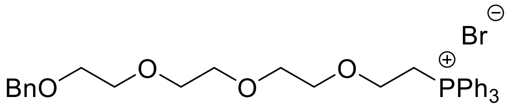

Triphenylphosphine (321 mg, 1.22 mmol, 2.0 eq) was added to solution of alkyl bromide **14** (213 mg, 0.61 mmol, 1.0 eq) in MeCN (2 mL) in a microwave vial. The vial was flushed with argon, sealed, and heated to 120 °C for 4 h (CAUTION Sealed tube). The reaction was cooled, concentrated *in vacuo* and the crude material was purified by column chromatography using a 12 g Agela cartridge elution DCM:MeOH (100:0) increasing to (90:10) over 10 column volumes to give the **TPP_{4EG}-OBn** as colourless viscous oil (348 mg, 93%). δ_H (400 MHz, $CDCl_3$) 7.80–7.66 (9H, m, ArH of PPh_3), 7.66–7.56 (6H, m, ArH of PPh_3), 7.30– 7.16 (5H, m, ArH of Bn), 4.47 (2H, s, $PhCH_2$), 3.98 (2H, dt, *J* = 11.4 and 5.7 Hz, PCH_2), 3.84 (2H, dt, *J* = 22.4 and 5.6 Hz, PCH_2CH_2), 3.62–3.52 (4H, m, 2 × OCH_2), 3.50–3.45 (2H, m, OCH_2), 3.36–3.32 (2H, m, OCH_2), 3.30–3.25 (2H, m, OCH_2), 3.26–3.21 (2H, m, OCH_2). δ_C (101 MHz, $CDCl_3$) 138.06 (C), 134.63 (d, *J* = 3.1 Hz, CH), 133.95 (d, *J* = 10.3 Hz, CH), 130.02 (d, *J* = 12.8 Hz, CH), 128.30 (CH), 127.66 (CH), 127.58 (CH), 118.75 (d, *J* = 86.9 Hz, C), 73.15 (CH_2), 70.51 (CH_2), 70.47 (CH_2), 70.31 (CH_2), 70.29 (CH_2), 69.88 (CH_2), 69.44 (CH_2), 63.87 (d, *J* = 7.4 Hz, (CH_2), 25.26 (d, *J* = 52.9 Hz, (CH_2). δ_P (162 MHz, $CDCl_3$): 25.27 (s). *m/z* (ESI) 529.2503 (M^+. $C_{33}H_{38}O_4P$ requires 529.2502).

4-Amino-*N*-(8'-triphenylphosphoniooct-1'-yl)-1,8-naphthalimide bromide LuciferTPPAlkyl

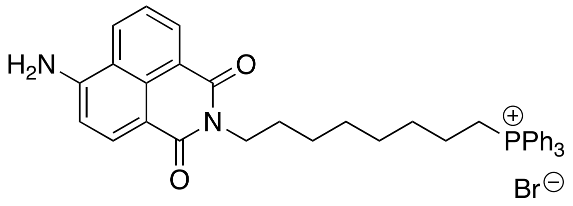

Triphenylphosphine (304 mg, 1.16 mmol, 2.0 eq) was added to a solution of alkyl bromide **21** (234 mg, 0.58 mmol, 1.0 eq) in MeCN (10 mL). The vial was flushed with argon and heated to 80°C overnight (CAUTION Sealed tube). After cooling to RT the product was concentrated under vacuum and purified by column chromatography using a 12 g Agela cartridge eluting DCM:MeOH (100:0) increasing to (93:7) over 10 column volumes to give phosphonium salt as a yellow solid (174 mg, 45%). δ_H (400 MHz, DMSO-d$_6$) 8.62 (1H, dd, *J* = 8.4 and 1.2 Hz, H-5 or H-7), 8.40 (1H, dd, *J* = 7.3 and 1.1 Hz, H-5 or H-7), 8.16 (1H, d, *J* = 8.4 Hz, H-2 or H-3), 7.93–7.86 (3H, m, Ar-H), 7.84–7.71 (12H, m, Ar-H), 7.64 (1H, dd, *J* = 8.4, 7.3 Hz, H-6), 7.45 (2H, s, NH_2), 6.84 (1H, d, *J* = 8.4 Hz, H-6), 3.97 (2H, t, *J* = 7.4 Hz, NCH_2), 3.64–3.46 (2H, m, PCH_2), 1.68–1.48 (4H, m, 2 × CH_2), 1.49–1.39 (2H, m, CH_2), 1.35–1.18 (6H, m, 3 × CH_2). δ_H (400 MHz, DMSO-d$_6$) 163.73 (C), 162.86 (C), 152.71 (C), 134.84 (CH, d, *J* = 3.1 Hz), 133.92 (CH), 133.56 (CH, d, *J* = 10.1 Hz), 130.96 (CH), 130.20 (CH, d, *J* = 12.4 Hz), 129.65 (C), 129.32 (CH), 123.97 (CH), 121.75 (C), 119.34 (C), 118.57 (C, d, *J* = 85.6 Hz), 108.14 (CH), 107.48 (C), 29.66 (CH_2, d, *J* = 16.7 Hz), 28.33 (CH_2), 27.81 (CH_2), 27.53 (CH_2), 26.36 (CH_2), 21.59 (CH_2, d, *J* = 4.2 Hz), 20.06 (CH_2, d, *J* = 49.9 Hz). NCH_2 obscured by solvent peak. *m/z* (ESI) 585.2666 (M^+. $C_{38}H_{38}N_2O_2P$ requires 585.2665).

4-Amino-*N*-{2'-[2"-(triphenylphosphonioethoxy)ethoxy]ethyl}-1,8-naphthalimide bromide LuciferTPPGlycol

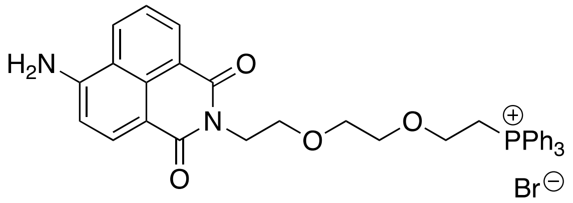

In a microwave vial, triphenylphosphine (37 mg, 0.162 mmol, 3.0 eq) was added to a solution of alkyl bromide **22** (22 mg, 0.054 mmol, 1.0 eq) in MeCN (1 mL). The vial was flushed with argon, sealed and heated to 120°C overnight (CAUTION Sealed tube). After cooling to RT the product was concentrated under vacuum and purified by column chromatography using a 12 g Agela cartridge eluting DCM:MeOH (100:0) increasing to (85:15) over 10 column volumes to give phosphonium salt as a yellow solid (25 mg, 69%). δ_H (400 MHz, DMSO-d$_6$) 8.63 (1H, dd, *J* = 8.5 and 1.2 Hz, H-5 or H-7), 8.43 (1H, dd, *J* = 7.3 and 1.1 Hz, H-5 or H-7), 8.19 (1H, d, *J* = 8.4 Hz, H-2 or H-3), 7.89–7.70 (16H, m, ArH of PPh_3 and H-2 or H-3), 7.66 (1H, dd, *J* = 8.4 and 7.3 Hz, H-6), 7.50 (2H, s, NH_2), 6.85 (1H, d, *J* = 8.4 Hz, H-2 or H-3), 4.11 (2H, t, *J* = 6.6 Hz, NCH_2), 3.86 (2H, dt, *J* 12.5 and 5.9 Hz, PCH_2), 3.65 (2 H, dt, *J* = 20.7 and 5.9 Hz, PCH_2*CH_2*)3.42 (2 H, t, *J* = 6.6 Hz, NCH_2*CH_2*), 3.31–3.22 (4 H, m, 2 × OCH_2). δ_C (101 MHz, DMSO-d$_6$) 163.84 (C), 162.89 (C), 152.88 (C), 134.62 (d, *J* = 3.1 Hz, CH), 134.07 (CH), 133.72 (d, *J* = 10.4 Hz, CH), 131.13 (CH), 129.91 (d, *J* = 12.6 Hz, CH), 129.77 (C), 129.52 (CH), 124.04 (CH), 121.66 (C), 119.39 (C), 118.95 (d, *J* = 85.0 Hz, (C), 108.23 (CH), 107.34 (C), 69.88 (CH_2), 69.17 (CH_2), 67.19 (CH_2), 63.25 (d, *J* = 6.4 Hz, (CH_2), 38.26 (CH_2), 22.83 (d, *J* = 52.0 Hz, (CH_2). δ_P (162 MHz, DMSO) 24.80. *m/z* (ESI) 589.2263 (M^+. $C_{36}H_{34}N_2O_4P$ requires 589.2251).

4-Amino-*N*-hexyl-1,8-naphthalimide LuciferAlkyl

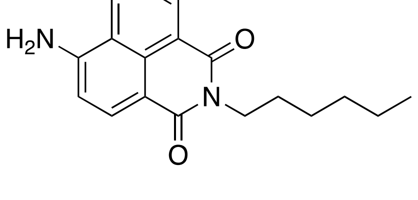

Argon was bubbled through a stirred solution of 4-nitronaphthalimide **23** (200 mg, 613 µmol) in ethanol (10 mL) 5 min. Pd/C (20 mg, 19 µmol) was added to the stirred solution under a stream of argon. Argon was bubbled through the stirred solution for 10 min. Hydrogen gas was then bubbled through the solution for 10 min and the reaction mixture left to stir for 10 h at RT under a hydrogen atmosphere. Argon was bubbled through the mixture for 5 min, and then

the mixture was filtered through celite, and concentrated *in vacuo* to yield **LuciferAlkyl** (180 mg, 99%) as an amorphous orange solid. M.P. 148-149 °C. δ_H (400 MHz, DMSO-d$_6$): 8.61 (1H, d, J = 7.8 Hz, H-5 or H-7), 8.42 (1H, d, J = 6.5 Hz, H-5 or H-7), 8.18 (1H, d, J = 7.9 Hz, H-2 or H-3), 7.65 (1H, t, J = 7.6 Hz, H-6), 7.43 (2H, s, NH_2), 6.83 (1H, d, J = 8.2 Hz, H-2 or H-3), 3.99 (2H, t, J = 6.7 Hz, NCH_2), 1.68-1.48 (2H, m, CH_2), 1.39-1.11 (6H, m, 3 × CH_2), 0.93-0.76 (3H, m, CH_3). δ_C (101 MHz, DMSO-d$_6$): 163.77 (C), 162.91 (C), 152.69 (C), 133.93 (CH), 130.97 (CH), 129.69 (C), 129.27 (CH), 123.97 (CH), 121.83 (C), 119.39 (C), 108.17 (CH), 107.61 (C), 39.17 (CH_2), 31.00 (CH_2), 27.61 (CH_2), 26.23 (CH_2), 21.99 (CH_2), 13.88 (CH_3). HRMS (ESI): *m/z* 297.1597 [(M+H)$^+$. $C_{18}H_{21}N_2O_2$ requires 297.1598].

3.2 Synthetic Intermediates (Ordered by Compound Number)

8-Bromooct-1-yl acetate 3

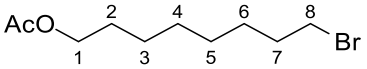

8-Bromo-1-octanol **2** (212 mg, 1.014 mmol) was dissolved in anhydrous dichloromethane (10 mL) and cooled to 0 °C. Acetyl chloride (94.0 µL, 1.318 mmol) was added followed by triethylamine (212 µL, 1.521 mmol). The reaction was allowed to warm to rt over 19 h. The reaction was then cooled to 0 °C and quenched by addition of a saturated aqueous solution of ammonium chloride (5 mL). The solution was diluted with water (10 mL), extracted with dichloromethane (10 mL × 3), dried over $MgSO_4$, filtered and concentrated *in vacuo*. The crude material was purified by manual flash column chromatography on silica, eluting 5% ethyl acetate in petroleum ether (40–60) to produce ester **3** (210 mg, 83%) as a colourless oil. δ_H (400 MHz, $CDCl_3$): δ 4.05 (2H, t, *J* = 6.7 Hz, 1-H_2), 3.41 (2H, t, *J* = 6.8 Hz, 8-H_2), 2.04 (3H, s, CH_3), 1.90–1.79 (2H, m, 7-H_2), 1.67–1.57 (2H, m, 2-H_2), 1.49–1.28 (8H, m, 3–6-H_2). δ_C (101 MHz, $CDCl_3$): 171.35 (C), 64.69 (CH_2), 34.06 (CH_2), 32.90 (CH_2), 29.18 (CH_2), 28.77 (CH_2), 28.71 (CH_2), 28.20 (CH_2), 25.95 (CH_2), 21.15 (CH_3). *m/z* (ESI) 273.0461 [(M+H)$^+$. $C_{10}H_{19}{}^{79}BrNaO_2$ requires 273.0461]. ^{1}H and ^{13}C NMR data agree with the literature.[9]

11-Bromoundec-1-yl acetate 4

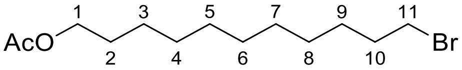

11-Bromo-1-undecanol **2** (599 mg, 2.384 mmol) was dissolved in anhydrous dichloromethane (10 mL) and cooled to 0 °C. Acetyl chloride (220 µL, 3.010 mmol) was added followed by triethylamine (498 µL, 3.577 mmol). The reaction was allowed to warm to rt over 20 h. The reaction was then cooled to 0 °C and quenched by addition of a saturated aqueous solution of ammonium chloride (5 mL). The solution was diluted with water (10 mL), extracted with dichloromethane (10 mL × 3), dried over $MgSO_4$, filtered and concentrated *in vacuo*. The crude material was purified by manual flash column chromatography on silica, eluting 10% ethyl acetate in petroleum ether (40–60) to produce ester **4** (677 mg, 97%) as a colourless oil. δ_H (400 MHz, $CDCl_3$): 4.05 (2H, t, J = 6.8 Hz, 1-H_2), 3.41 (2H, t, J = 6.9 Hz, 11-H_2), 2.04 (3H, s, CH_3), 1.91–1.80 (2H, m, 10-H_2), 1.67–1.56 (2H, m, 2-H_2), 1.47–1.23 (14H, m, 3–9-H_2). δ_C (101 MHz, $CDCl_3$): 171.37 (C), 64.78 (CH_2), 34.15 (CH_2), 32.98 (CH_2), 29.59 (CH_2), 29.57 (CH_2), 29.53 (CH_2), 29.37 (CH_2), 28.89 (CH_2), 28.75 (CH_2), 28.31 (CH_2), 26.04 (CH_2), 21.16 (CH_3). *m/z* (ESI) 315.0930 [(M+Na)$^+$. $C_{13}H_{25}{}^{79}BrNaO_2$ requires 315.0930]. ^{1}H and ^{13}C NMR data agree with the literature.[10]

11-Bromoundec-1-yl benzyl ether 6

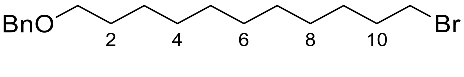

11-Bromo-1-undecanol **2** (518 mg, 2.062 mmol) was dissolved in anhydrous tetrahydrofuran (15 mL) and cooled to 0 °C. Sodium hydride (86.0 mg, 2.165 mmol, 60% dispersion in mineral oil) was added and the solution was allowed to stir for 15 minutes. Benzyl bromide (294 µL, 2.475 mmol) and tetrabutylammonium iodide (76.0 mg, 0.2062 mmol) were added and the mixture was heated under reflux for 6 h. The reaction was then cooled to 0 °C and quenched by addition of a saturated aqueous solution of ammonium chloride (10 mL). The solution was diluted with water (10 mL), extracted with ethyl acetate (10 mL × 3), dried over $MgSO_4$, filtered and concentrated *in vacuo*. The crude material was purified by manual flash column chromatography on silica, eluting 30% dichloromethane in petroleum ether (40–60) to produce benzyl ether **6** (256 mg, 36%) as a colourless oil. δ_H (400 MHz, $CDCl_3$): 7.36–7.25 (5H, m, ArH × 5), 4.50

(s, 2H, C*H₂*Ph), 3.46 (2H, t, J = 6.6 Hz, 1-H_2), 3.41 (2H, t, J = 6.9 Hz, 11-H_2), 1.90–1.80 (2H, m, 10-H_2), 1.66–1.56 (2H, m, 2-H_2), 1.47–1.23 (14H, m, 3–9-H_2). δ_C (101 MHz, $CDCl_3$): 138.90 (C), 128.48 (CH × 2), 127.76 (CH × 2), 127.60 (CH), 73.01 (CH_2), 70.68 (CH_2), 34.18 (CH_2), 33.00 (CH_2), 29.93 (CH_2), 29.68 (CH_2), 29.61 (CH_2 × 2), 29.56 (CH_2), 28.91 (CH_2), 28.33 (CH_2), 26.34 (CH_2). *m/z* (ESI) 363.1294 (M+Na)$^+$. $C_{18}H_{29}{}^{79}BrNaO$ requires 363.1294). ^1H and ^{13}C NMR data agree with the literature.[11]

2-[2′-(2″-Bromoethoxy)ethoxy]ethanol 9

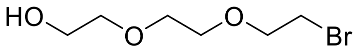

Triethylene glycol **7** (534 mg, 3.556 mmol) was dissolved in toluene (10 mL) and 48% hydrogen bromide in water (2.01 mL, 17.78 mmol) was added. The mixture was heated under reflux for 4 h. The reaction was then cooled and concentrated *in vacuo*. The crude material was purified by manual flash column chromatography on silica, eluting 1% methanol in diethyl ether to produce alkyl bromide **9** (124 mg, 16%) as a colourless oil. δ_H (400 MHz, $CDCl_3$): 3.82 (2H, t, *J* = 6.2 Hz, CH_2CH_2Br), 3.70–3.78 (2H, m, OCH_2), 3.69 (4H, s, 2 × OCH_2), 3.65–3.60 (2H, m, OCH_2), 3.48 (2H, t, *J* = 6.2 Hz, CH_2Br), 2.28 (br s, 1H, OH). δ_C (101 MHz, $CDCl_3$): 72.61 (CH_2), 71.31 (CH_2), 70.67 (CH_2), 70.50 (CH_2), 61.95 (CH_2), 30.36 (CH_2). *m/z* (ESI) 234.9941 [(M+Na)$^+$. $C_6H_{13}{}^{79}BrNaO_3$ requires 234.9940]. ^1H and ^{13}C NMR data agree with the literature.[12]

2-{2'-[2"-(2"'-Bromoethoxy)ethoxy]ethoxy}ethanol 10

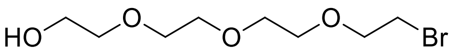

Tetraethylene glycol **8** (517 mg, 2.662 mmol) was dissolved in toluene (10 mL) and 48% hydrogen bromide in water (1264 µL, 11.18 mmol) was added. The mixture was heated under reflux for 48 h. The reaction was then cooled and concentrated *in vacuo*. The crude material was purified by manual flash column chromatography on silica, eluting 2% methanol in diethyl ether to produce alkyl bromide **10** (185 mg, 27%) as a colourless oil. δ_H (400 MHz, $CDCl_3$): 3.81 (2H, t, *J* = 6.3 Hz, OCH_2), 3.75–3.71 (2H, m, OCH_2) 3.70–3.65 (8H, m, 4 × OCH_2), 3.64–3.59 (2H, m, OCH_2), 3.48 (2H, t, *J* = 6.3 Hz, CH_2Br), 2.47 (1H, br s, OH). δ_C (101 MHz, $CDCl_3$): 72.62 (CH_2), 71.38 (CH_2), 70.84 (CH_2), 70.70 (CH_2), 70.67 (CH_2), 70.52 (CH_2), 61.93 (CH_2), 30.37 (CH_2). *m/z* (ESI) 279.0203 [(M+Na)$^+$. $C_8H_{17}{}^{79}BrNaO_4$ requires 279.0202]. ^1H and ^{13}C NMR data agree with the literature.[12]

2-[2-(Bromoethoxy)ethoxy]ethyl acetate 11

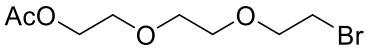

Alcohol **9** (124 mg, 0.4861 mmol) was dissolved in anhydrous dichloromethane (5 mL) and cooled to 0 °C. Acetyl chloride (45.0 µL, 0.6319 mmol) was added followed by triethylamine (101 µL, 0.7291 mmol). The reaction was allowed to warm to rt over 20 h. The reaction was then cooled to 0 °C and quenched by addition of a saturated aqueous solution of ammonium chloride (5 mL). The solution was diluted with water (10 mL), extracted with dichloromethane (10 mL × 3), dried over $MgSO_4$, filtered and concentrated *in vacuo*. The crude material was purified by manual flash column chromatography on silica, eluting 5% ethyl acetate in petroleum ether (40–60) to produce acetate ester **11** (127 mg, 87%) as a colourless oil. δ_H (400 MHz, $CDCl_3$): 4.25–4.20 (2H, m, AcO*CH₂*), 3.81 (2H, t, *J* = 6.3 Hz, $BrCH_2$*CH₂*), 3.69–3.73 (2 H, m, $AcOCH_2$*CH₂*), 3.64–3.69 (4 H, m, 2 × OCH_2), 3.47 (2H, t, *J* = 6.3 Hz, Br*CH₂*), 2.08 (3H, s, CH_3). ^{13}C NMR (101 MHz, $CDCl_3$) δ 171.18 (C), 71.42 (CH_2), 70.73 (CH_2), 70.70 (CH_2), 69.38 (CH_2), 63.70 (CH_2), 30.38 (CH_2), 21.10 (CH_3). *m/z* (ESI) 277.0047 [(M+Na)$^+$. $C_8H_{15}{}^{79}BrNaO_4$ requires 277.0046]. ^1H NMR data agree with the literature.[13]

2-{2-[2-(Bromoethoxy)ethoxy]ethoxy}ethyl acetate 12

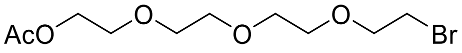

Alcohol **10** (125 mg, 0.4861 mmol) was dissolved in anhydrous dichloromethane (5 mL) and cooled to 0 °C. Acetyl chloride (45.0 µL, 0.6319 mmol) was added followed by triethylamine (101 µL, 0.7291 mmol). The reaction was allowed to warm to rt over 20 h. The reaction was then cooled to 0 °C and quenched by addition of a saturated aqueous solution of ammonium chloride (5 mL). The solution was diluted with water (10 mL), extracted with dichloromethane

(10 mL × 3), dried over $MgSO_4$, filtered and concentrated *in vacuo*. The crude material was purified by manual flash column chromatography on silica, eluting 5% ethyl acetate in petroleum ether (40–60) to produce acetate ester **12** (127 mg, 87%) as a colourless oil. δ_H (400 MHz, $CDCl_3$): 4.25–4.20 (2H, m, AcO*CH₂*), 3.81 (2H, t, *J* = 6.3 Hz, *CH₂*CH₂Br), 3.68–3.72 (2H, m, AcOCH₂*CH₂*), 3.73–3.64 (m, 8H, 4 × OCH_2), 3.47 (2H, t, *J* = 6.3 Hz, *CH₂*Br), 2.08 (s, 3H, CH_3). δ_C (101 MHz, $CDCl_3$): 171.17 (C), 71.38 (CH_2), 70.82 (CH_2), 70.80 (CH_2), 70.75 (CH_2), 70.71 (CH_2), 69.31 (CH_2), 63.74 (CH_2), 30.42 (CH_2), 21.11 (CH_3). *m/z* (ESI) 321.0311 [(M+Na)$^+$. $C_{10}H_{19}{}^{79}BrNaO_5$ requires 321.0308]. ^{1}H and ^{13}C NMR data agree with the literature.[14]

N-(8'-Hydroxyoct-1'-yl)-4-nitro-1,8-naphthalimide 17

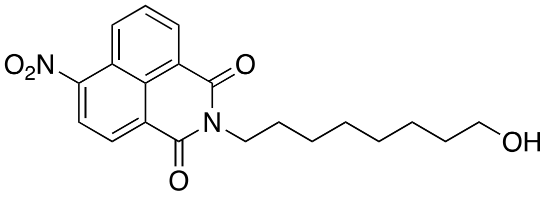

8-Amino-octan-1-ol **15** (3.62 g, 24.92 mmol, 1.0 eq) was added to a suspension of 4-nitro-1,8-naphthalic anhydride (6.66 g, 27.41 mmol, 1.1 eq) in EtOH (80 mL) then heated to 70°C overnight under an atmosphere of argon. After cooling to RT and concentration under vacuum the resulting viscous oil was purified by column chromatography eluting dichloromethane to elute unreacted starting material then EtOAc to elute the product. After concentration under vacuum the naphthalimide **17** was obtained as a beige solid (3.70 g, 40%). δ_H (400 MHz, $CDCl_3$) 8.84 (1H, dd, *J* 8.8 and 1.1 Hz, H-5 or H-7), 8.74 (1H, dd, *J* = 7.3 and 1.1 Hz, H-5 or H-7), 8.69 (1H, d, *J* = 8.0 Hz, H-2 or H-3), 8.41 (1H, d, *J* = 8.0 Hz, H-2 or H-3), 7.99 (1H, dd, *J* = 8.8 and 7.3 Hz, H-6), 4.12–4.25 (2H, m, N*CH₂*CH₂), 3.64 (2H, t, *J* = 6.6 Hz, *CH₂*OH), 1.69–1.80 (2H, m, NCH₂*CH₂*), 1.32–1.51 (4H, m, 2 × CH_2), 1.32–1.51 (6H, m, 3 × CH_2), 1.24 (1H, s, OH). δ_C (101 MHz, $CDCl_3$) 163.46 (C), 162.63 (C), 149.71 (C), 132.58 (CH), 130.08 (CH), 129.93 (CH), 129.44 (CH), 129.25 (C), 127.19 (C), 124.05 (CH), 123.84 (C), 123.21 (C), 63.18 (CH_2), 41.00 (CH_2), 32.87 (CH_2), 29.37 (CH_2), 29.35 (CH_2), 28.09 (CH_2), 27.11 (CH_2), 25.79 (CH_2). *m/z* (ESI) 371.1609 [(M+H)$^+$. $C_{20}H_{23}N_2O_5$ requires 371.1601].

N-{2'-[2"-(Hydroxyethoxy)ethoxy]ethyl}-4-nitro-1,8-naphthalimide 18

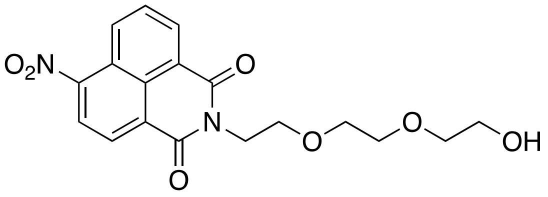

Amino alcohol **16** (1.00 g, 5.71 mmol, 1.0 eq) was added to a suspension of 4-nitro-1,8-naphthalic anhydride (1.52 g, 6.27 mmol, 1.1 eq) in EtOH (20 mL) then heated to 90°C overnight under an atmosphere of argon. After cooling to RT and concentration under vacuum the resulting viscous oil was purified by column chromatography using a 40g Agela cartridge eluting dichloromethane:EtOAc (50:50) increasing to EtOAc (100%) over 10 column volumes to give the napthalimide **18** as a yellow solid (2.13 g, 79%). δ_H (400 MHz, $CDCl_3$) 8.82 (1H, dd, *J* 8.8 and 1.1 Hz, H-5 or H-7), 8.72 (1H, dd, *J* 7.3 and 1.1 Hz, H-5 or H-7), 8.68 (1H, d, *J* = 8.0 Hz, H-2 or H-3), 8.39 (1H, d, *J* = 8.0 Hz, H-2 or H-3), 7.97 (1H, dd, *J* 8.7 and 7.3 Hz, H6), 4.45 (2H, t, *J* 5.8 Hz, NCH_2), 3.86 (2H, t, *J* 5.8 Hz, NCH₂*CH₂*), 3.71–3.68 (2H, m, OCH_2), 3.65–3.59 (4H, m, 2 × OCH_2), 3.55–3.51 (2 H, m, OCH_2), 2.53 (1 H, v broad s, OH). δ_C (101 MHz, $CDCl_3$) 163.56 (C), 162.76 (C), 149.74 (C), 132.65 (CH), 130.05 (2 × CH), 129.52 (CH), 129.26 (C), 126.99 (C), 124.01 (CH), 123.78 (C), 123.04 (C), 72.53 (CH_2), 70.56 (CH_2), 70.26 (CH_2), 68.00 (CH_2), 61.85 (CH_2), 39.69 (CH_2). *m/z* (ESI) 397.1009 [(M+Na)$^+$. $C_{18}H_{18}N_2O_7Na$ requires 397.1006].

4-Amino-N-(8'-hydroxyoct-1'-yl)-1,8-naphthalimide 19

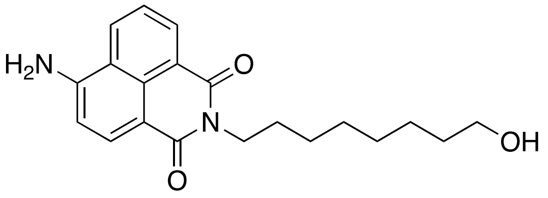

Palladium on carbon (10% Pd, 200 mg) was added to a solution 4-nitronaphthalimide **17** (3.70 g, 9.99 mmol, 1.0 eq) in MeOH (200 mL). The solution was placed under an atmosphere of hydrogen and stirred at RT overnight. The solution was passed through a plug of celite and washed with MeOH to give the amine as brown solid (2.89 g, 85%). A small amount was recrystallised from toluene for characterisation to give the amine **19** as a yellow solid. δ_H (400 MHz, DMSO-d_6) 8.60 (1H, dd, *J* = 8.4 and 1.2 Hz, H-5 or H-7), 8.42 (1H, dd, *J* = 7.3 and 1.1 Hz, H-5 or H-7), 8.18 (1H, d, *J* = 8.4 Hz, H-2 or H-3), 7.64 (1H, dd, *J* = 8.4 and 7.3 Hz, H-6), 7.42 (2 H, s, NH_2), 6.84 (1H, d, *J* = 8.4 Hz, H-2 or H-3), 4.30 (1H, t, *J*= 5.2 Hz, OH), 3.98 (2H, t, *J* = 7.5 Hz, NCH_2), 3.36 (2H, td, *J* = 6.5 and 5.2 Hz, CH_2OH), 1.58 (2H, qn, *J* = 6.3 Hz, NCH_2CH_2), 1.38 (2H, q, *J* = 6.7 Hz, CH_2CH_2OH), 1.35–1.18 (8 H, m, 4 × CH_2). δ_C (101 MHz, DMSO-d_6) 163.74 (C), 162.88 (C), 152.67 (C), 133.93 (CH), 130.97 (CH), 129.66 (C), 129.26 (CH), 123.96 (CH), 121.80 (C), 119.36 (C), 108.15 (CH), 107.57 (C), 60.70 (CH_2), 39.17 (CH_2) (obscured by solvent signal), 32.52 (CH_2), 28.85 (CH_2), 28.83 (CH_2), 27.64 (CH_2), 26.55 (CH_2), 25.46 (CH_2). *m/z* (ESI) 341.1869 [$(M+H)^+$. $C_{20}H_{24}N_2O_3$ requires 341.1860].

4-Amino-*N*-{2'-[2"-(hydroxyethoxy)ethoxy]ethyl}-1,8-naphthalimide 20

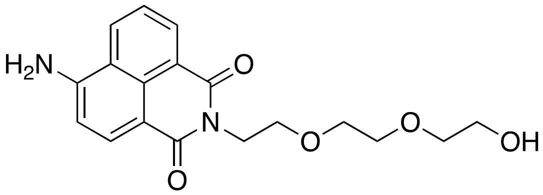

Palladium on carbon (10% w/w Pd, 50 mg) was added to a solution 4-nitronaphthalimide **18** (500 mg, 1.33 mmol, 1.0 eq) in MeOH (30 mL). The solution was placed under an atmosphere of hydrogen and stirred at RT overnight. The solution was passed through a plug of celite and washed with MeOH to give the amine as brown solid (359 g, 78%). δ_H (500 MHz, DMSO-d_6) 8.63–8.58 (1H, d, *J* = 8.4 Hz, H-5 or H-7), 8.43–8.38 (1H, d, *J* = 7.2 Hz, H-5 or H-7), 8.18 (1H, d, *J* = 8.3 Hz, H-2 or H-3), 7.64 (1H, apparent t, *J* = 7.8 Hz, H-6), 7.46 (2H, s, NH_2), 6.83 (1H, d, *J* = 8.3 Hz, H-2 or H-3), 4.55 (1H, t, *J* = 5.3 Hz, OH), 4.19 (2H, t, *J* = 6.6 Hz, NCH_2), 3.61 (2H, t, *J* = 6.6 Hz, NCH_2CH_2), 3.55 (2H, dd, *J* = 5.9 and 3.7 Hz, OCH_2), 3.48 (2H, dd, *J* = 5.9 and 3.7 Hz, OCH_2), 3.39–3.45 (2H, m, OCH_2), 3.38–3.33 (2H, m, OCH_2). δ_C (126 MHz, DMSO-d_6) 163.83 (C), 162.88 (C), 152.80 (C), 134.02 (CH), 131.06 (CH), 129.74 (C), 129.40 (CH), 123.97 (CH), 121.68 (C), 119.35 (C), 108.18 (CH), 107.39 (C), 72.36 (CH_2), 69.67 (CH_2), 69.60 (CH_2), 67.11 (CH_2), 60.17 (CH_2), 38.28 (CH_2). *m/z* (ESI) 367.1266 [$(M+Na)^+$. $C_{18}H_{20}N_2O_5Na$ requires 367.1264].

4-Amino-*N*-(8'-bromooct-1'-yl)-1,8-naphthalimide 21

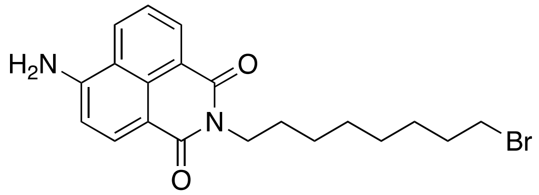

Triphenylphoshine (272 mg, 1.04 mmol, 1.4 eq) was added to a suspension of carbon tetrabromide (294 mg, 0.88 mmol, 1.2 eq) and alcohol **19** (252 mg, 0.74 mmol, 1.0 eq) in DCE (10 mL). After stirring at RT under argon for 2h the solution was purified directly using a 12g Agela cartridge eluting 100% DCM to give the bromide as a beige solid (273 mg, 58%). A small amount was recrystallised from toluene to give the alkyl bromide **21** as a yellow solid. δ_H (400 MHz, DMSO-d_6) 8.60 (1H, d, *J* = 8.4 Hz, H-5 or H-7), 8.41 (1H, d, *J* = 7.3 Hz, H-2 or H-3), 8.18 (1H, d, *J* = 8.4 Hz, H-5 or H-7), 7.64 (1H, apparent t, *J* = 7.9 Hz, H-6), 7.42 (2H, s, NH_2), 6.84 (1H, d, *J* = 8.4 Hz, H-2 or H-3), 3.98 (2H, t, *J* = 7.4 Hz, NCH_2), 3.49 (2H, t, *J* = 6.7 Hz, CH_2Br), 1.75 (2H, qn, *J* = 6.8 Hz, CH_2CH_2Br), 1.57 (2H, q, *J* = 7.1 Hz, CH_2CH_2N), 1.40–1.22 (8H, m 4 × CH_2). δ_C (101 MHz, DMSO-d_6) 163.72 (C), 162.86 (C), 152.64 (C), 133.88 (CH), 130.93 (CH), 129.64 (CH), 129.23 (C), 123.92 (CH), 121.77 (C), 119.35 (C), 108.13 (CH), 107.57 (C), 39.07 (CH_2) (obscured by solvent signal), 35.09 (CH_2), 32.17 (CH_2), 28.55 (CH_2), 27.94 (CH_2), 27.56 (CH_2), 27.42 (CH_2), 26.41 (CH_2). *m/z* (ESI) 403.1006 [$(M+H)^+$. $C_{20}H_{24}{}^{79}BrN_2O_2$ requires 403.1016].

4-Amino-*N*-{2'-[2"-(bromoethoxy)ethoxy]ethyl}-1,8-naphthalimide 22

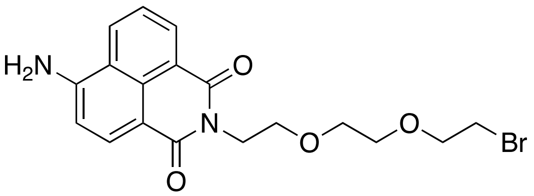

Triphenylphosphine (85 mg, 0.32 mmol, 1.4 eq) was added to a solution of carbon tetrabromide (93 mg, 0.28 mmol, 1.2 eq) and alcohol **20** (80 mg, 0.23mmol, 1.0 eq) in DCE (2 mL). The solution was stirred at RT under argon for 3h. The solution was then purified directly using a 12g Agela cartridge eluting DCM:EtOAc (100:0) increasing to (60:40) over 10 column volumes. The product containing fractions were concentrated under vacuum and the residue recrystallised from toluene to give the alkyl bromide **22** as an orange solid (46 mg, 49%). δ_H (500 MHz, DMSO-d$_6$) 8.61 (1H, dd, J = 8.4 and 1.2 Hz, H-5 or H-7), 8.42 (1H, dd, J = 7.3 and 1.1 Hz, H-5 or H-7), 8.18 (1H, d, J = 8.4 Hz, H-2 or H-3), 7.64 (1H, dd, J = 8.4 and 7.3 Hz, H-6), 7.45 (2H, s, NH_2), 6.84 (1H, d, J = 8.4 Hz, H-2 or H-3), 4.20 (2H, t, J = 6.5 Hz, NCH_2)), 3.66 (2H, t, J = 5.8 Hz, CH_2Br), 3.63 (2H, t, J = 6.5 Hz, $NCH_2\mathit{CH_2}$), 3.57–3.54 (2H, m, OCH_2), 3.51–3.54 (2H, m, OCH_2), 3.47 (2H, t, J = 5.8 Hz, $\mathit{CH_2}CH_2Br$). δ_C (126 MHz, DMSO-d$_6$) 163.83 (C), 162.89 (C), 152.78 (C), 134.02 (CH), 131.07 (CH), 129.74 (C), 129.37 (CH), 123.98 (CH), 121.70 (C), 119.36 (C), 108.18 (CH), 107.43 (C), 70.33 (CH_2), 69.56 (CH_2), 69.47 (CH_2), 67.17 (CH_2), 38.29 (CH_2), 32.12 (CH_2). *m/z* (ESI) 429.0431 [(M+Na)$^+$. $C_{18}H_{19}BrN_2O_5Na$ requires 429.0420].

***N*-Hexyl-4-nitro-1,8-naphthalimide 23**

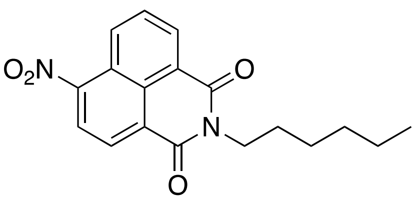

4-Nitro-1,8-naphthalic anhydride (1.00 g, 4.22 mmol) was added to a stirred solution of hexylamine (517 µL, 3.91 mmol) in EtOH (25 mL) and the mixture stirred under reflux for 17 h. The mixture was then cooled to RT, concentrated *in vacuo*, and column chromatography [SiO_2, EtOAc-hexane (20:80)–(100:0)] yielded naphthalimide **23** (723 mg, 54%) as pale-yellow needles R_f [SiO_2, EtOAc-hexane (50:50)]: 0.27. M.P. 145-146 °C. δ_H (400 MHz, $CDCl_3$): 8.84 (1H, dd, J = 8.8 and 1.1 Hz, H-5 or 7), 8.74 (1H, dd, J = 7.3 and 1.0 Hz, H-5 or 7), 8.69 (1H, d, J = 8.0 Hz, H-2 or 3), 8.40 (1H, d, J = 8.0 Hz, H-2 or 3), 7.99 (1H, dd, J = 8.8 and 7.3 Hz, H-6), 4.17 (1H, t, J = 7.6 Hz, NCH_2), 1.78-1.68 (2H, m, CH_2) 1.48-1.27 (6H, m, 3 × CH_2), 0.93-0.85 (3H, m, CH_3). (δ_C (101 MHz, $CDCl_3$): 163.44 (C), 162.62 (C), 149.72 (C), 132.56 (CH), 130.08 (CH), 129.91 (CH), 129.41 (CH), 129.26 (C), 127.23 (C), 124.04 (CH), 123.85 (C), 123.26 (C), 41.06 (CH_2), 31.64 (CH_2), 28.12 (CH_2), 26.89 (CH_2), 22.69 (CH_2), 14.18 (CH_3). *m/z* (ESI) 327.1342 [(M+H)$^+$. $C_{18}H_{19}N_2O_4$ requires 327.1339].

4. References

1. A. H. G. David, P. García-Cerezo, A. G. Campaña, F. Santoyo-González and V. Blanco, *Chem. Eur. J.*, 2019, **25**, 6170–6179.
2. M. Felber, M. Bauwens, J. M. Mateos, S. Imstepf, F. M. Mottaghy and R. Alberto, *Chem. Eur. J.*, 2015, **21**, 6090–6099.
3. P. A. Puchkov, I. A. Kartashova, E. V. Shmendel, A. S. Luneva, N. G. Morozova, M. A. Zenkova and M. A. Maslov, *Bioorg. Med. Chem. Lett.*, 2017, **27**, 3284–3288.
4. C. Salvagnini, S. Gharbi, T. Boxus and J. Marchand-Brynaert, *Eur. J. Med. Chem.*, 2007, **42**, 37–53.
5. H. Lei and J. Atkinson, *J. Org. Chem.*, 2000, **65**, 2560–2567.
6. M. Catalán, V. Castro-Castillo, J. Gajardo-de la Fuente, J. Aguilera, J. Ferreira, R. Ramires-Fernandez, I. Olmedo, A. Molina-Berríos, C. Palominos, M. Valencia, M. Domínguez, J. A. Souto and J. A. Jara, *RSC Med. Chem.*, 2020, **11**, 1210–1225.
7. T. Iwasaki, Y. Tajimi, K. Kameda, C. Kingwell, W. Wcislo, K. Osaka, M. Yamawaki, T. Morita and Y. Yoshimi, *J. Org. Chem.*, 2019, **84**, 8019–8026.
8. T. Muller, D. Coowar, M. Hanbali, P. Heuschling and B. Luu, *Tetrahedron*, 2006, **62**, 12025–12040.
9. N. Vicart, G.-S. Saboukoulou, Y. Ramondenc and G. Plé, *Synth. Commun.*, 2003, **33**, 1509–1521.
10. J.-S. Chen, P.-H. Huang, Y.-C. Hsieh, J.-W. Liu, H.-L. Hsu, K.-M. Zhang, R.-T. Wu, T.-S. Chang, Y.-H. Liu, H.-R. Wu and S.-Y. Luo, *Synthesis*, 2022, **54**, 754–762.
11. J. T. Roland and Z. Guan, *J. Am. Chem. Soc.*, 2004, **126**, 14328–14329.
12. C. A. Hurley, J. B. Wong, J. Ho, M. Writer, S. A. Irvine, M. J. Lawrence, S. L. Hart, A. B. Tabor and H. C. Hailes, *Org. Biomol. Chem.*, 2008, **6**, 2554–2559.
13. C. Ruzié, P. Even and B. Boitrel, *Org. Biomol. Chem.*, 2007, **5**, 1601–1604.
14. A. Mohammadi, L. Kudsiova, M. F. M. Mustapa, F. Campbell, D. Vlaho, K. Welser, H. Story, A. D. Tagalakis, S. L. Hart, D. J. Barlow, A. B. Tabor, M. J. Lawrence and H. C. Hailes, *Org. Biomol. Chem.*, 2019, **17**, 945–957.

AHH094
user Alex Harkiss
AHH094 Product
PROTON.GLA CDCl3 /u alhark 35

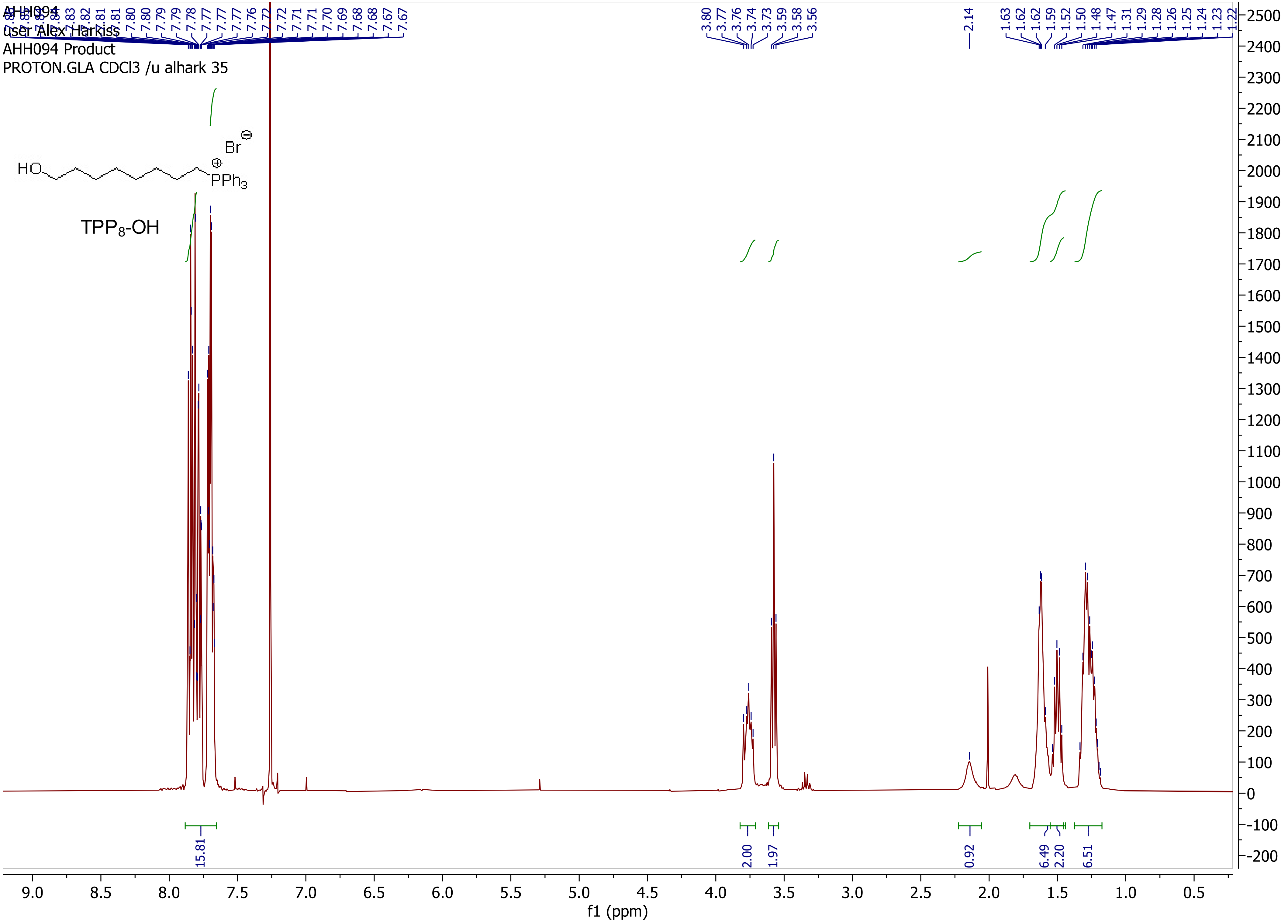

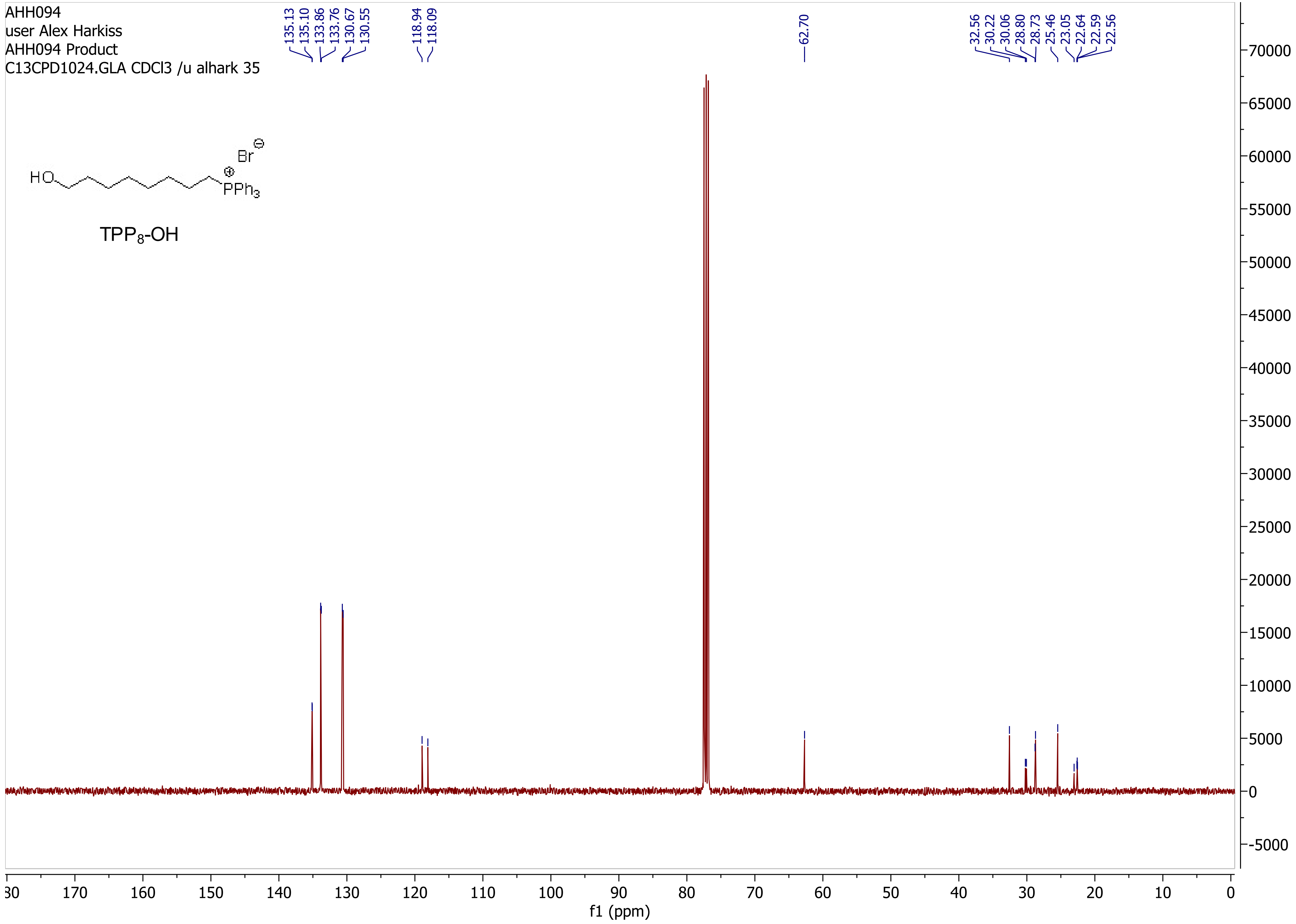
AHH094
user Alex Harkiss
AHH094 Product
C13CPD1024.GLA CDCl3 /u alhark 35
135.13
135.10
133.86
133.76
130.67
130.55
118.94
118.09
62.70
32.56
30.22
30.06
28.80
28.73
25.46
23.05
22.64
22.59
22.56
HO
Br
PPh3
TPP8-OH
f1 (ppm)

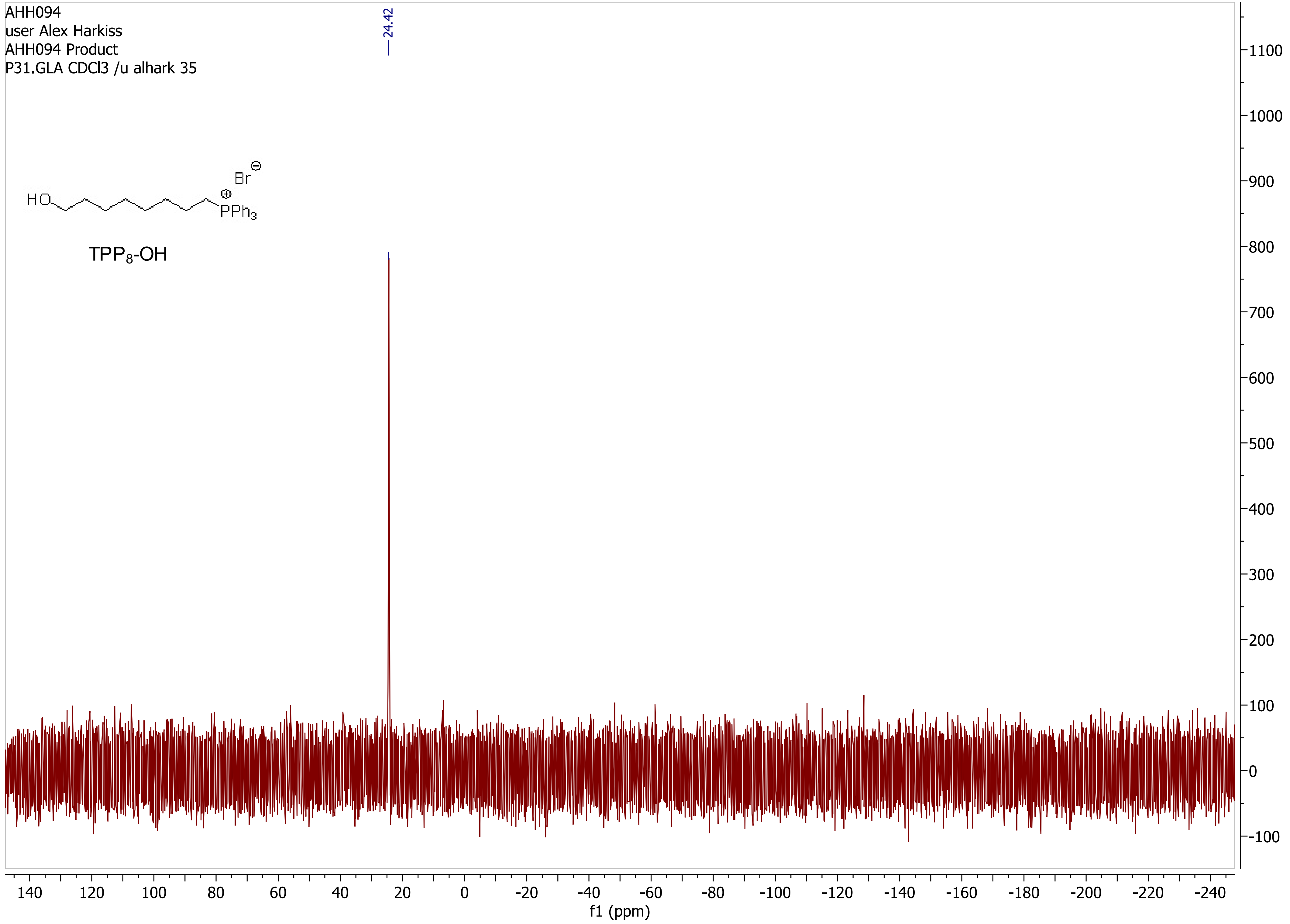
AHH094
user Alex Harkiss
AHH094 Product
P31.GLA CDCl3 /u alhark 35
24.42
HO
Br
PPh3
TPP8-OH
f1 (ppm)

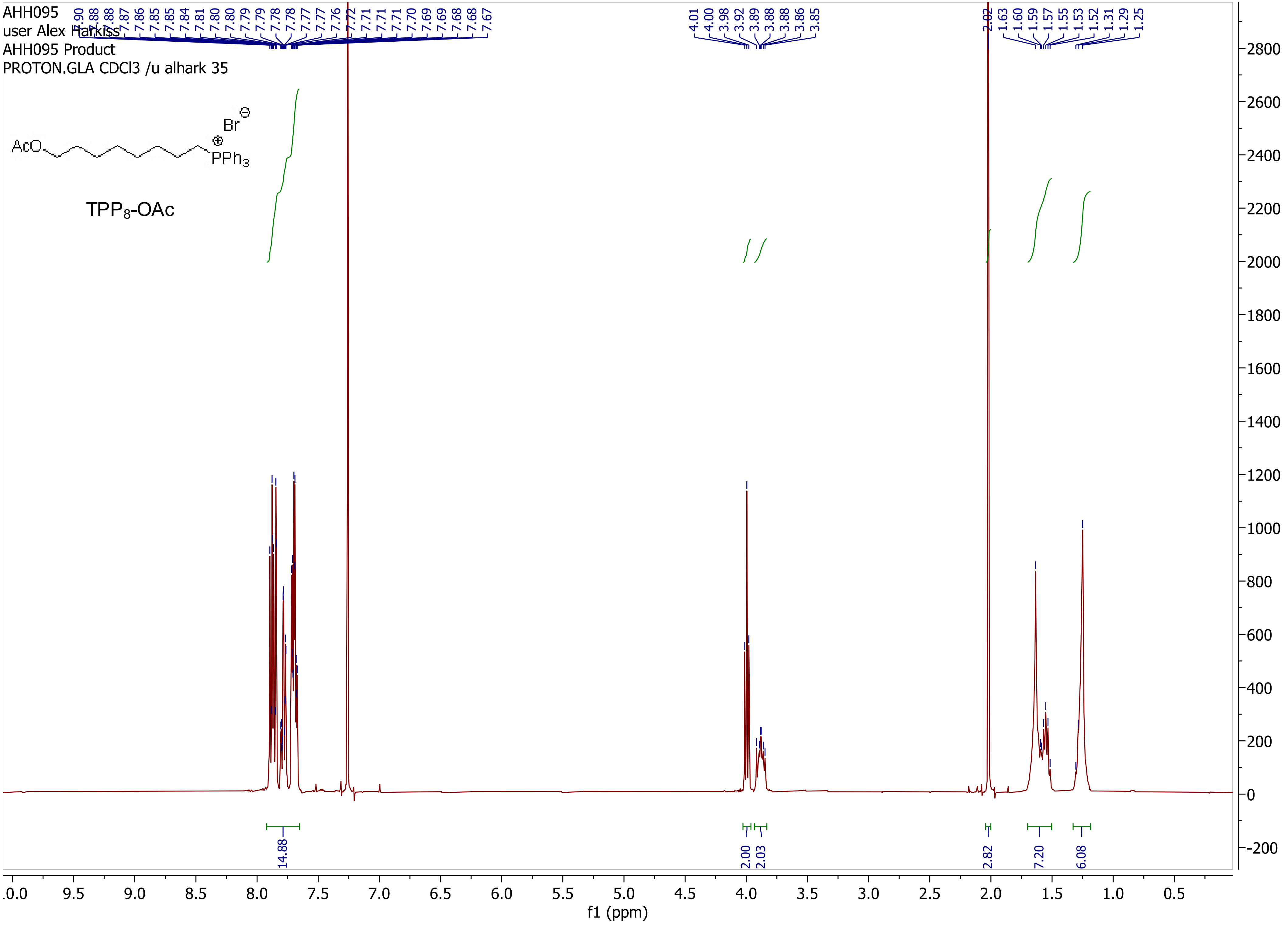
AHH095
user Alex Harkiss
AHH095 Product
PROTON.GLA CDCl3 /u alhark 35
7.90 7.88 7.88 7.87 7.86 7.85 7.85 7.84 7.81 7.80 7.80 7.79 7.79 7.78 7.78 7.77 7.77 7.76 7.72 7.71 7.71 7.71 7.70 7.69 7.69 7.68 7.68 7.67
4.01 4.00 3.98 3.92 3.89 3.88 3.88 3.86 3.85
2.02 1.63 1.60 1.59 1.57 1.55 1.53 1.52 1.31 1.29 1.25
AcO
Br
PPh3
TPP8-OAc
14.88
2.00
2.03
2.82
7.20
6.08
f1 (ppm)

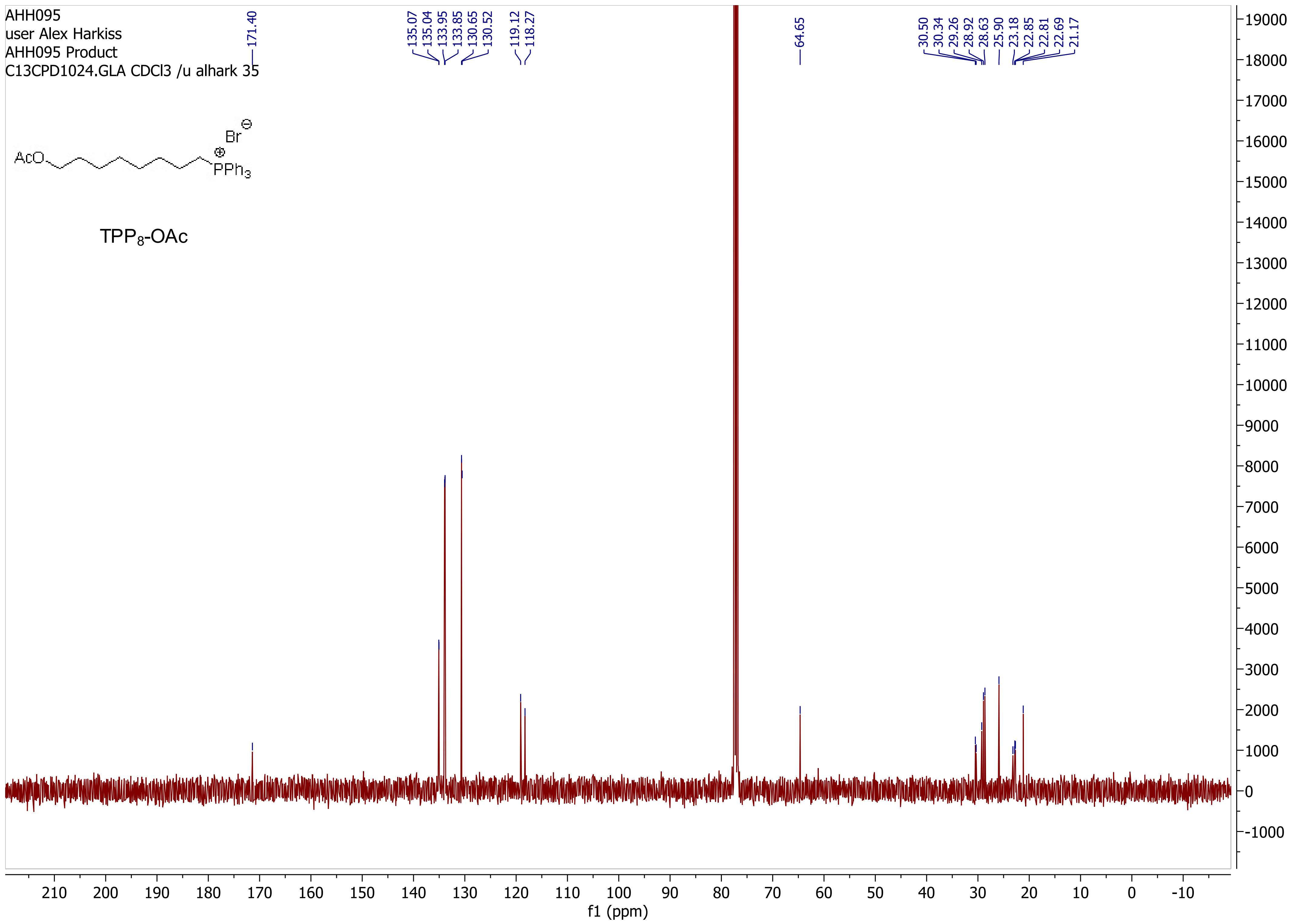

AHH095
user Alex Harkiss
AHH095 Product
C13CPD1024.GLA CDCl3 /u alhark 35
171.40
135.07
135.04
133.95
133.85
130.65
130.52
119.12
118.27
64.65
30.50
30.34
29.26
28.92
28.63
25.90
23.18
22.85
22.81
22.69
21.17
AcO
Br
PPh3
TPP8-OAc
f1 (ppm)

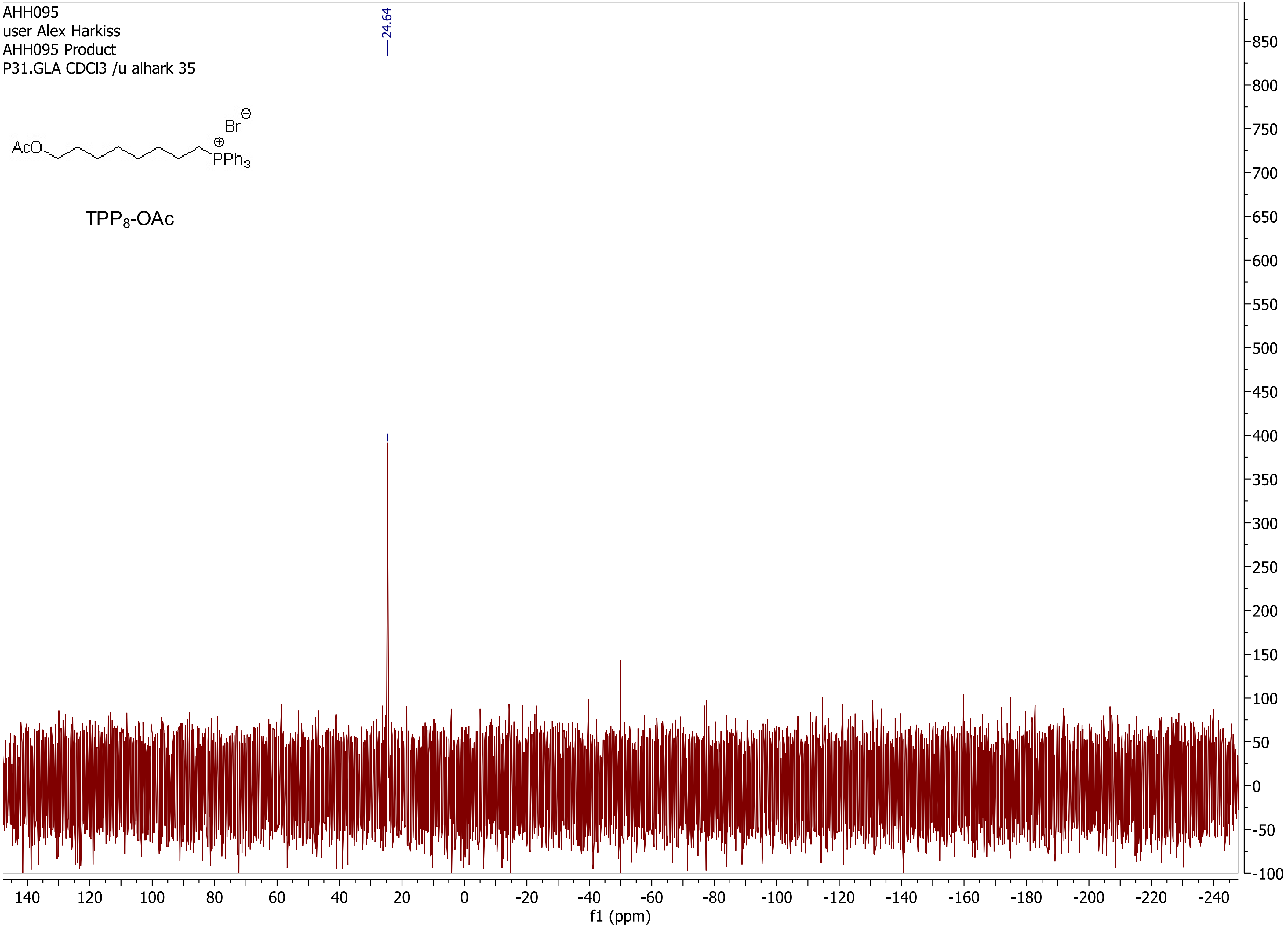
AHH095
user Alex Harkiss
AHH095 Product
P31.GLA CDCl3 /u alhark 35
24.64
Br
PPh3
AcO
TPP8-OAc
f1 (ppm)

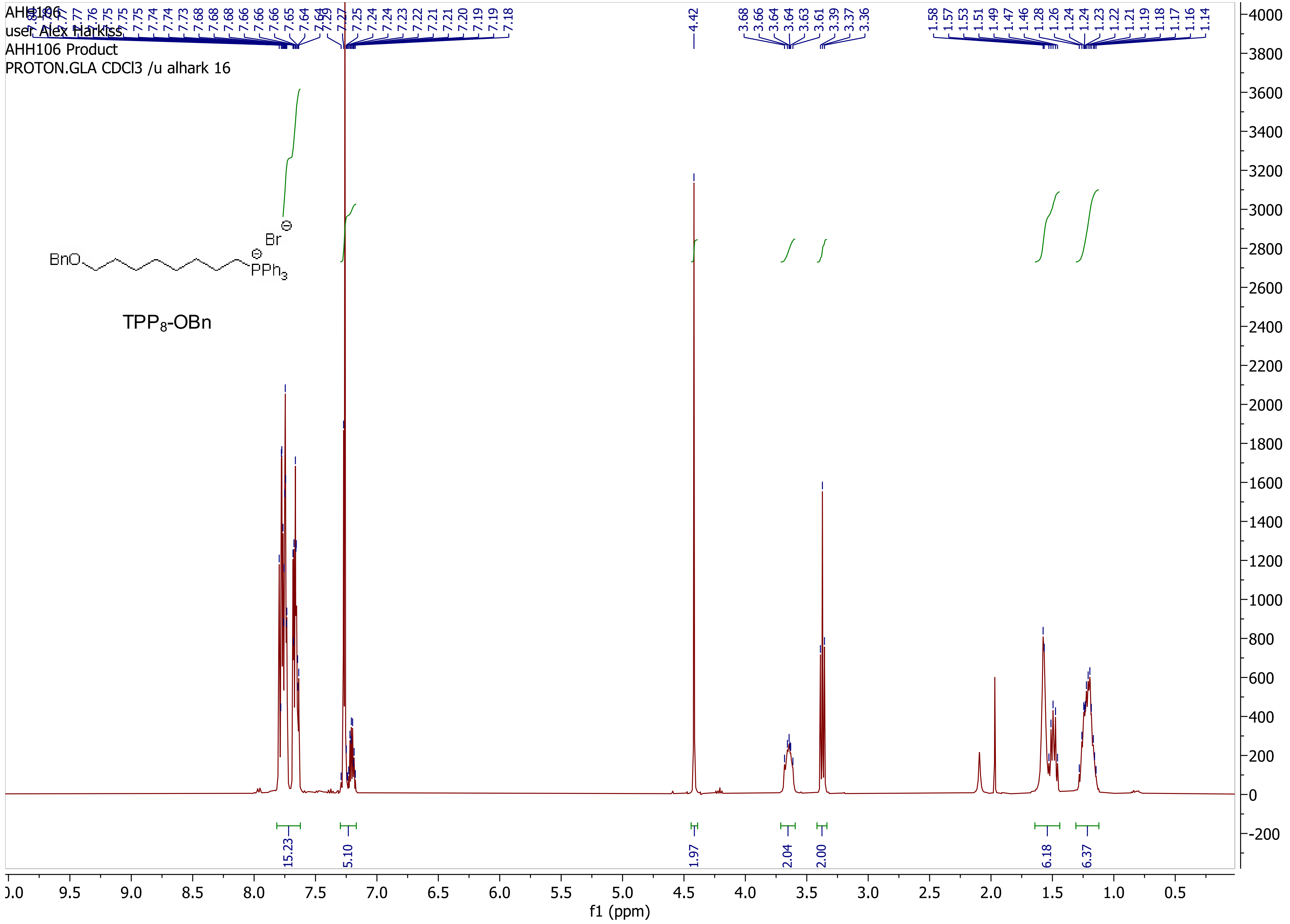
AHH106
user Alex Harkiss
AHH106 Product
PROTON.GLA CDCl3 /u alhark 16
BnO
Br
PPh3
TPP8-OBn
4.42
3.68
3.66
3.64
3.64
3.63
3.61
3.39
3.37
3.36
1.58
1.57
1.53
1.51
1.49
1.47
1.46
1.28
1.26
1.24
1.24
1.23
1.22
1.21
1.19
1.18
1.17
1.16
1.14
15.23
5.10
1.97
2.04
2.00
6.18
6.37
f1 (ppm)

AHH106
user Alex Harkiss
AHH106 Product
C13CPD1024.GLA CDCl3 /u alhark 16

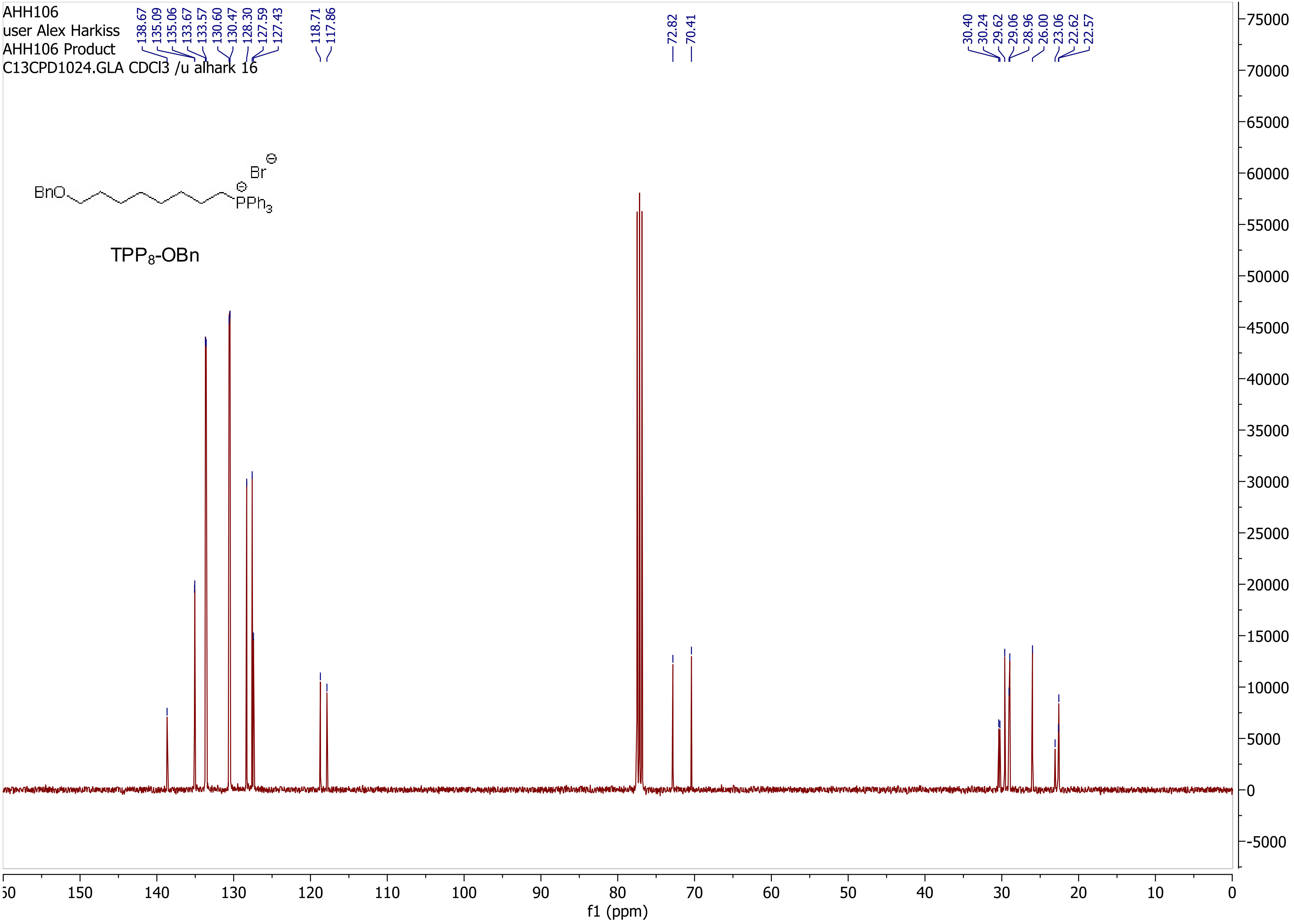

AHH106
user Alex Harkiss
AHH106 Product
P31.GLA CDCl3 /u alhark 16

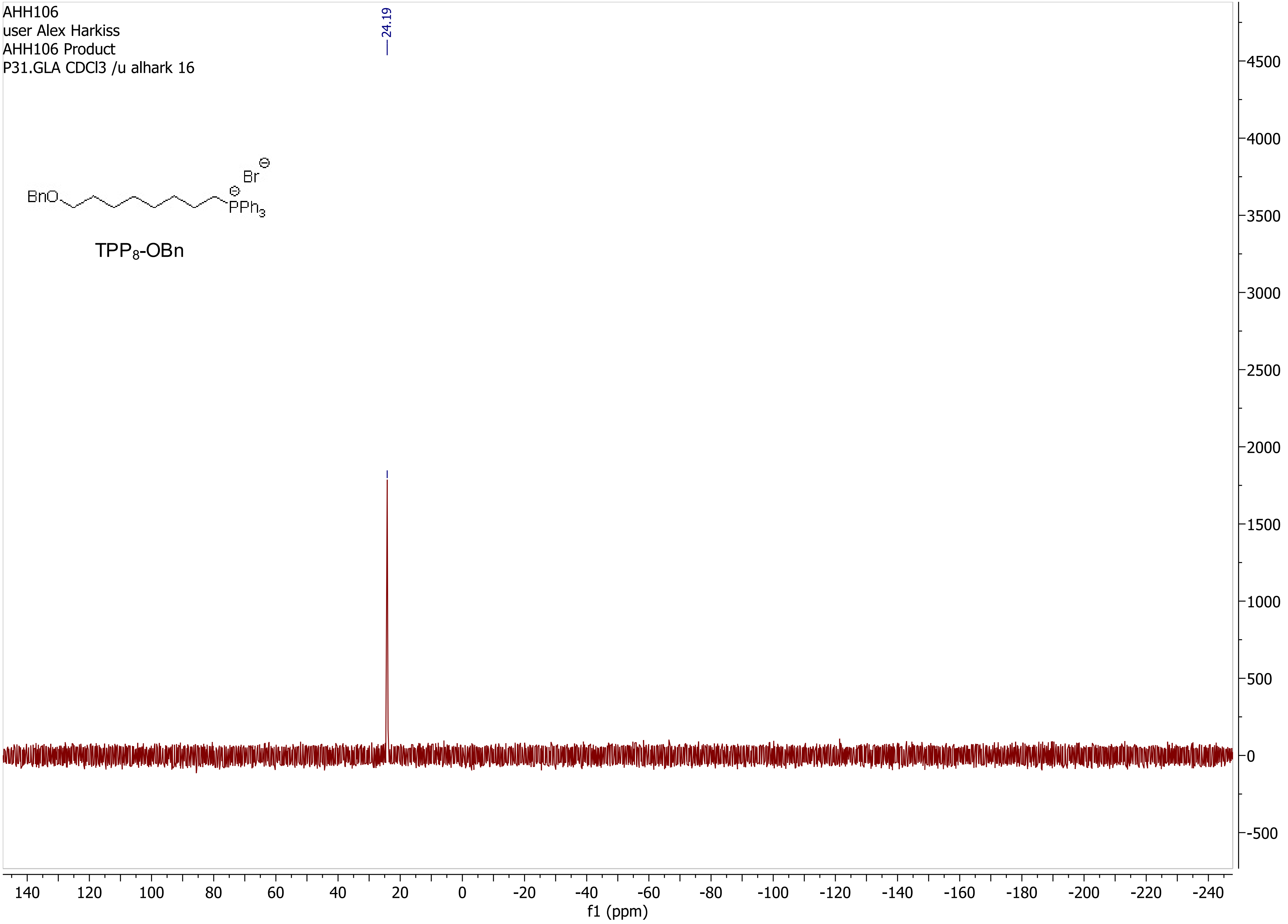

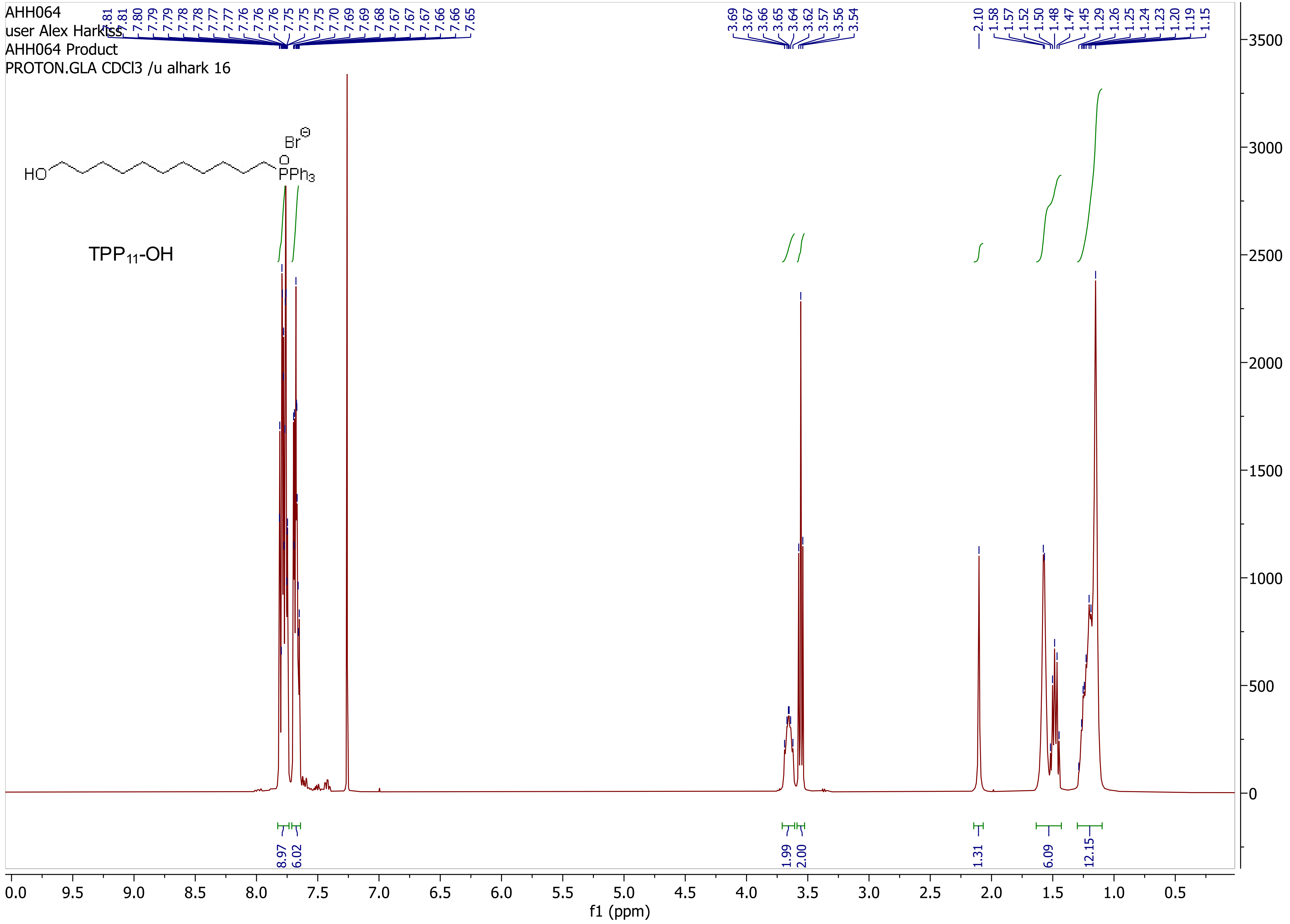

AHH064
user Alex Harkiss
AHH064 Product
PROTON.GLA CDCl3 /u alhark 16
7.81 7.81 7.80 7.79 7.79 7.78 7.78 7.77 7.77 7.76 7.76 7.76 7.75 7.75 7.75 7.70 7.69 7.69 7.68 7.67 7.67 7.67 7.66 7.66 7.65
3.69 3.67 3.66 3.65 3.64 3.62 3.57 3.56 3.54
2.10
1.58 1.57 1.52 1.50 1.48 1.47 1.45 1.29 1.26 1.25 1.24 1.23 1.20 1.19 1.15
Br
HO
PPh3
TPP11-OH
8.97
6.02
1.99
2.00
1.31
6.09
12.15
0.0 9.5 9.0 8.5 8.0 7.5 7.0 6.5 6.0 5.5 5.0 4.5 4.0 3.5 3.0 2.5 2.0 1.5 1.0 0.5
f1 (ppm)
3500 3000 2500 2000 1500 1000 500 0

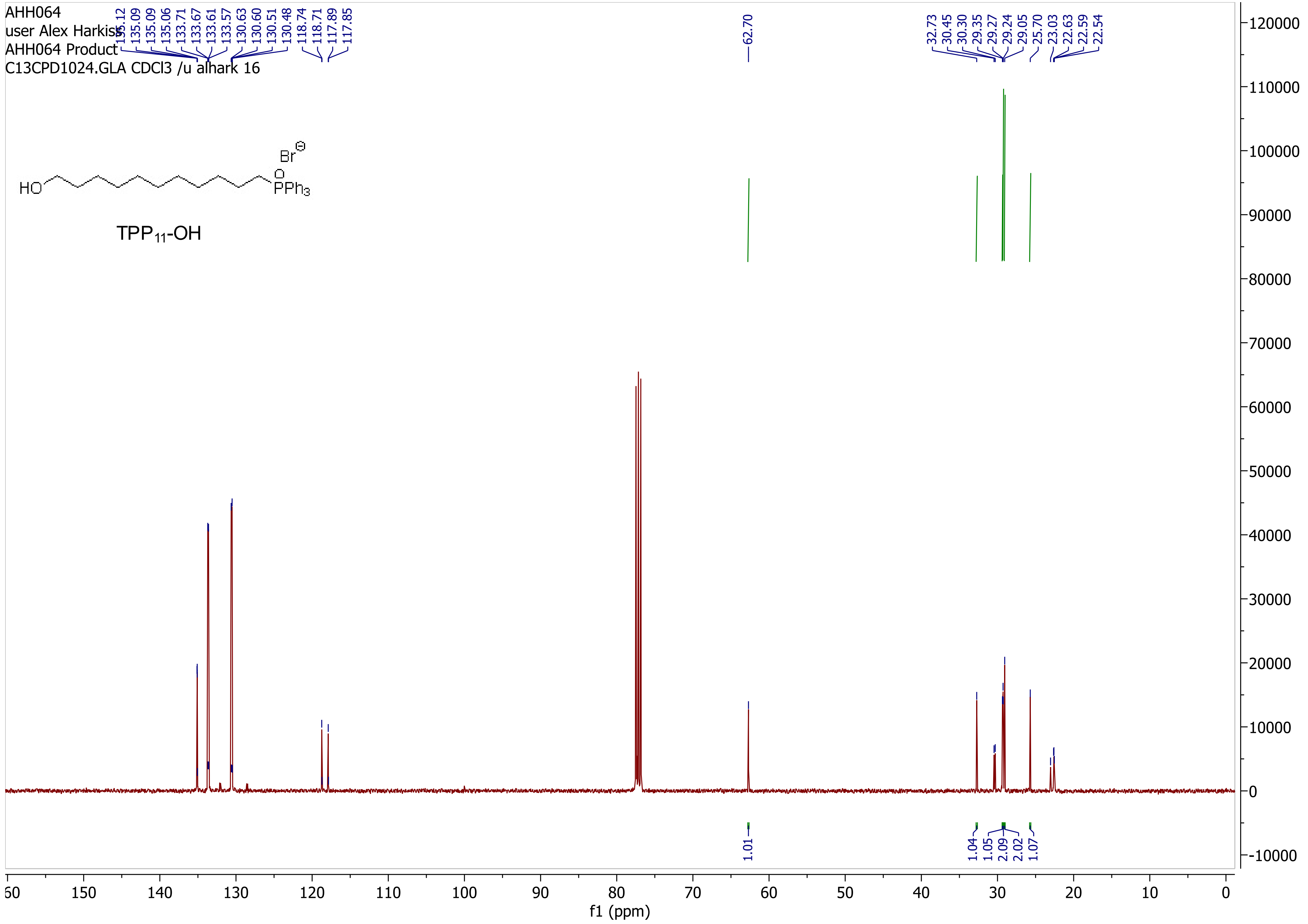
AHH064
user Alex Harkiss
AHH064 Product
C13CPD1024.GLA CDCl3 /u alhark 16
135.12
135.09
135.09
135.06
133.71
133.67
133.61
133.57
130.63
130.60
130.51
130.48
118.74
118.71
117.89
117.85
62.70
32.73
30.45
30.30
29.35
29.27
29.24
29.05
25.70
23.03
22.63
22.59
22.54
HO
Br
PPh3
TPP11-OH
1.01
1.04
1.05
2.09
2.02
1.07
f1 (ppm)

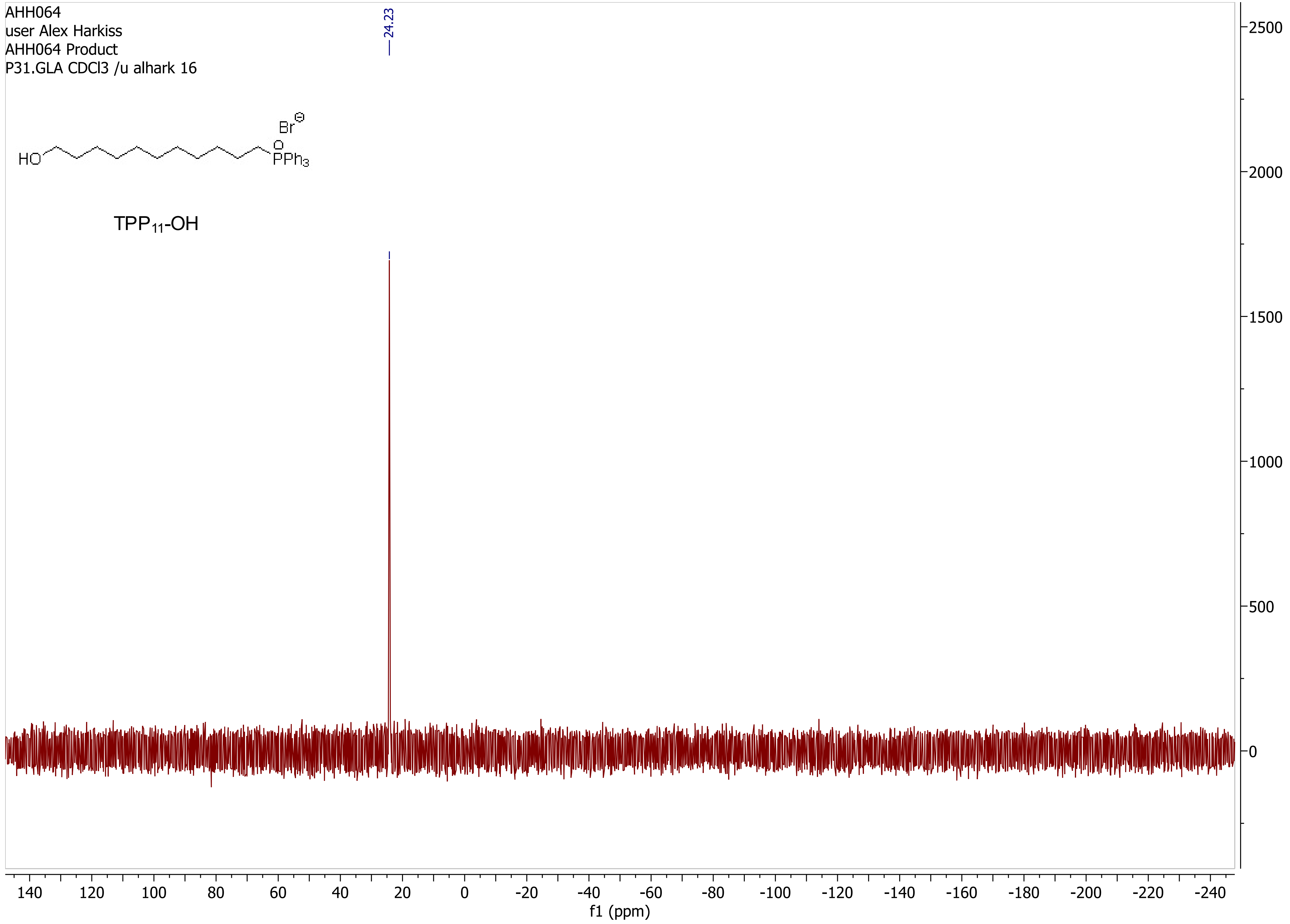
AHH064
user Alex Harkiss
AHH064 Product
P31.GLA CDCl3 /u alhark 16
Br
HO
PPh3
TPP11-OH
24.23
2500
2000
1500
1000
500
0
140 120 100 80 60 40 20 0 -20 -40 -60 -80 -100 -120 -140 -160 -180 -200 -220 -240
f1 (ppm)

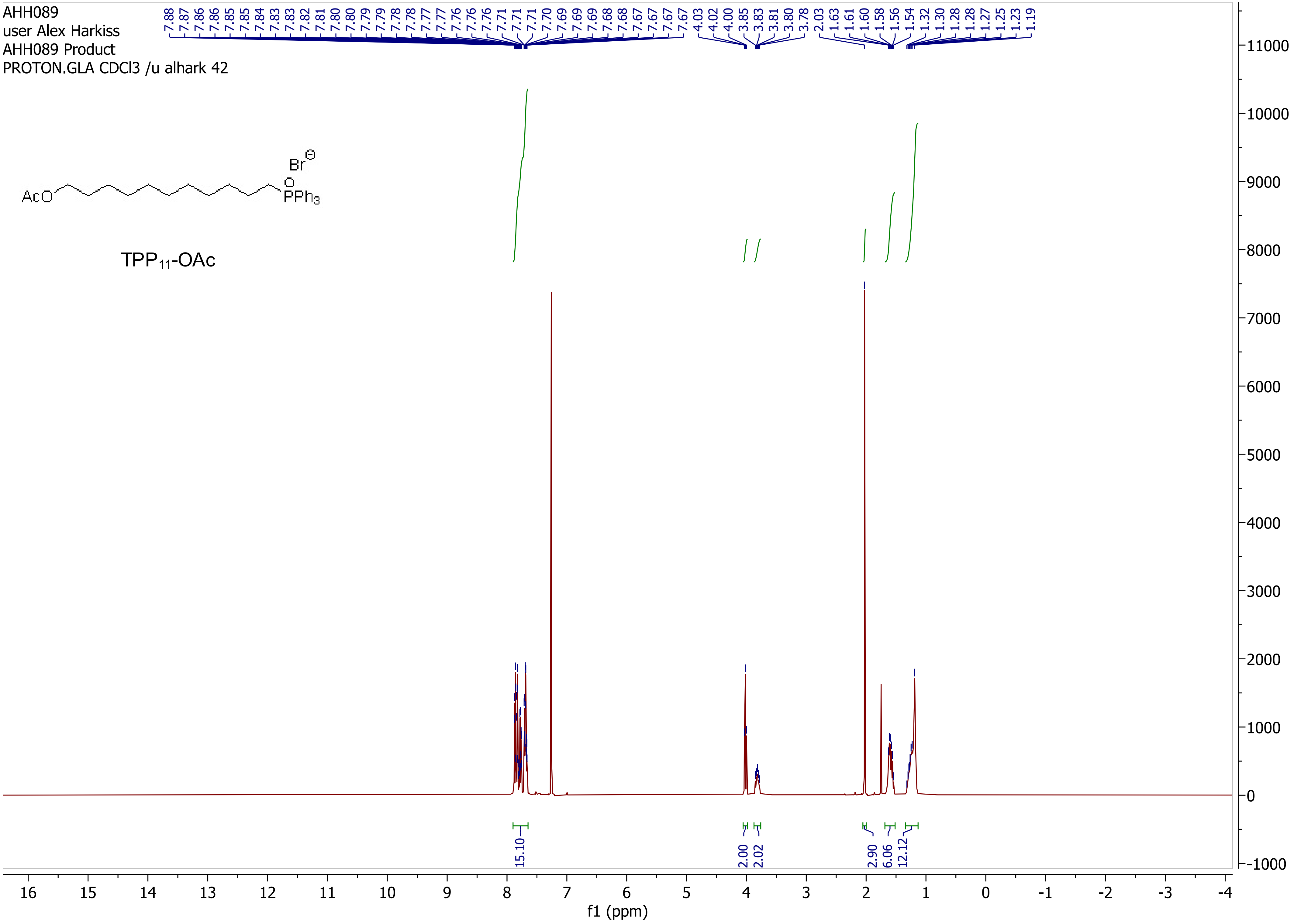
AHH089
user Alex Harkiss
AHH089 Product
PROTON.GLA CDCl3 /u alhark 42
7.88 7.87 7.86 7.86 7.85 7.85 7.84 7.83 7.83 7.82 7.81 7.80 7.80 7.79 7.79 7.78 7.78 7.77 7.77 7.76 7.76 7.76 7.71 7.71 7.71 7.70 7.69 7.69 7.69 7.68 7.68 7.67 7.67 7.67 7.67 4.03 4.02 4.00 3.85 3.83 3.81 3.80 3.78 2.03 1.63 1.61 1.60 1.58 1.56 1.54 1.32 1.30 1.28 1.28 1.27 1.25 1.23 1.19
Br
AcO
O
PPh3
TPP11-OAc
15.10
2.00
2.02
2.90
6.06
12.12
f1 (ppm)

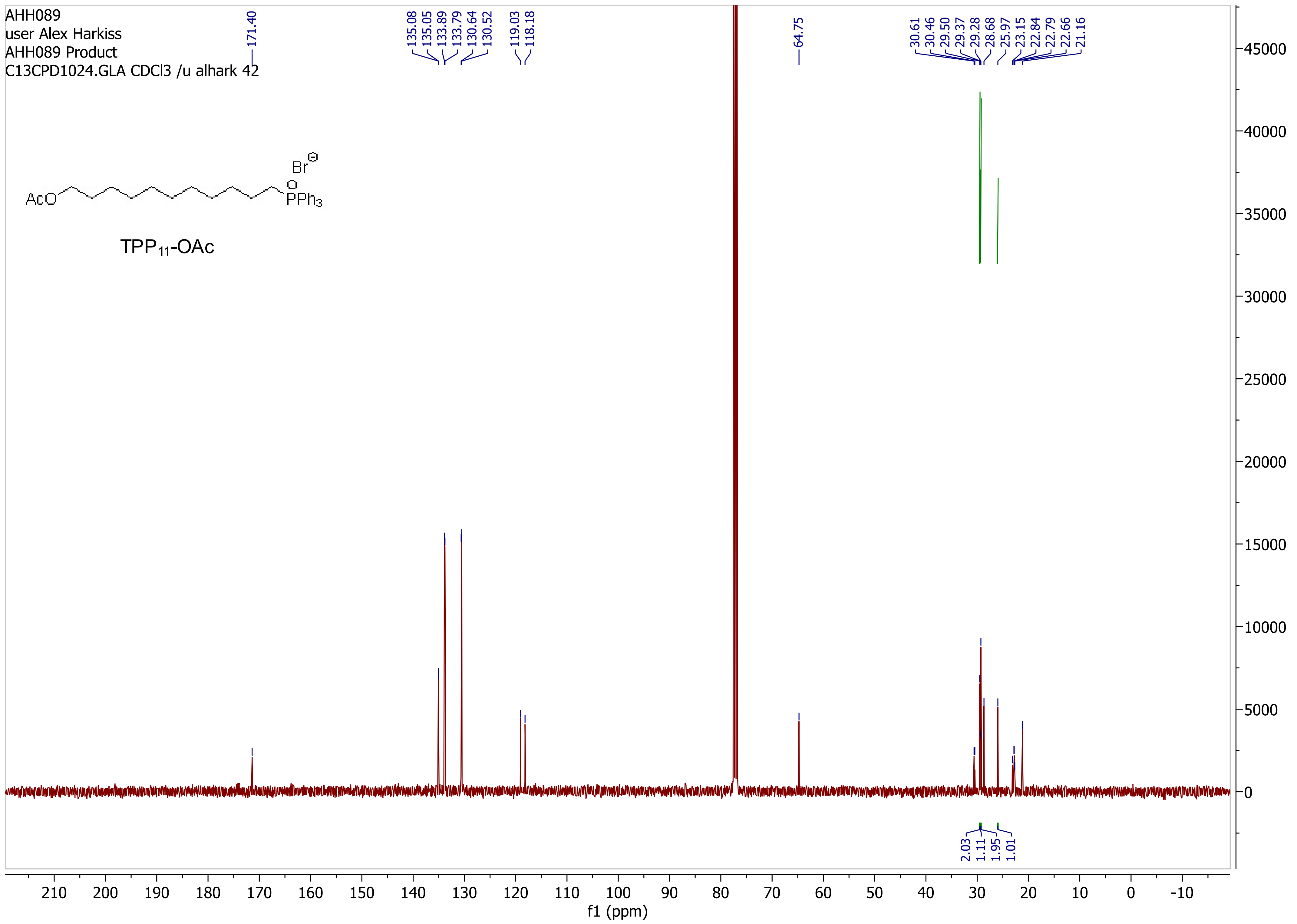
AHH089
user Alex Harkiss
AHH089 Product
C13CPD1024.GLA CDCl3 /u alhark 42
171.40
135.08
135.05
133.89
133.79
130.64
130.52
119.03
118.18
64.75
30.61
30.46
29.50
29.37
29.28
28.68
25.97
23.15
22.84
22.79
22.66
21.16
Br
AcO
O
PPh3
TPP11-OAc
2.03
1.11
1.95
1.01
f1 (ppm)

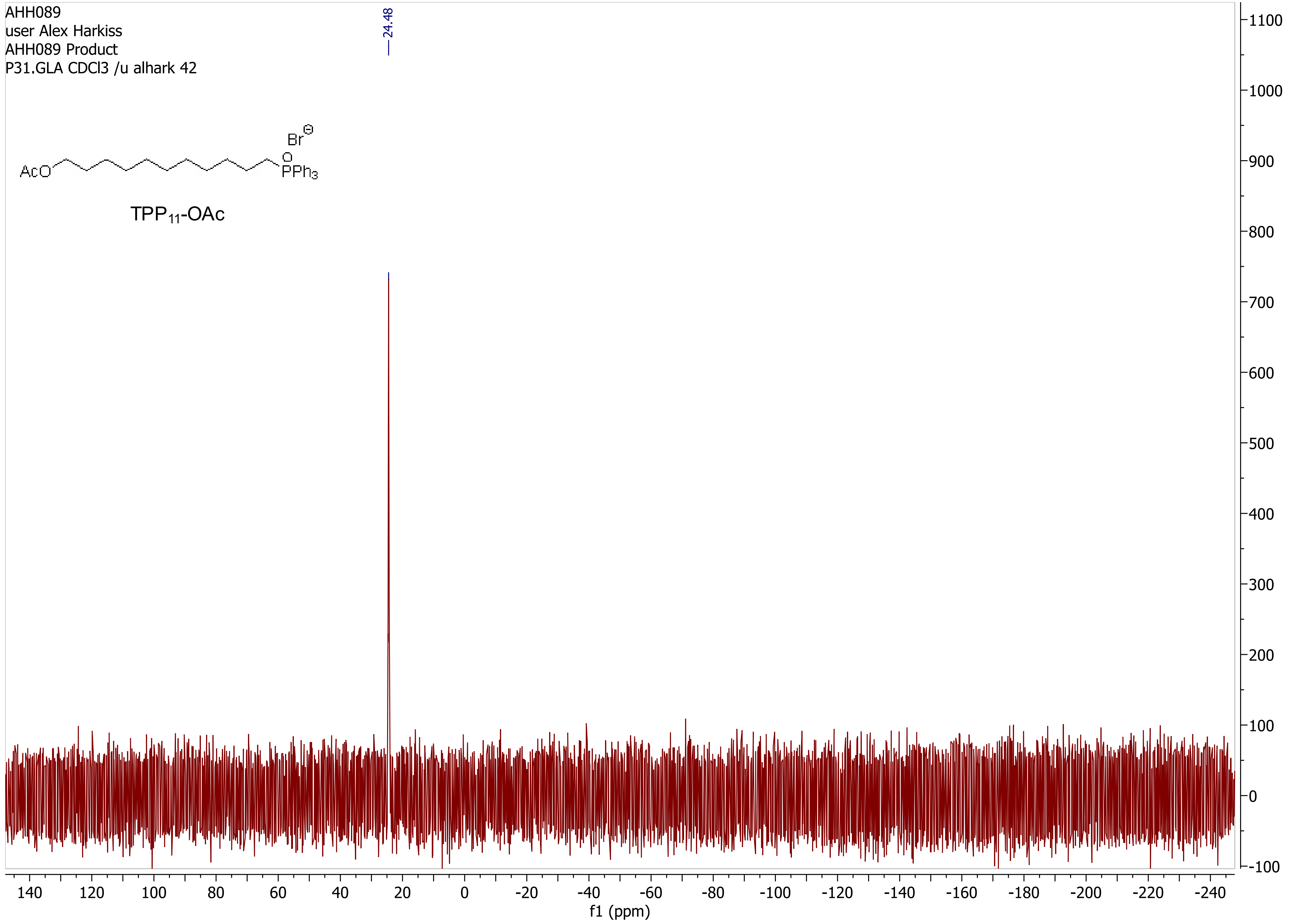
AHH089
user Alex Harkiss
AHH089 Product
P31.GLA CDCl3 /u alhark 42
24.48
AcO
Br
PPh3
TPP11-OAc
f1 (ppm)

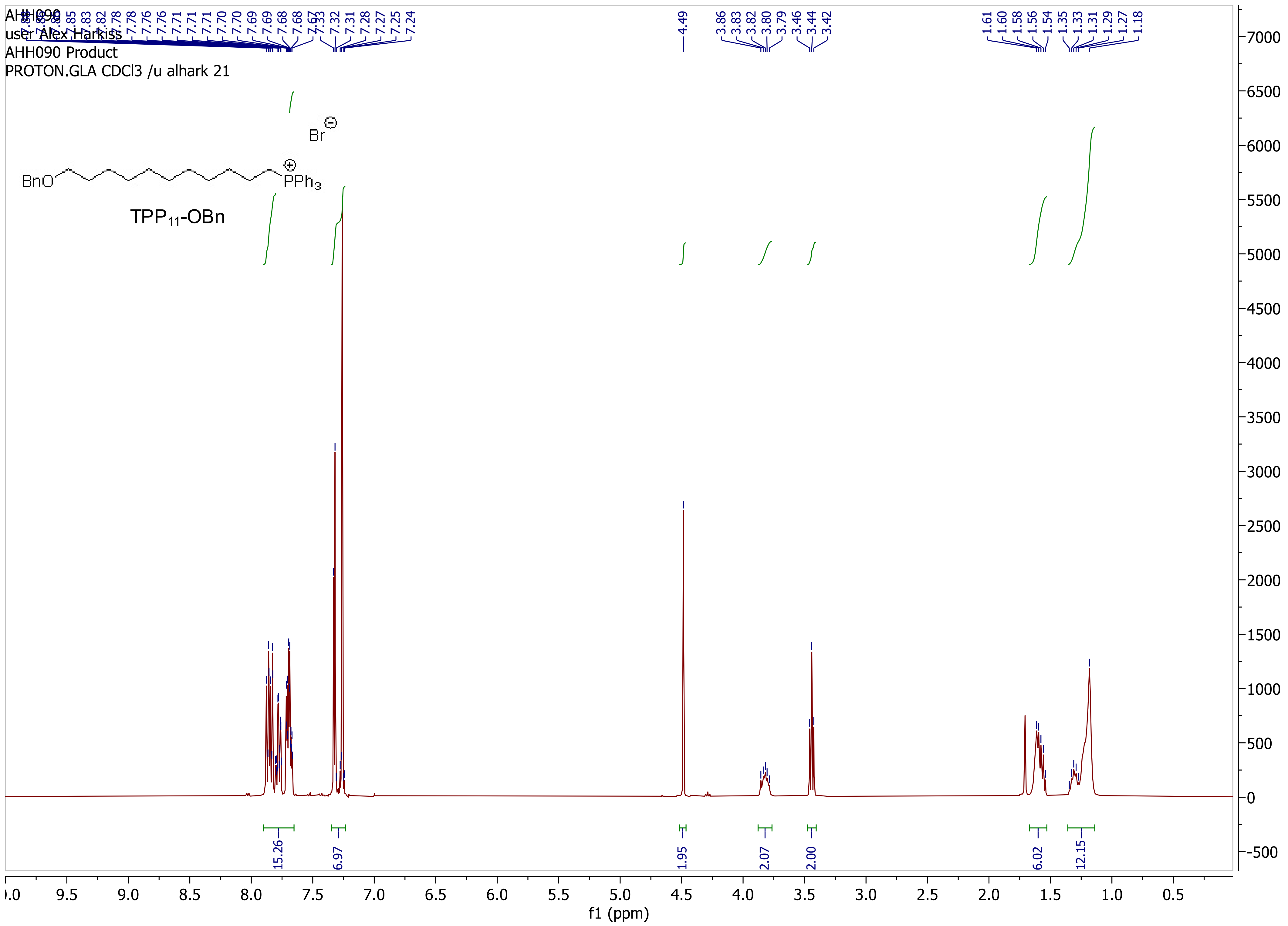
AHH090
user Alex Harkiss
AHH090 Product
PROTON.GLA CDCl3 /u alhark 21
BnO
PPh3
Br
TPP11-OBn
4.49
3.86
3.83
3.82
3.80
3.79
3.46
3.44
3.42
1.61
1.60
1.58
1.56
1.54
1.35
1.33
1.31
1.29
1.27
1.18
15.26
6.97
1.95
2.07
2.00
6.02
12.15
f1 (ppm)

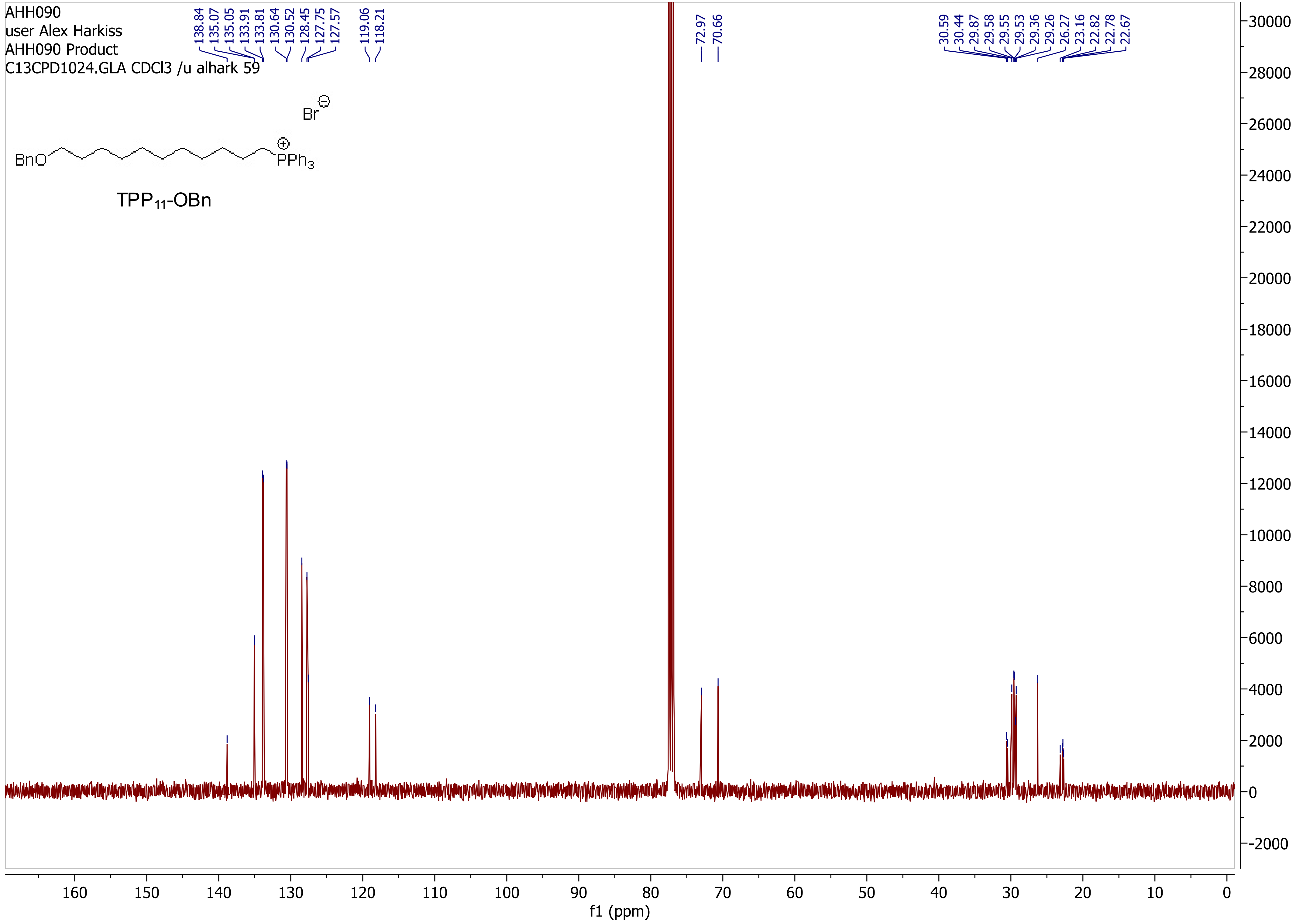
AHH090
user Alex Harkiss
AHH090 Product
C13CPD1024.GLA CDCl3 /u alhark 59
138.84
135.07
135.05
133.91
133.81
130.64
130.52
128.45
127.75
127.57
119.06
118.21
72.97
70.66
30.59
30.44
29.87
29.58
29.55
29.53
29.36
29.26
26.27
23.16
22.82
22.78
22.67
Br
BnO
PPh3
TPP11-OBn
f1 (ppm)

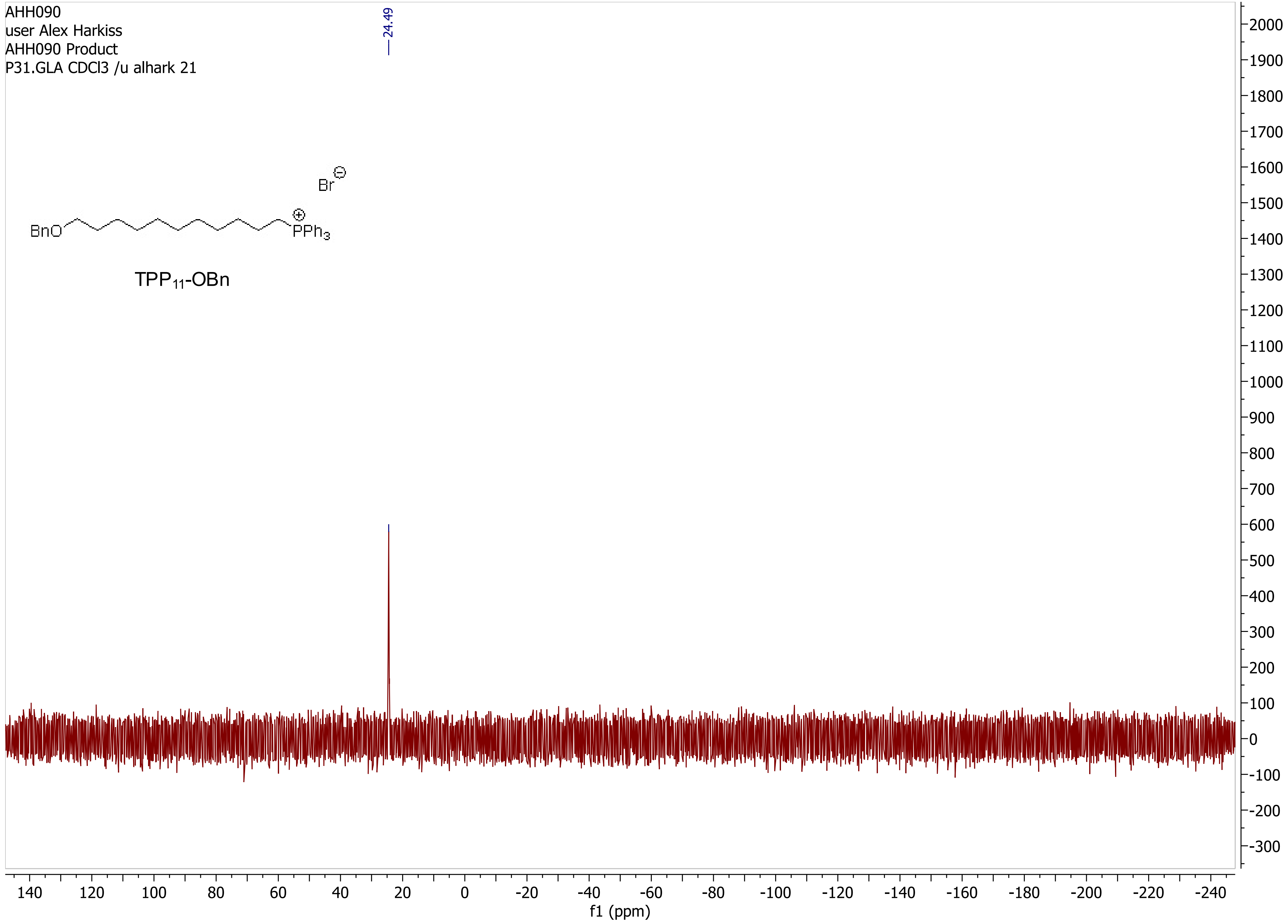
AHH090
user Alex Harkiss
AHH090 Product
P31.GLA CDCl3 /u alhark 21
24.49
BnO
PPh3
Br
TPP11-OBn
f1 (ppm)

AHH104
user Alex Harkiss
AHH104 Product
PROTON.GLA CDCl3 /u alhark 2

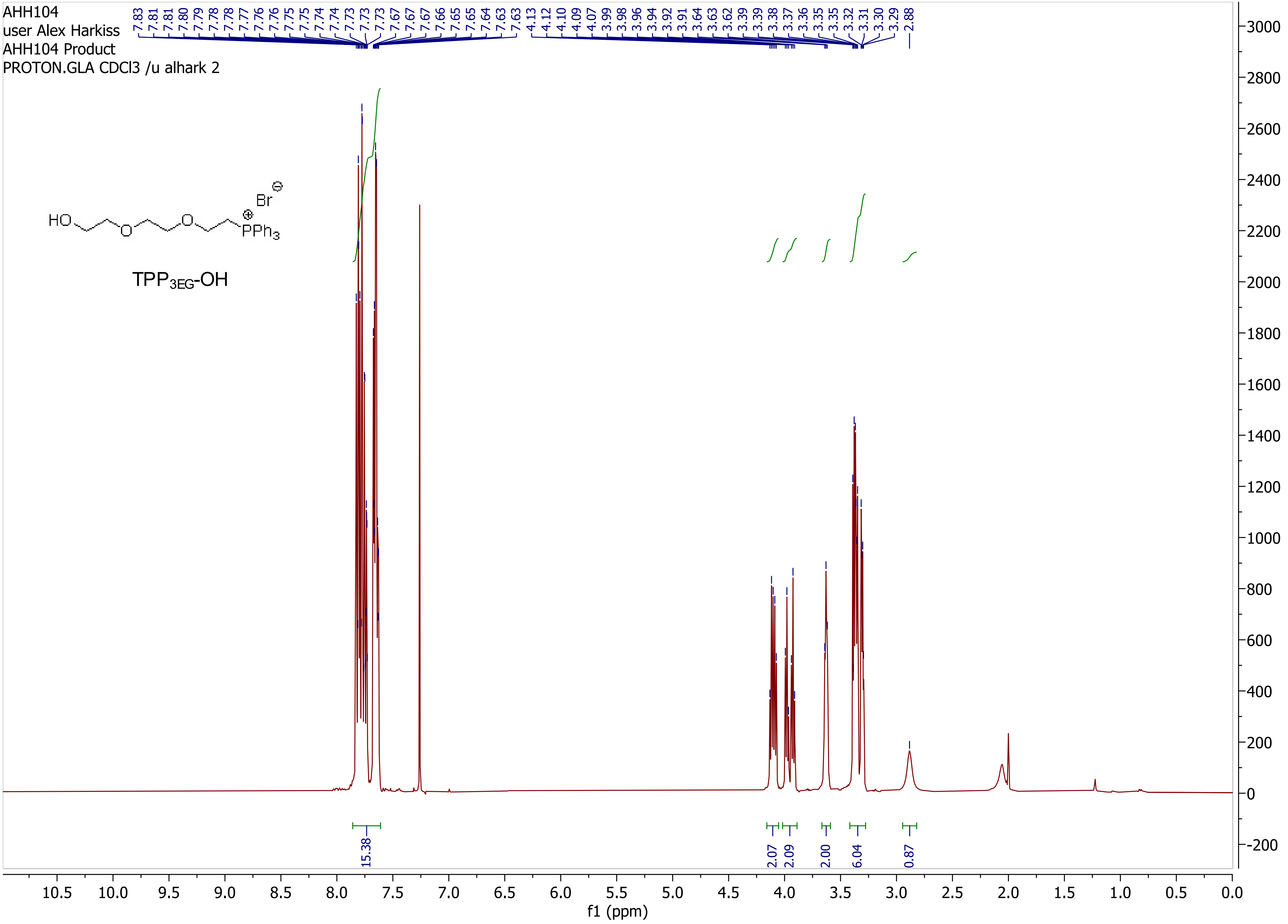

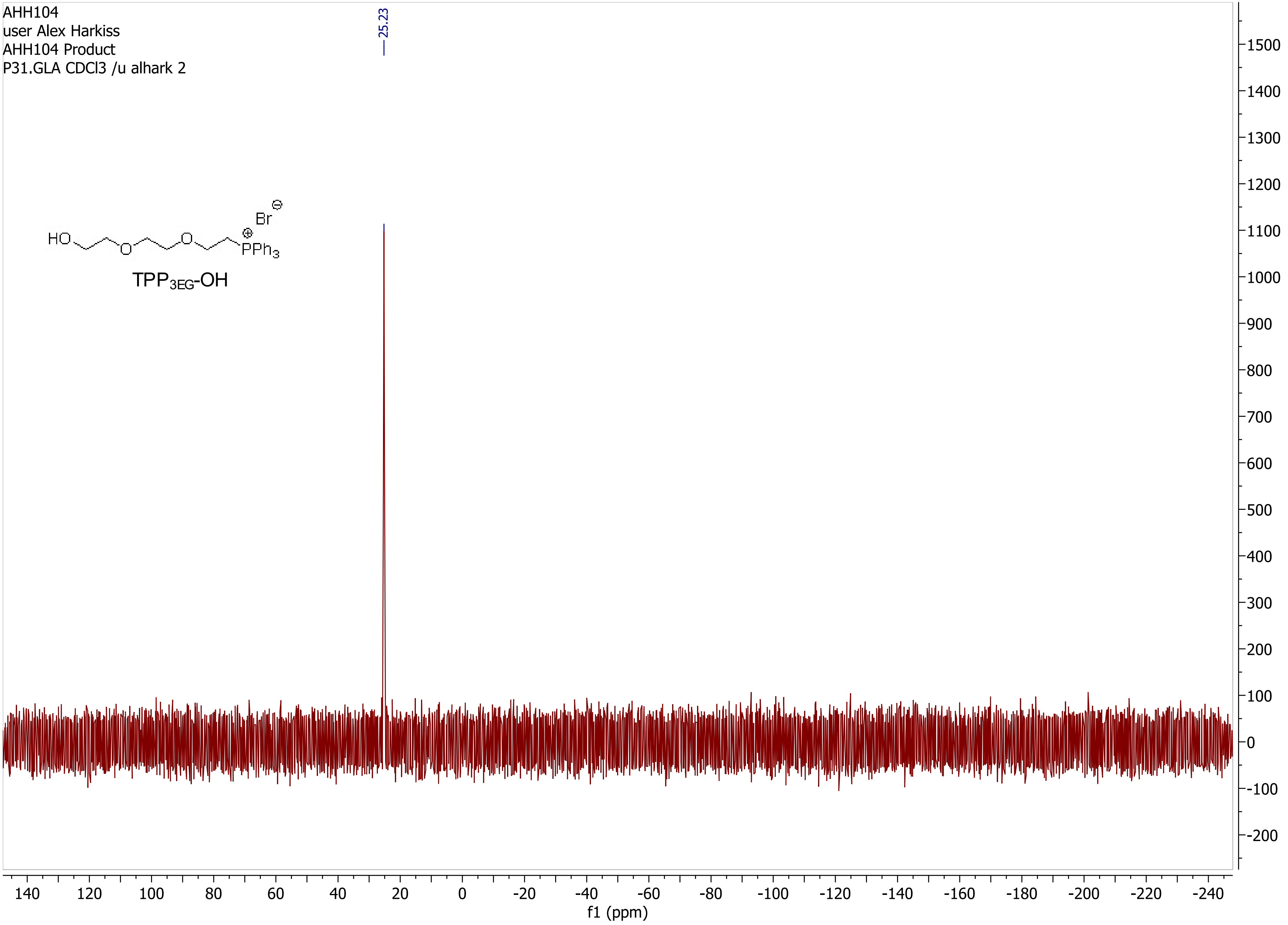
AHH104
user Alex Harkiss
AHH104 Product
P31.GLA CDCl3 /u alhark 2
25.23
HO
O
O
Br
PPh3
TPP3EG-OH
1500
1400
1300
1200
1100
1000
900
800
700
600
500
400
300
200
100
0
-100
-200
140
120
100
80
60
40
20
0
-20
-40
-60
-80
-100
-120
-140
-160
-180
-200
-220
-240
f1 (ppm)

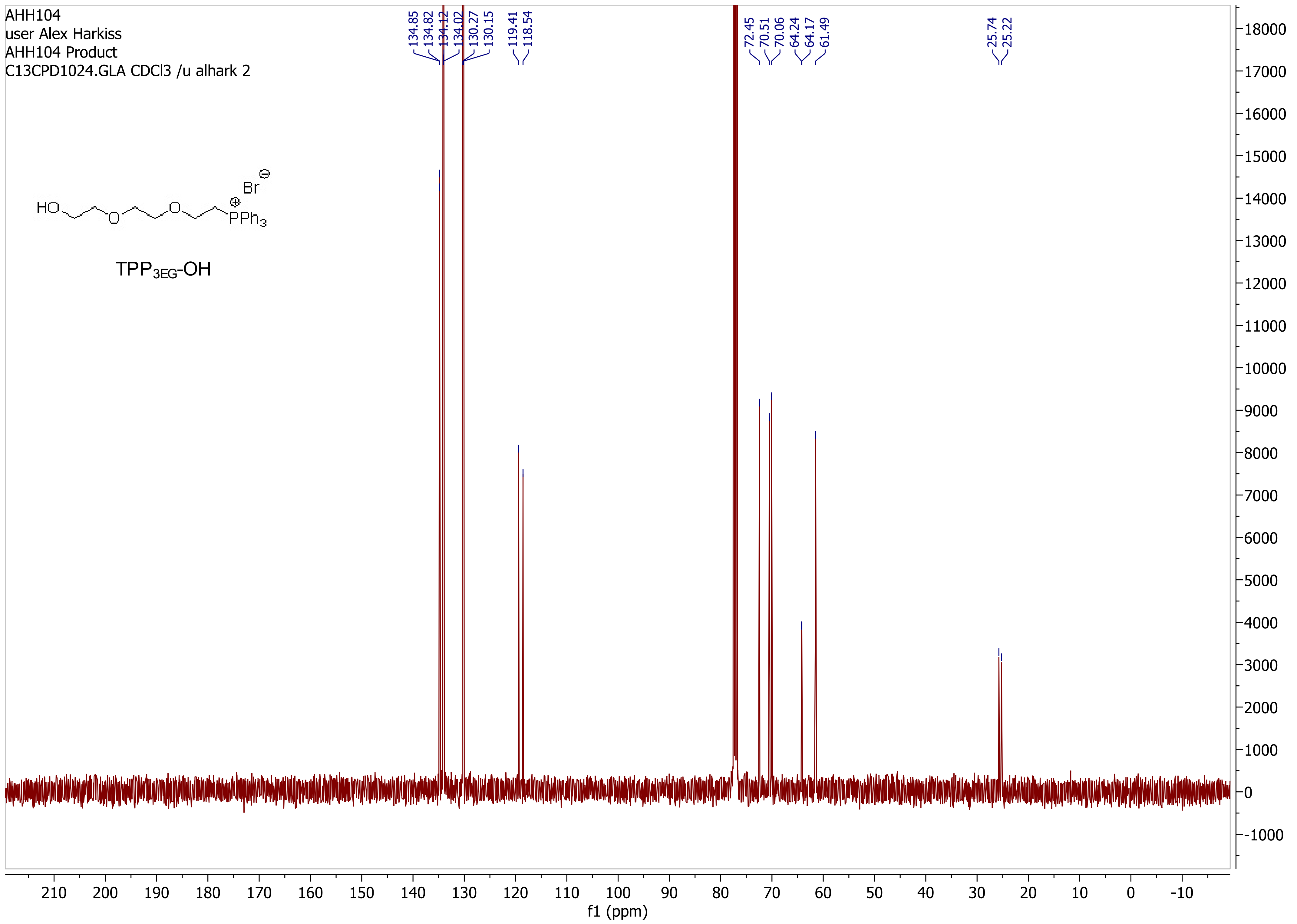
AHH104
user Alex Harkiss
AHH104 Product
C13CPD1024.GLA CDCl3 /u alhark 2
134.85
134.82
134.12
134.02
130.27
130.15
119.41
118.54
72.45
70.51
70.06
64.24
64.17
61.49
25.74
25.22
TPP3EG-OH
f1 (ppm)

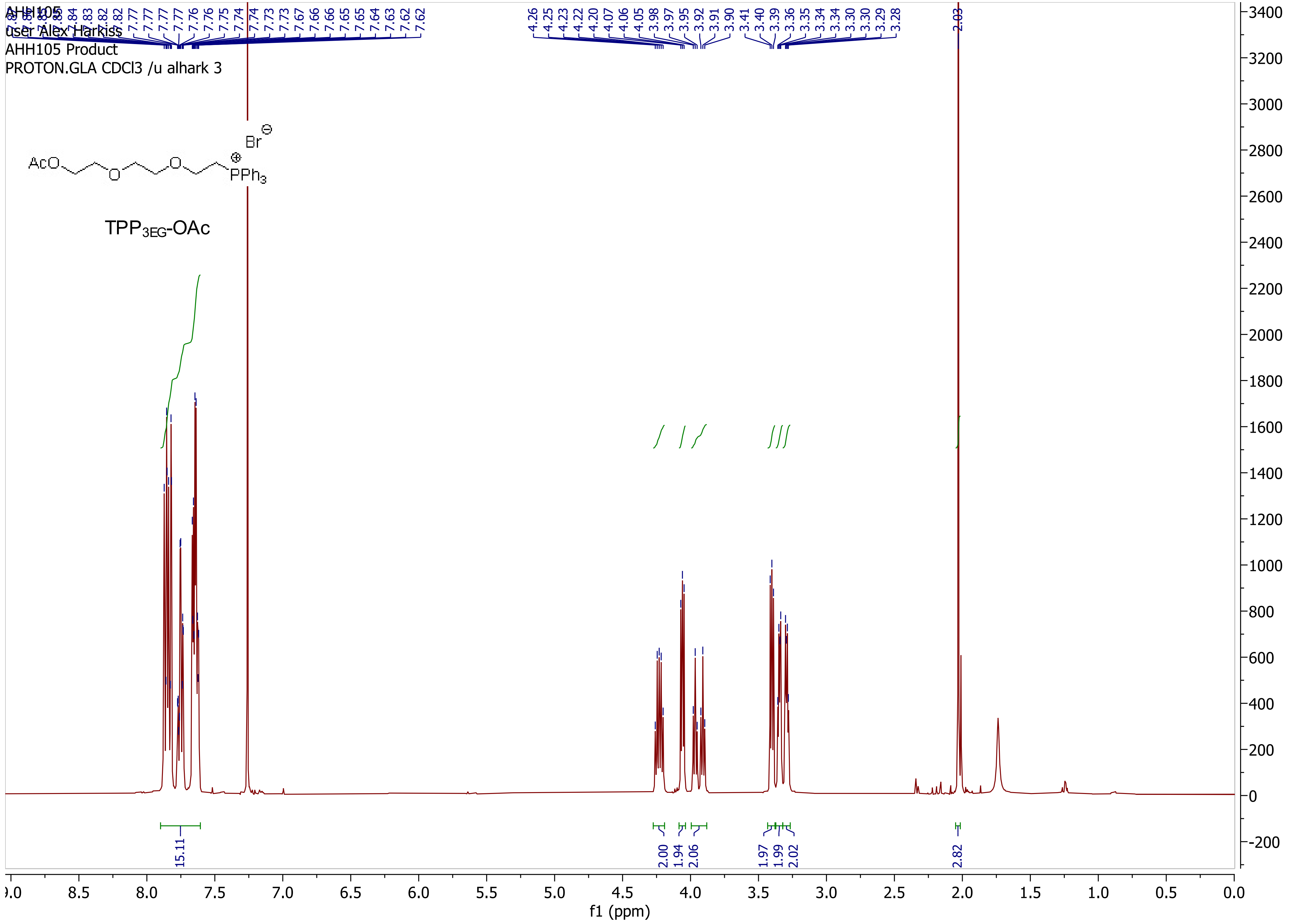

AHH105
user Alex Harkiss
AHH105 Product
PROTON.GLA CDCl3 /u alhark 3
Br
PPh3
AcO
O
O
TPP3EG-OAc
15.11
2.00
1.94
2.06
1.97
1.99
2.02
2.82
f1 (ppm)

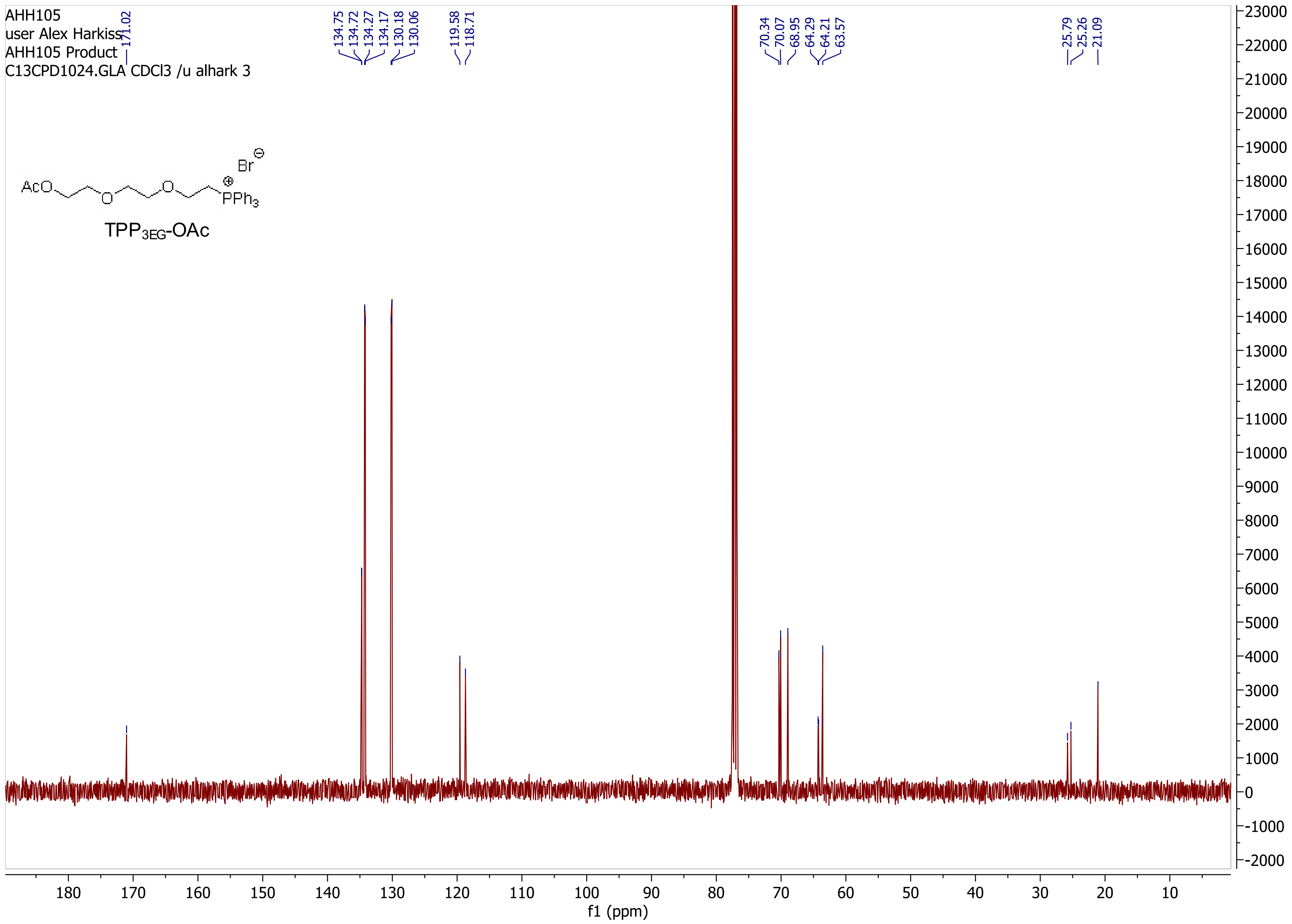

AHH105
user Alex Harkiss
AHH105 Product
C13CPD1024.GLA CDCl3 /u alhark 3
171.02
134.75
134.72
134.27
134.17
130.18
130.06
119.58
118.71
70.34
70.07
68.95
64.29
64.21
63.57
25.79
25.26
21.09
AcO
O
O
Br
PPh3
TPP3EG-OAc
f1 (ppm)

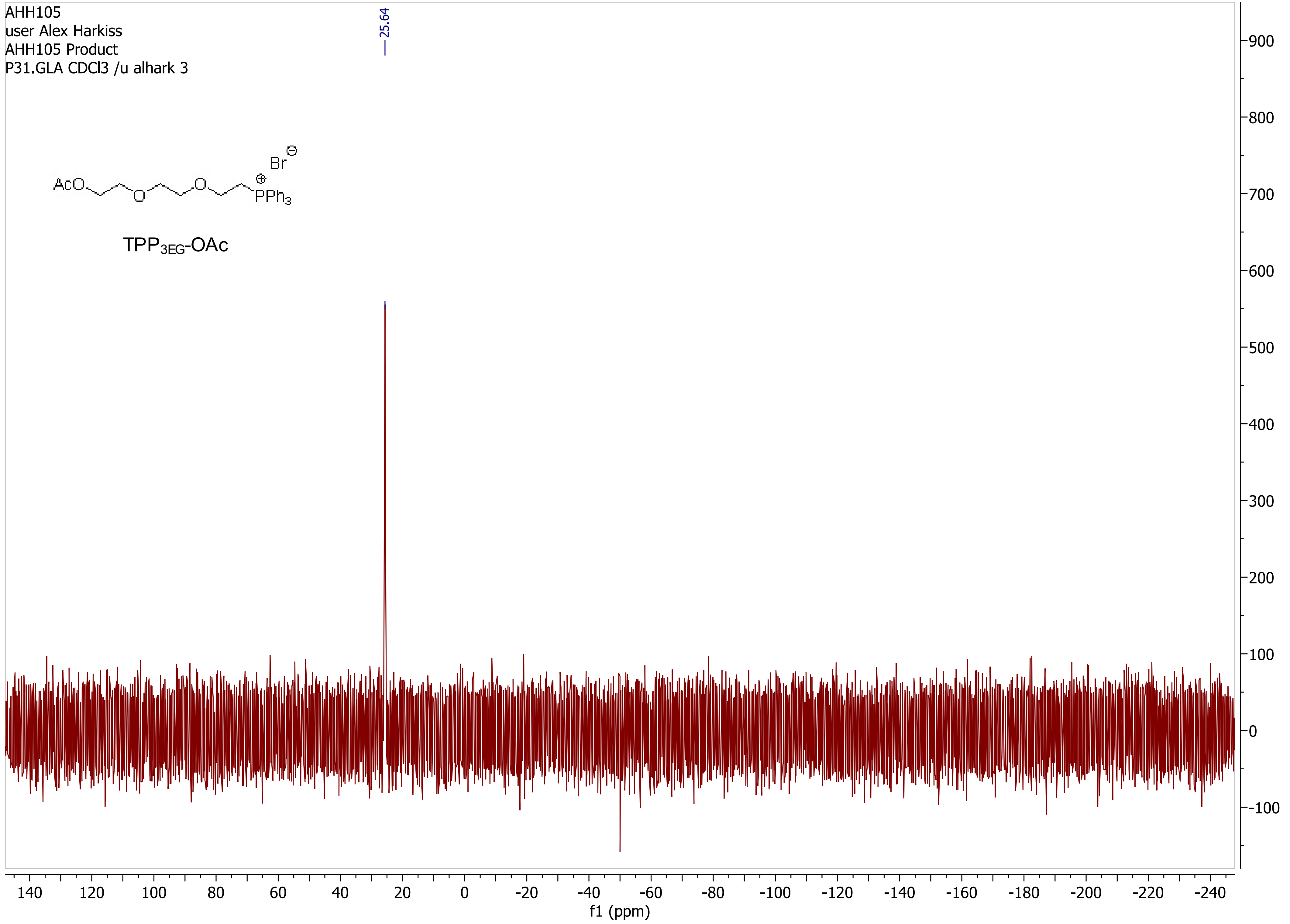
AHH105
user Alex Harkiss
AHH105 Product
P31.GLA CDCl3 /u alhark 3
25.64
AcO
O
O
Br
PPh3
TPP3EG-OAc
900
800
700
600
500
400
300
200
100
0
-100
140
120
100
80
60
40
20
0
-20
-40
-60
-80
-100
-120
-140
-160
-180
-200
-220
-240
f1 (ppm)

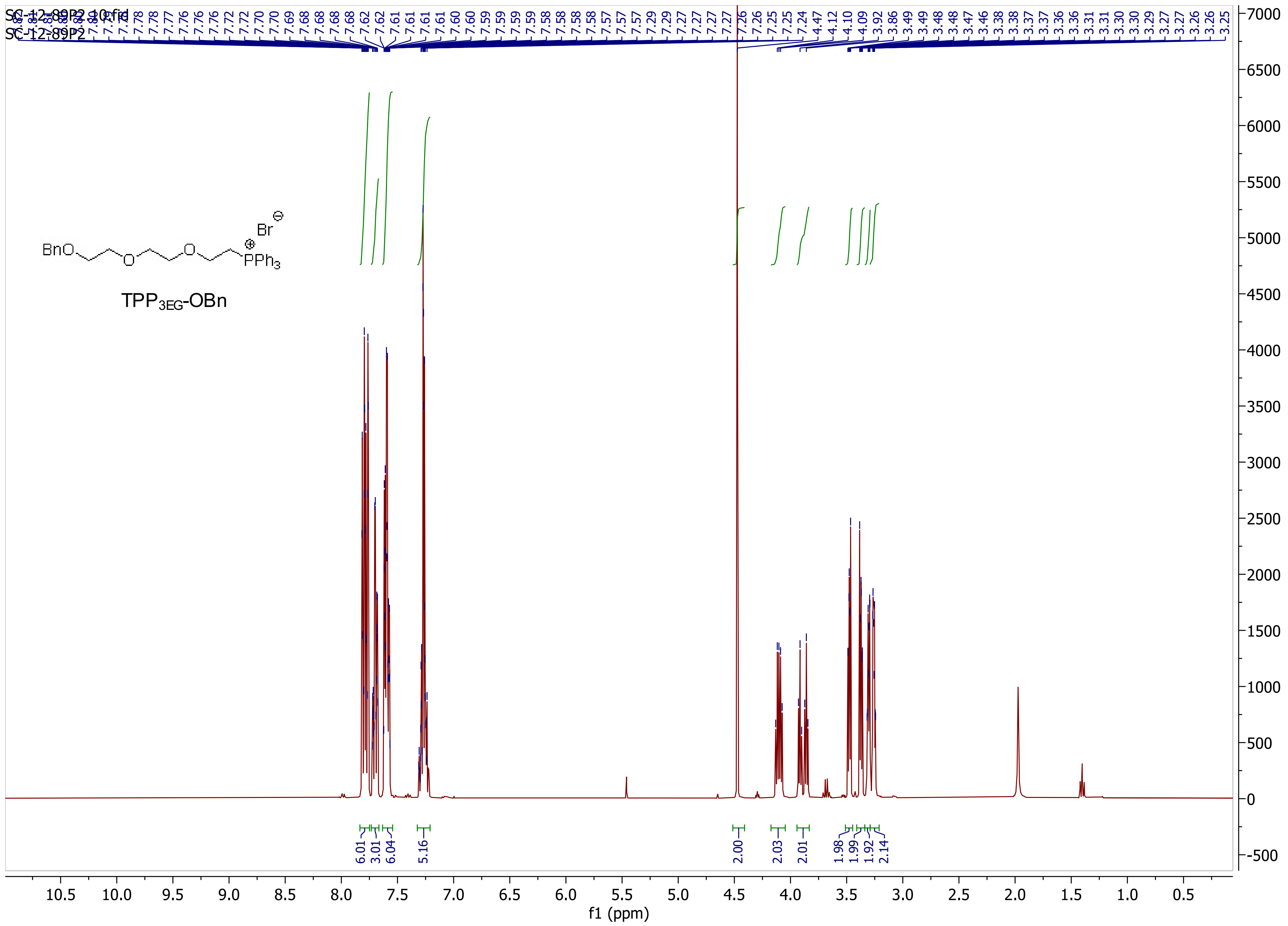

SC-12-89P2.10.fid
SC-12-89P2
7.78 7.78 7.77 7.76 7.76 7.76 7.72 7.72 7.70 7.70 7.69 7.68 7.68 7.68 7.68 7.62 7.62 7.61 7.61 7.61 7.61 7.60 7.60 7.59 7.59 7.59 7.59 7.58 7.58 7.58 7.57 7.57 7.57 7.29 7.29 7.27 7.27 7.27 7.27 7.26 7.26 7.25 7.25 7.24 4.47 4.12 4.10 4.09 3.92 3.86 3.49 3.49 3.48 3.48 3.47 3.46 3.38 3.38 3.37 3.37 3.36 3.36 3.31 3.31 3.30 3.30 3.29 3.27 3.27 3.26 3.26 3.25
BnO
O
O
Br
PPh3
TPP3EG-OBn
6.01
3.01
6.04
5.16
2.00
2.03
2.01
1.98
1.99
1.92
2.14
f1 (ppm)

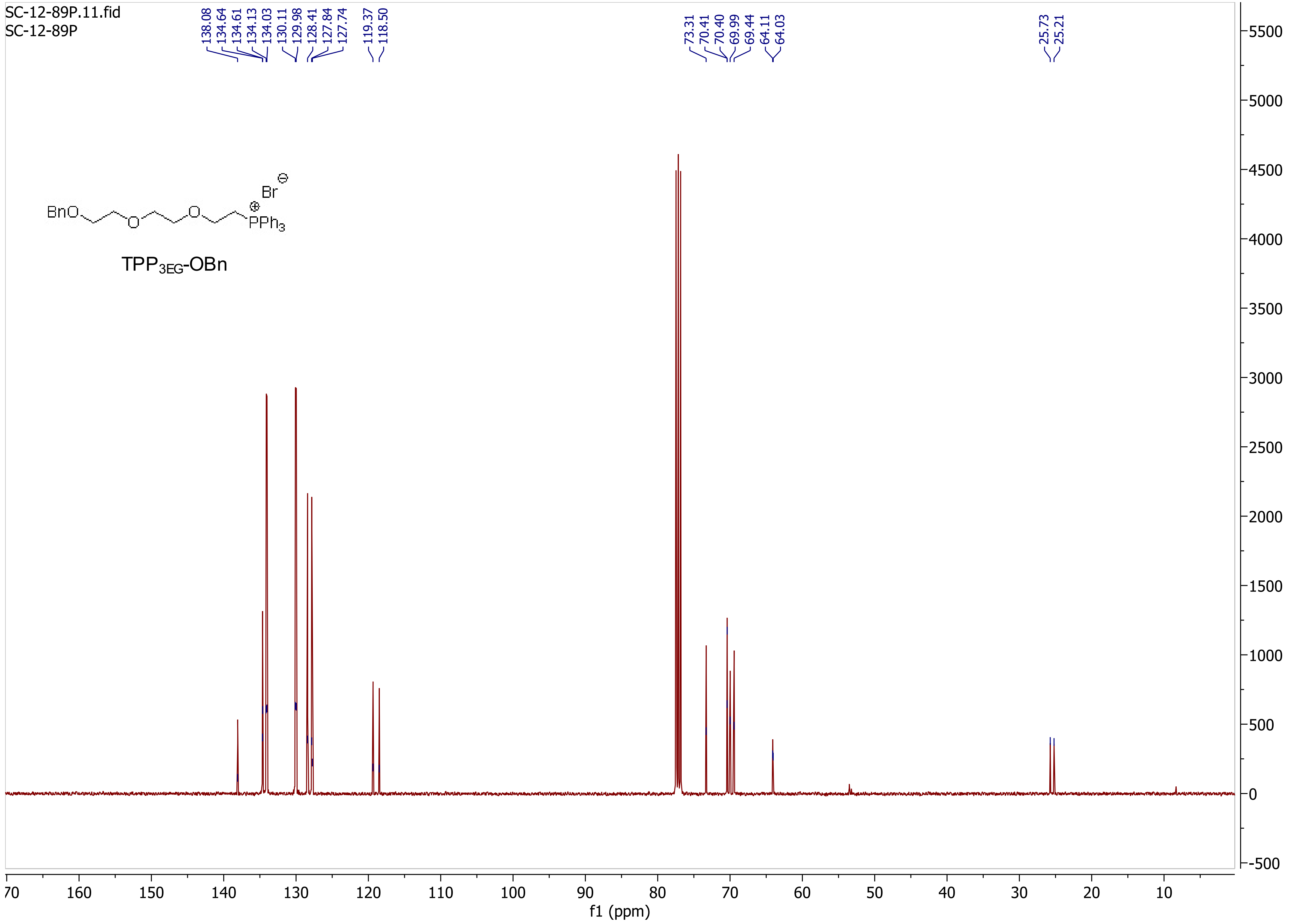
SC-12-89P.11.fid
SC-12-89P
138.08
134.64
134.61
134.13
134.03
130.11
129.98
128.41
127.84
127.74
119.37
118.50
73.31
70.41
70.40
69.99
69.44
64.11
64.03
25.73
25.21
BnO
O
O
PPh3
Br
TPP3EG-OBn
f1 (ppm)

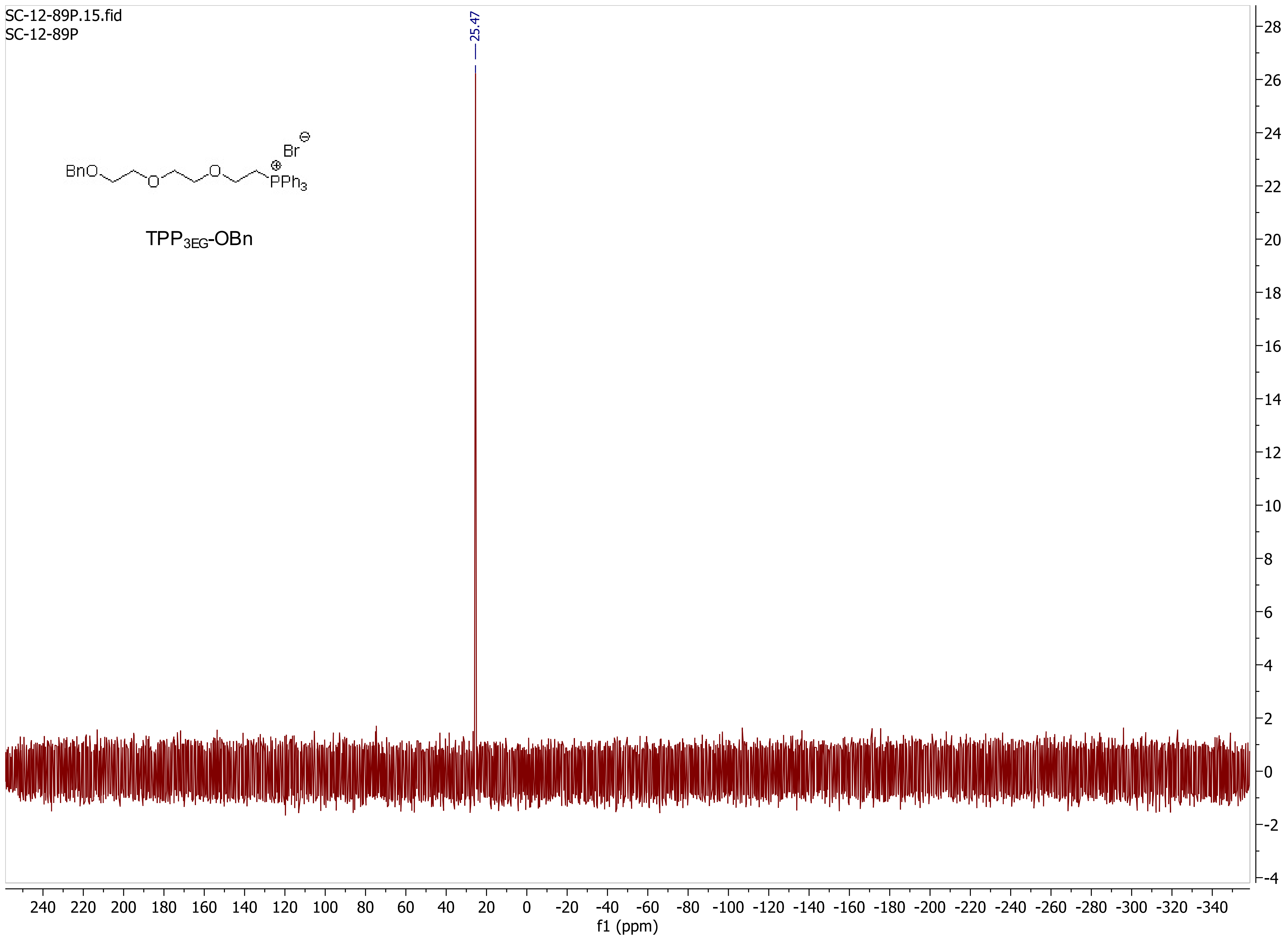
SC-12-89P.15.fid
SC-12-89P
25.47
BnO O O PPh3 Br
TPP3EG-OBn
f1 (ppm)

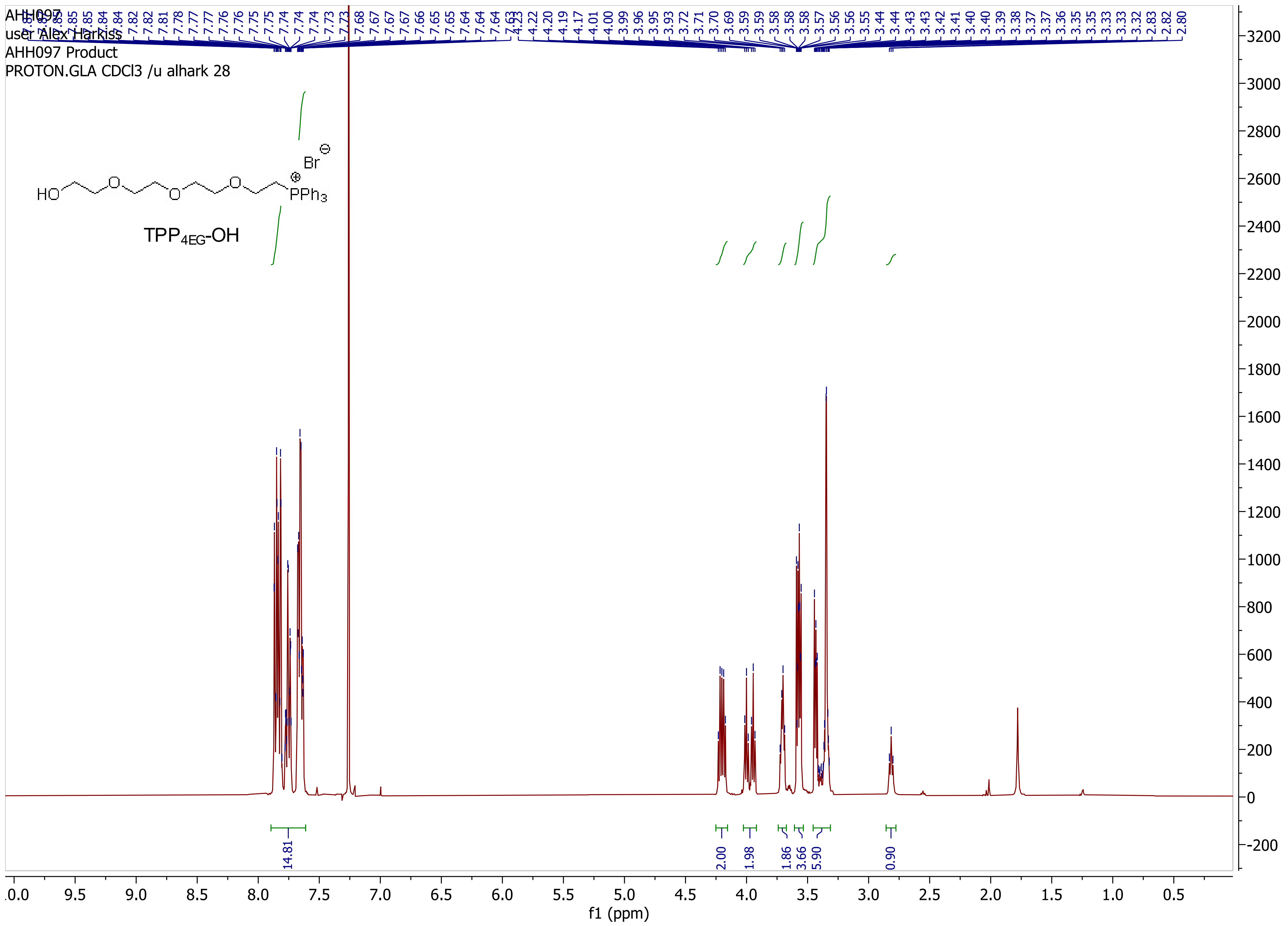
AHH097
user Alex Harkiss
AHH097 Product
PROTON.GLA CDCl3 /u alhark 28
TPP4EG-OH
Br
PPh3
HO
f1 (ppm)
14.81
2.00
1.98
1.86
3.66
5.90
0.90

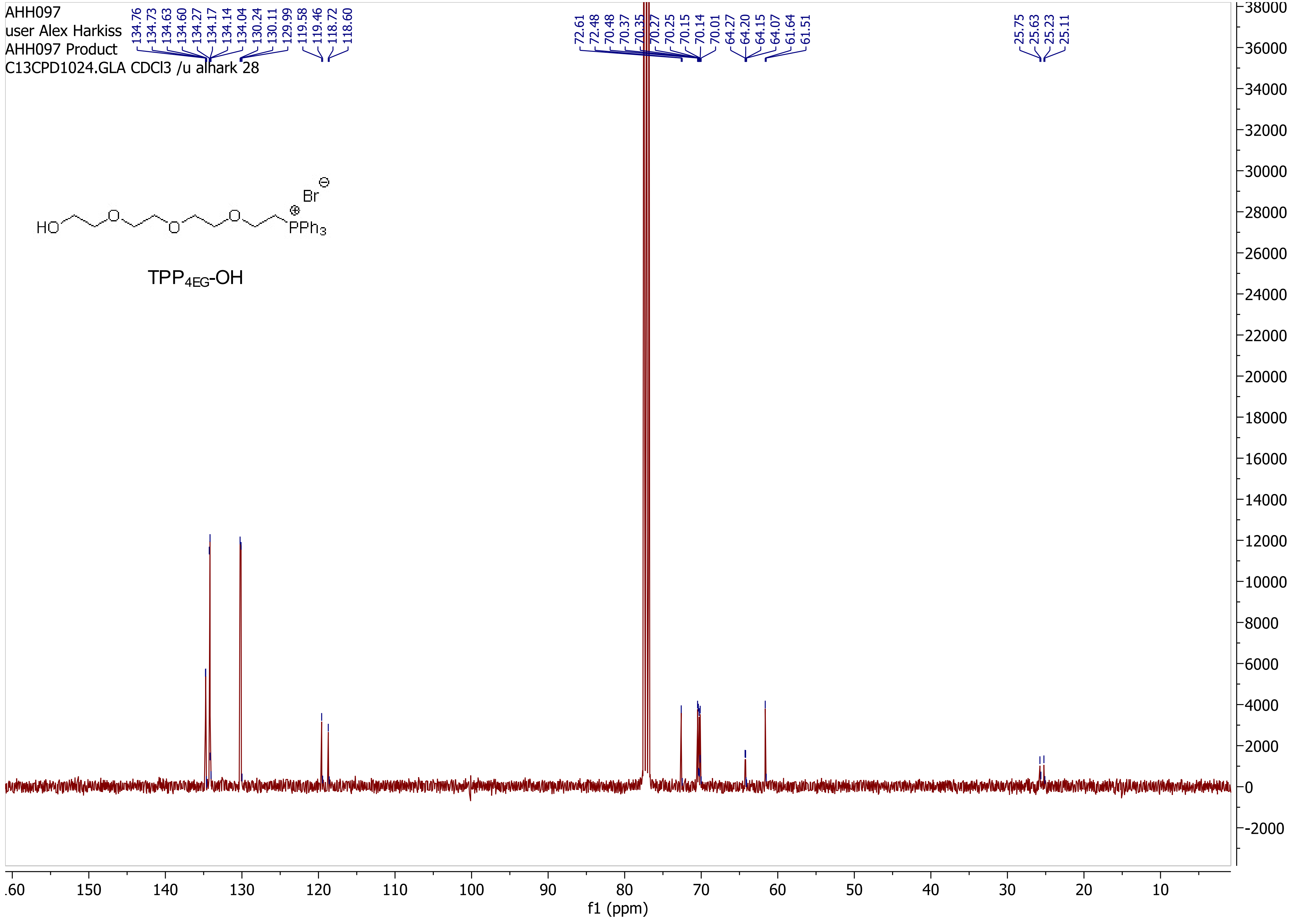

AHH097
user Alex Harkiss
AHH097 Product
C13CPD1024.GLA CDCl3 /u alhark 28
134.76
134.73
134.63
134.60
134.27
134.17
134.14
134.04
130.24
130.11
129.99
119.58
119.46
118.72
118.60
72.61
72.48
70.48
70.37
70.35
70.27
70.25
70.15
70.14
70.01
64.27
64.20
64.15
64.07
61.64
61.51
25.75
25.63
25.23
25.11
HO
O
O
O
Br
PPh3
TPP4EG-OH
f1 (ppm)

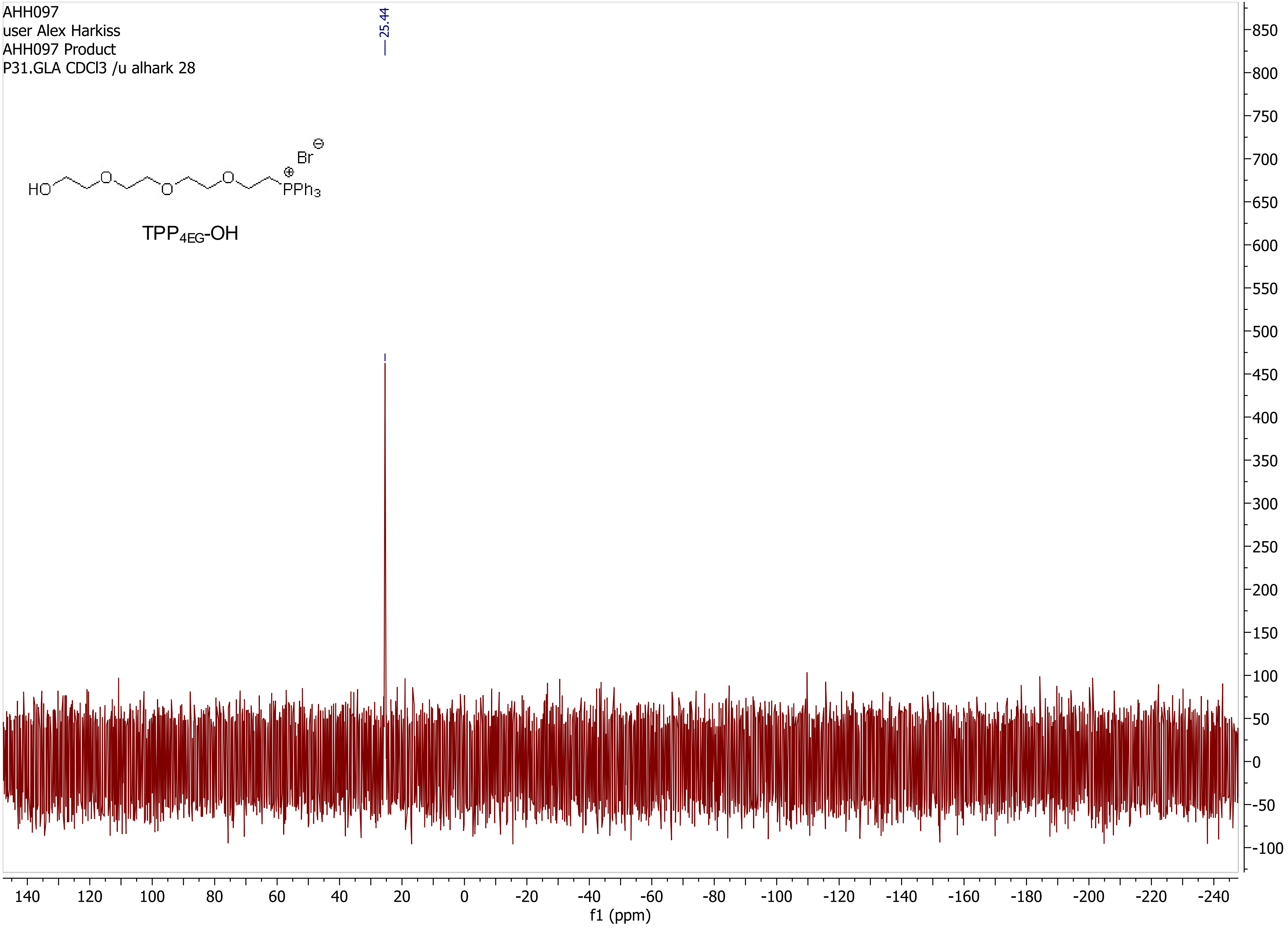
AHH097
user Alex Harkiss
AHH097 Product
P31.GLA CDCl3 /u alhark 28
25.44
Br
PPh3
HO
O
TPP4EG-OH
f1 (ppm)

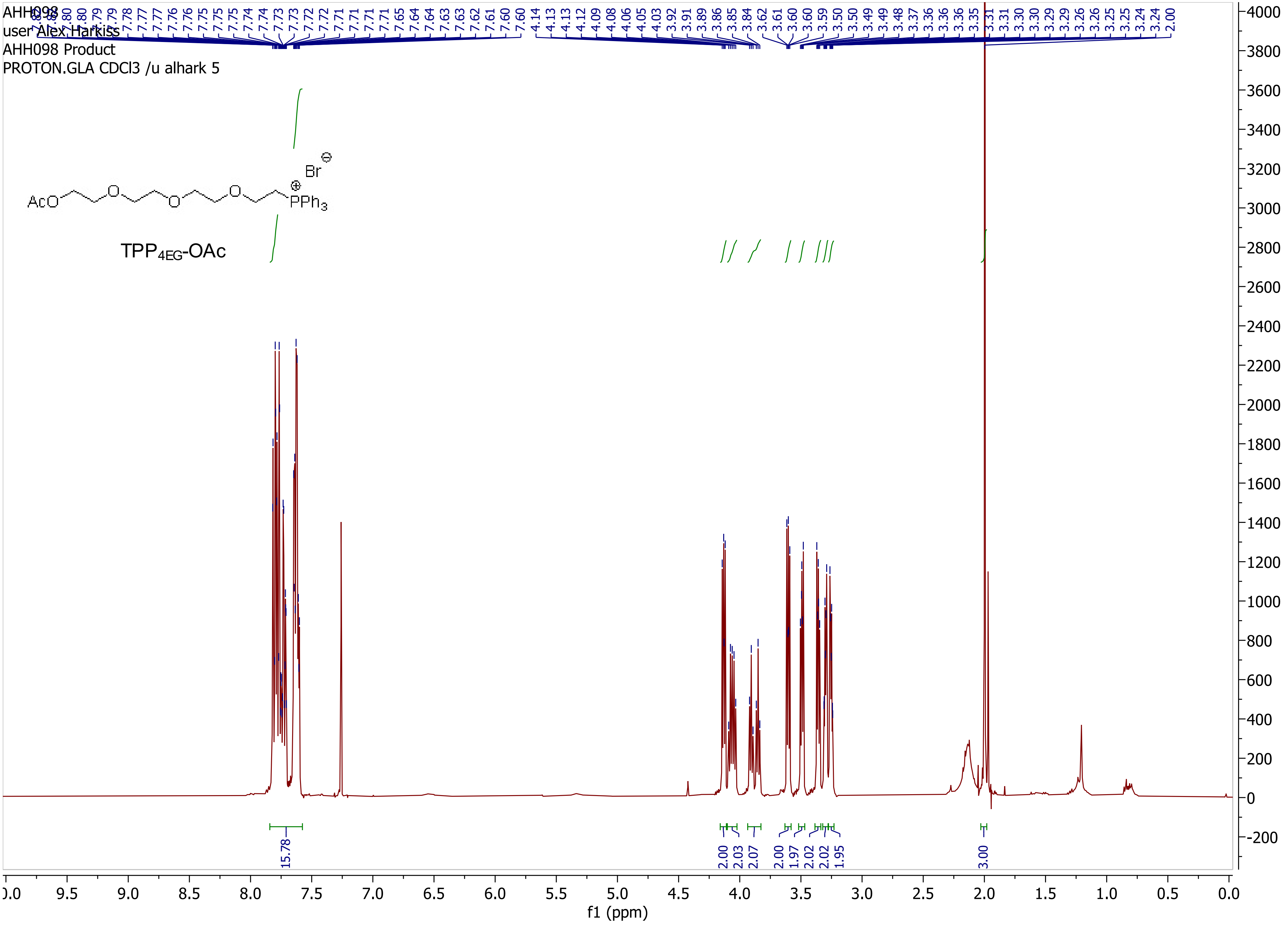
AHH098
user Alex Harkiss
AHH098 Product
PROTON.GLA CDCl3 /u alhark 5
AcO
O
O
O
⊕
PPh3
Br ⊖
TPP4EG-OAc
7.80 7.80 7.79 7.79 7.78 7.77 7.77 7.76 7.76 7.75 7.75 7.75 7.74 7.74 7.73 7.73 7.72 7.72 7.71 7.71 7.71 7.71 7.65 7.64 7.64 7.63 7.63 7.62 7.61 7.60 7.60
4.14 4.13 4.13 4.12 4.09 4.08 4.06 4.05 4.03 3.92 3.91 3.89 3.86 3.85 3.84 3.62 3.61 3.60 3.60 3.59 3.50 3.50 3.49 3.48 3.37 3.36 3.36 3.36 3.35 3.31 3.31 3.30 3.30 3.29 3.26 3.26 3.25 3.25 3.24 3.24 2.00
15.78
2.00 2.03 2.07 2.00 1.97 2.02 2.02 1.95
3.00
f1 (ppm)

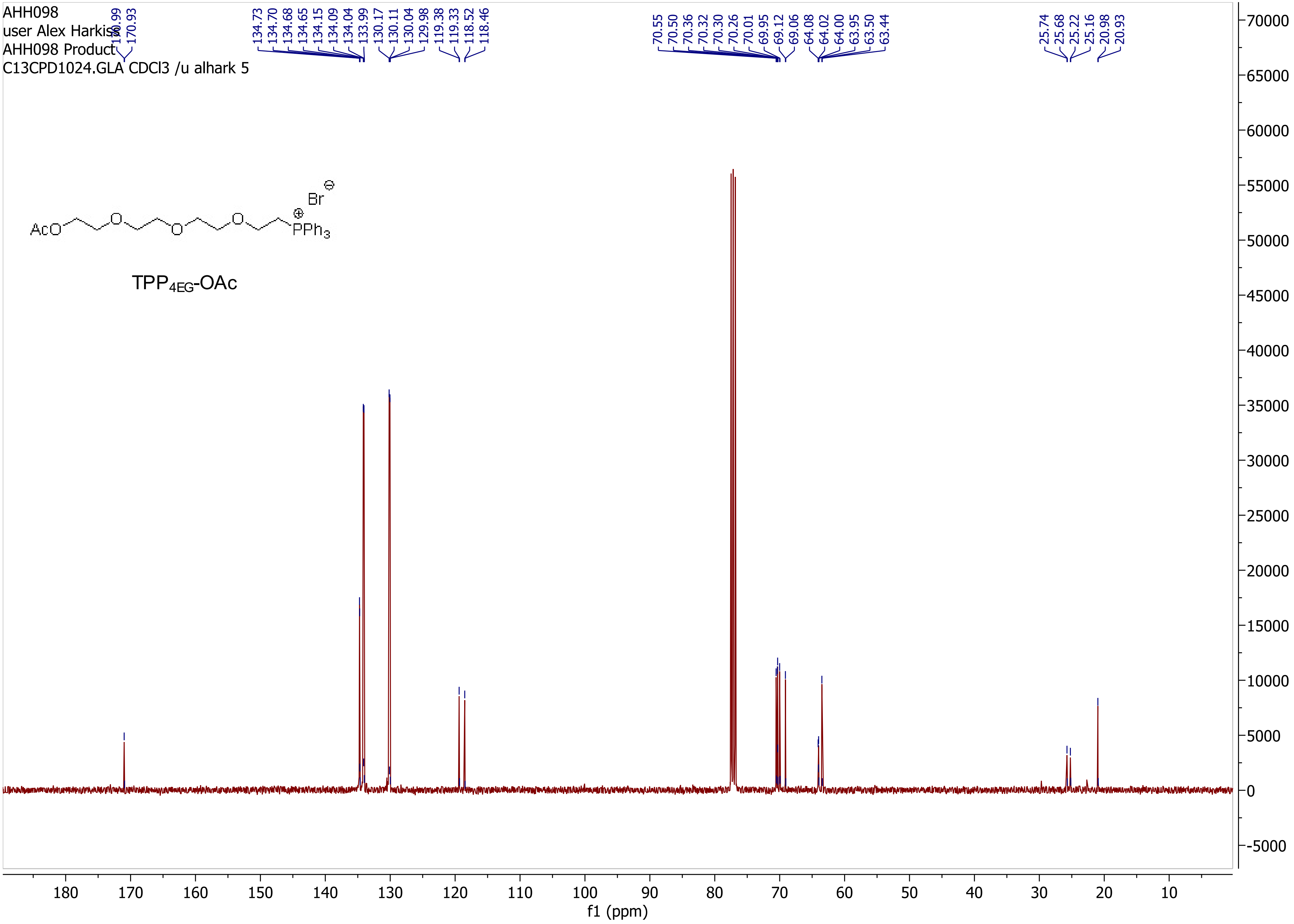
AHH098
user Alex Harkiss
AHH098 Product
C13CPD1024.GLA CDCl3 /u alhark 5
170.99
170.93
134.73
134.70
134.68
134.65
134.15
134.09
134.04
133.99
130.17
130.11
130.04
129.98
119.38
119.33
118.52
118.46
70.55
70.50
70.36
70.32
70.30
70.26
70.01
69.95
69.12
69.06
64.08
64.02
64.00
63.95
63.50
63.44
25.74
25.68
25.22
25.16
20.98
20.93
AcO
O
O
O
PPh3
Br
TPP4EG-OAc
f1 (ppm)

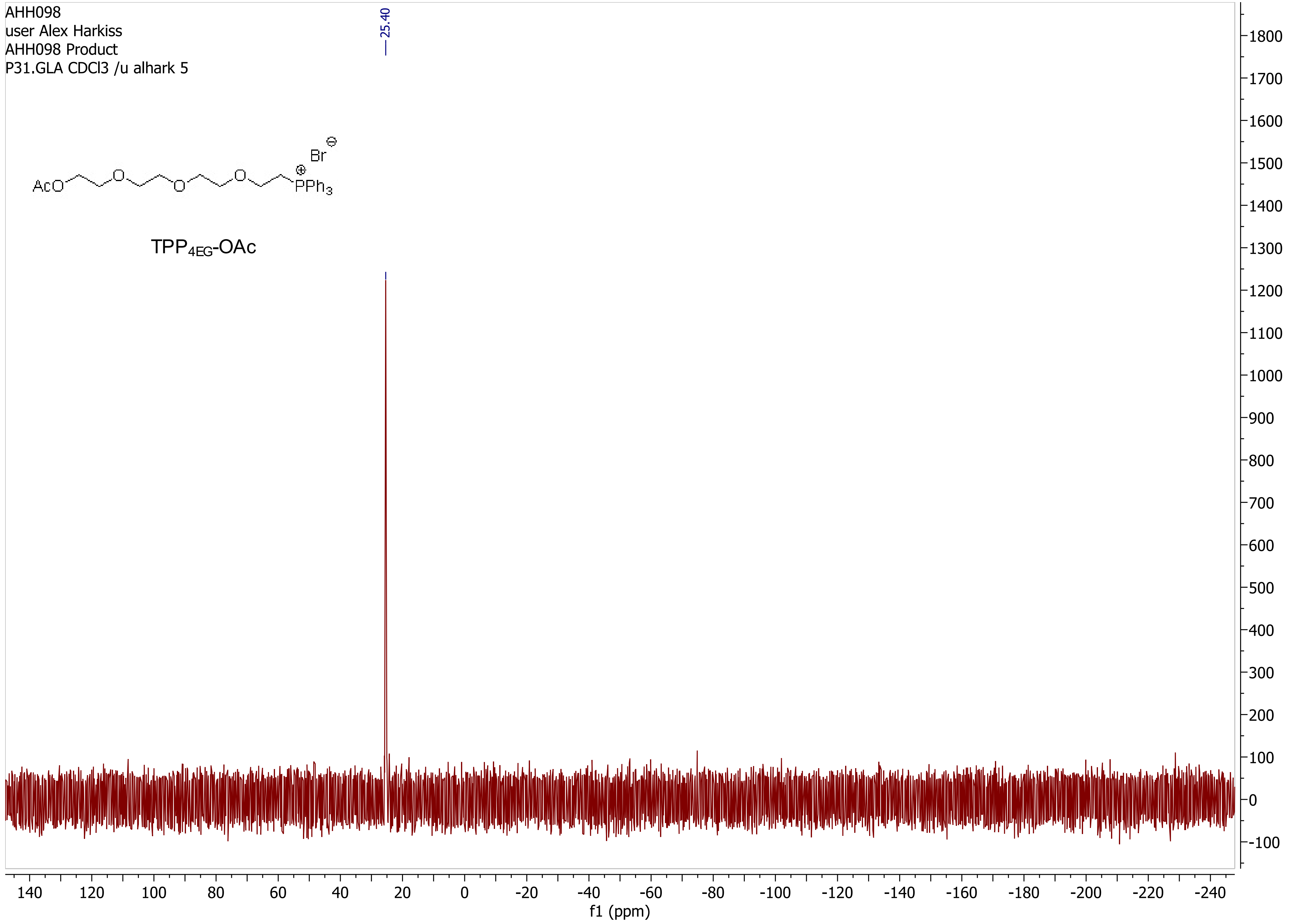
AHH098
user Alex Harkiss
AHH098 Product
P31.GLA CDCl3 /u alhark 5
25.40
AcO
O
Br
PPh3
TPP4EG-OAc
f1 (ppm)

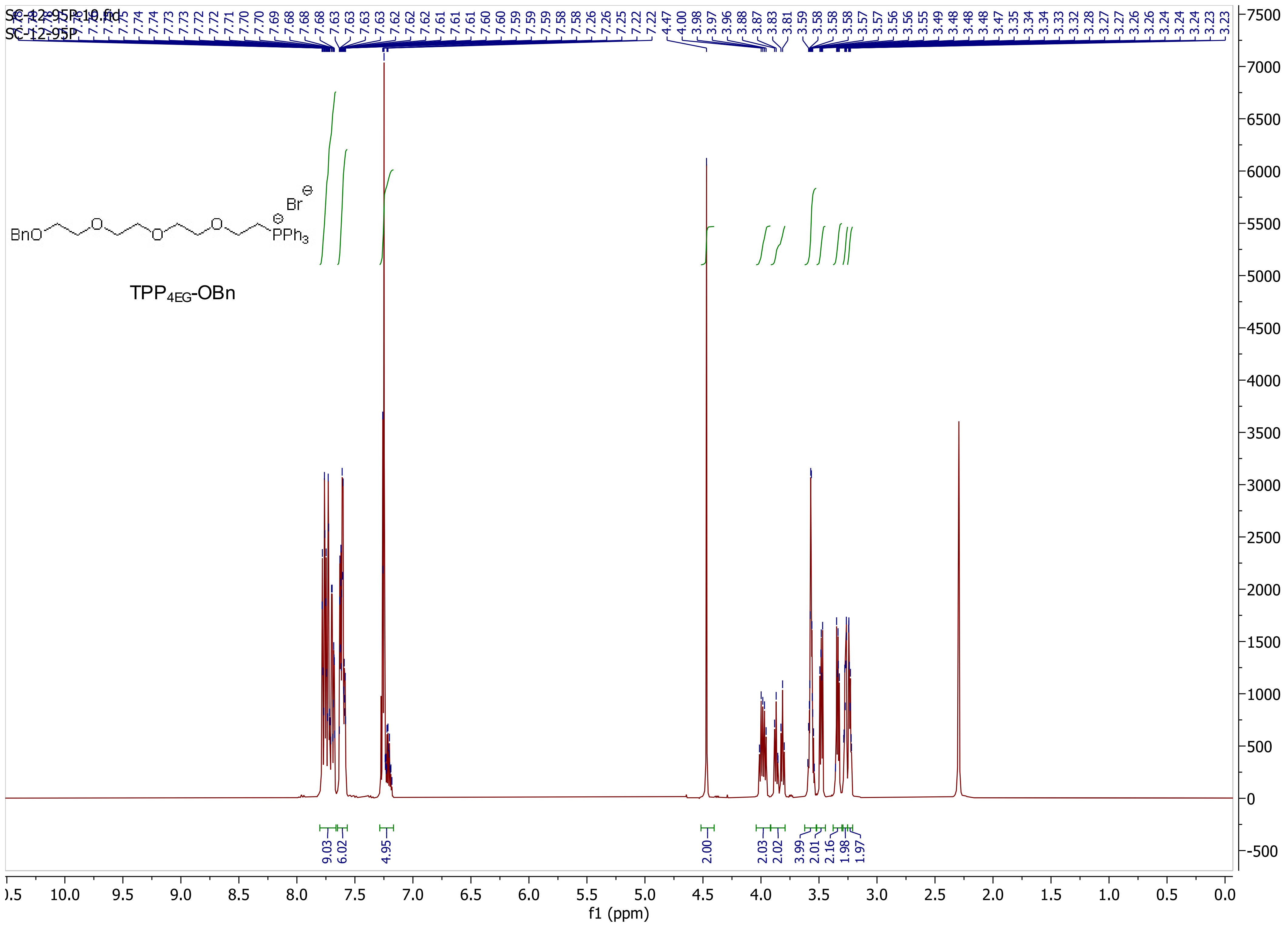

SC-12-95P-10.fid
SC-12-95P
BnO
O
O
O
Br
PPh3
TPP4EG-OBn
9.03
6.02
4.95
2.00
2.03
2.02
3.99
2.01
2.16
1.98
1.97
f1 (ppm)

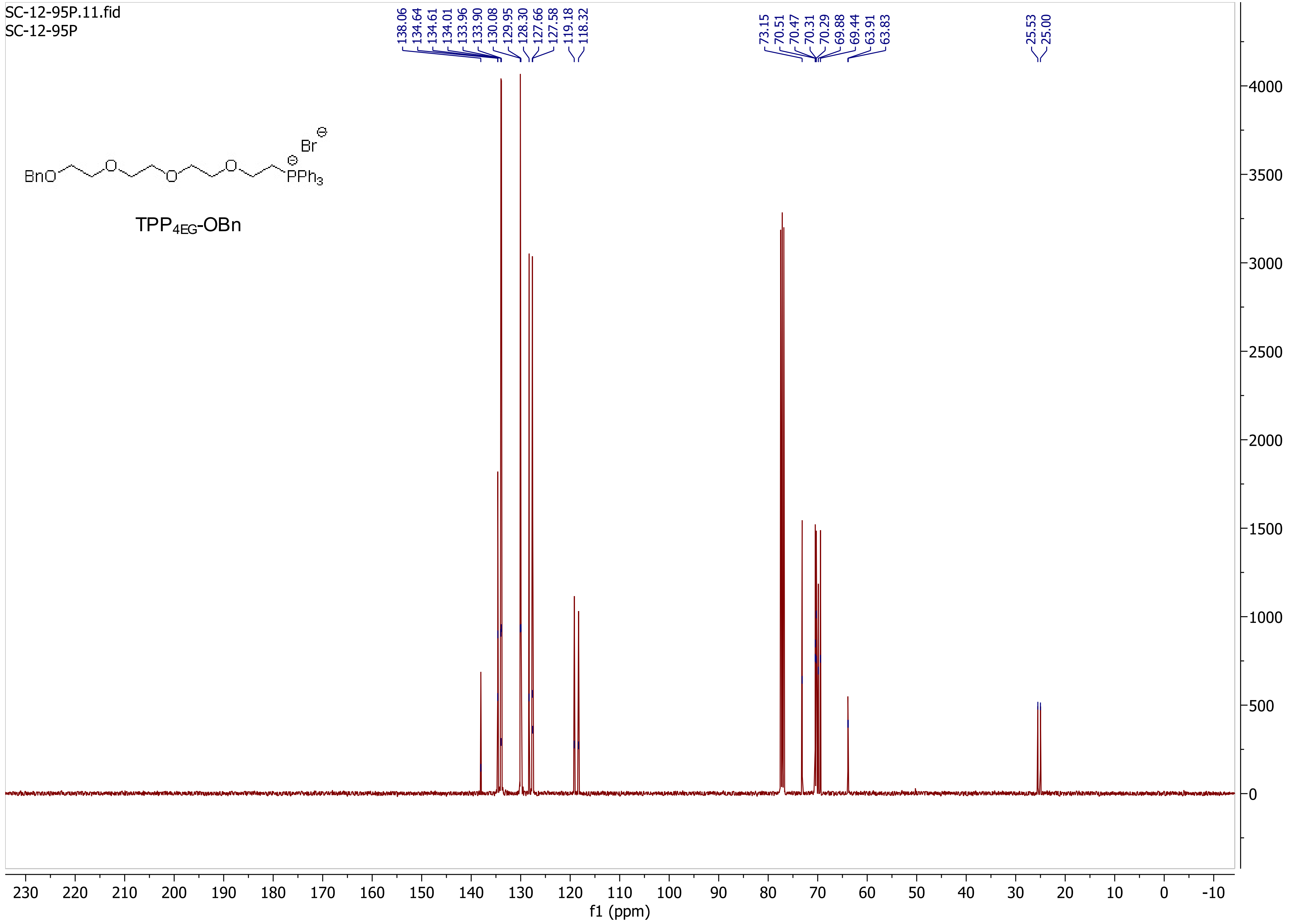
SC-12-95P.11.fid
SC-12-95P
138.06
134.64
134.61
134.01
133.96
133.90
130.08
129.95
128.30
127.66
127.58
119.18
118.32
73.15
70.51
70.47
70.31
70.29
69.88
69.44
63.91
63.83
25.53
25.00
Br
BnO
O
O
O
PPh3
TPP4EG-OBn
4000
3500
3000
2500
2000
1500
1000
500
0
230
220
210
200
190
180
170
160
150
140
130
120
110
100
90
80
70
60
50
40
30
20
10
0
-10
f1 (ppm)

SC-12-95P.15.fid
SC-12-95P

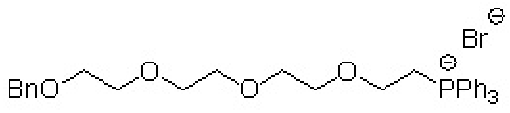

TPP$_{4EG}$-OBn

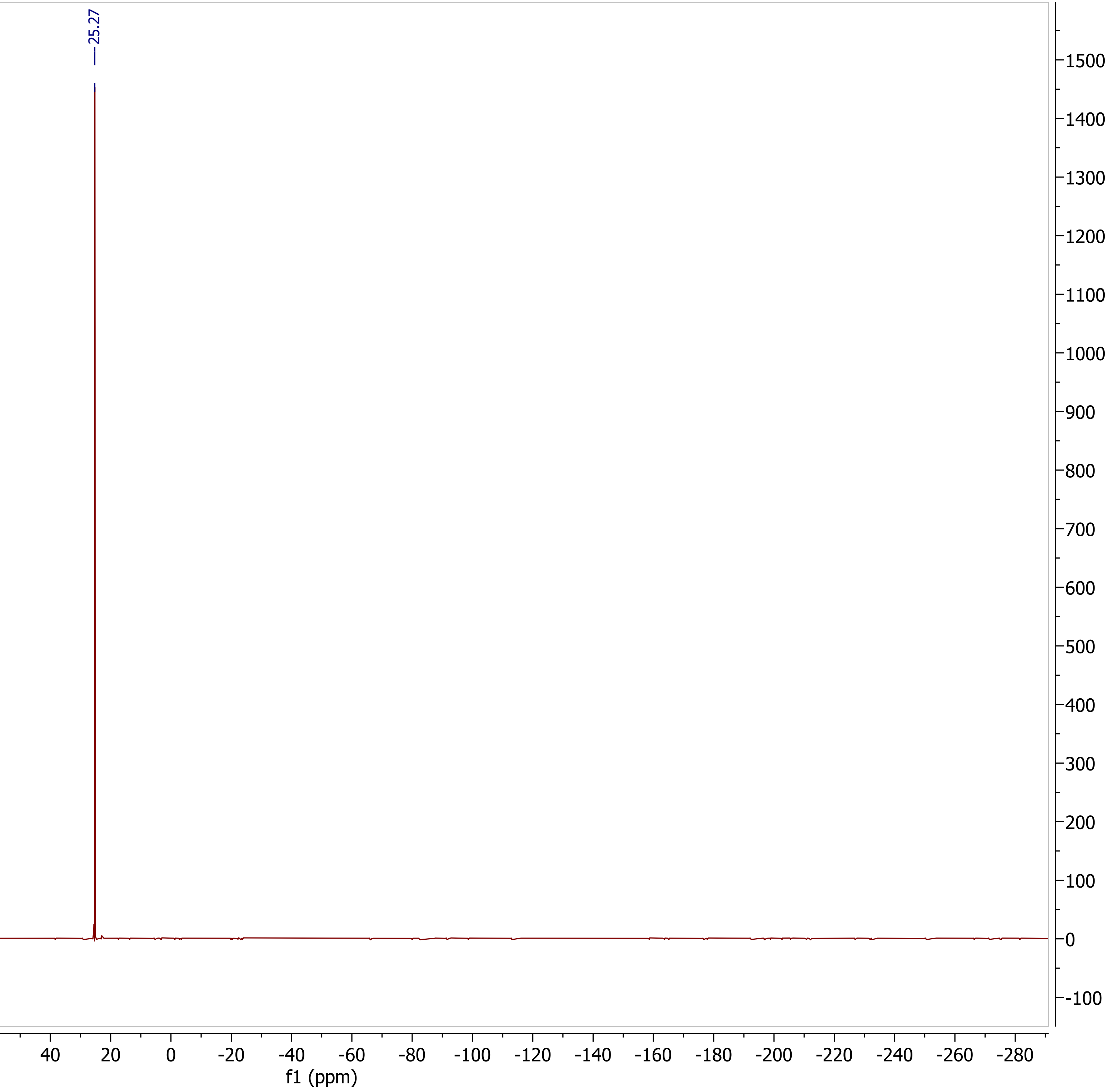

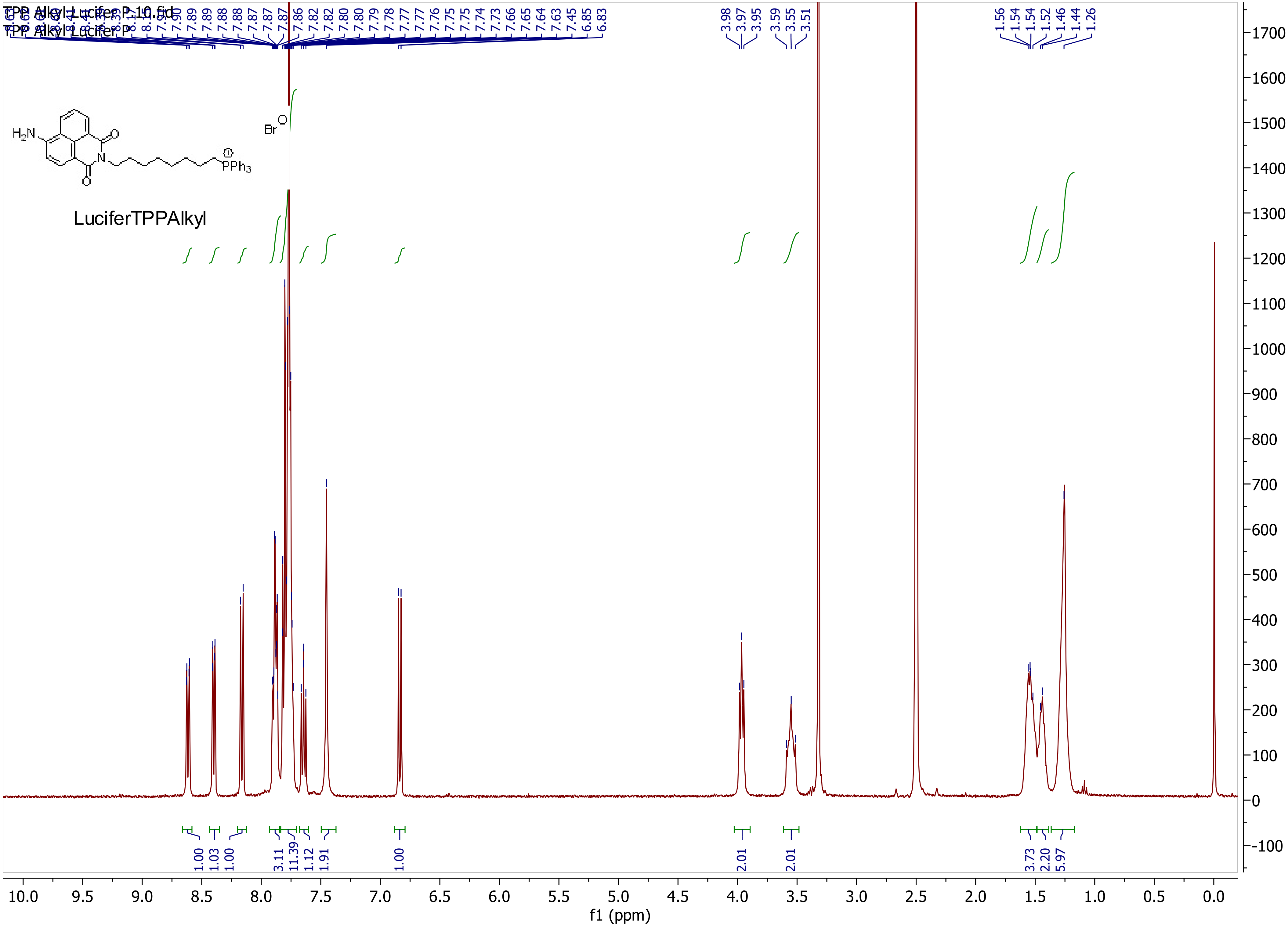
TPP Alkyl Lucifer P.10.fid
TPP Alkyl Lucifer P
7.89 7.89 7.88 7.88 7.87 7.87 7.87 7.86 7.82 7.82 7.80 7.80 7.79 7.78 7.77 7.77 7.76 7.75 7.75 7.74 7.73 7.66 7.65 7.64 7.63 7.45 6.85 6.83
3.98 3.97 3.95 3.59 3.55 3.51
1.56 1.54 1.54 1.52 1.46 1.44 1.26
H2N
N
O
O
Br
PPh3
LuciferTPPAlkyl
1.00 1.03 1.00 3.11 11.39 1.12 1.91 1.00 2.01 2.01 3.73 2.20 5.97
f1 (ppm)

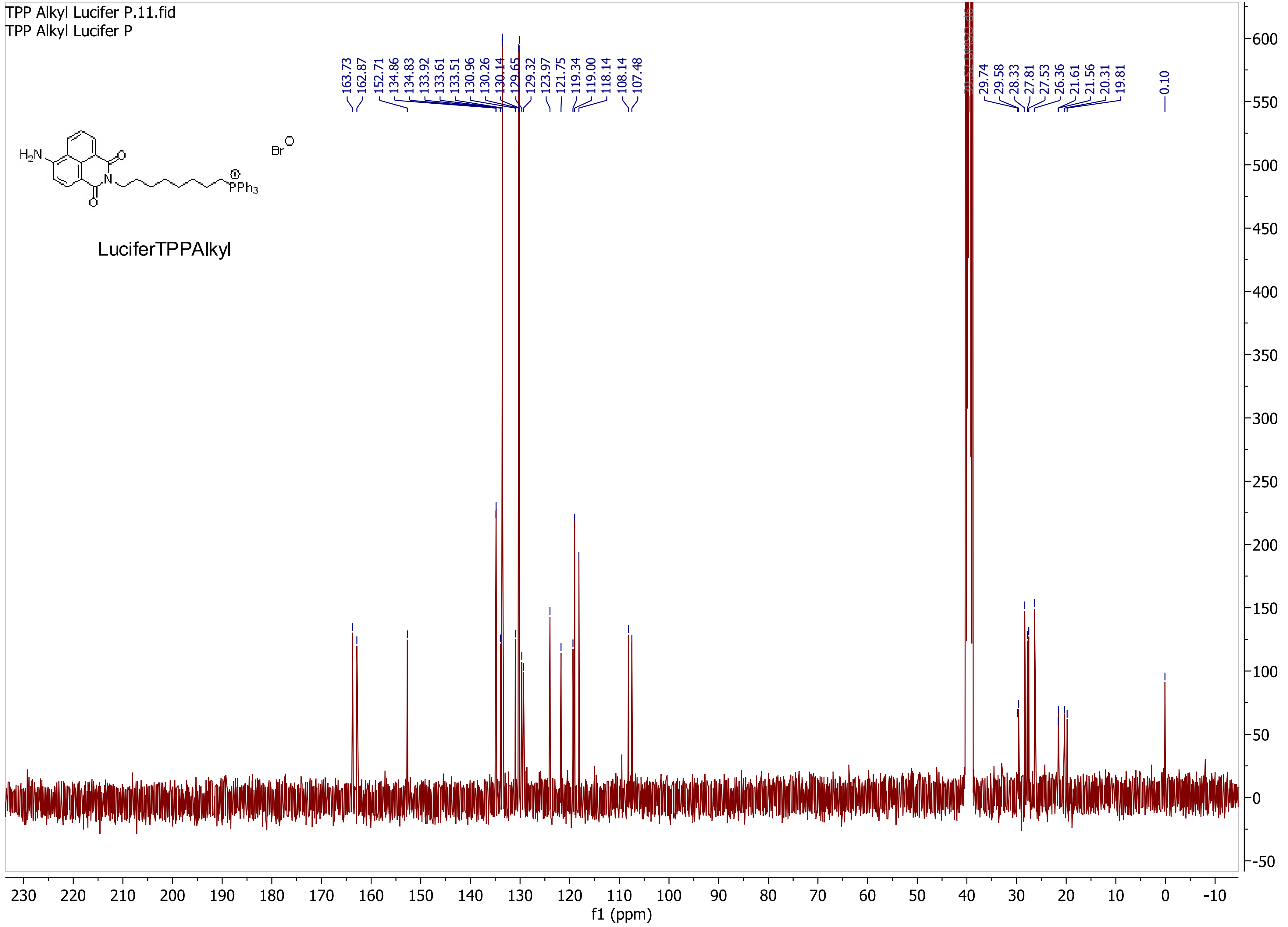
TPP Alkyl Lucifer P.11.fid
TPP Alkyl Lucifer P
H2N
N
Br
PPh3
LuciferTPPAlkyl
163.73
162.87
152.71
134.86
134.83
133.92
133.61
133.51
130.96
130.26
130.14
129.65
129.32
123.97
121.75
119.34
119.00
118.14
108.14
107.48
29.74
29.58
28.33
27.81
27.53
26.36
21.61
21.56
20.31
19.81
0.10
f1 (ppm)

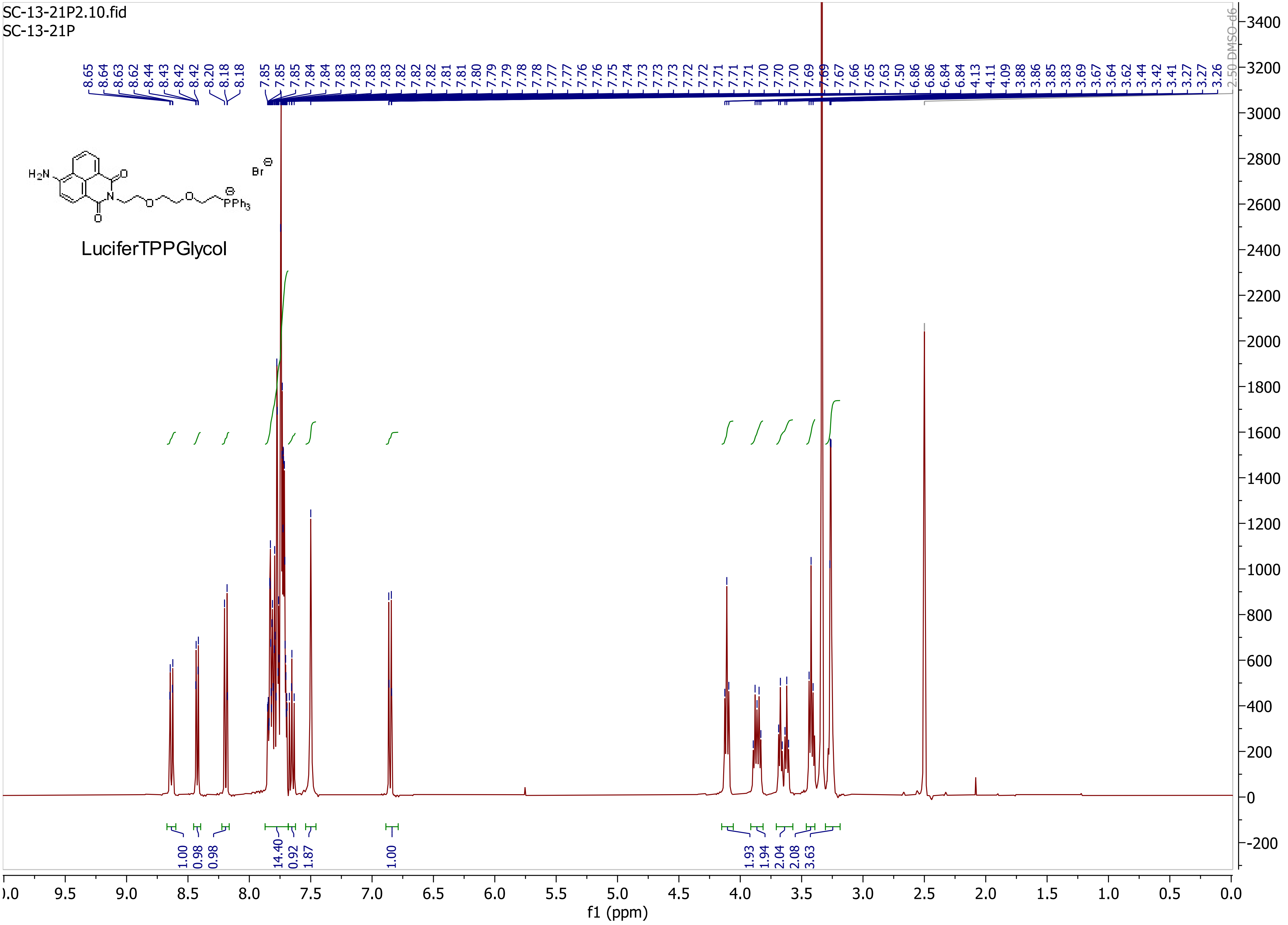
SC-13-21P2.10.fid
SC-13-21P
LuciferTPPGlycol
8.65 8.64 8.63 8.62 8.44 8.43 8.42 8.42 8.20 8.18 8.18 7.85 7.85 7.85 7.84 7.83 7.83 7.83 7.83 7.82 7.82 7.82 7.81 7.80 7.79 7.79 7.78 7.78 7.77 7.77 7.76 7.76 7.75 7.74 7.73 7.73 7.73 7.72 7.72 7.71 7.71 7.71 7.70 7.70 7.70 7.69 7.69 7.67 7.66 7.65 7.63 7.50 6.86 6.86 6.84 6.84 4.13 4.11 4.09 3.88 3.86 3.85 3.83 3.69 3.67 3.64 3.62 3.44 3.42 3.41 3.27 3.27 3.26
2.50 DMSO-d6
1.00 0.98 0.98 14.40 0.92 1.87 1.00 1.93 1.94 2.04 2.08 3.63
f1 (ppm)

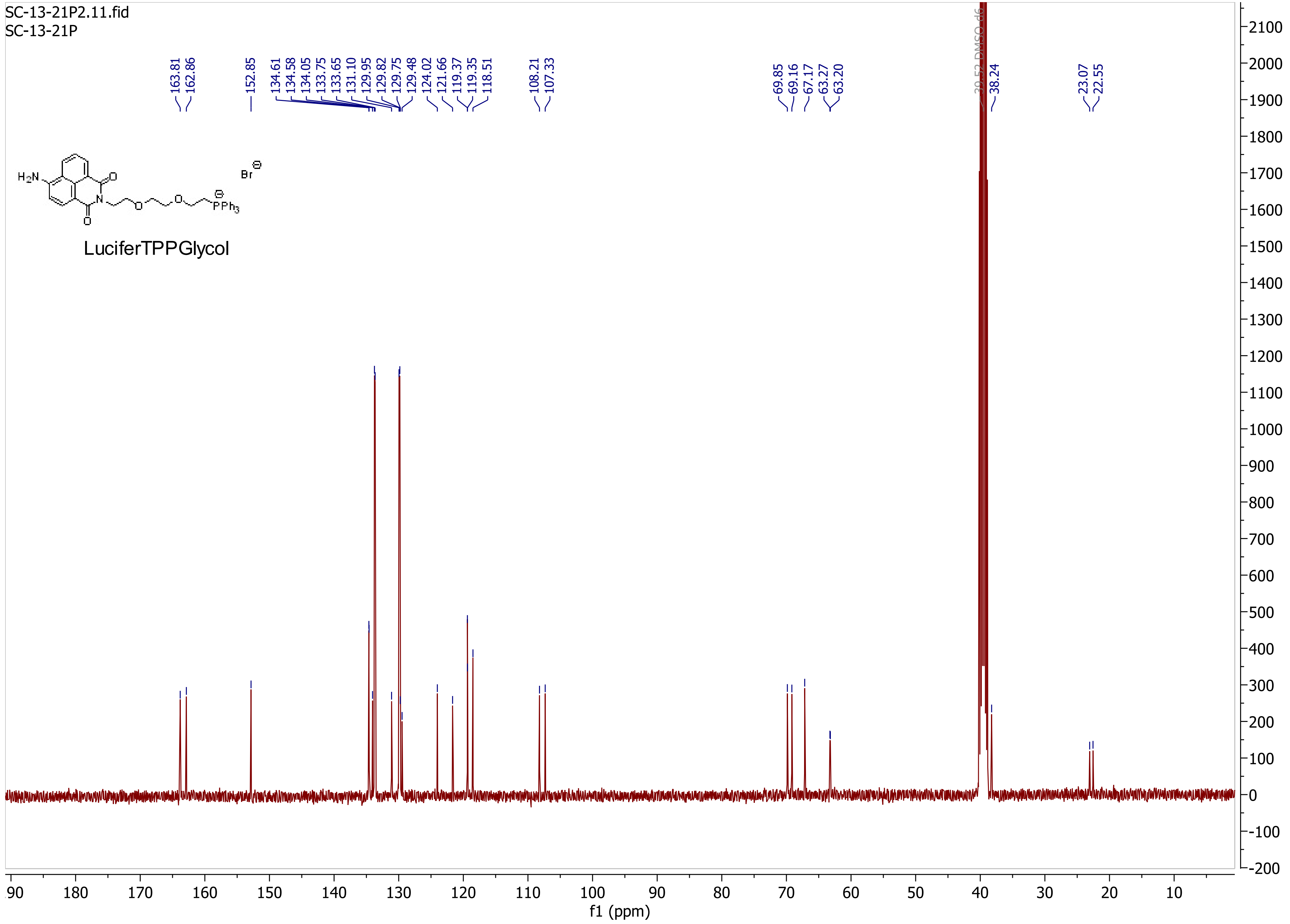
SC-13-21P2.11.fid
SC-13-21P
163.81
162.86
152.85
134.61
134.58
134.05
133.75
133.65
131.10
129.95
129.82
129.75
129.48
124.02
121.66
119.37
119.35
118.51
108.21
107.33
69.85
69.16
67.17
63.27
63.20
39.52 DMSO-d6
38.24
23.07
22.55
LuciferTPPGlycol
f1 (ppm)

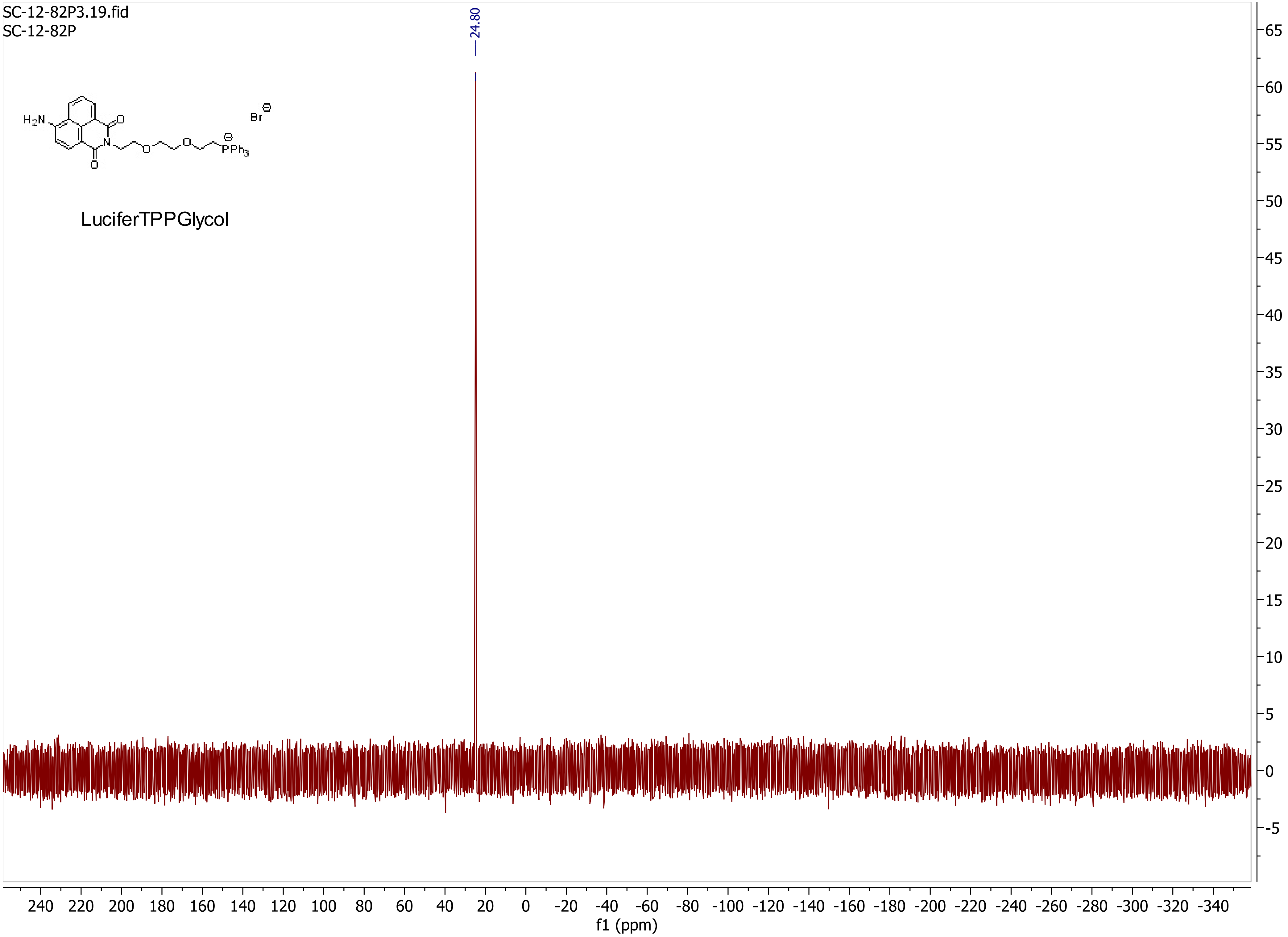
SC-12-82P3.19.fid
SC-12-82P
H2N
O
N
O
O
O
PPh3
Br
LuciferTPPGlycol
24.80
65
60
55
50
45
40
35
30
25
20
15
10
5
0
-5
240 220 200 180 160 140 120 100 80 60 40 20 0 -20 -40 -60 -80 -100 -120 -140 -160 -180 -200 -220 -240 -260 -280 -300 -320 -340
f1 (ppm)

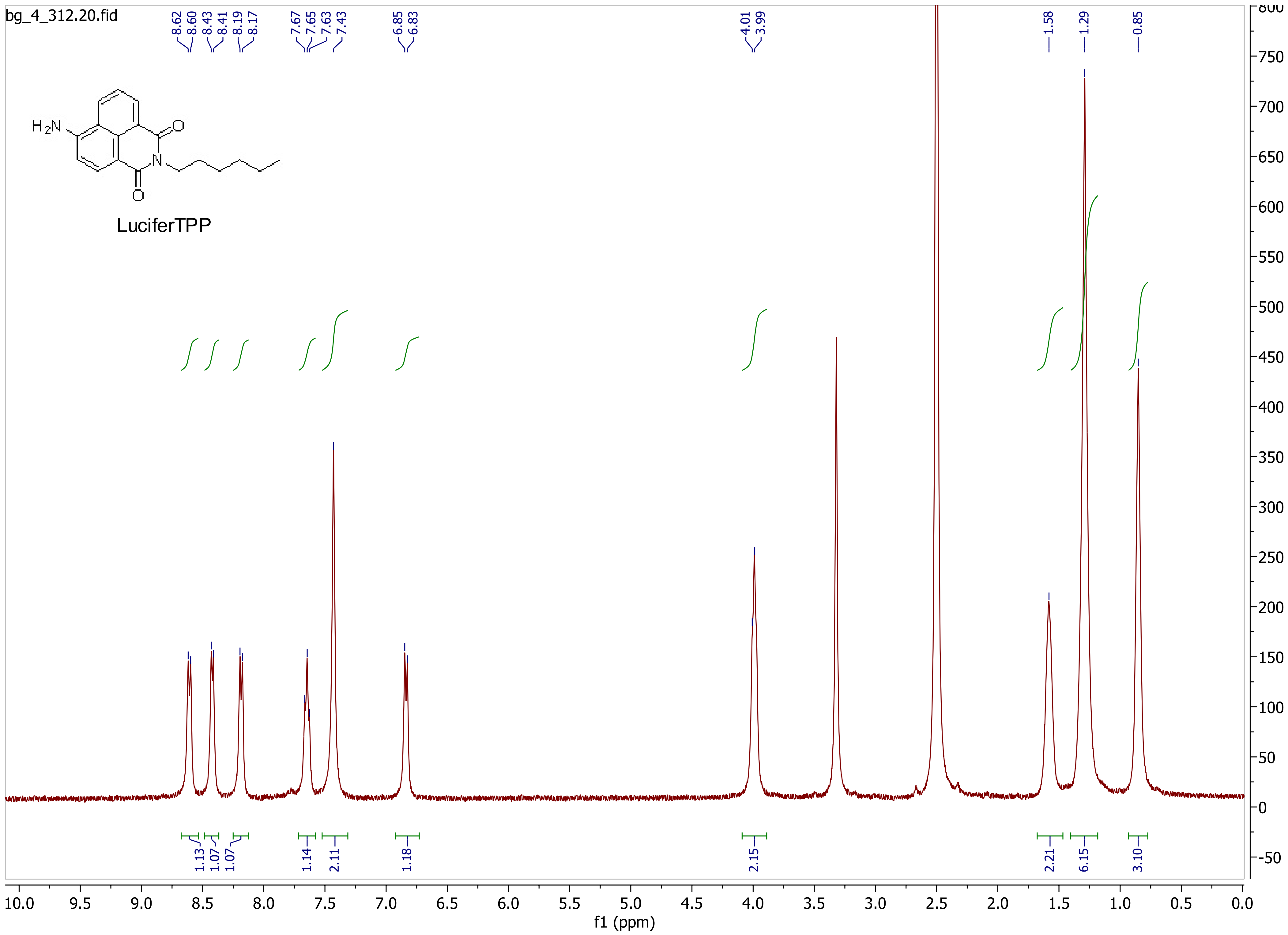

bg_4_312.20.fid
8.62
8.60
8.43
8.41
8.19
8.17
7.67
7.65
7.63
7.43
6.85
6.83
4.01
3.99
1.58
1.29
0.85
H2N
O
N
O
LuciferTPP
1.13
1.07
1.07
1.14
2.11
1.18
2.15
2.21
6.15
3.10
f1 (ppm)

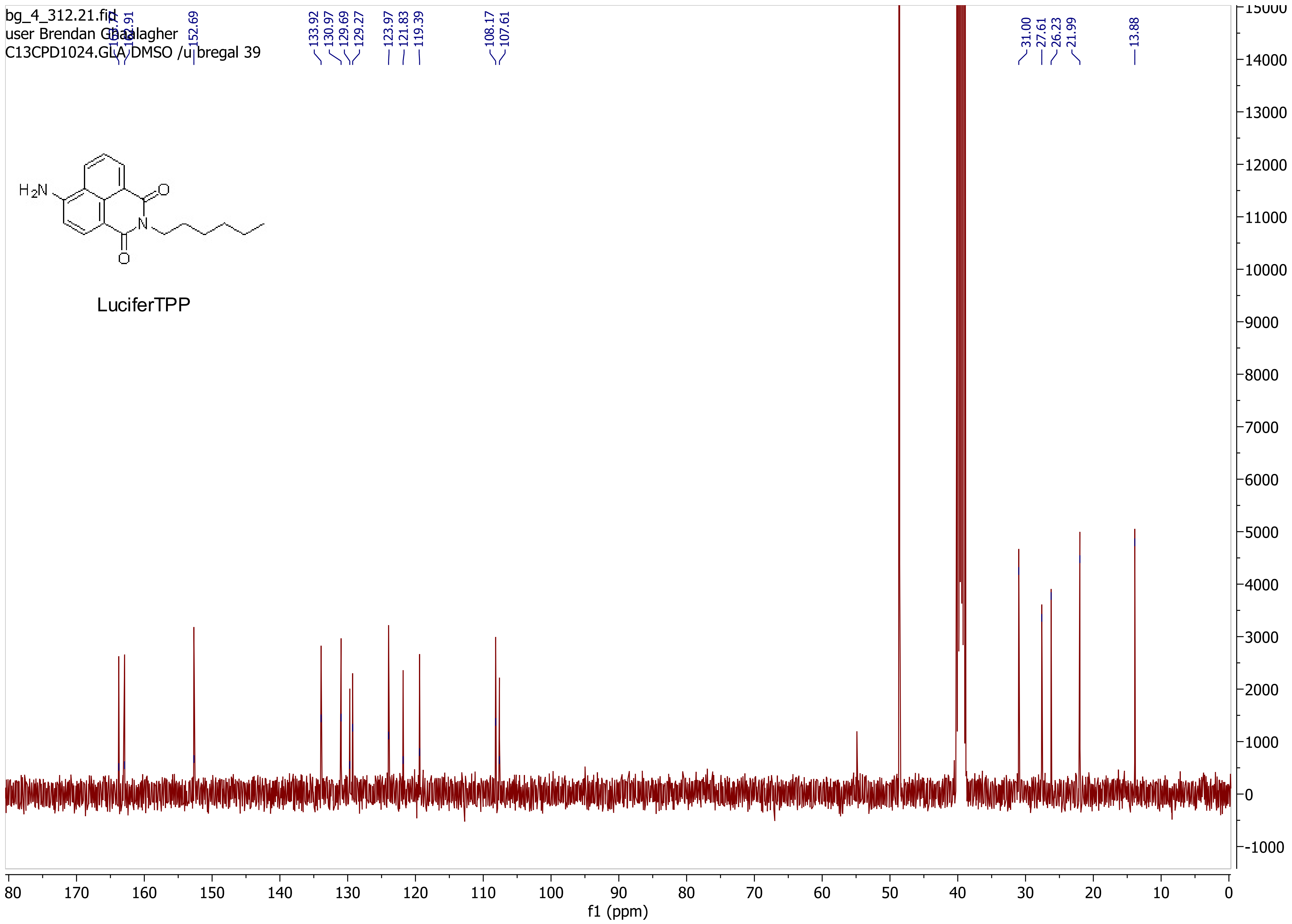

bg_4_312.21.fid
user Brendan Gallagher
C13CPD1024.GLA DMSO /u bregal 39
163.77
162.91
152.69
133.92
130.97
129.69
129.27
123.97
121.83
119.39
108.17
107.61
31.00
27.61
26.23
21.99
13.88
H2N
O
N
O
LuciferTPP
15000
14000
13000
12000
11000
10000
9000
8000
7000
6000
5000
4000
3000
2000
1000
0
-1000
180
170
160
150
140
130
120
110
100
90
80
70
60
50
40
30
20
10
0
f1 (ppm)

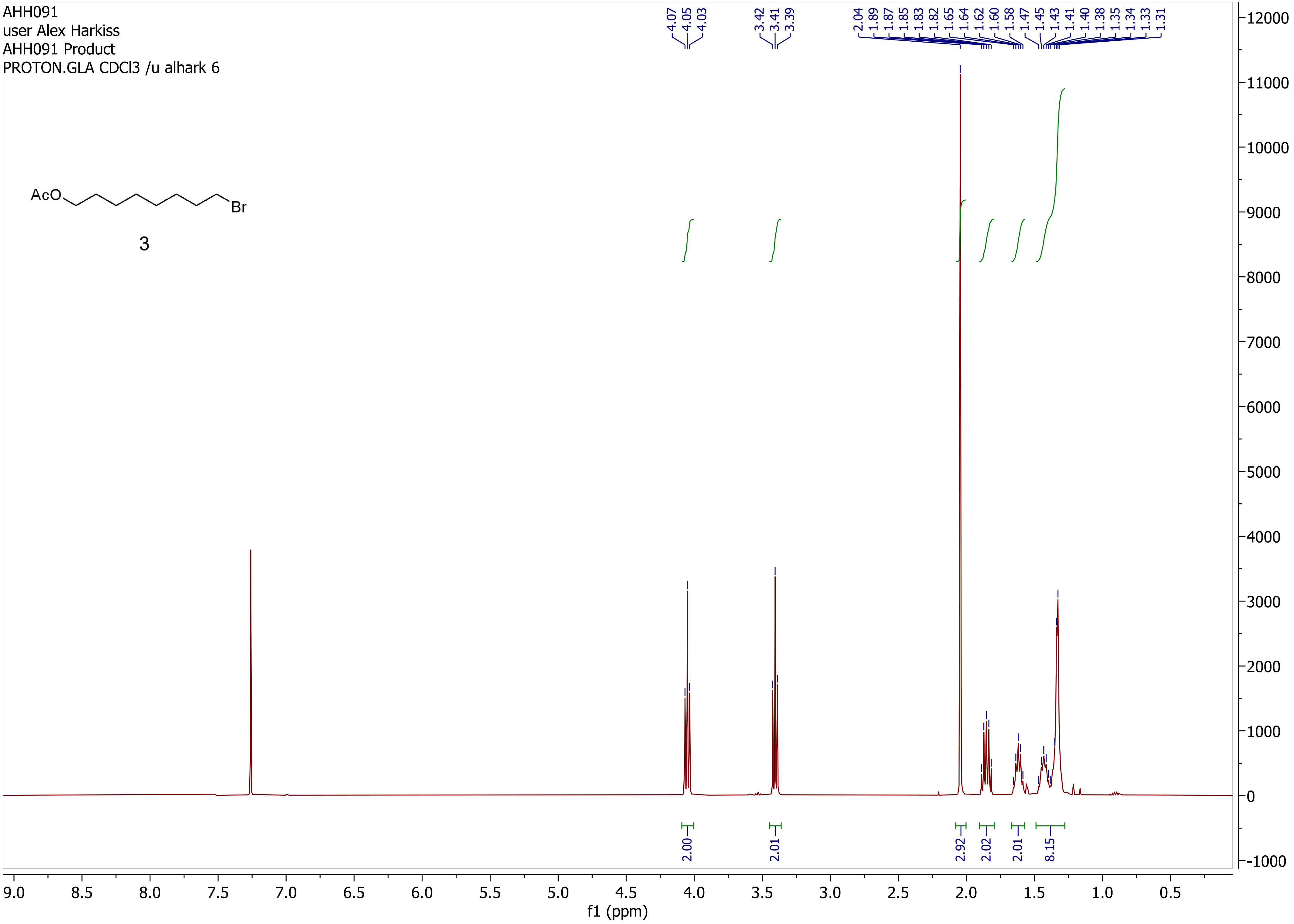
AHH091
user Alex Harkiss
AHH091 Product
PROTON.GLA CDCl3 /u alhark 6
AcO
Br
3
4.07
4.05
4.03
3.42
3.41
3.39
2.04
1.89
1.87
1.85
1.83
1.82
1.65
1.64
1.62
1.60
1.58
1.47
1.45
1.43
1.41
1.40
1.38
1.35
1.34
1.33
1.31
2.00
2.01
2.92
2.02
2.01
8.15
f1 (ppm)

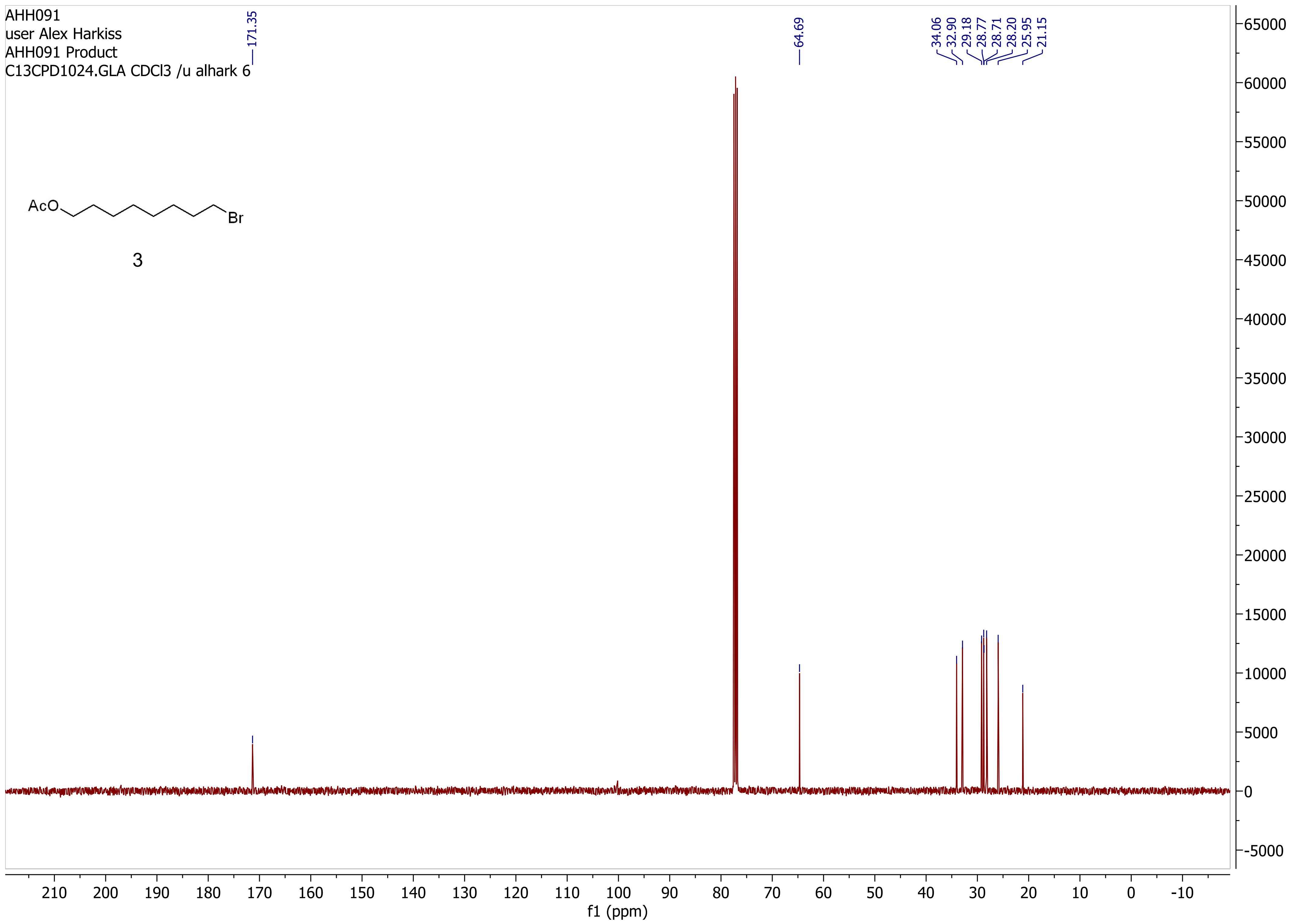
AHH091
user Alex Harkiss
AHH091 Product
C13CPD1024.GLA CDCl3 /u alhark 6
171.35
64.69
34.06
32.90
29.18
28.77
28.71
28.20
25.95
21.15
AcO
Br
3
65000
60000
55000
50000
45000
40000
35000
30000
25000
20000
15000
10000
5000
0
-5000
210 200 190 180 170 160 150 140 130 120 110 100 90 80 70 60 50 40 30 20 10 0 -10
f1 (ppm)

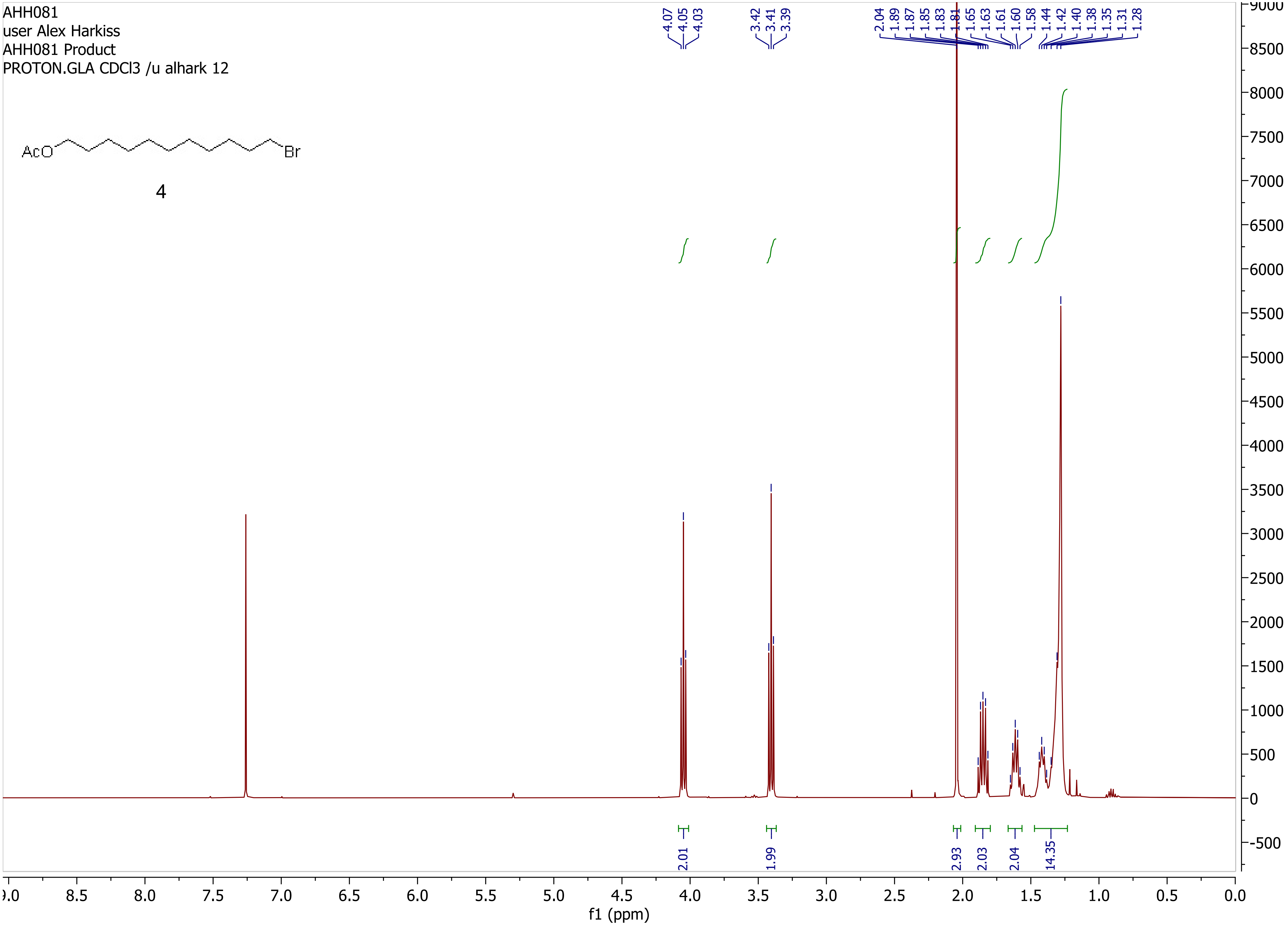
AHH081
user Alex Harkiss
AHH081 Product
PROTON.GLA CDCl3 /u alhark 12
AcO
Br
4
4.07
4.05
4.03
3.42
3.41
3.39
2.04
1.89
1.87
1.85
1.83
1.81
1.65
1.63
1.61
1.60
1.58
1.44
1.42
1.40
1.38
1.35
1.31
1.28
2.01
1.99
2.93
2.03
2.04
14.35
f1 (ppm)

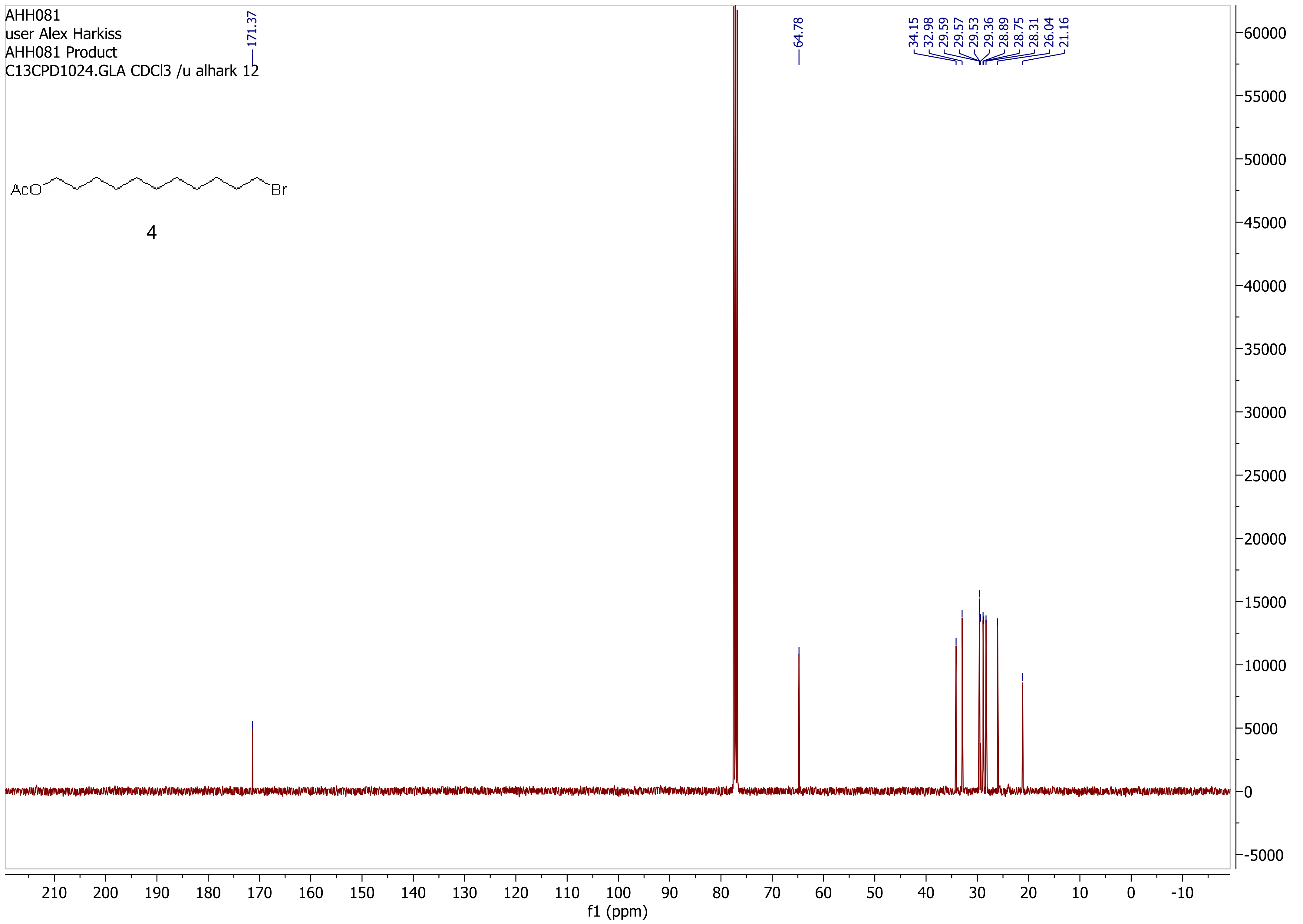
AHH081
user Alex Harkiss
AHH081 Product
C13CPD1024.GLA CDCl3 /u alhark 12
171.37
64.78
34.15
32.98
29.59
29.57
29.53
29.36
28.89
28.75
28.31
26.04
21.16
AcO
Br
4
60000
55000
50000
45000
40000
35000
30000
25000
20000
15000
10000
5000
0
-5000
210
200
190
180
170
160
150
140
130
120
110
100
90
80
70
60
50
40
30
20
10
0
-10
f1 (ppm)

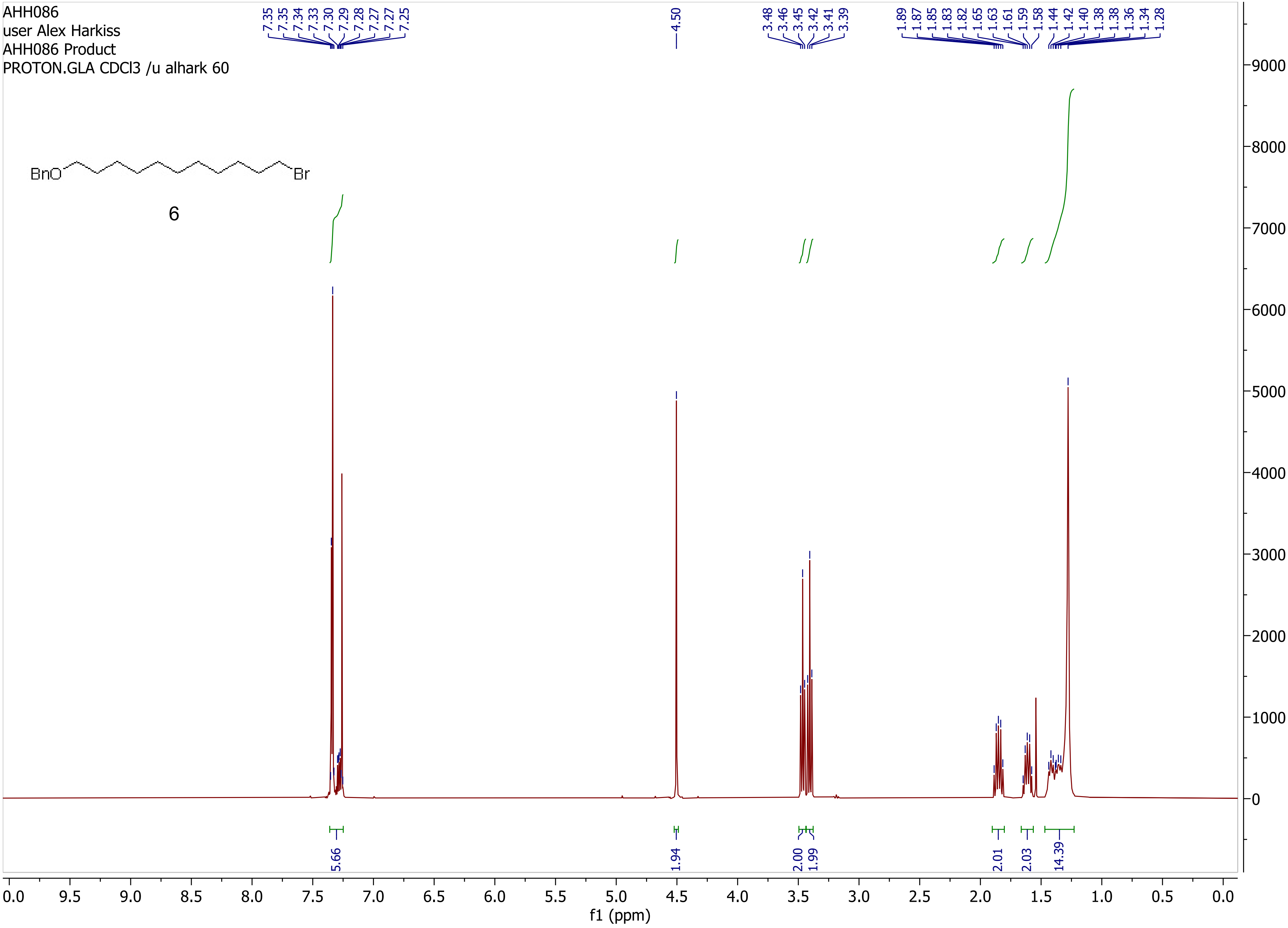
AHH086
user Alex Harkiss
AHH086 Product
PROTON.GLA CDCl3 /u alhark 60
BnO
Br
6
7.35 7.35 7.34 7.33 7.30 7.29 7.28 7.27 7.27 7.25
4.50
3.48 3.46 3.45 3.42 3.41 3.39
1.89 1.87 1.85 1.83 1.82 1.65 1.63 1.61 1.59 1.58 1.44 1.42 1.40 1.38 1.38 1.36 1.34 1.28
5.66
1.94
2.00
1.99
2.01
2.03
14.39
f1 (ppm)

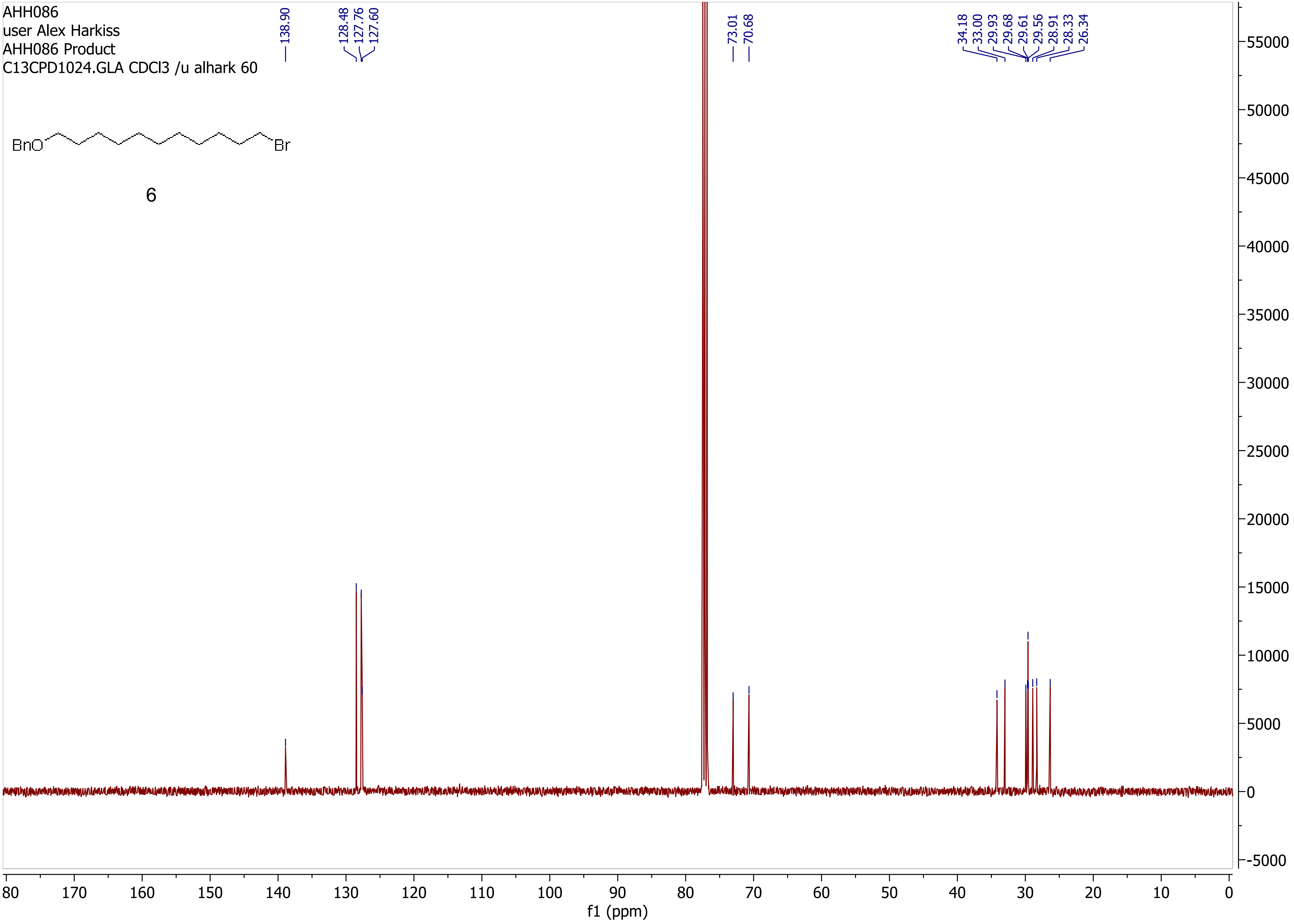
AHH086
user Alex Harkiss
AHH086 Product
C13CPD1024.GLA CDCl3 /u alhark 60
BnO
Br
6
138.90
128.48
127.76
127.60
73.01
70.68
34.18
33.00
29.93
29.68
29.61
29.56
28.91
28.33
26.34
f1 (ppm)

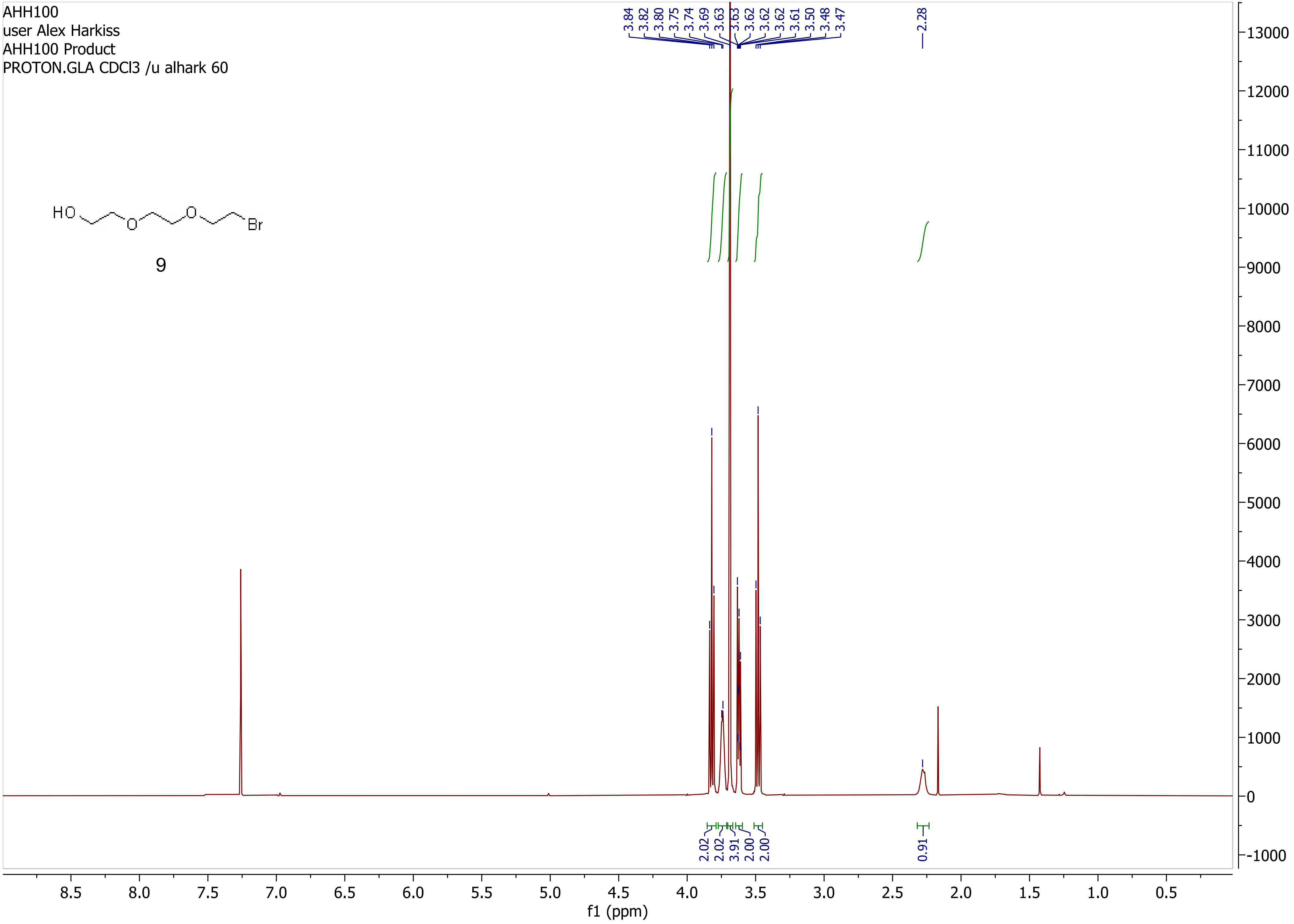
AHH100
user Alex Harkiss
AHH100 Product
PROTON.GLA CDCl3 /u alhark 60
HO
O
O
Br
9
3.84
3.82
3.80
3.75
3.74
3.69
3.63
3.63
3.62
3.62
3.62
3.61
3.50
3.48
3.47
2.28
2.02
2.02
3.91
2.00
2.00
0.91
f1 (ppm)

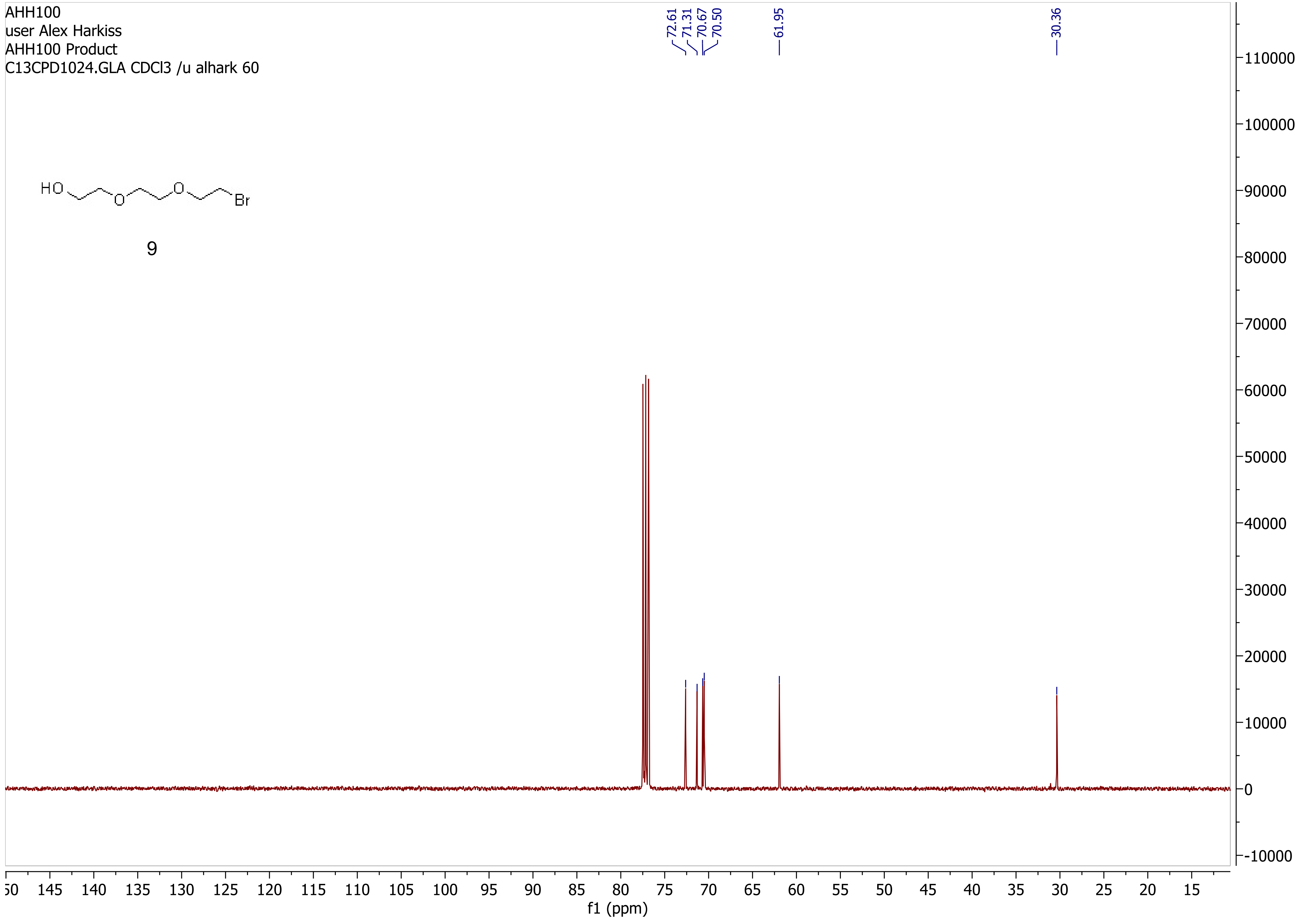
AHH100
user Alex Harkiss
AHH100 Product
C13CPD1024.GLA CDCl3 /u alhark 60
HO
O
O
Br
9
72.61
71.31
70.67
70.50
61.95
30.36
f1 (ppm)

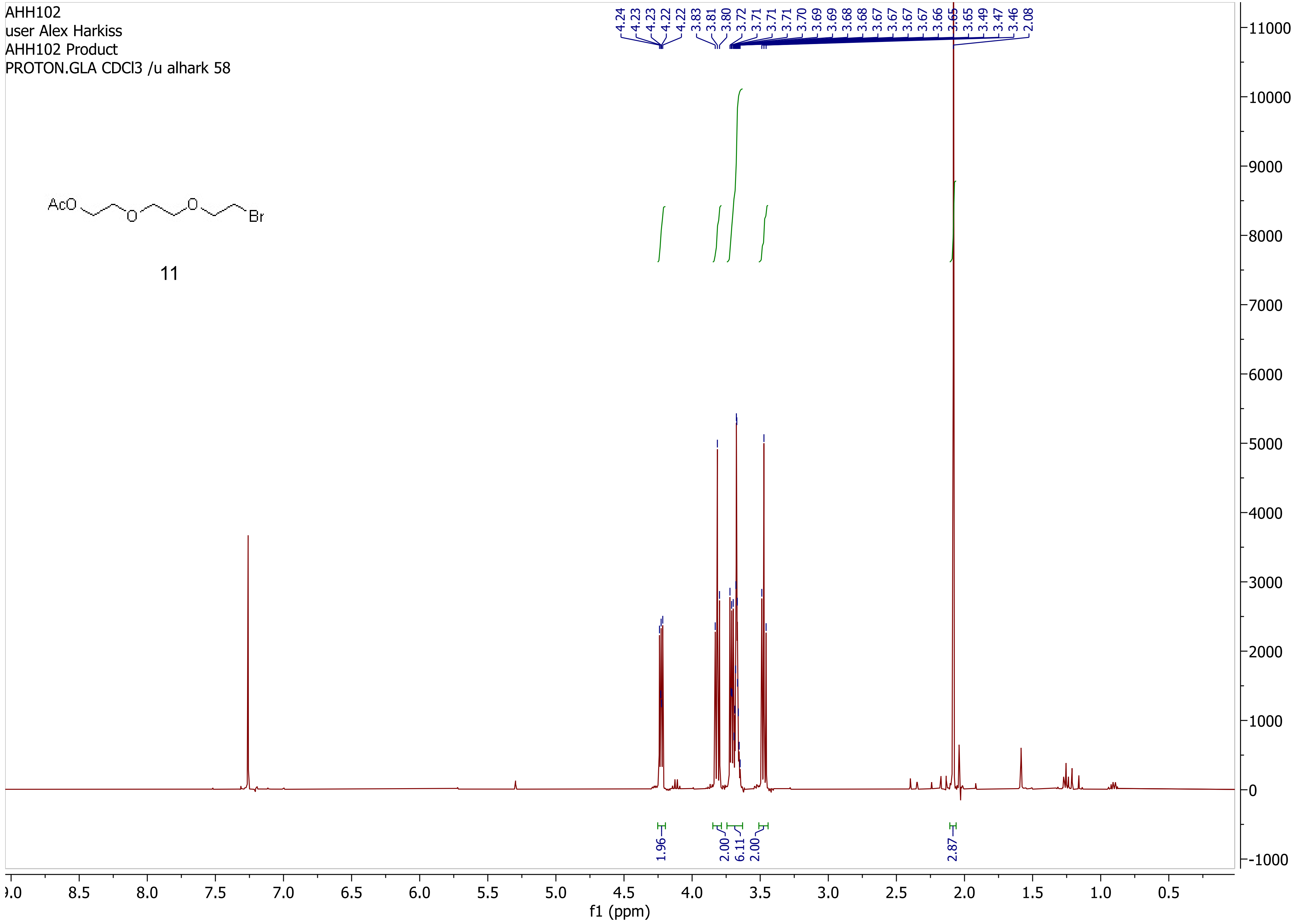
AHH102
user Alex Harkiss
AHH102 Product
PROTON.GLA CDCl3 /u alhark 58
AcO
O
O
Br
11
4.24
4.23
4.23
4.22
4.22
3.83
3.81
3.80
3.72
3.71
3.71
3.71
3.70
3.69
3.69
3.68
3.68
3.67
3.67
3.67
3.67
3.66
3.65
3.65
3.49
3.47
3.46
2.08
1.96
2.00
6.11
2.00
2.87
f1 (ppm)

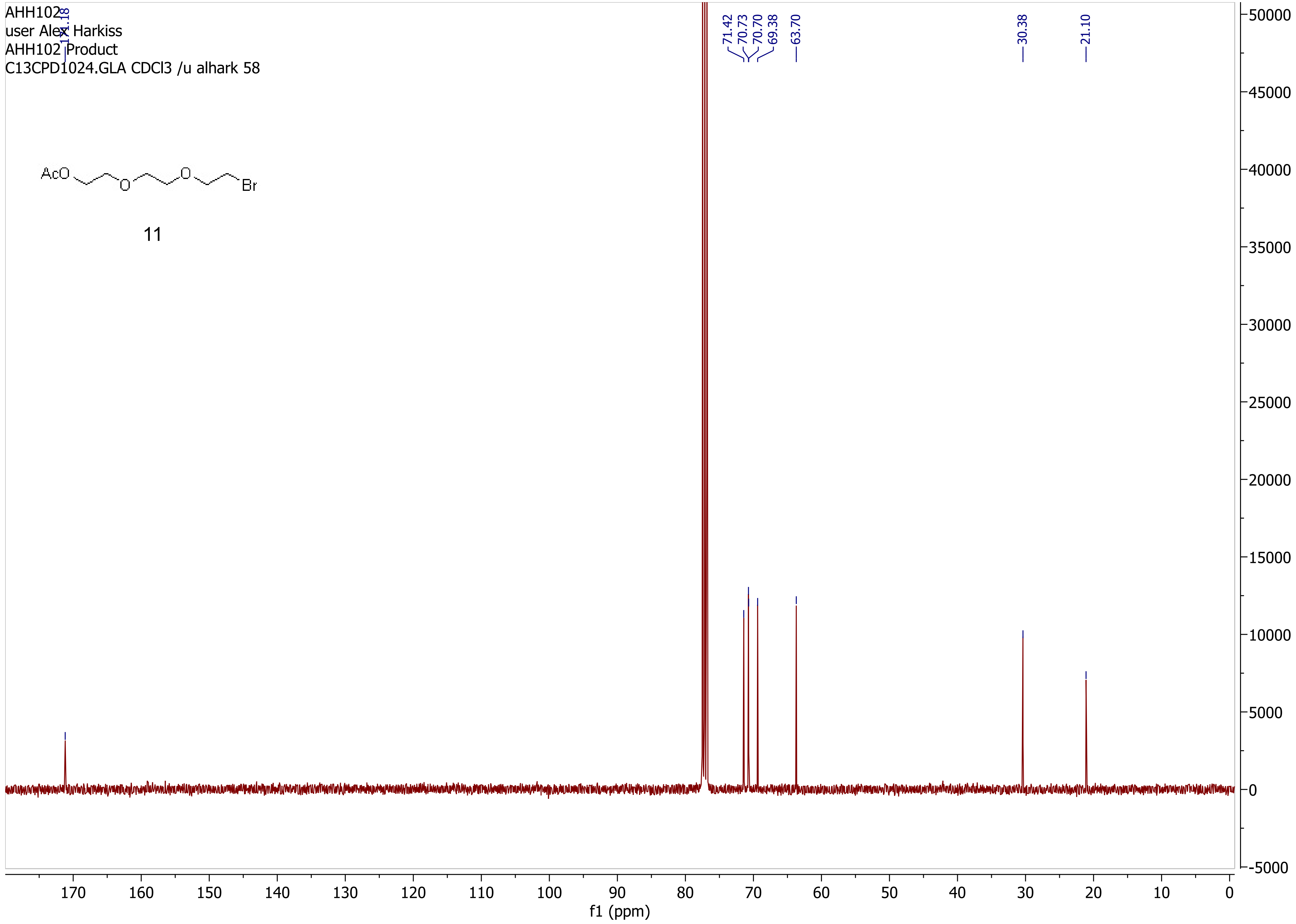
AHH102
user Alex Harkiss
AHH102 Product
C13CPD1024.GLA CDCl3 /u alhark 58
171.18
71.42
70.73
70.70
69.38
63.70
30.38
21.10
AcO
O
O
Br
11
f1 (ppm)

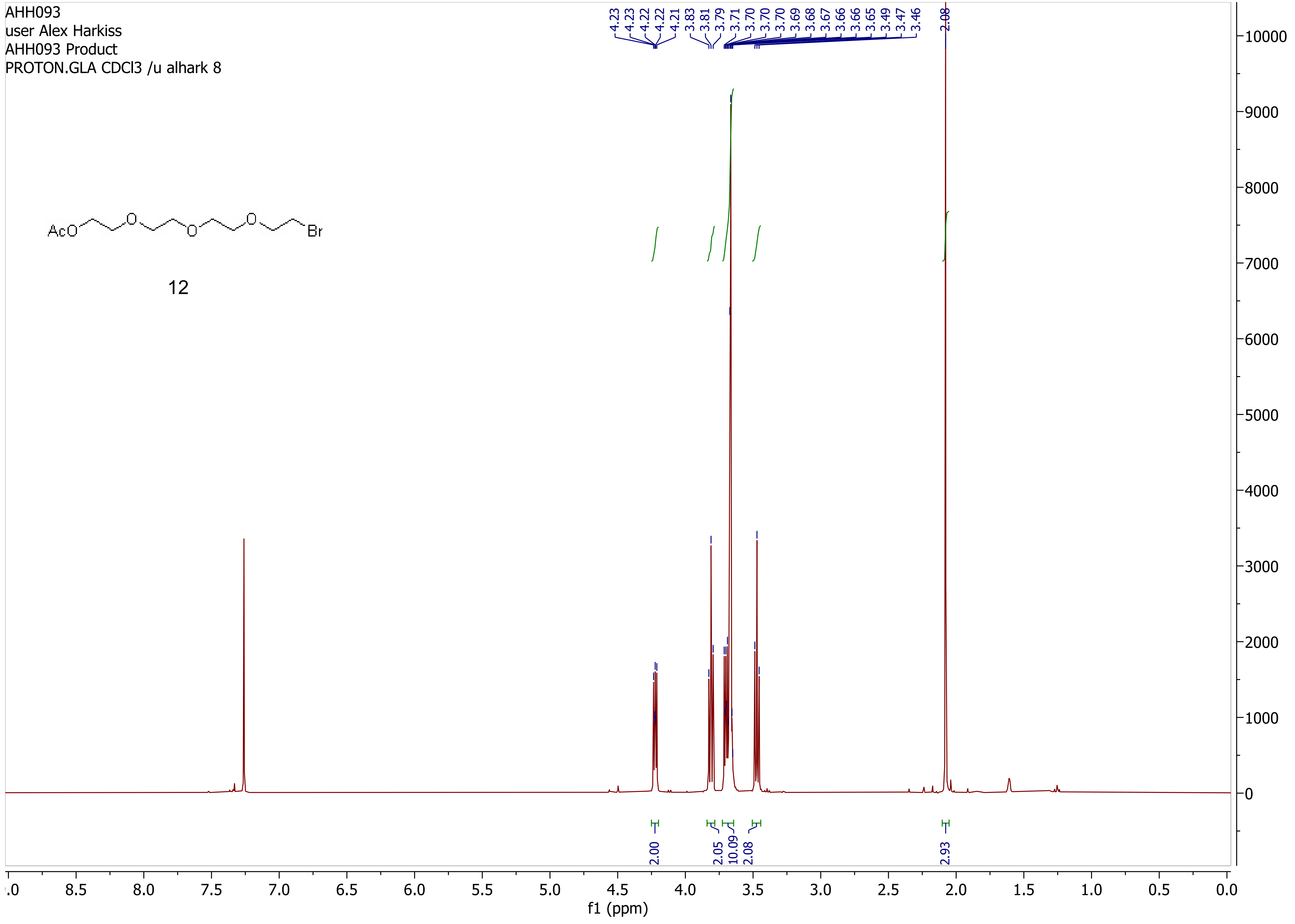
AHH093
user Alex Harkiss
AHH093 Product
PROTON.GLA CDCl3 /u alhark 8
AcO
O
O
O
Br
12
4.23
4.23
4.22
4.22
4.21
3.83
3.81
3.79
3.71
3.70
3.70
3.70
3.69
3.68
3.67
3.66
3.66
3.65
3.49
3.47
3.46
2.08
2.00
2.05
10.09
2.08
2.93
10000
9000
8000
7000
6000
5000
4000
3000
2000
1000
0
8.5
8.0
7.5
7.0
6.5
6.0
5.5
5.0
4.5
4.0
3.5
3.0
2.5
2.0
1.5
1.0
0.5
0.0
f1 (ppm)

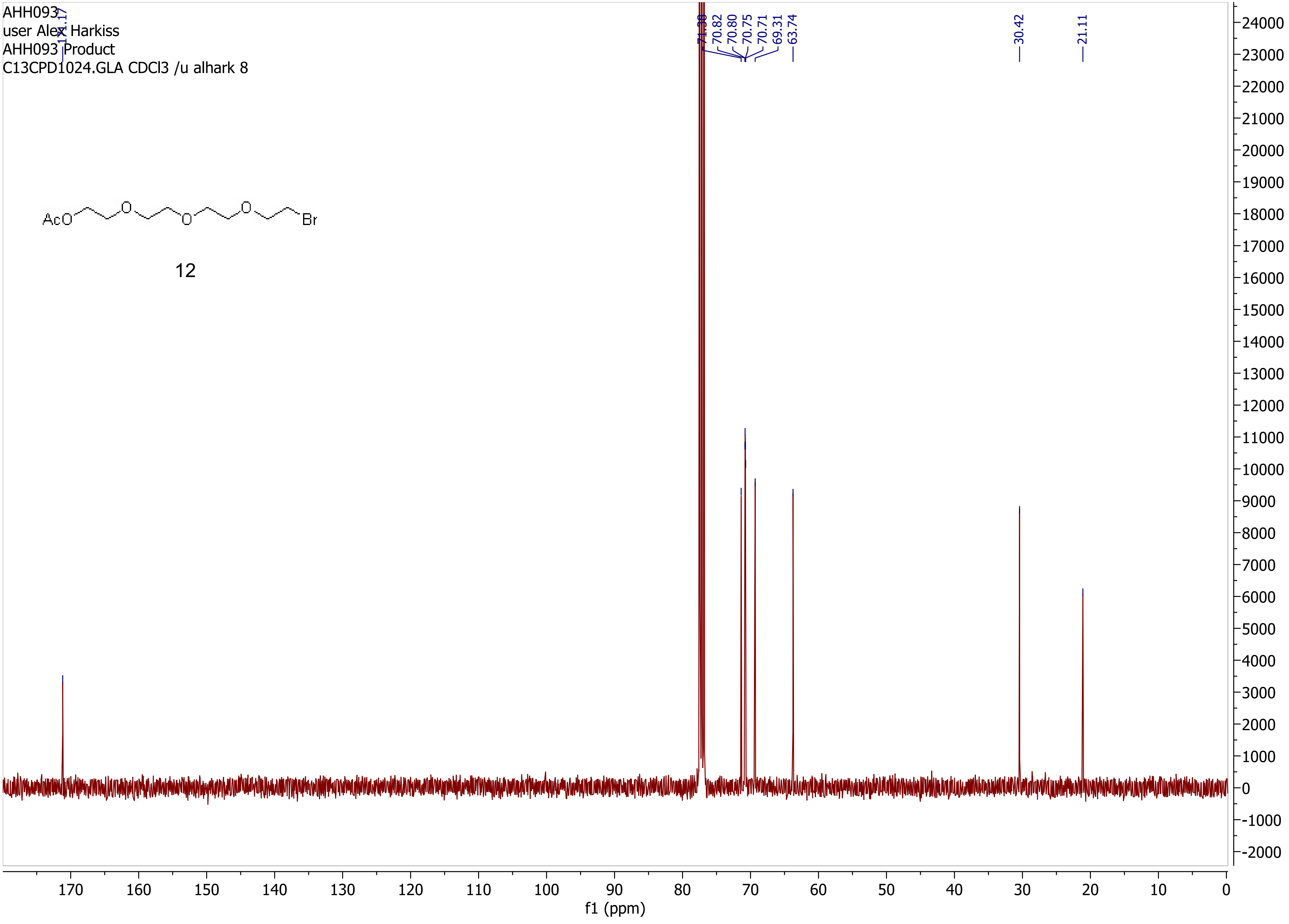

AHH093
user Alex Harkiss
AHH093 Product
C13CPD1024.GLA CDCl3 /u alhark 8
171.17
71.38
70.82
70.80
70.75
70.71
69.31
63.74
30.42
21.11
AcO
O
O
O
Br
12
f1 (ppm)

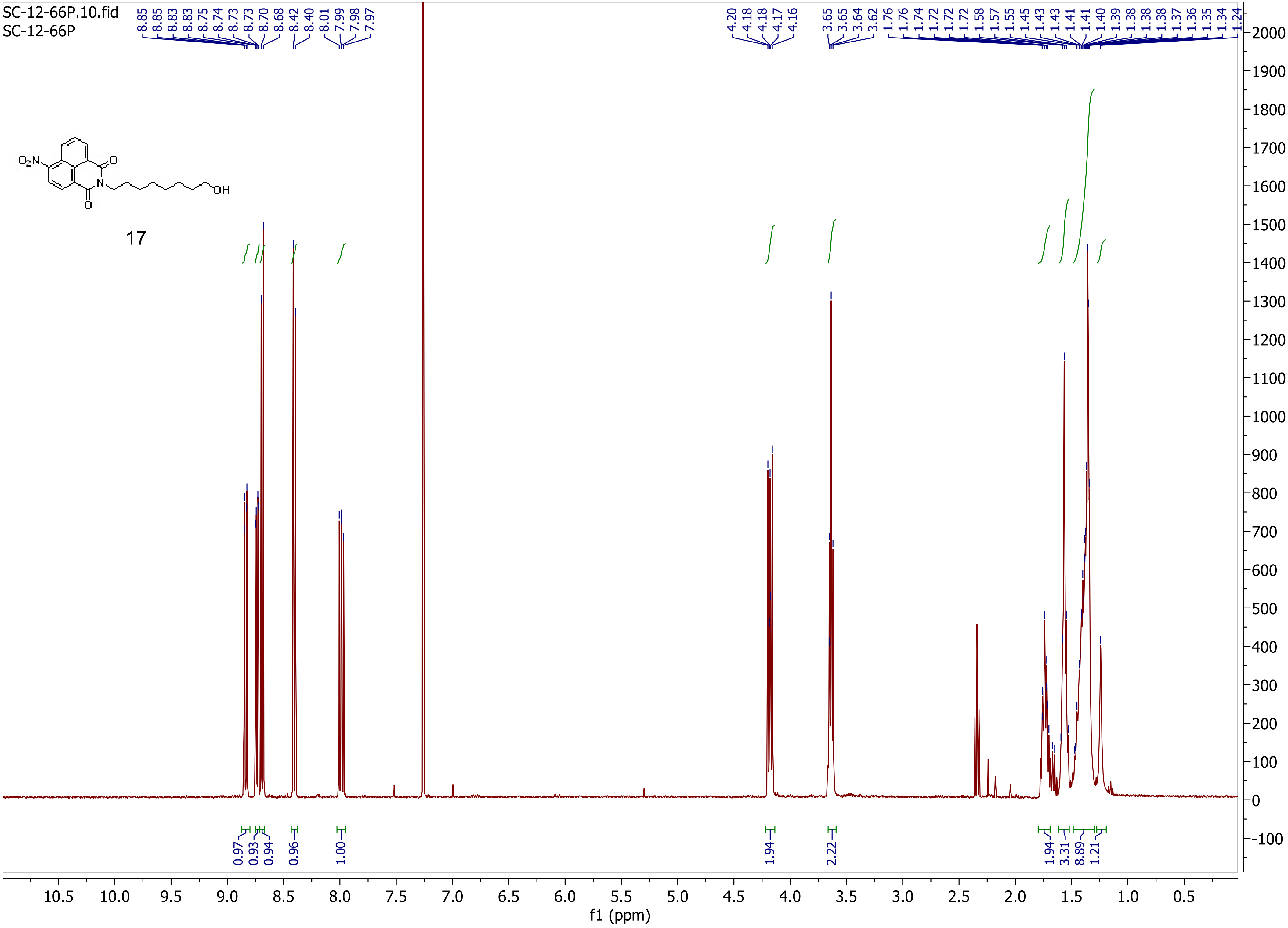
SC-12-66P.10.fid
SC-12-66P
8.85 8.85 8.83 8.83 8.75 8.74 8.73 8.73 8.70 8.68 8.42 8.40 8.01 7.99 7.98 7.97
4.20 4.18 4.18 4.17 4.16
3.65 3.65 3.64 3.62
1.76 1.76 1.74 1.72 1.72 1.72 1.58 1.57 1.55 1.45 1.43 1.43 1.41 1.41 1.40 1.39 1.38 1.38 1.37 1.36 1.35 1.34 1.24
O2N
O
N
OH
17
0.97 0.93 0.94 0.96 1.00 1.94 2.22 1.94 3.31 8.89 1.21
2000 1900 1800 1700 1600 1500 1400 1300 1200 1100 1000 900 800 700 600 500 400 300 200 100 0 -100
10.5 10.0 9.5 9.0 8.5 8.0 7.5 7.0 6.5 6.0 5.5 5.0 4.5 4.0 3.5 3.0 2.5 2.0 1.5 1.0 0.5
f1 (ppm)

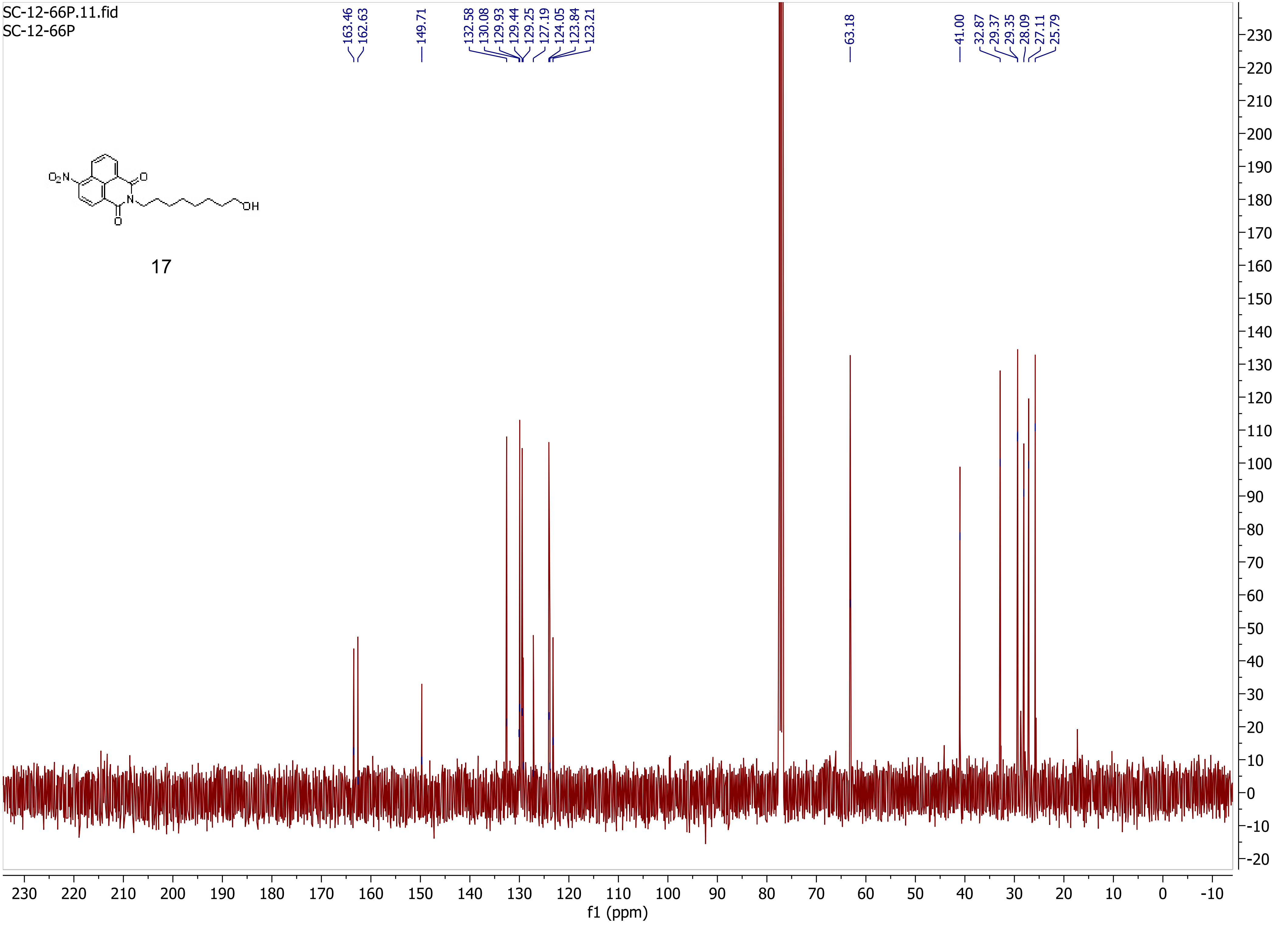
SC-12-66P.11.fid
SC-12-66P
163.46
162.63
149.71
132.58
130.08
129.93
129.44
129.25
127.19
124.05
123.84
123.21
63.18
41.00
32.87
29.37
29.35
28.09
27.11
25.79
O2N
O
N
OH
17
f1 (ppm)

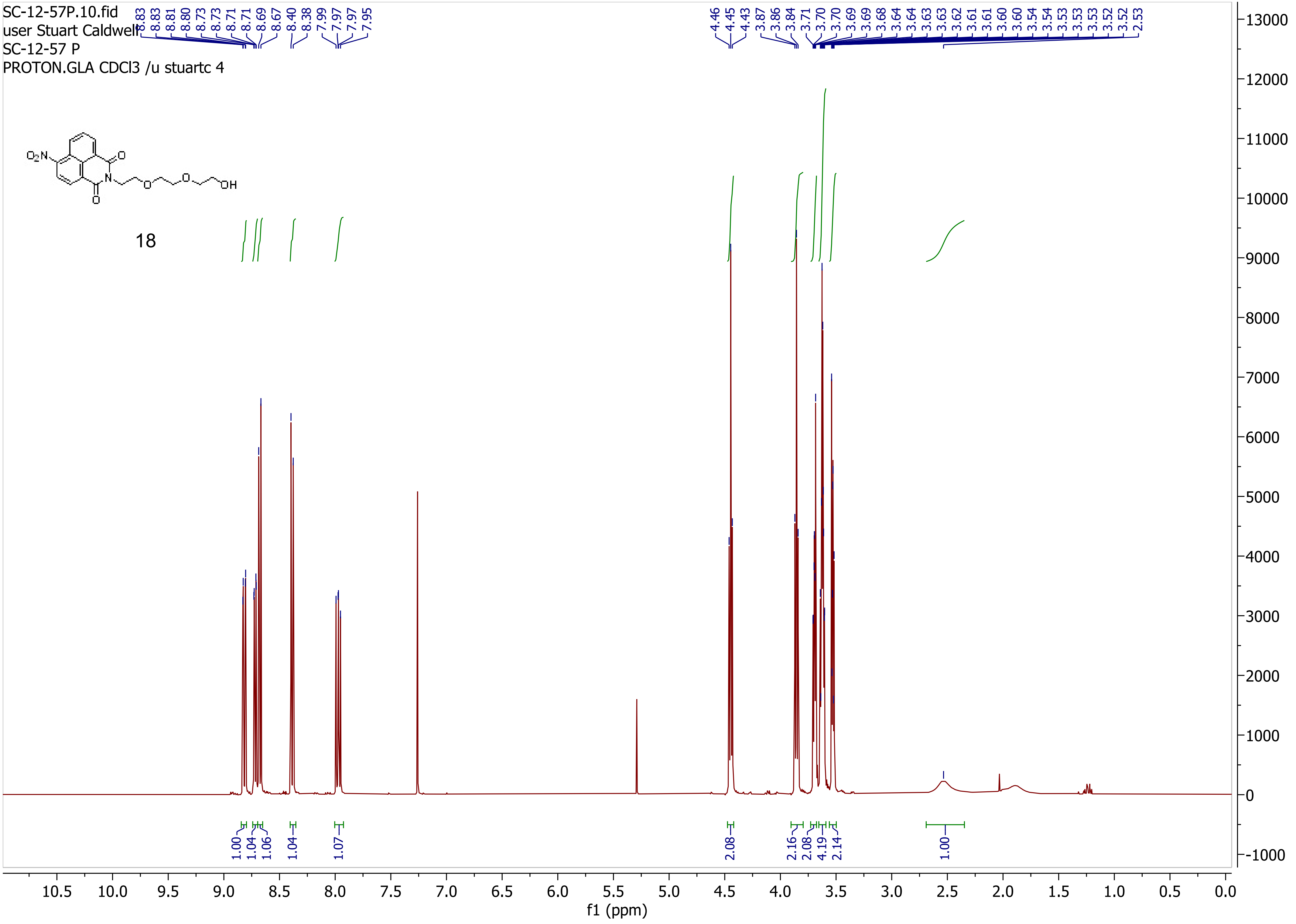
SC-12-57P.10.fid
user Stuart Caldwell
SC-12-57 P
PROTON.GLA CDCl3 /u stuartc 4
8.83 8.83 8.81 8.80 8.73 8.73 8.71 8.71 8.69 8.67 8.40 8.38 7.99 7.97 7.97 7.95
4.46 4.45 4.43 3.87 3.86 3.84 3.71 3.70 3.70 3.69 3.69 3.68 3.64 3.64 3.63 3.63 3.62 3.61 3.61 3.60 3.60 3.54 3.53 3.53 3.53 3.53 3.52 3.52 2.53
O_2N
O
N
O
O
O
OH
18
1.00 1.04 1.06 1.04 1.07 2.08 2.16 2.08 4.19 2.14 1.00
13000 12000 11000 10000 9000 8000 7000 6000 5000 4000 3000 2000 1000 0 -1000
10.5 10.0 9.5 9.0 8.5 8.0 7.5 7.0 6.5 6.0 5.5 5.0 4.5 4.0 3.5 3.0 2.5 2.0 1.5 1.0 0.5 0.0
f1 (ppm)

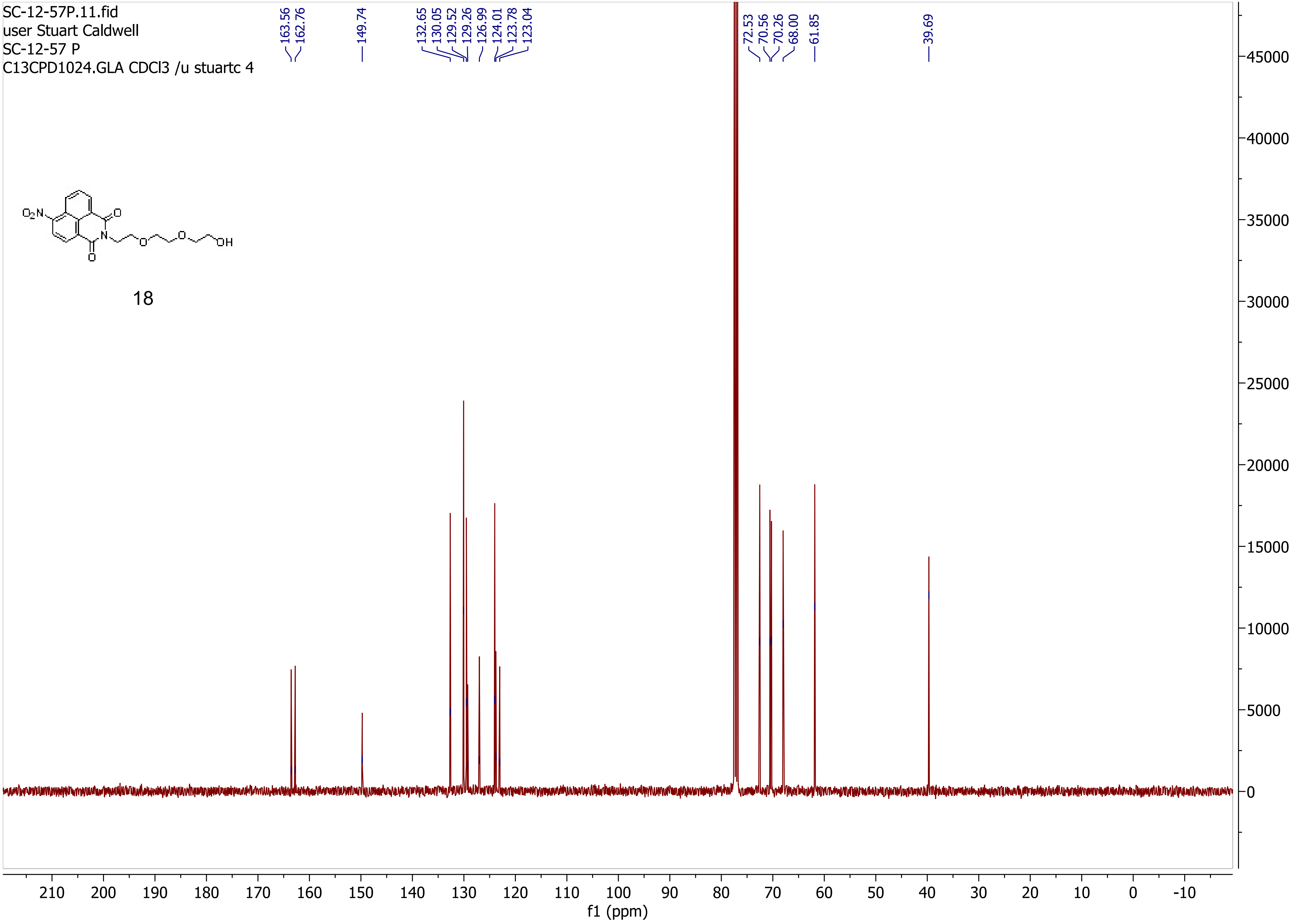
SC-12-57P.11.fid
user Stuart Caldwell
SC-12-57 P
C13CPD1024.GLA CDCl3 /u stuartc 4
163.56
162.76
149.74
132.65
130.05
129.52
129.26
126.99
124.01
123.78
123.04
72.53
70.56
70.26
68.00
61.85
39.69
O2N
N
O
O
O
O
OH
18
45000
40000
35000
30000
25000
20000
15000
10000
5000
0
210
200
190
180
170
160
150
140
130
120
110
100
90
80
70
60
50
40
30
20
10
0
-10
f1 (ppm)

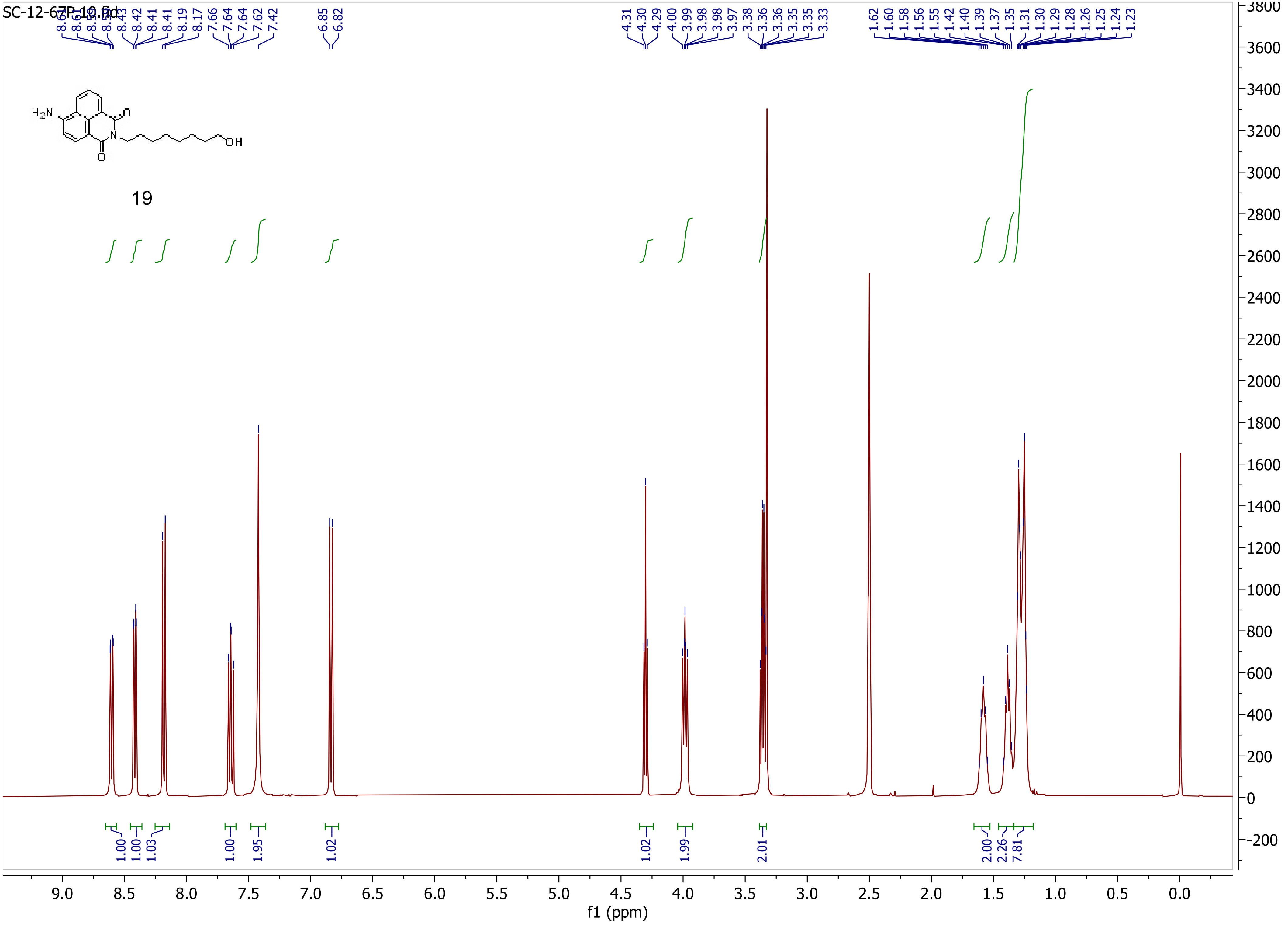
SC-12-67P-10.fid
8.43
8.42
8.41
8.41
8.19
8.17
7.66
7.64
7.64
7.62
7.42
6.85
6.82
4.31
4.30
4.29
4.00
3.99
3.98
3.98
3.97
3.38
3.36
3.36
3.35
3.35
3.33
1.62
1.60
1.58
1.56
1.55
1.42
1.40
1.39
1.37
1.35
1.31
1.30
1.29
1.28
1.26
1.25
1.24
1.23
H2N
O
N
OH
O
1.00
1.00
1.03
1.00
1.95
1.02
1.02
1.99
2.01
2.00
2.26
7.81
9.0
8.5
8.0
7.5
7.0
6.5
6.0
5.5
5.0
4.5
4.0
3.5
3.0
2.5
2.0
1.5
1.0
0.5
0.0
f1 (ppm)
3800
3600
3400
3200
3000
2800
2600
2400
2200
2000
1800
1600
1400
1200
1000
800
600
400
200
0
-200

19

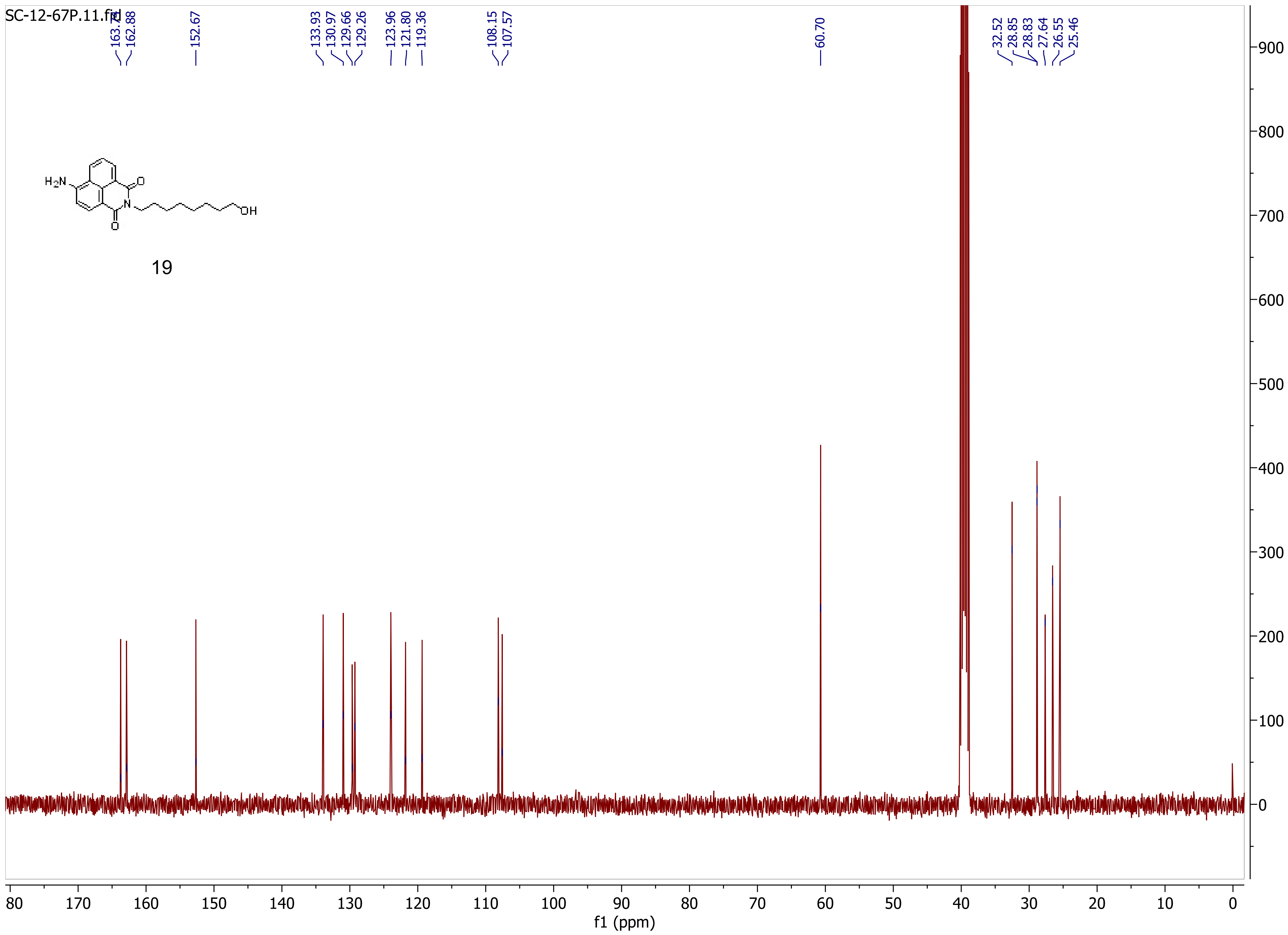
SC-12-67P.11.fid
162.88
152.67
133.93
130.97
129.66
129.26
123.96
121.80
119.36
108.15
107.57
60.70
32.52
28.85
28.83
27.64
26.55
25.46
H2N
O
N
OH
O
19
f1 (ppm)

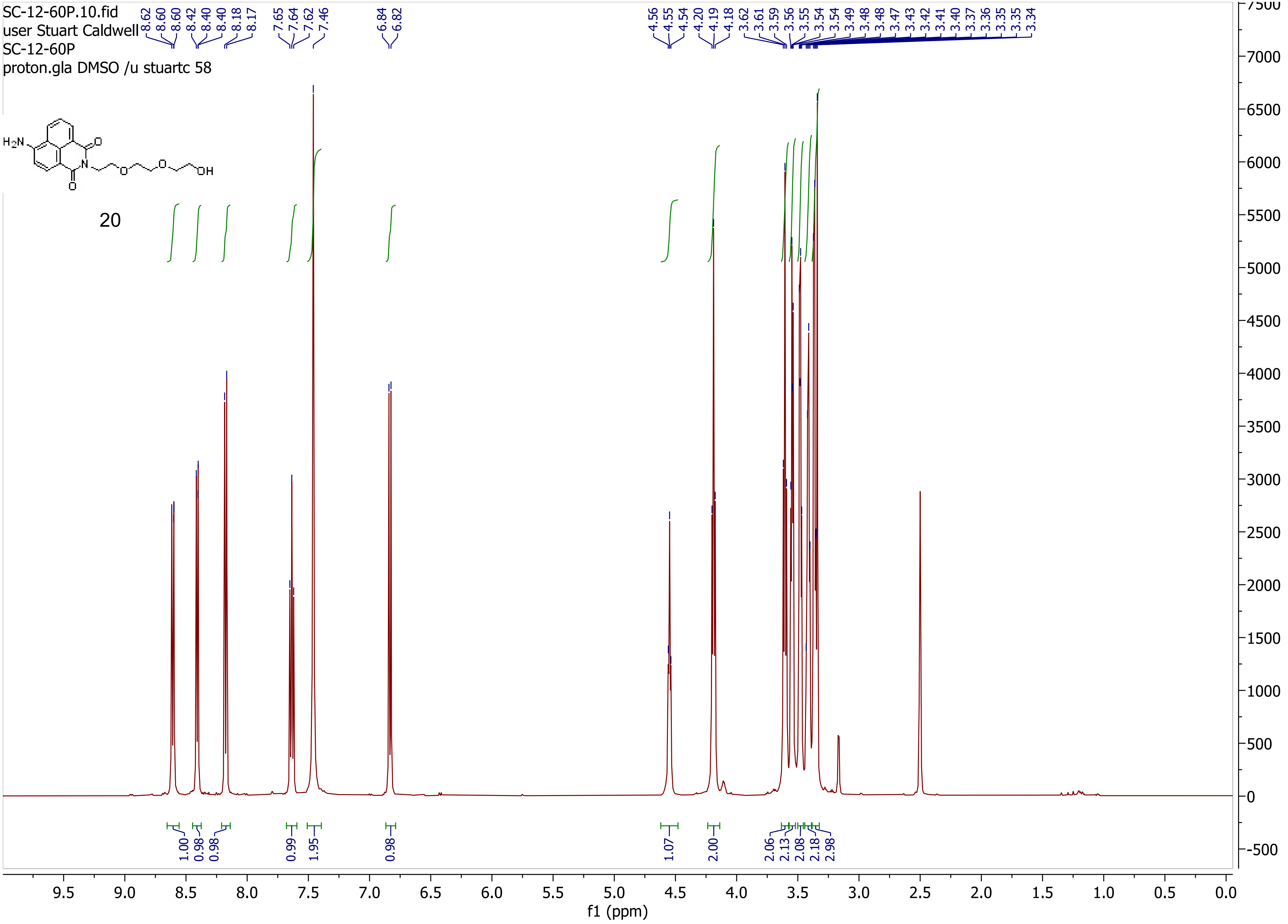
SC-12-60P.10.fid
user Stuart Caldwell
SC-12-60P
proton.gla DMSO /u stuartc 58
8.62 8.60 8.60 8.42 8.40 8.40 8.18 8.17 7.65 7.64 7.62 7.46 6.84 6.82
4.56 4.55 4.54 4.20 4.19 4.18 3.62 3.61 3.59 3.56 3.55 3.54 3.54 3.49 3.48 3.48 3.47 3.43 3.42 3.41 3.40 3.37 3.36 3.35 3.35 3.34
H2N
O
N
OH
20
1.00 0.98 0.98 0.99 1.95 0.98 1.07 2.00 2.06 2.13 2.08 2.18 2.98
f1 (ppm)

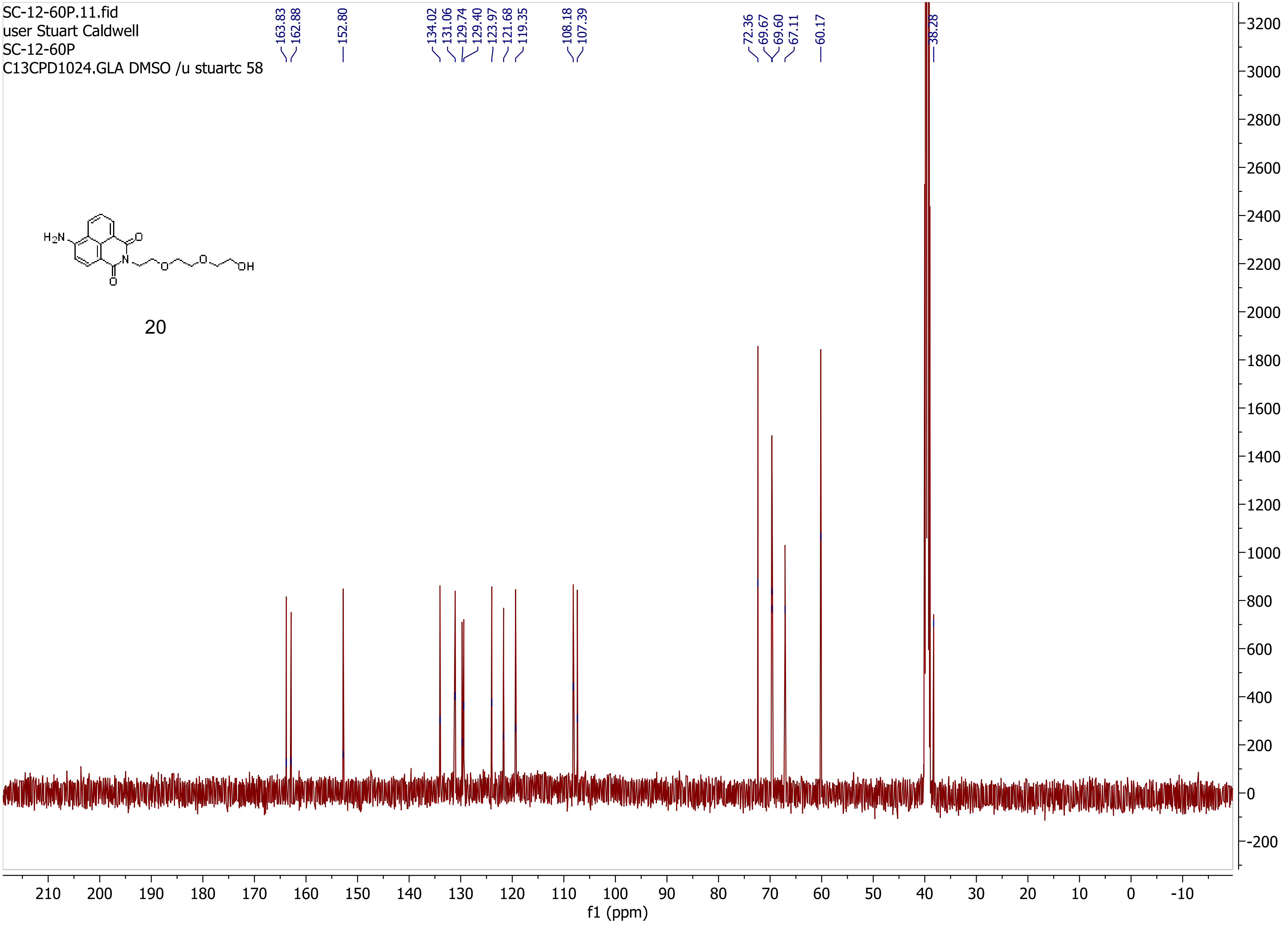
SC-12-60P.11.fid
user Stuart Caldwell
SC-12-60P
C13CPD1024.GLA DMSO /u stuartc 58
163.83
162.88
152.80
134.02
131.06
129.74
129.40
123.97
121.68
119.35
108.18
107.39
72.36
69.67
69.60
67.11
60.17
38.28
H2N
O
N
O
O
O
OH
20
f1 (ppm)
210 200 190 180 170 160 150 140 130 120 110 100 90 80 70 60 50 40 30 20 10 0 -10
3200 3000 2800 2600 2400 2200 2000 1800 1600 1400 1200 1000 800 600 400 200 0 -200

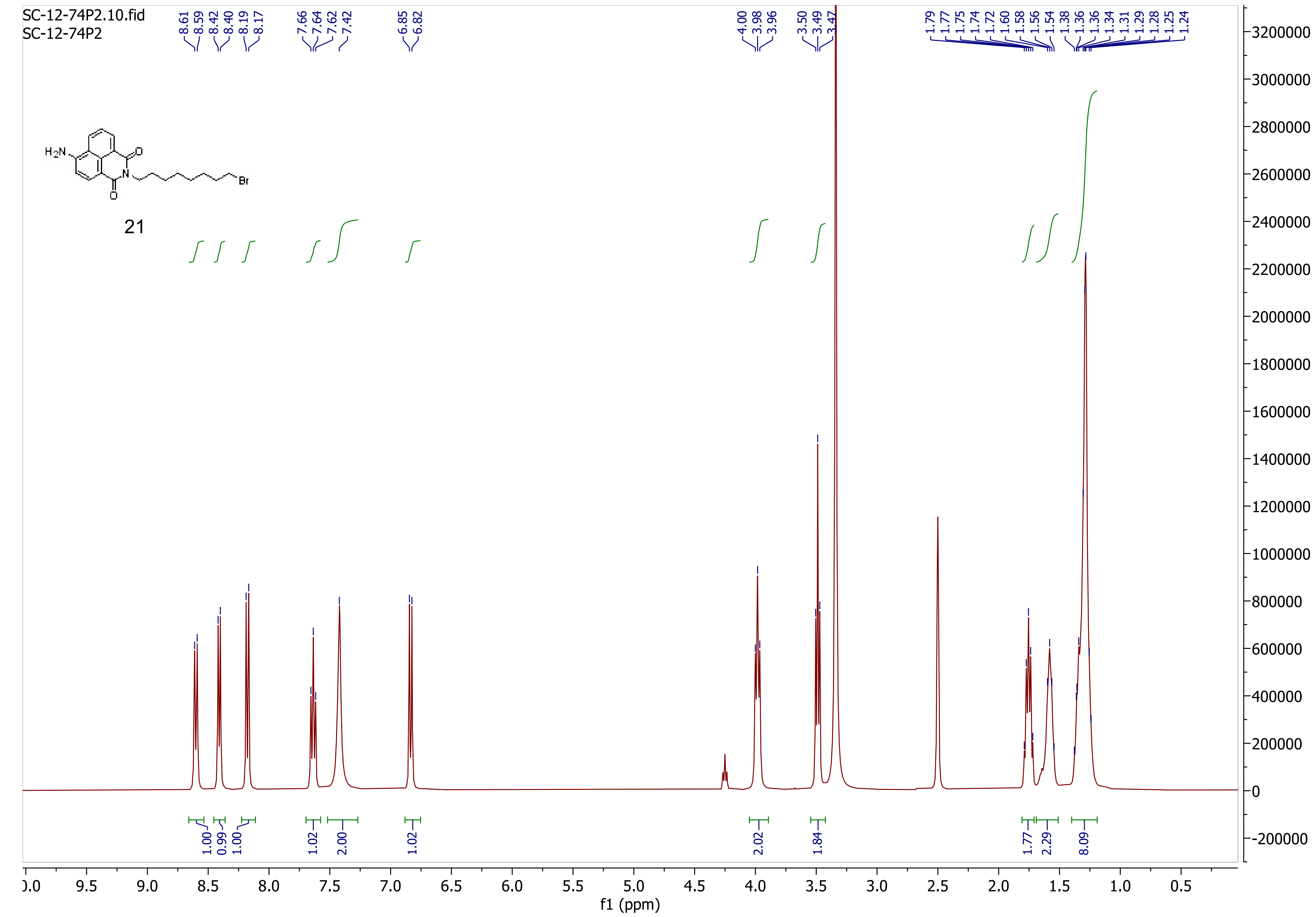
SC-12-74P2.10.fid
SC-12-74P2
8.61 8.59 8.42 8.40 8.19 8.17
7.66 7.64 7.62 7.42
6.85 6.82
4.00 3.98 3.96
3.50 3.49 3.47
1.79 1.77 1.75 1.74 1.72 1.60 1.58 1.56 1.54 1.38 1.36 1.36 1.34 1.31 1.29 1.28 1.25 1.24
H2N
O
N
Br
O
21
1.00 0.99 1.00 1.02 2.00 1.02 2.02 1.84 1.77 2.29 8.09
3200000 3000000 2800000 2600000 2400000 2200000 2000000 1800000 1600000 1400000 1200000 1000000 800000 600000 400000 200000 0 -200000
9.5 9.0 8.5 8.0 7.5 7.0 6.5 6.0 5.5 5.0 4.5 4.0 3.5 3.0 2.5 2.0 1.5 1.0 0.5
f1 (ppm)

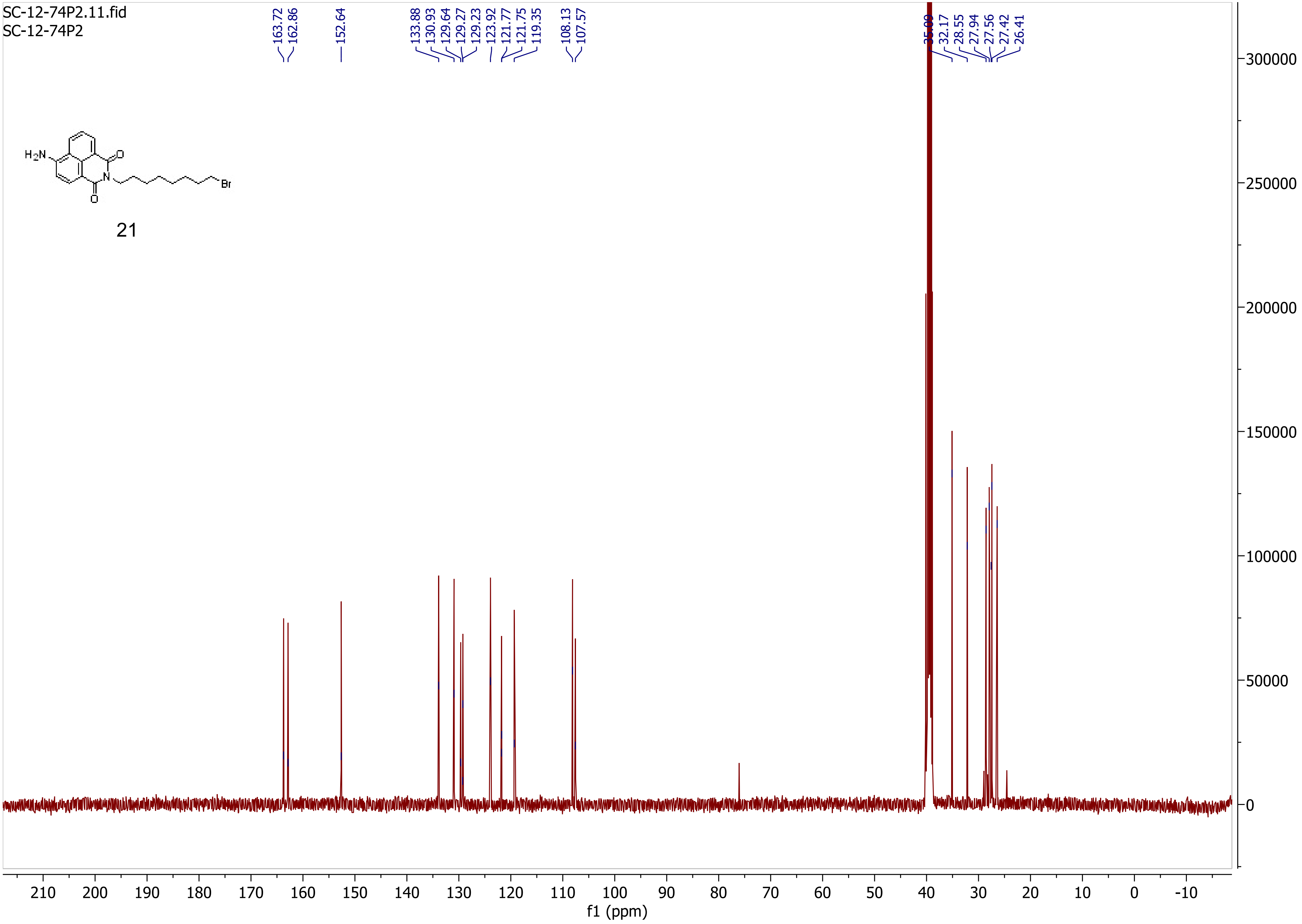
SC-12-74P2.11.fid
SC-12-74P2
163.72
162.86
152.64
133.88
130.93
129.64
129.27
129.23
123.92
121.77
121.75
119.35
108.13
107.57
35.09
32.17
28.55
27.94
27.56
27.42
26.41
H_2N
N
O
O
Br
21
300000
250000
200000
150000
100000
50000
0
210 200 190 180 170 160 150 140 130 120 110 100 90 80 70 60 50 40 30 20 10 0 -10
f1 (ppm)

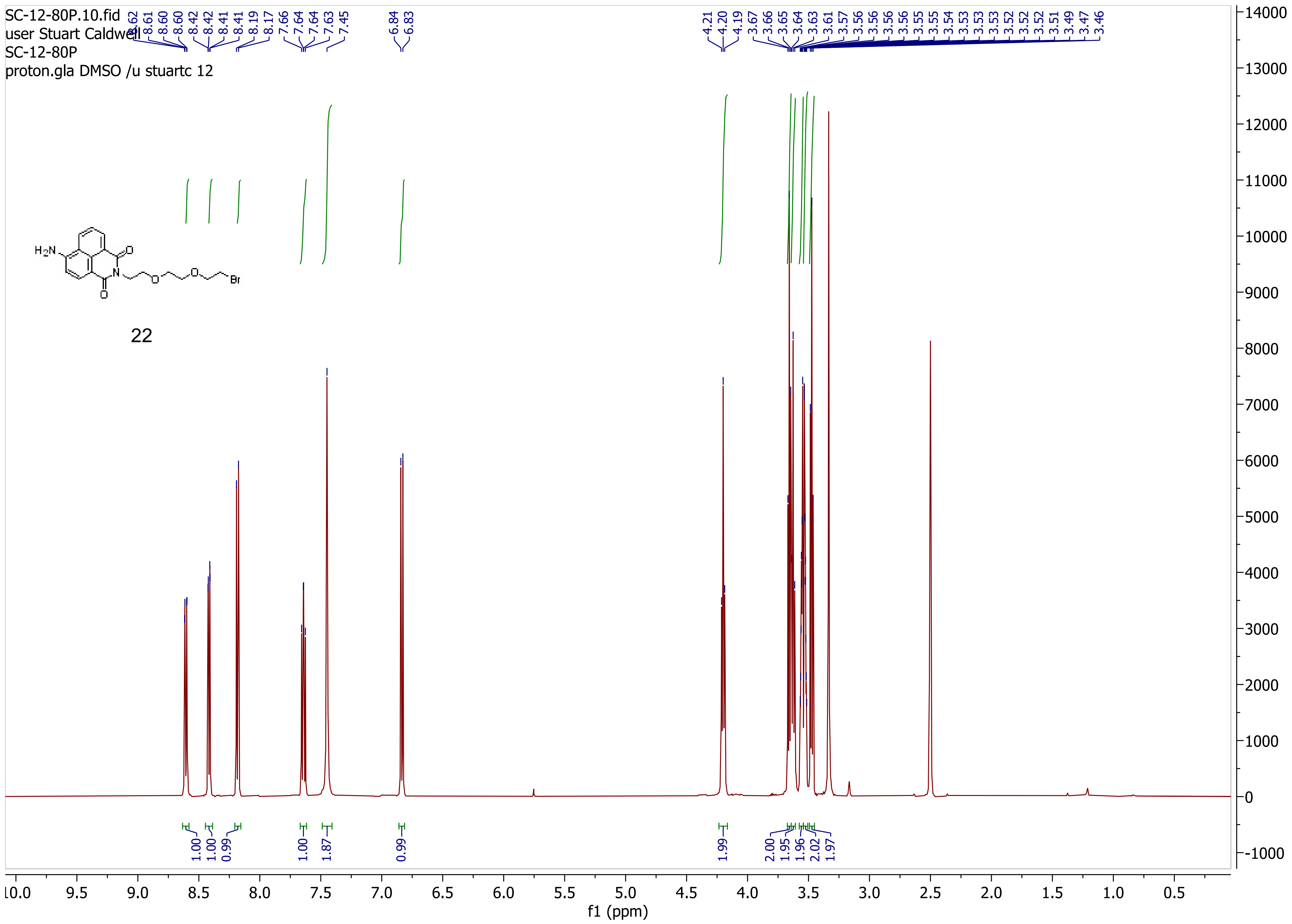
SC-12-80P.10.fid
user Stuart Caldwell
SC-12-80P
proton.gla DMSO /u stuartc 12
8.62 8.61 8.60 8.60 8.42 8.42 8.41 8.41 8.19 8.17 7.66 7.64 7.64 7.63 7.45
6.84 6.83
4.21 4.20 4.19 3.67 3.66 3.65 3.64 3.63 3.61 3.57 3.56 3.56 3.56 3.55 3.55 3.54 3.53 3.53 3.53 3.52 3.52 3.51 3.49 3.47 3.46
H2N
O
N
Br
22
1.00 1.00 0.99 1.00 1.87 0.99 1.99 2.00 1.95 1.96 2.02 1.97
14000 13000 12000 11000 10000 9000 8000 7000 6000 5000 4000 3000 2000 1000 0 -1000
10.0 9.5 9.0 8.5 8.0 7.5 7.0 6.5 6.0 5.5 5.0 4.5 4.0 3.5 3.0 2.5 2.0 1.5 1.0 0.5
f1 (ppm)

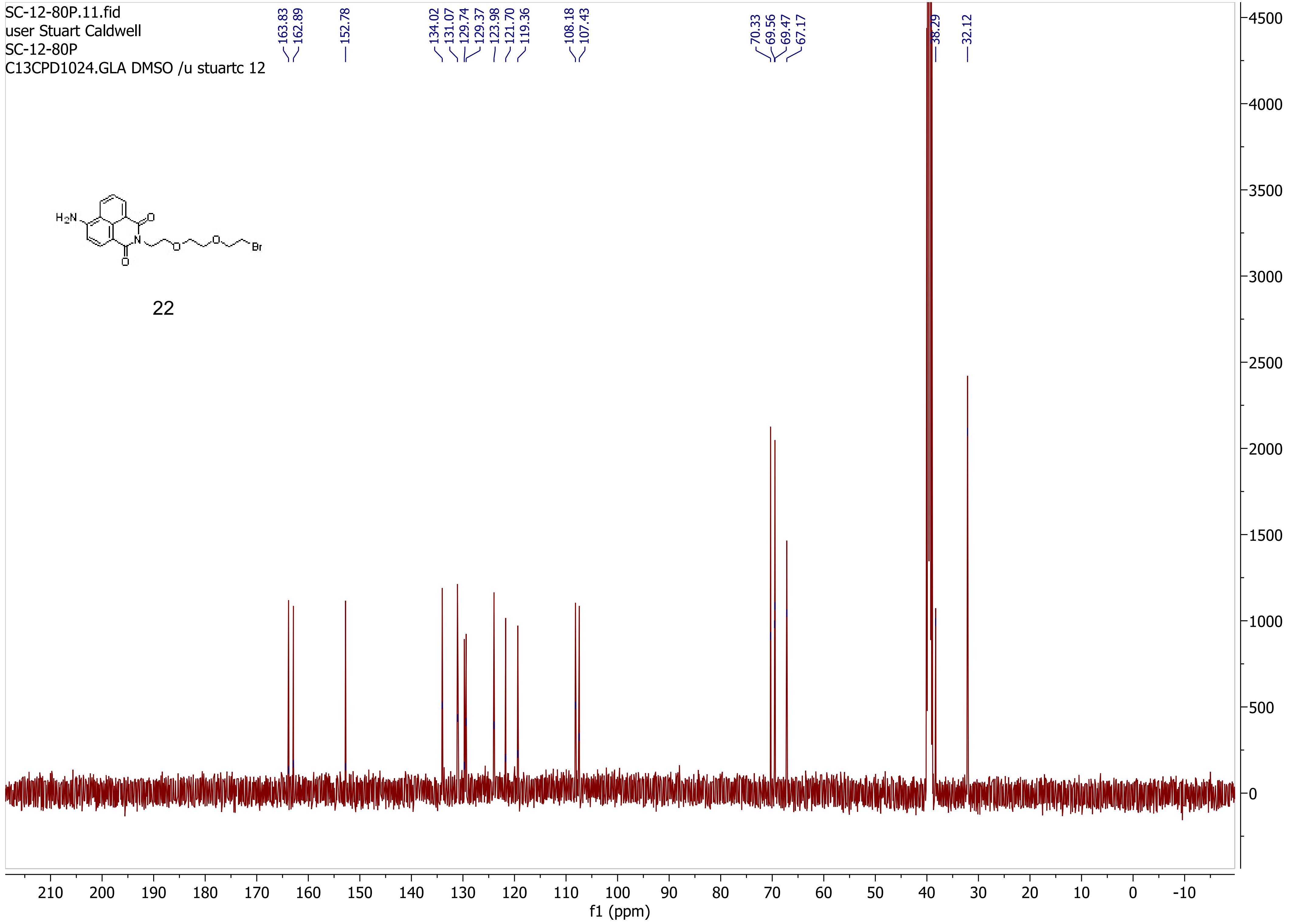
SC-12-80P.11.fid
user Stuart Caldwell
SC-12-80P
C13CPD1024.GLA DMSO /u stuartc 12
163.83
162.89
152.78
134.02
131.07
129.74
129.37
123.98
121.70
119.36
108.18
107.43
70.33
69.56
69.47
67.17
38.29
32.12
22
4500
4000
3500
3000
2500
2000
1500
1000
500
0
210 200 190 180 170 160 150 140 130 120 110 100 90 80 70 60 50 40 30 20 10 0 -10
f1 (ppm)

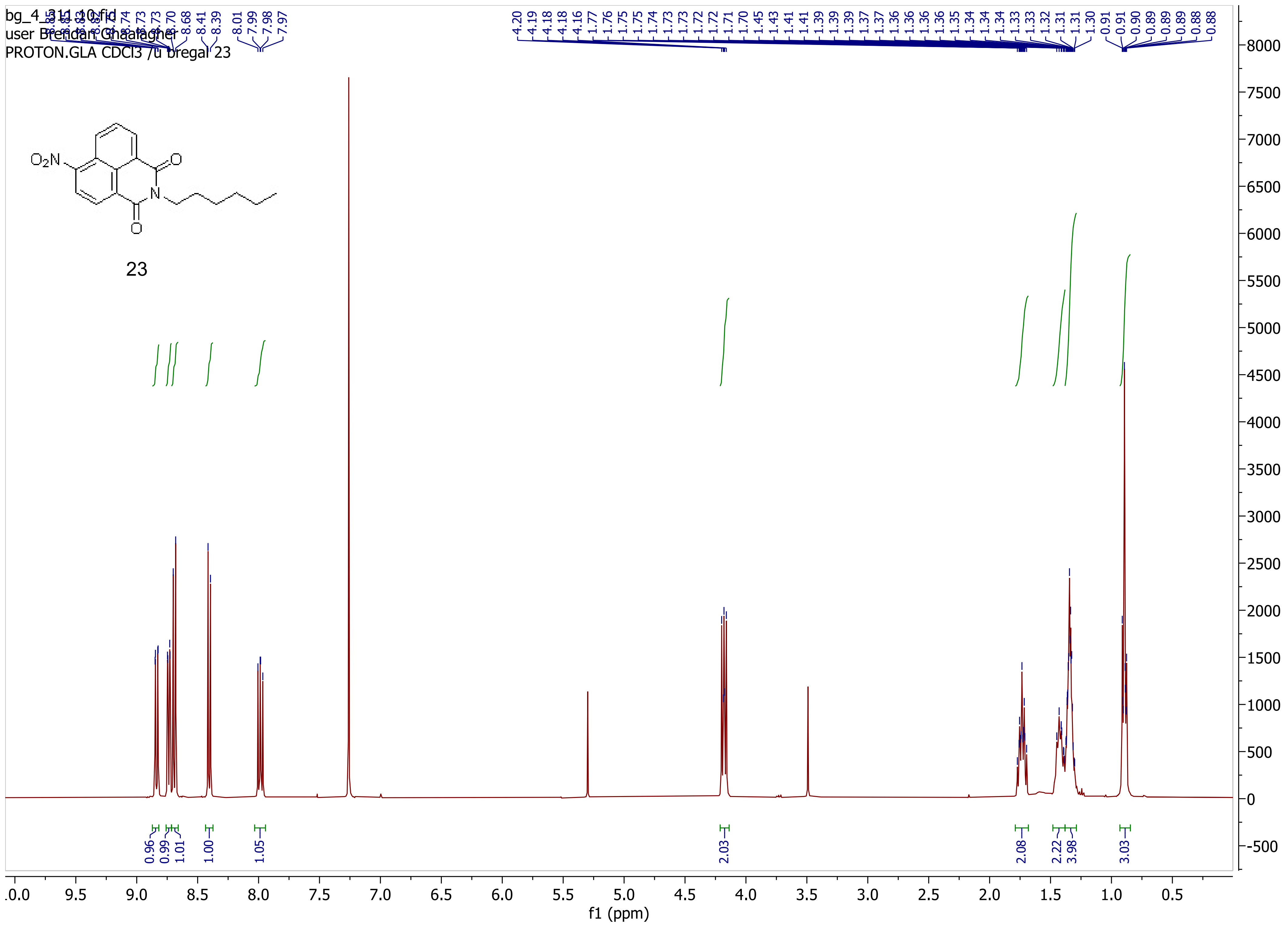
bg_4_311.10.fid
user Brendan Gallagher
PROTON.GLA CDCl3 /u bregal 23
8.70
8.68
8.41
8.39
8.01
7.99
7.98
7.97
4.20
4.19
4.18
4.16
1.77
1.76
1.75
1.75
1.74
1.73
1.73
1.72
1.72
1.71
1.70
1.45
1.43
1.41
1.41
1.39
1.39
1.39
1.37
1.37
1.36
1.36
1.36
1.35
1.34
1.34
1.34
1.33
1.33
1.32
1.31
1.31
1.30
0.91
0.91
0.90
0.89
0.89
0.89
0.88
0.88
O2N
O
N
O
23
0.96
0.99
1.01
1.00
1.05
2.03
2.08
2.22
3.98
3.03
f1 (ppm)

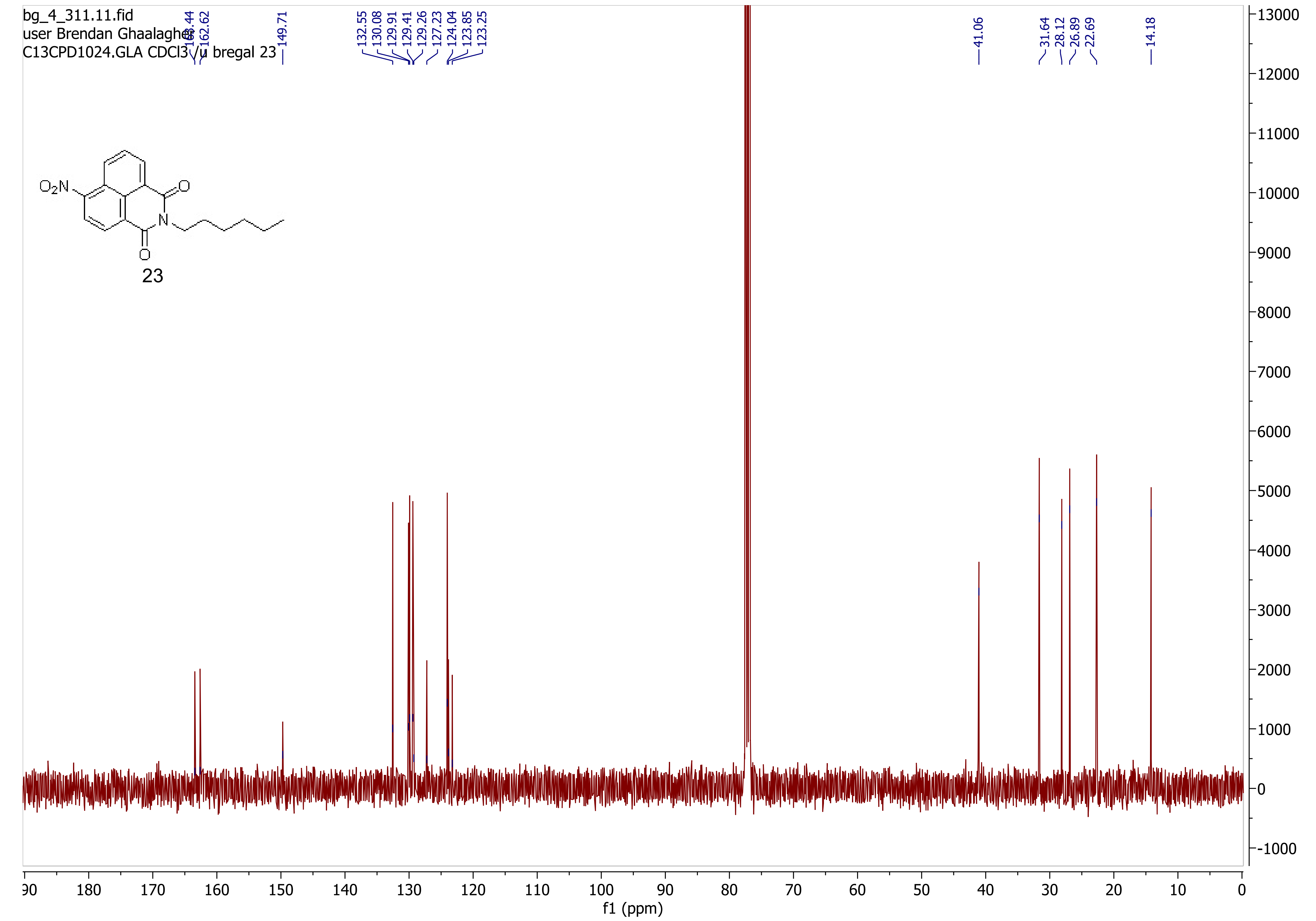

bg_4_311.11.fid
user Brendan Ghaalagher
C13CPD1024.GLA CDCl3 \u bregal 23
162.62
149.71
132.55
130.08
129.91
129.41
129.26
127.23
124.04
123.85
123.25
41.06
31.64
28.12
26.89
22.69
14.18
O2N
O
N
O
23
f1 (ppm)
